# Benzophosphol-3-yl Triflates as Precursors of 1,3-Diarylbenzophosphole Oxides

**DOI:** 10.1021/acs.joc.2c02355

**Published:** 2023-06-05

**Authors:** Agnieszka Brzyska, Sebastian Majewski, Łukasz Ponikiewski, Monika Zubik-Duda, Agnieszka Lipke, Agnieszka Gładysz-Płaska, Sylwia Sowa

**Affiliations:** † Jerzy Haber Institute of Catalysis and Surface Chemistry, Polish Academy of Sciences, 8 Niezapominajek St., Krakow PL-30-239, Poland; ‡ Department of Organic Chemistry and Crystallochemistry, Faculty of Chemistry, Institute of Chemical Sciences, Maria Curie-Sklodowska University in Lublin, 33 Gliniana St., Lublin PL-20-614, Poland; § Department of Inorganic Chemistry, Faculty of Chemistry, Gdańsk University of Technology, 11/12 G. Narutowicza St., Gdańsk PL-80-233, Poland; ∥ Department of Biophysics, Institute of Physics, Maria Curie-Sklodowska University in Lublin, PL-20-031 Lublin, Poland; ⊥ Institute of Chemical Sciences, Maria Curie-Sklodowska University in Lublin, 2/9 M. Curie-Sklodowska sq., Lublin PL-20-031, Poland; # Department of Inorganic Chemistry, Faculty of Chemistry, Institute of Chemical Sciences, Maria Curie-Sklodowska University in Lublin, 2/13-15A M. Curie-Sklodowska sq., Lublin PL-20-031, Poland

## Abstract

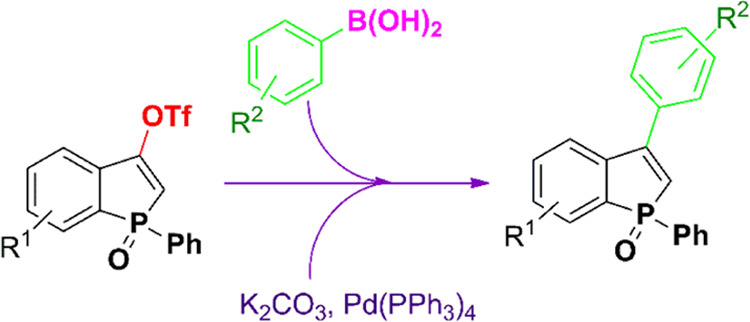

A simple method for the synthesis of 3-arylbenzophosphole oxides under Suzuki–Miyaura coupling conditions has been presented. It employs benzophosphol-3-yl triflate starting materials which, prior to our work, had not been used for the synthesis of 3-arylbenzophosphole oxides. The reactions proceed over 24 h and provide a library of 3-arylbenzophosphole oxides. The synthetic access to the benzophosphol-3-yl triflates has been improved. The preliminary photophysical properties of some 3-arylbenzophosphole oxides have been investigated by absorption and emission measurements. The theoretical calculations were performed to establish structure–property relationships.

## Introduction

**Scheme 1:**
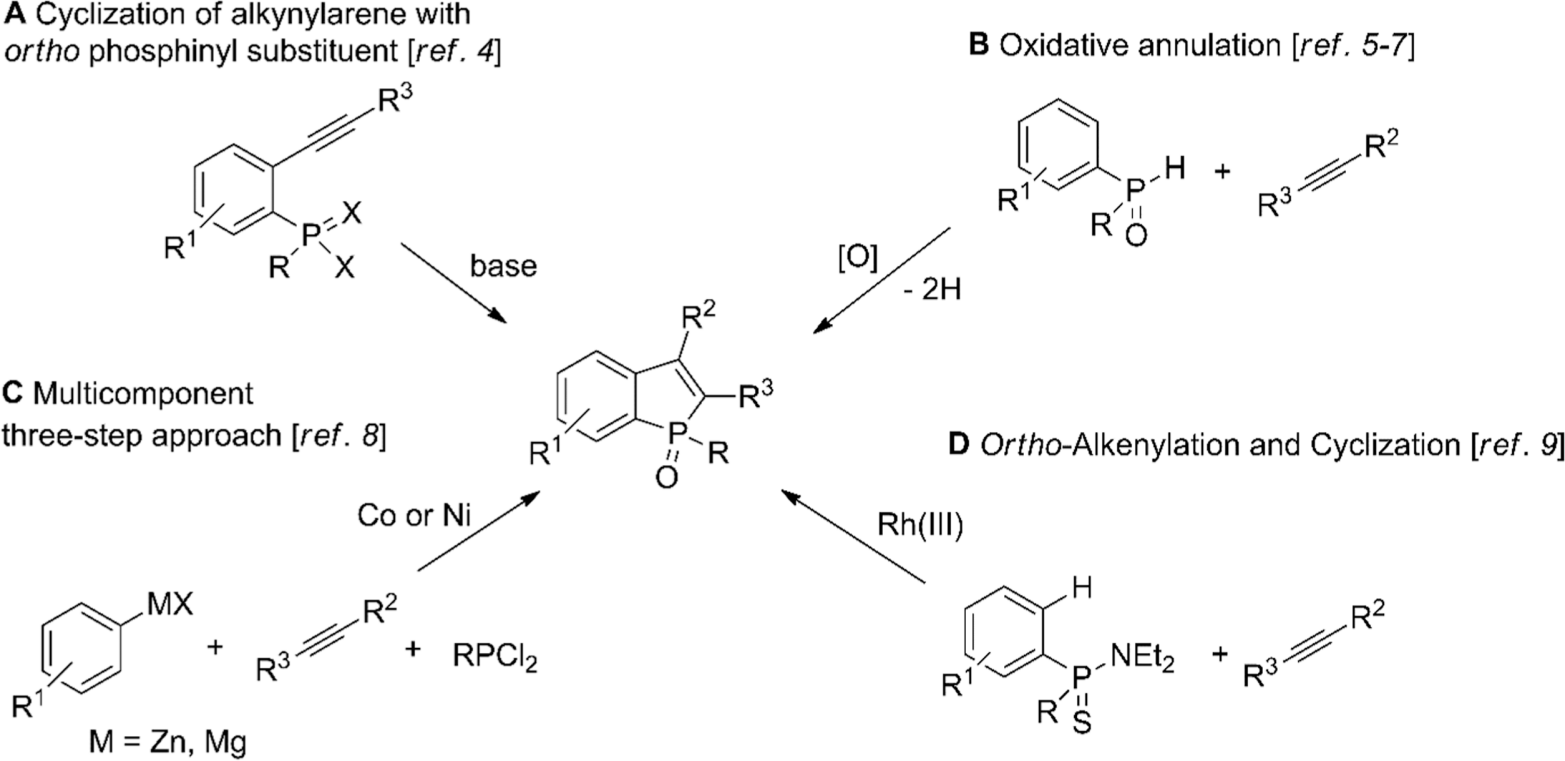
Previous Preparations of 2,3-Diarylbenzophoshole Oxides

Benzo[*b*]phosphole oxides have recently become important in the chemistry of organic materials because they have well-established semiconducting, fluorescent, and coordinating properties that have led to uses in processes as diverse as organic electronics, bioimaging, coordination chemistry, and catalysis.^1^ Historically, however, they have been much less thoroughly explored than many other heterocyclic compounds.^2^ Conventional syntheses of benzo[*b*]phosphole rings generally involve improvements to the protocol pioneered by Winter and Butters,^3^ and rely on base-mediated intramolecular cyclization of (2-alkynylphenyl)phenylarylphosphine derivatives (Scheme 1A).^4^ Modern routes to benzophospholes that are decorated with aryl substituents can be divided into several classes (Scheme 1B–D). The first is based on oxidative annulation in the presence of a metal-based oxidant (silver,^5^ manganese,^5a,5b^ copper^6^ compounds, or others^7^) (Scheme 1B). A second method involves a one-pot multicomponent reaction using aryl organozinc or Grignard reagents (Scheme 1C).^8^ A third approach, proposed by Miura and Satoh in 2016, involves *ortho*-alkenylation of arylthiophosphinamides (Scheme 1D).^9^ Each of these methods tolerates some degree of functionalization in the benzene ring of benzophosphole, and they have given access to benzo[*b*]phospholes substituted at both 2- and 3-positions (Scheme 1). It is noteworthy that these methods are most effective in the preparations of 2,3-diarylbenzophosphole oxides. In turn, the application of radical addition/cyclization of diaryl(arylethynyl)phosphine with alkanes is limited to the formation of a benzophosphole core possessing an alkyl substituent at the 2-positions.^10^

**Scheme 2:**
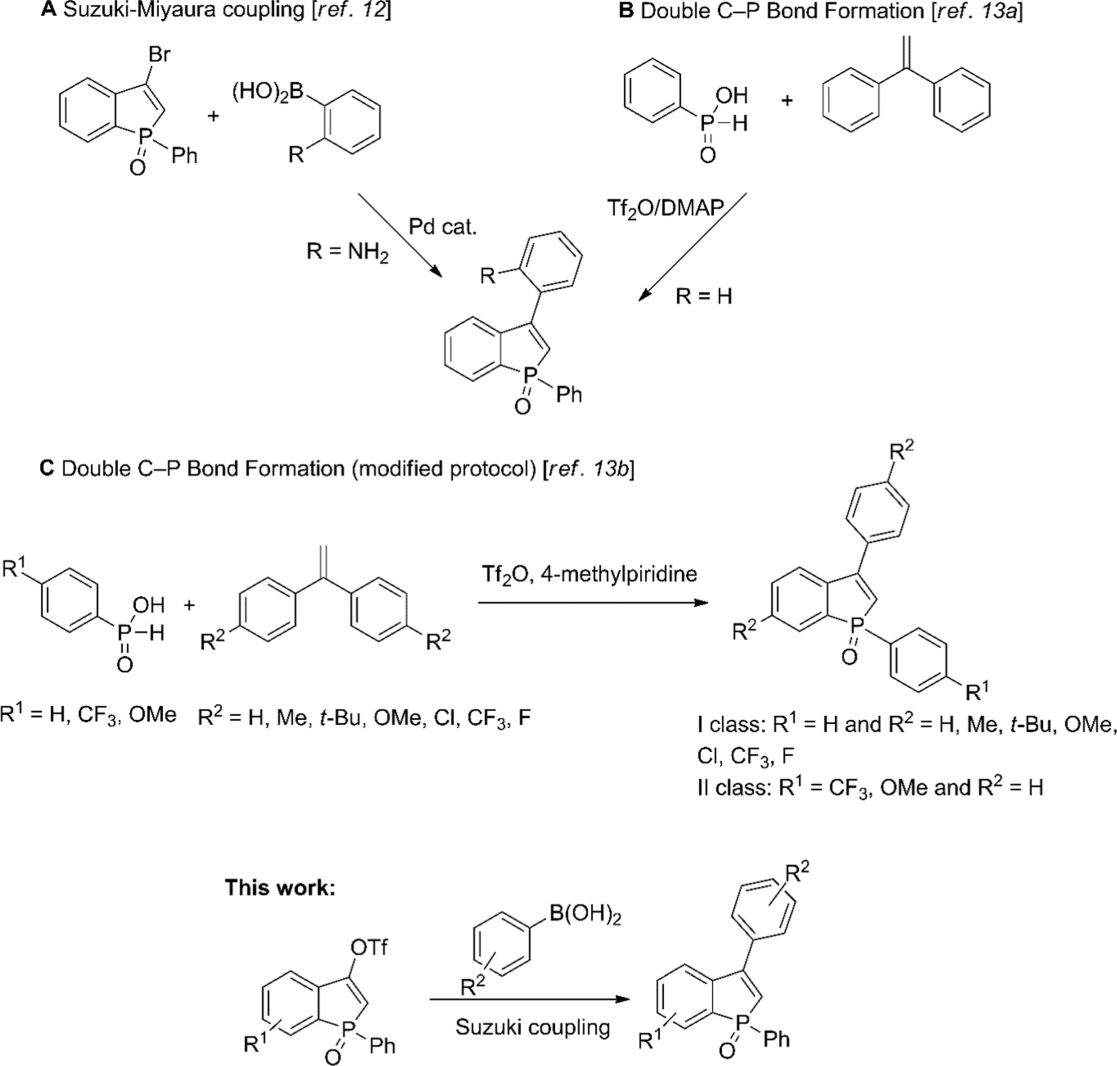
Examples of Preparations of 3-Arylbenzophoshole Oxides

Benzophosphole oxides having a single ring substituent have received much less attention^4c,4d,11^ and, to the best of our knowledge, only two methods for preparing 3-arylbenzophosphole oxides have been reported to date (Scheme 2). First, the Suzuki coupling of 3-bromo-1-phenylbenzophosphole oxide with 2-aminophenylboronic acid was presented in Doosan’s patent (Scheme 2A).^12^ More recently, 1,3-diphenylbenzophosphole oxide was obtained from phenyl-*H*-phosphinous acid in a reaction that involves double C-P formation (Scheme 2B).^13^ Subsequent work by the same group has provided analogues having *p*-substituted aryl groups in the 3-position, or modified P-phenyl rings (Scheme 2C).^13b^ However, with this methodology, the substitution pattern in the product is constrained by the substituents in the parent alkene or *H*-phosphinic acid so that the substituent which appears in the *p*-position of the 3-phenyl substituent will also appear in the 6-position of the benzophosphole skeleton. The development of procedures leading to 3-arylbenzo[*b*]phosphole oxides from easily available starting materials that diverge at a late stage of the synthesis is therefore desirable. Physicochemical investigations have revealed that substitution at the 3-position has a significant effect on the photoluminescence of quite heavily substituted benzophosphole oxides,^14^ but the photoluminescence properties of simple 2-*H*-3-arylbenzophosphole oxides have not yet been presented in the literature. We therefore focus on preparing and analyzing this class here.

## Results and Discussion

**Scheme 3:**
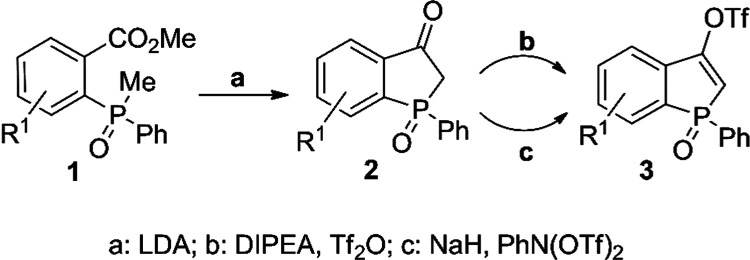
Synthesis of Triflates **3**

The synthesis of 3-arylbenzophosphole oxides presented here involves a Suzuki–Miyaura protocol that couples aryl boronic acids to a recently described class of benzophosphol-3-yl triflates (Scheme 3).^15^ In a previous study, where we showed that these reagents have interesting reactivity toward Grignard reagents, we prepared them through the reaction of benzophospholan-3-one oxides **2** with Tf_2_O in the presence of *N*,*N*-diisopropylethylamine (DIPEA) (Scheme 3b). However, when carried out on a larger scale, this reaction proved difficult to control. We have therefore developed a more scalable synthesis of triflates **3** that employs a milder triflating agent, PhN(OTf)_2_, in the presence of NaH in tetrahydrofuran (THF). Under these conditions, we have successfully obtained triflates **3** from benzophospholan-3-one oxides **2** with good yields on gram scales (Scheme 3c). The structure of **3d** was additionally determined by X-ray crystallography (see the Supporting Information (SI), Figure S1a–c, Tables S1–S2).

Having triflates **3** in hand, we optimized conditions for the reaction of triflates **3** with aryl boronic acids. As a model triflate, we have used compound **3a** and subjected it to the reaction with phenylboronic acid (**4a**) (Table 1). The reaction condition was chosen in accordance with the best literature report for Suzuki–Miyaura couplings of vinyl triflates.^16^

**Table 1:**
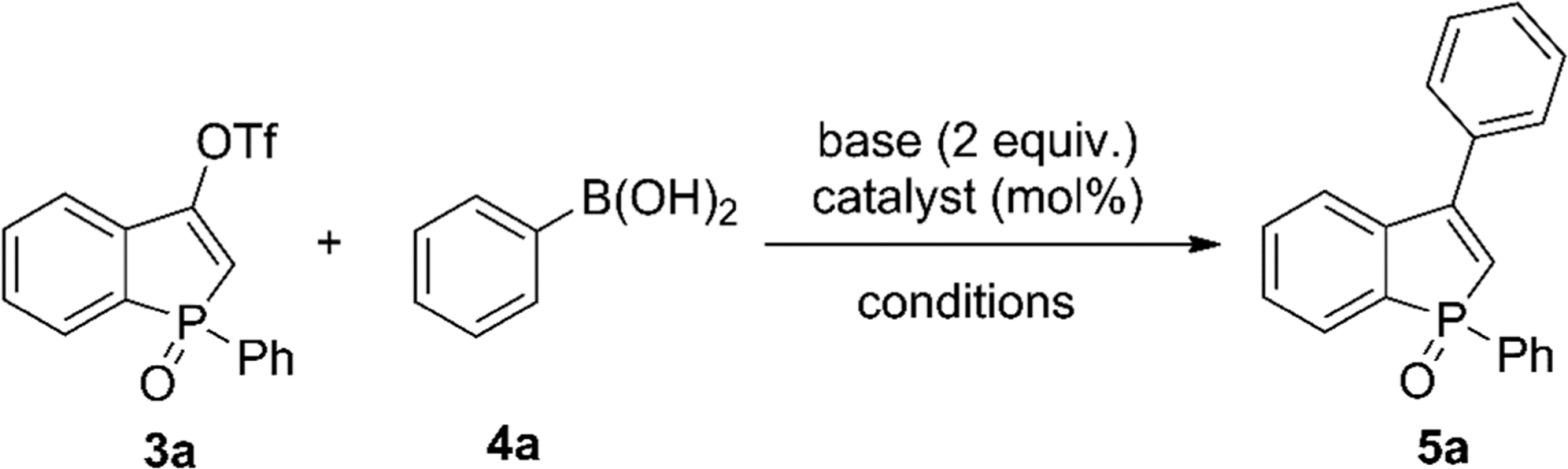
Optimization of the Conditions of Reaction of **3a** with Phenylboronic Acid (**4a**)^a^

no.	base	catalyst (mol %)/ligand (mol %)	conditions	yield of **5a** (%)^b^^,^^c^
1	K_2_CO_3_	Pd(PPh_3_)_4_ (5)	DMF, 110 °C	85 (100)^d^
2	K_2_CO_3_	Pd(PPh_3_)_4_ (5)	THF, 60 °C	57 (100)
3	K_2_CO_3_	Pd(PPh_3_)_4_ (5)	toluene, 80 °C	52 (76)
4	K_2_CO_3_	Pd(PPh_3_)_4_ (5)	toluene, 110 °C	87 (100)
5	K_2_CO_3_	Pd(PPh_3_)_4_ (5)	1,4-dioxane, rt	79 (82)
6	K_2_CO_3_	Pd(PPh_3_)_4_ (5)	1,4-dioxane, 80 °C	74 (100)
7	K_2_CO_3_	Pd(PPh_3_)_4_ (5)	DME, 50 °C	55 (88.5)
8	K_2_CO_3_	Pd(PPh_3_)_4_ (5)	DME, 80 °C	61 (85)
9	K_2_CO_3_	Pd(PPh_3_)_4_ (5)	DME, 110 °C	88 (100)
10	Na_2_CO_3_(aq.)	Pd(PPh_3_)_4_ (5)	DME, 110 °C	62 (100)
11	Na_2_CO_3_ (aq.)	Pd(PPh_3_)_4_ (5)	DME, 110 °C	60 (100)^e^
12	KF	Pd(OAc)_2_ (2)/PCy_3_ (2.4)	DME, 85 °C	21 (46)^f^
13	KF	Pd(OAc)_2_ (2)/PTol_3_ (2.4)	THF, rt	17 (33)^g^
14	Na_2_CO_3_	PdCl_2_ (5)/PTol_3_ (5)	THF, 40 °C	13 (20)^h^

a Reaction conditions: **3a** (0.1 mmol), **4a** (0.12 mmol), base (0.2 mmol), Pd(PPh_3_)_4_ (5.0 μmol), solvent (1 mL), temp. (as indicated), 24 h (entries 1–11); **3a** (0.267 mmol), **4a** (0.32 mmol), base (0.534 mmol), catalyst (as indicated), ligand (as indicated), solvent (1 mL), temp. (as indicated), 24 h (entries 12–14).

b Isolated yields.

c Numbers in parentheses indicate estimated yields (in reference to the starting material) according to ^31^P NMR.

d Yield according to the ^1^H NMR spectrum.

e 3 equiv of LiCl was added to the reaction mixture.

f The formation of ketone **2a** was observed, but it was not isolated.

g Additionally, 24% of ketone **2a** was isolated.

h Additionally, 13% of ketone **2a** was isolated.

In general, the most active catalyst for the reaction of **3a** with boronic acid **4a** was found to be Pd(PPh_3_)_4_ (Table 1, entries 1–11), which is consistent with literature reports of other Suzuki couplings involving vinyl triflates.^16^ The Pd(PPh_3_)_4_ catalyst was compatible with a variety of solvents (THF, 1,2-dimethoxyethane (DME), *N*,*N*-dimethylformamide (DMF), 1,4-dioxane, toluene) and each provided the product in moderate to good yields (Table 1, entries 1–10). From the outset, the degree of consumption of the starting material was found to depend strongly on temperature. In reactions conducted below the solvent boiling point: DME (50 and 80 °C), toluene (80 °C), and 1,4-dioxane (rt), significant amounts of unreacted triflate **3a** were recovered (Table 1, entries 3, 5, 7–8), and the expected product **5a** was isolated in only 52, 79, 55, and 61% yields, respectively. The coupling was found to work best when the reaction was carried out in sealed tubes at 110 °C (for DMF, toluene, and DME); complete conversion of the triflate **3a** was then observed, and the desired product **5a** was obtained in yields ranging from 85 to 88% (Table 1, entries 1, 4, 9). The highest product purity was obtained when reactions were carried out in DME (Table 1, entry 9); this is because some aromatic coproducts (probably arising from the polymerization of the boronic acid) were observed in toluene and DMF, and these complicated the purification of **5a** (Table 1, entries 1, 4). DME was also the best solvent in terms of separating the benzophosphole oxide product **5a** from Ph_3_P(O) (a by-product that arises from catalyst decomposition in yields of up to 15%, depending on the conditions). For the most demanding (*i.e.*, least soluble) compounds, DMF provides a useful option (Table 1, entry 1). Both of the bases used to promote the reaction in DME (solid K_2_CO_3_, aq. Na_2_CO_3_) gave **5a** as the sole product (Table 1, entries 9–10), but a better workup was achieved with K_3_CO_3_ rather than Na_2_CO_3_ (88 vs 62%, Table 1, entries 9, 10). The addition of LiCl (by analogy with the literature^16a^) failed to improve the isolated yield (60%, Table 1, entry 11) and also produced significant quantities of Ph_3_P(O) by-product that complicated the workup. Simultaneously, we verified the efficiency of other catalytic systems. Unfortunately, the assays made with Pd(OAc)_2_^17^ were both significantly less effective than reactions using Pd(PPh_3_)_4_ (Table 1, entries 12–13) and gave similar outcomes in terms of conversion and selectivity (ketone **2a** was formed in the reaction mixture). The poorest results came when PdCl_2_ alone was used as the catalyst (Table 1, entry 13).^18^ In this case, only 20% conversion was observed, and product **5a** was isolated in poor yield (13%).

Given that the results of this preliminary investigation seemed to delineate Pd(PPh_3_)_4_/K_3_CO_3_ in DME at 110 °C over a 24 h system (Table 1, entry 9) as a system without significant deficiencies, we adopted these conditions for our subsequent studies (Table 2).

**Table 2:**
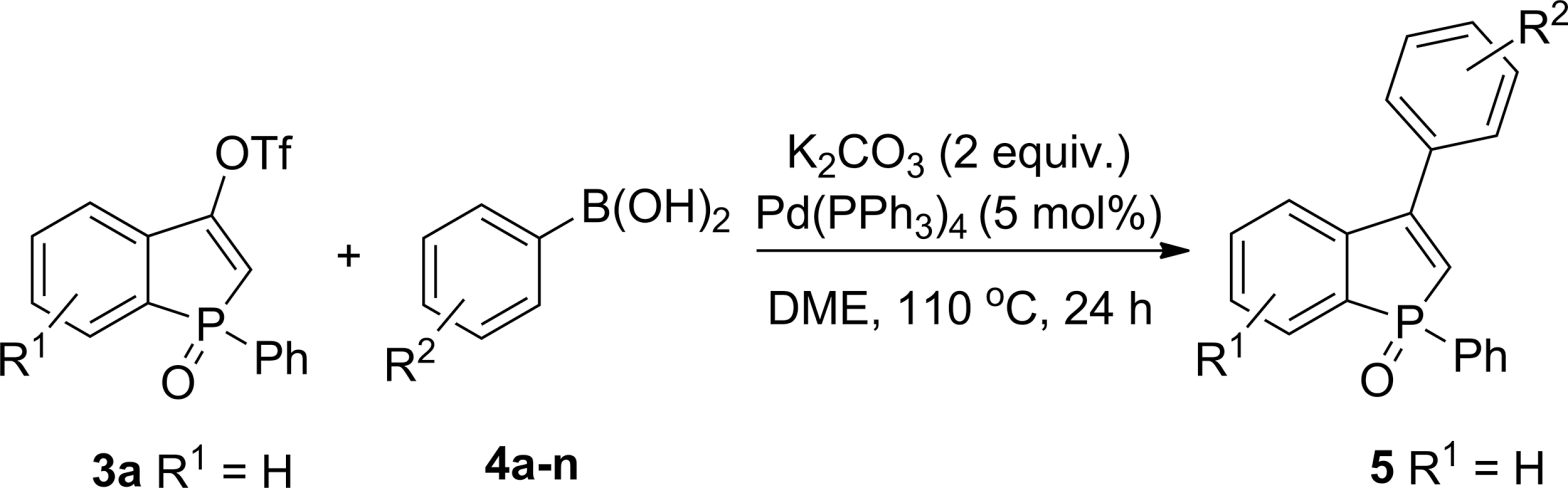

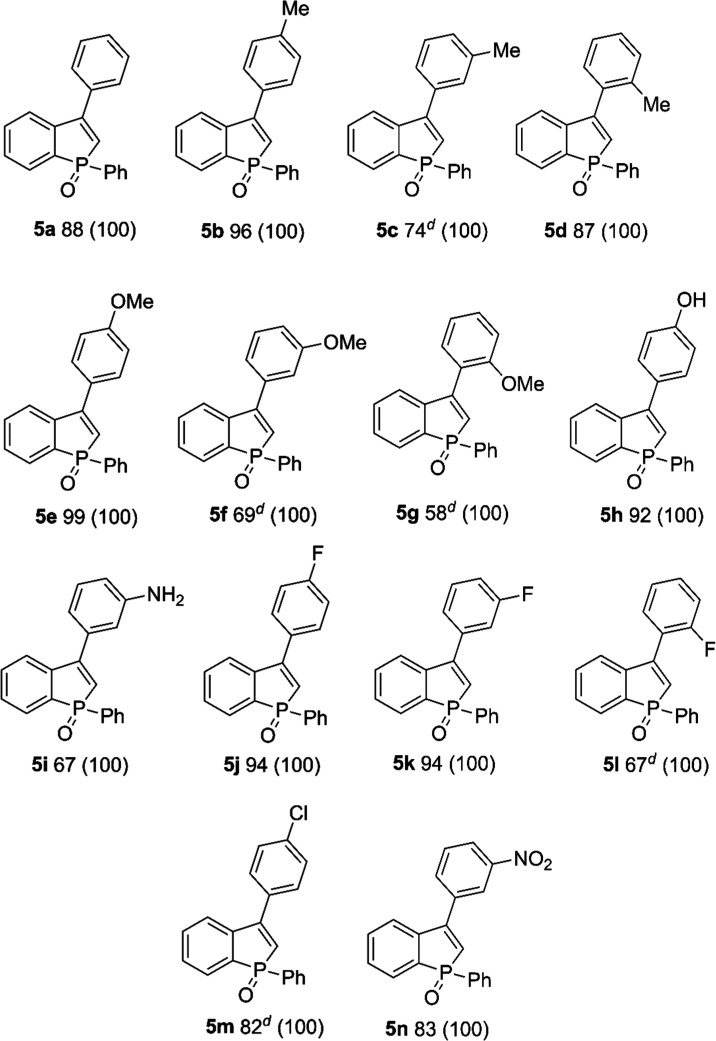
Scope of the Reaction of **3a** with Different Aryl Boronic Acids **4**^a^^,^^b^^,^^c^

a Reaction conditions: **3a** (0.134 mmol), **4** (0.16 mmol), K_2_CO_3_ (0.27 mmol), Pd(PPh_3_)_4_ (6.7 μmol), DME (1 mL), 110 °C, 24 h.

b Isolated yields.

c Numbers in parentheses indicate estimated yields (in reference to the starting material) according to ^31^P NMR.

d For fractions that, after purification, were contaminated with up to 5–7% of Ph_3_P(O), the yields of products were calculated according to ^1^H NMR.

The promising optimization results prompted us to investigate the reactivity of triflate **3a** toward other aryl boronic acids **4b–n**. Under the best conditions (Table 1, entry 9), we were able to achieve complete conversions of **3a** for a variety of boronic acids with electron-donating substituents (methyl, methoxy, hydroxyl, amino groups) as well as electron-withdrawing groups (halogens—chlorine, fluorine, and nitro group). Overall, the desired benzophosphole oxides **5b–n** were obtained selectively with good to excellent yields. In general, the isolated yields of benzophosphole oxides possessing a *p*-substituted ring (**5b**, **5e**, **5h**, and **5j**) were very good, in a range of 92–99%, and their separation from Ph_3_P(O) was easily achieved by column chromatography. In turn, *p*-chloro derivative **5m** and benzophosphole oxides arising from *m*- and *o*-substituted phenylboronic acids (**4c**, **4d**, **4f**, **4g**, **4l**) have formed inseparable mixtures with Ph_3_P(O), which did not allow to calculate isolated yields. The yields of fractions of **5c**,**d**,**f**,**g**,**l**, and **5m** after chromatography columns possessing up to 5–7% of Ph_3_P(O) were calculated according to ^1^H NMR (58–82%). The exception was the *m*-(fluorophenyl)benzophosphole **5k**, obtained from **4k**, which was isolated in 94% yield. The separation of more polar benzophosphole oxides **5h**, **5i**, and **5n** was more successful. For benzophosphole oxides **5h** and **5n**, which possess unprotected *p*-hydroxyl and nitro groups, the isolated yield were high (83–92%). Benzophosphole **5i**, which features a *m*-aminophenyl substituent, proved difficult to work up, with some compounds being lost during chromatography, despite the addition of triethylamine to the eluent.

Subsequently, we investigated the influence of the substituent in the benzo ring of benzophosphol-3-yl triflates **3b–d** upon the reaction with various boronic acids (Table 3). In general, the reaction appears to be quite tolerant to a range of substitution patterns in the benzophosphole rings, and we observed full conversion in all reactions conducted. Except for **6a**, **6b**, and **6j**, the benzophosphole oxides **6** derived from **3b** were isolated in high purity and excellent yields (84–99%). **7a**, obtained from **3c**, was isolated in only moderate yield (68%) because it was difficult to separate from Ph_3_P(O). Since our reactions were routinely carried out on a submillimolar scale, we decided to investigate a scaled-up preparation starting from 1 mmol of **3b**. It was found that **3b** was fully consumed upon reaction with **4n**, and **6n** was isolated in 91% yield. In turn, benzophosphole oxides **8a**, **8b**, and **8n** derived from **3d** revealed difficulties in complete separation from Ph_3_P(O), and their yields were calculated according to ^1^H NMR (81–90%). Their more polar analogues **8h** and **8i** were isolated free of Ph_3_P(O).

**Table 3:**
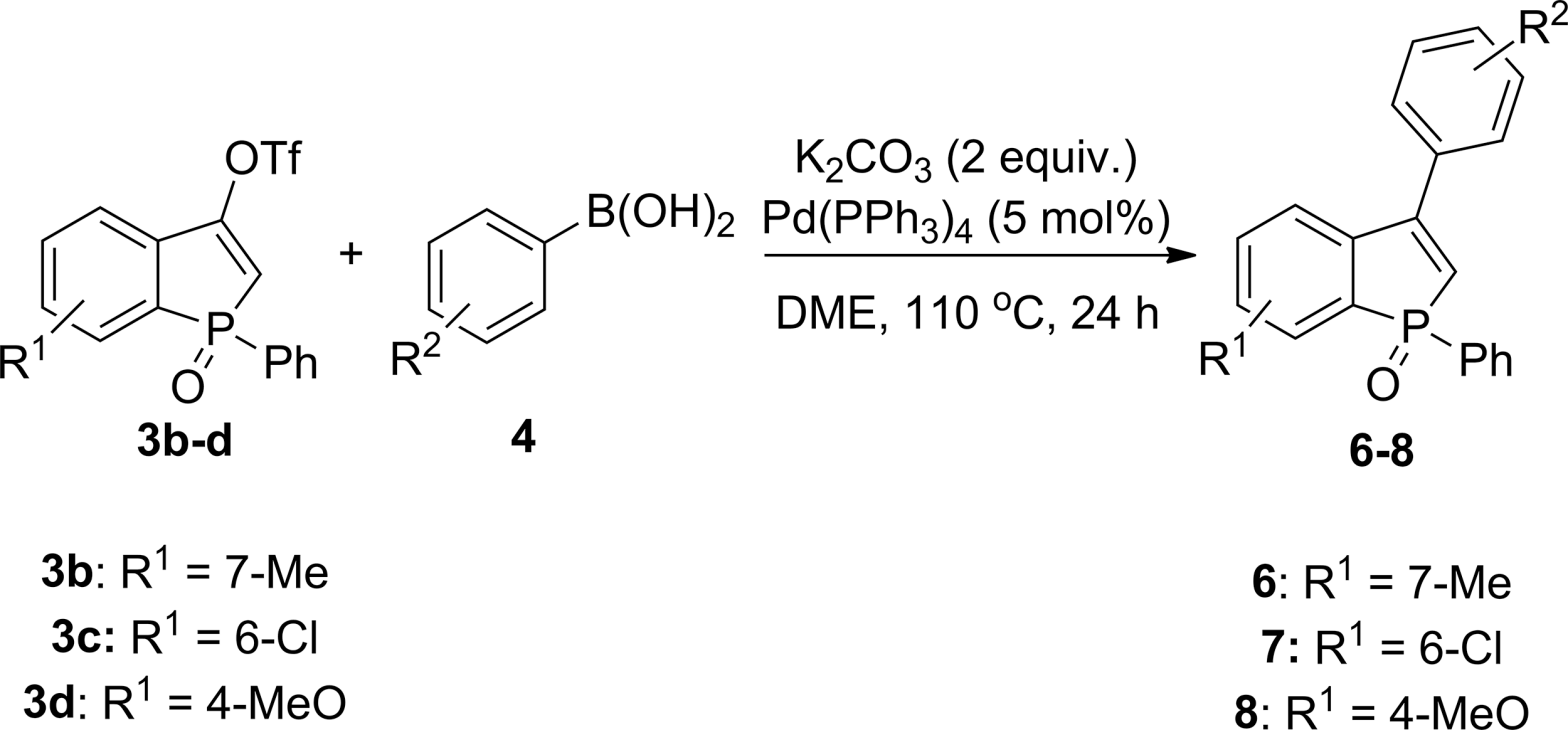

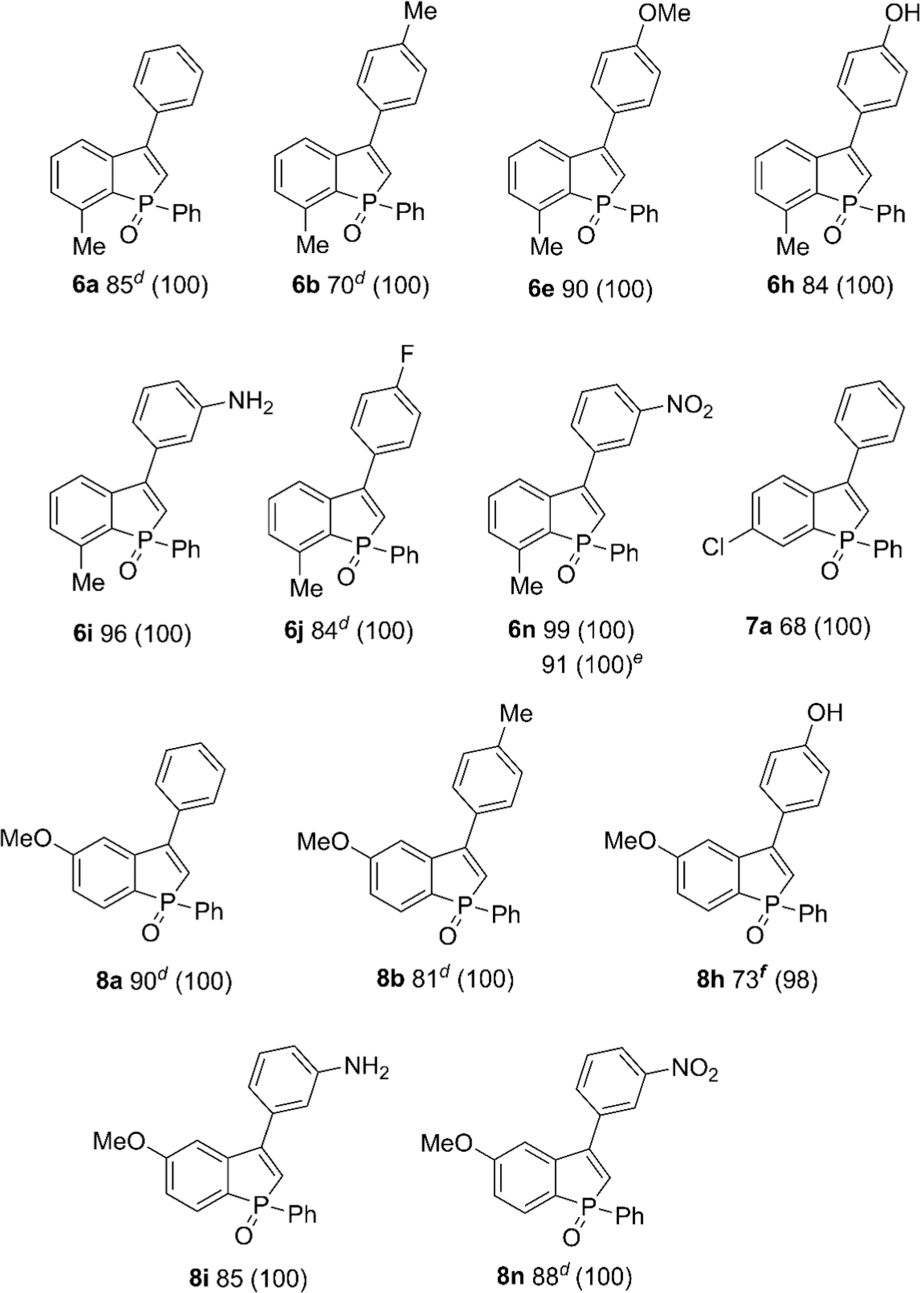
Reaction of Other Benzophosph-3-yl Triflates **3b–d** with Aryl Boronic Acids **4**^a^^,^^b^^,^^c^

a Reaction conditions: **3** (0.134 mmol), **4** (0.16 mmol), K_2_CO_3_ (0.27 mmol), Pd(PPh_3_)_4_ (6.7 μmol), DME (1 mL), 110 °C, 24 h.

b Isolated yields.

c Numbers in parentheses indicate estimated (in reference to the starting material) yields according to ^31^P NMR.

d For fractions that, after purification, were contaminated with up to 5–7% of Ph_3_P(O), the yields of products were calculated according to ^1^H NMR.

e Reaction was carried out in a 1 mmol scale starting from 0.389 g of **3b**, **4n** (1.2 mmol), K_2_CO_3_ (2 mmol), Pd(PPh_3_)_4_ (0.05 mmol), DME (5 mL), 110 °C, 24 h.

f **8h** was contaminated with 2% of **3d** according to ^1^H NMR.

Most of the benzophosphole oxide products were isolated in the form of waxy solids or oils. Only those possessing hydroxy (**5h**, **6h**, **8h**), amino (**5i**, **6i**, **8i**), and nitro groups (**5n**, **6n**, **8n**) were obtained as solids. Good-quality diffraction patterns were obtained only for compounds **5n** and **6n**, and these were fully characterized by X-ray studies. Compounds **5n** (see the SI, Figure S2) and **6n** (see the SI, Figure S3) crystallize in the monoclinic space groups: *I*2/*a* (**5n**) and **P**21/*n* (**6n**) with eight and four molecules in the unit cell, respectively (see the SI, Table S2).

**Figure 1:**
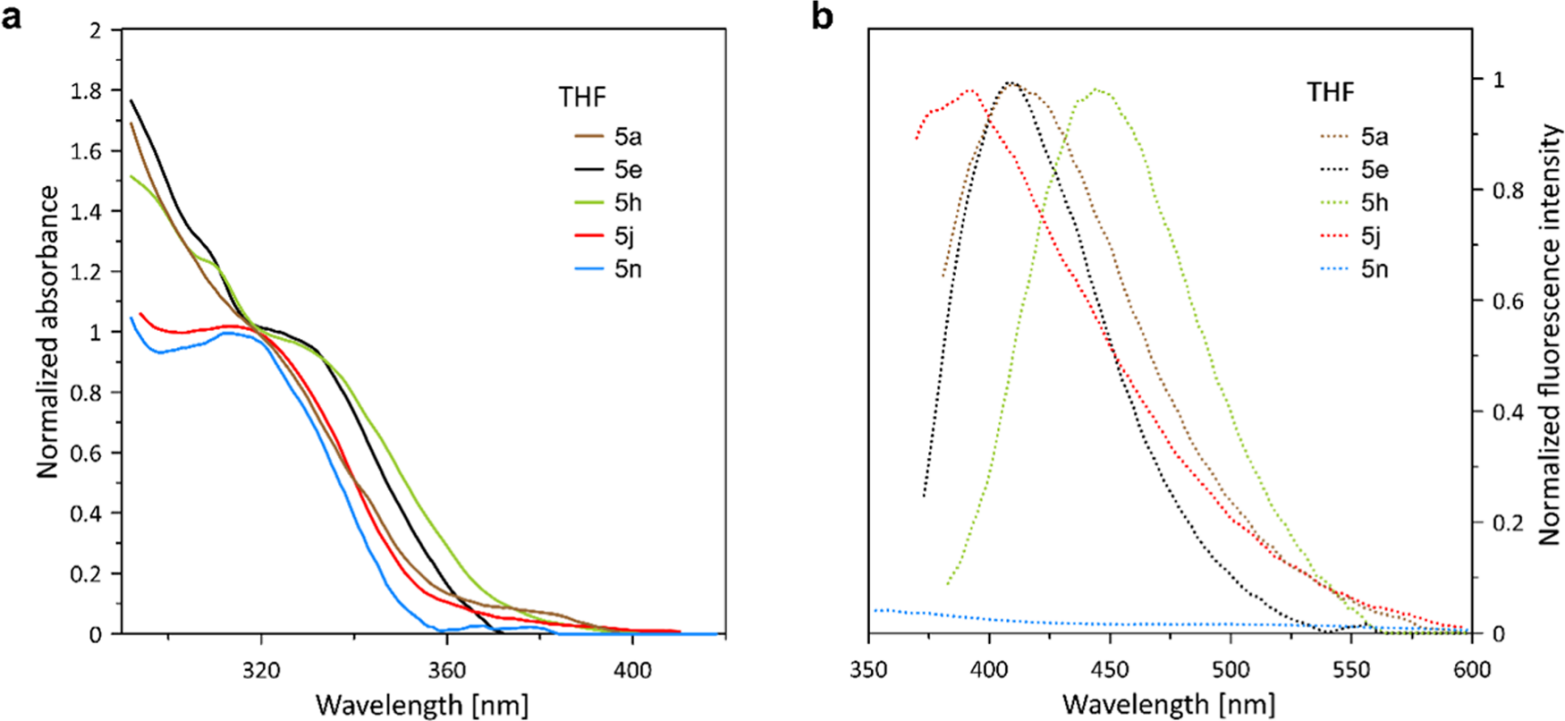
(a) Normalized absorption spectra of **5a,e,h**,**j,n** compounds in THF solutions. (b) Normalized fluorescence emission spectra of **5a,e,h**,**j,n** compounds in THF solutions. The fluorescence emission spectrum of compound **5n** was not normalized due to the lack of emission. For the emission measurements, the excitation wavelength was set at a wavelength corresponding to the absorption maximum of each compound (λ_abs._). All spectra were recorded for concentration 10^–5^ M.

The closest analogues of 3-arylbenzo[*b*]phosphole oxides **5**: 1,2,3-triphenylbenzophosphole oxide (**TPPIO**, λ_em_ = 462 nm, Φ_F_ = 1.2%)^19^ and other 2,3-disubstituted derivatives^14,20^ revealed weak fluorescence in diluted THF solutions, while 1,2-diphenylbenzophosphole oxide (λ_em_ = 415–417 nm, Φ_F_ = 30–83%)^4a,4d,21^ is a good fluorophore. To briefly screen the optical properties of benzo[*b*]phosphole oxides **5** under similar conditions (Figure 1), we have studied several examples showing a variety of substitution patterns in the phenyl ring: unsubstituted **5a**, **5e**, and **5h** bearing electron-donating groups (OMe and OH, respectively) and **5f**, **5n** possessing electron-withdrawing groups (F, NO_2_, respectively) (data collected in Table 4). The emission properties of **5j** and **5a** compounds are characterized by lower fluorescence quantum yields (0.46–0.68%, respectively) and blue-shifted maxima relative to **TPPIO**. As expected, the hypsochromic effect is less marked relative to 1,2-diphenylbenzophosphole oxide.^4a^ In contrast to **5a**, **5n** bearing a nitro substituent was not fluorescent in THF solutions. In turn, the presence of electron-donating groups (OMe and OH) in **5e** and **5h** affected both absorption and emission properties in THF solutions. Two absorption maxima were found in this region at 310 and 330 nm for both compounds.^22^ The emissive properties of **5e** (*R*^2^ = OMe, Φ_F_ = 0.73%) and **5h** (*R*^2^ = OH, Φ_F_ = 1.53%) in THF were improved in comparison to **5a**.^14b,14c^ Both compounds **5e** and **5h** have revealed the absolute fluorescence yields, which were comparable to **TPPIO**(19) and 1,2-diphenyl-3-(*p*-methoxyphenyl)benzophosphole oxide^14a^ (Φ_F_ = 1.0%) but still, emission peaks for compounds **5e** (λ_em_ = 409 nm) and **5h** (λ_em_ = 445 nm) are blue-shifted compared to **TPPIO**(19) (λ_em_ = 462 nm) and 1,3-diphenyl-2-(*p*-hydroxyphenyl)benzophosphole oxide^14a^ (λ_em_ = 485 nm). The optical properties of **5** can be attributed to reduced conjugation that follows from the absence of an aryl substituent at the 2-position. In turn, low Φ_F_ observed for investigated compounds could be due to the intramolecular rotation or vibration of the 3-aryl groups in a solution like in **TPPIO**.^19^

**Table 4:** Optical Data for **5a,e,h,j,n**

comp.	solvent	λ_abs_ [nm]	ε [10^3^ M^–1^*cm^–1^]	λ_em_ [nm]^a^	Φ_F_ (%)^b^	Stokes shift [cm^–1^]
**5a**	toluene	325	2.12	405	0.17	6078
	THF	316	3.87	410	n/d,^c^ 0.68^d^	7315
	DMSO	318	2.34	410	0.70	7056
	DMF	318	2.68	408	0.41	6937
	ACN	320	2.34	414	0.20	7095
**5e**	toluene	332	3.72	407	0.18	5550
	THF	330	2.72	409	0.73^c^	5853
	DMSO	328	3.90	425	0.60	6958
	DMF	326	3.74	419	0.38	6808
	ACN	326	3.37	416	0.19	6636
**5j**	toluene	324	2.22	398	0.18	5739
	THF	316	2.59	390	n/d,^c^ 0.46^d^	6646
	DMSO	318	2.42	410	0.67	7056
	DMF	318	2.28	398	0.44	6321
	ACN	322	2.10	397	0.22	5867
**5h**	toluene	325	1.40	414	1.74	6615
	THF	330	4.19	445	1.53^c^	7831
	DMSO	334	2.90	450	0.55	7718
	DMF	332	3.03	450	0.29	7898
	ACN	329	2.60	433	0.12	7300
**5n**	toluene	325	0.42	484	0.43	10 108
	THF	315	1.60	-	-	-
	DMSO	317	2.27	-	-	-
	DMF	317	3.20	-	-	-
	ACN	317	2.59	-	-	-

a Excitation longest wavelength: λ_abs_.

b Fluorescence quantum yields were determined by comparison with a fluorescence standard quinine sulfate dehydrate.

c Absolute fluorescence yield was calculated using a calibrating sphere.

d For **5a** (λ_abs_ = 410 nm) and **5j** (λ_abs_ = 390 nm), faintly fluorescent at 10^–5^ M, absolute fluorescence yield was calculated using a calibrating sphere at 10^–4^ M [for both *A*_max_ = 0.175, ε = 1.75 × 10^3^ M^–1^*cm^–1^] (see the SI, Figure S8).

**Figure 2:**
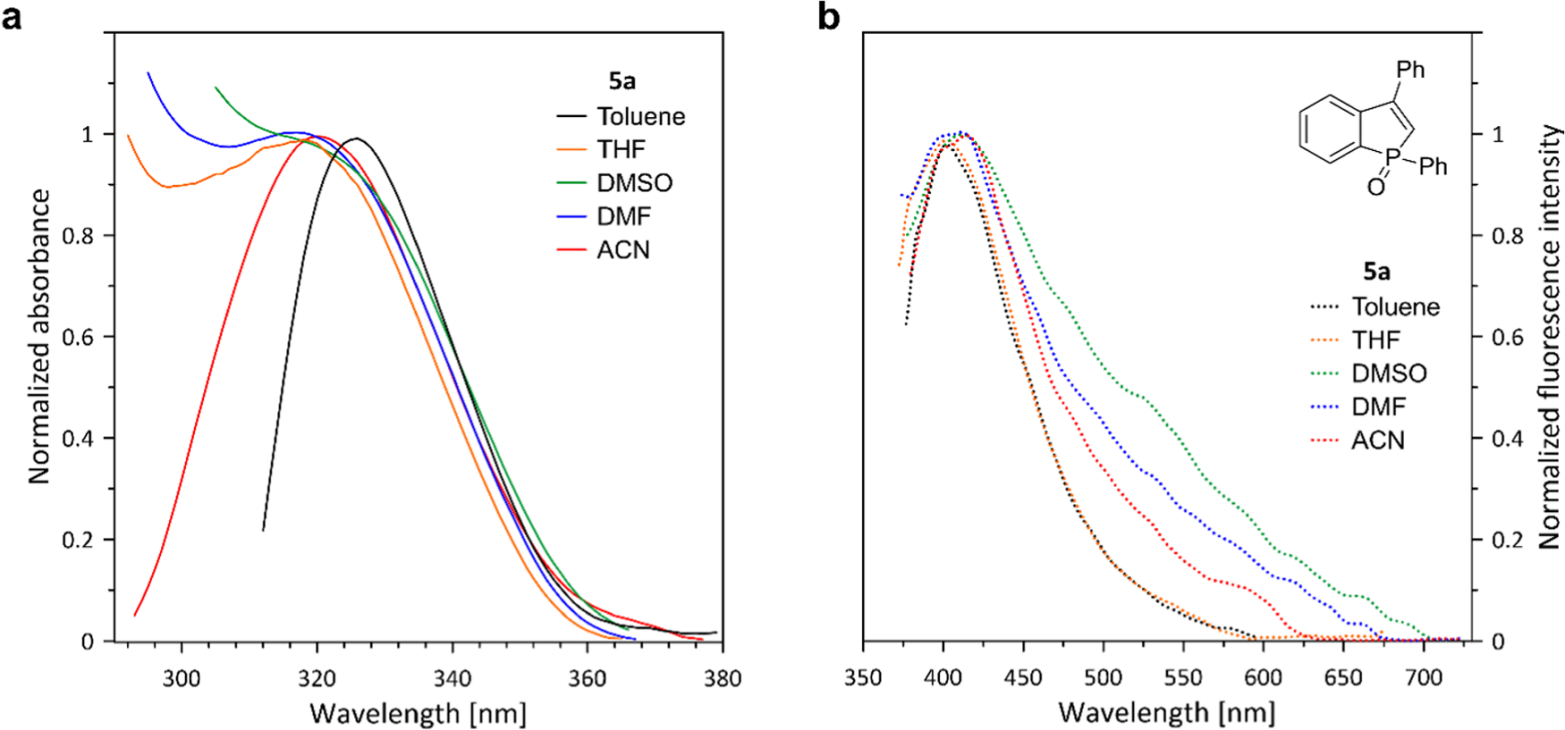
(a) Normalized absorption spectra of **5a** in different solvents at 10^–5^ M. (b) Normalized fluorescence spectra of **5a** in different solvents at 10^–5^ M. Excitation wavelength: λ_abs_.

The optical properties of compounds **5a**, **5e**, **5j**, **5h**, and **5n** in various solvents are summarized in Table 4 and were used to draw the Lippert–Mataga plots (see the SI, Figure S8). A comparison of the absorption and emission spectra collected for compound **5a** is presented in Figure 2 (for spectra collected for compounds **5e**, **5j**, **5h**, and **5n**, see the SI, Figures S4–S7). For **5a** and other investigated compounds, the absorption bands in different polar solvents do not significantly differ. In turn, in much less polar toluene, most compounds revealed a single red-shifted absorption maximum at 325 nm (and for **5e** at 324 nm), probably caused by π-stacking interactions,^23^ which lower the ground-state energy. The emissive properties of unsubstituted benzophosphole oxide **5a** (λ_em_ = 405–414 nm) did not exhibit strong solvent dependence in λ_em_ values, and the corresponding Lippert–Mataga plot was highly linear (*R*^2^ = 0.93). The fluorescence quantum yield of **5a** increased from 0.17% (toluene) to the maximal value in DMSO (0.70%) and then gradually lowered from DMF (0.41%) to ACN (0.2%). Regardless of the substitution pattern, benzophosphole oxides **5e** and **5j** displayed similarities to **5a** in λ_em_ and Φ_F_ values in the investigated solvents. However, for both (**5e** and **5j**), the corresponding Lippert–Mataga plots display much worst linearity (*R*^2^ = 0.72 and *R*^2^ = 0.20). The emission peak maxima observed for benzophosphole oxides **5a**, **5e**, and **5j** come from the locally excited (LE) state. First, values of Φ_F_ are improved with solvent polarity, but then the possibility of nonradiative transitions increases, and fluorescence yield decreases (DMF and ACN). However, in DMSO, due to some other specific interactions, the emission spectrum becomes more structured, and the Φ_F_ value reflects these two effects.^24^

The properties of benzophosphole oxide **5h** stand out from those described for benzophosphole oxides **5a**, **5e**, and **5j.** Notably, in weakly solvating toluene compound, **5h** fluoresces at λ_em_ = 414 nm with the highest Φ_F_ value due to reduced possibility of nonradiative dissipation (ν_abs_ – ν_em_ = 6615 cm^–1^). The change in polarity from toluene to THF shifts a character of interactions from π–π stacking or O–H···π interactions^23^ (in toluene) to hydrogen bonds (from THF to ACN) and causes a bathochromic shift of emission bands (433–450 nm) in the latter. Unlike **5a**, for **5h**, the tendency in the fluorescence yield is gradually decreasing with polarity when the nonradiative transitions become more effective.

In turn, benzophosphole oxide **5n** is characterized by the strong Stokes-shifted ICT fluorescence in toluene (ν_abs_ – ν_em_ = 10 108 cm^–1^). However, its Φ_F_ is higher than those observed for **5a** (Table 4). In more polar solvents (THF, DMSO, DMF, ACN), benzophosphole oxide **5n** is not fluorescent mostly due to the quenching properties of the nitro group^25^ or the preferential nonradiative decay of ICT states.^14b,26^

It is generally accepted that the photoluminescence properties of benzophosphole oxides^27^ are principally associated with a π (HOMO) to π* (LUMO) transition within the benzophosphole core.^28^ Therefore, density functional theory (DFT) calculations at the DFT/B3LYP/6-31+G(d,p) level of theory^29^ supported the above interpretation of the optical data, with a correlation of the HOMO–LUMO bandgap to the various substituent groups present within our library of 3-arylbenzophosphole oxides (**5a–n**, see the SI, Figure S10). The comparison of calculated gap energies to reported cases allowed us to divide the investigated analogous **5a–n** into two main categories. Compounds (**5a–h**, **5j–m**) revealed lower conjugation levels arising from the absence of the substituent at the 2-position, which is manifested in significantly wider HOMO–LUMO gaps (4.18–4.49 eV) than 1,2,3-triphenylbenzophosphole oxide (**TPPIO**, 3.95 eV) possessing phenyl rings at both the 2- and 3-positions of the core benzophosphole.^19^ In this class, in analogy to **TPPIO**,^19^ the LUMO electron population density map is determined quite tightly by the benzophosphole ring, but the properties of the 3-aryl substituent express themselves more strongly upon the HOMO (with an effect that is most marked in benzophosphole oxides **5e, 5g**, and **5h**). The calculations at the TD-DFT/B3LYP/6-31+g-dp level proved that S_0_ → S_1_ transitions in **5a** and **5j** are mainly attributable to π → π* of the benzophosphole ring (see the SI, Table S4). For both compounds (**5a** and **5j**), the absorption band at 331 nm (which refers to 316 nm in solution) in the visible region comes from H → L excitation. In turn, for **5e** and **5h**, which have displayed two absorption maxima, two transitions with the highest probability and contribution level have been found (see the SI, Table S4 and Figure S13). For the higher energetic band at 305 nm (at 310 nm in solution), H-1 → L transition is responsible. The longest wavelength absorption maximum at 349 nm (at 330 nm in solution) arrives from localized transition H → L. In analogy to the absorption, the emission of benzophosphole oxides **5a**, **5e**, and **5j** originates from LE states. The small changes of dipole moments (μ) between S_0_ and S_1_ for compounds **5a**, **5e**, **5h**, and **5j** (see the SI, Table S5) indicate that those compounds do not form highly polarizable excited states affected by the solvent polarity, which are responsible for the Stokes-shifted ICT fluorescence. This stays in line with obtained experimental data and the analysis of the corresponding Lippert–Mataga plots.

The analysis above breaks down at the electronic extremes. The calculations revealed that the compounds possessing either amino (**5i**) or nitro (**5n**) substituents on the phenyl ring differ significantly from the others (**5a–h,j–m**) and **TPPIO**.^19^ The nitro substituent in compound **5n** causes the HOMO–LUMO gap to shrink significantly (3.75 eV), relative to **5a**, with both the LUMO and HOMO levels falling well below compound **5a**. Conversely, the HOMO–LUMO gaps for **5i** possessing an amino functional group equals 3.99 eV. Both these substituents (amino and nitro) also strongly affected the electron distribution within the orbitals, defining their acceptor–donor properties. For **5i**, donating the aminophenyl ring is the dominant component of the HOMO, while the accepting benzophosphole ring provides the major contribution to the LUMO. The reverse appears in **5n**, where the LUMO is dominated by the nitrophenyl group, and the HOMO is mainly localized on the benzophosphole. Therefore, the benzophosphole ring in **5n** behaves as a donor, and the *m*-NO_2_-C_6_H_4_ unit is an acceptor of charge. These orbital characteristics (high separation of charge and orbitals) imply that H → L has an intramolecular charge transfer (ICT) character. According to time-dependent density functional theory (TD-DFT) calculations for **5n**, the difference in μ between the excited state (4.88 D) and its ground-state counterpart (20.94 D) is significant, suggesting that ICT is a major factor in the observed fluorescence properties. However, the absorption band maximum of **5n** is determined by other transitions (H → L+1) because of the geometrically small overlap of the HOMO and the LUMO.

## Conclusions

In summary, we developed a synthetic route to 3-arylbenzophosphole oxides starting from the readily available benzophosphol-3-yl triflates. The access to benzophosphol-3-yl triflates on a larger scale has been presented. The tolerance of the method for diverse substitution patterns in the structure of boronic acid and the benzophosphole core has been proved. The applicability of the method for larger-scale preparations has been confirmed. Although some complications in the separation of the product from Ph_3_P(O) have been observed, this method gives access to 3-arylbenzophosphole oxides, which can be used for functionalization at the 2-position. A preliminary investigation of the optical properties of obtained 3-arylbenzophosphole oxides has been conducted. Despite the fluorescence quantum yields of 3-arybenzophosphole oxides remaining poor, these compounds can be used for the rational design of other fluorophores, especially when it comes to exploiting and improving the electron-donor properties of these model compounds. The theoretical studies regarding the HOMO–LUMO gaps, the influence of the substitution pattern, and the differences in the geometry of the ground and excited states of investigated compounds were undertaken. It was found that the electron-donating substituents enhance emissive properties. Further investigation of the reactivity and optical properties of benzophosphole oxides is on the way in our laboratory.

## Experimental Section

All reactions were performed under an argon atmosphere using Schlenk techniques. Only dry solvents were used, and glassware was heated under vacuum prior to use. All chemicals were used as received unless noted otherwise. Solvents for chromatography and crystallization were distilled once before use, and the solvents for extraction were used as received. THF and toluene were distilled from sodium/benzophenone ketyl under argon. Dichloromethane (DCM) was dried using P_4_O_10_ and distilled before use. 1,4-Dioxane and DME were predistilled and kept over molecular sieves.

^1^H NMR, ^31^P{^1^H} NMR, and ^13^C{^1^H} NMR spectra were recorded on a Bruker Advance 500 spectrometer at ambient temperature in CDCl_3_ unless otherwise noted. Chemical shifts (δ) are reported in ppm from tetramethylsilane with the solvent as an internal indicator (CDCl_3_ 7.27 ppm for ^1^H and 77 ppm for ^13^C). Structural assignments were made with additional information from DEPT experiments. Mass spectra were recorded on Shimadzu GC-MS QP2010S in electron ionization (EI). Melting points were determined on Büchi Melting Point M-560 in a capillary tube and were uncorrected. High-performance liquid chromatography-high-resolution mass spectrometry (HPLC-HRMS) was performed on Shimazu HRMS ESI-IT-TOF using reverse-phase stationary phase with water/MeCN 65:35 as an eluent, electrospray ionization (ESI), and the IT-TOF detector. Elementary analyses were performed on PERKIN ELMER CHN 2400. Thin-layer chromatography (TLC) was performed with precoated silica gel plates and visualized by UV light or KMnO_4_ solution or iodide on silica gel. The reaction mixtures were purified by column chromatography over silica gel (60–240 mesh).

Room temperature UV–vis absorption spectra (in THF) were recorded on a V-660 JASCO spectrophotometer. Photoluminescence measurements (in THF) were carried out with a Photon Technology International Inc. Spectrofluorometer equipped with a continuous wave Xe-arc lamp as a light source. The spectral resolution was maintained at 1 nm. The absolute fluorescence yield (Φ_F_) (in THF) was determined by using a K-Sphere “Petite” integrating sphere (80 mm diameter) connected to a spectrofluorometer.

UV–vis absorption spectra (in toluene, DCM, ACN, DMF, DMSO) were recorded with a Cary 50 Conc spectrophotometer (Varian, Australia). Steady-state fluorescence spectra were recorded with an FS5 spectrofluorometer (Edinburgh Instruments, U.K.). Fluorescence emission spectra (in toluene, DMSO, DMF, ACN) were recorded with excitation set at a wavelength corresponding to the maximum absorption of each sample. The excitation and emission slits were 2/1.5 nm, respectively. Emission spectra were corrected for the wavelength-dependent efficiency of the excitation source and the detector system. All spectroscopic measurements were performed using 1 cm path-length quartz cuvettes (Hellma, Germany) at room temperature (20 °C).

All samples were centrifuged before experiments (14 000 rpm, 10 min) to eliminate any aggregated form of compound suspended in a solvent. Analysis of the concentration of the samples before and after centrifugation (examination of absorbance value before and after centrifugation) confirmed the existence of only a monomeric form of the compound in the solution.

Fluorescence quantum yields were determined by comparison with a fluorescence standard Quinine sulfate dihydrate (0.5 mol L^–1^ H_2_SO_4_).^30^ The fluorescence spectra of dilute solutions (*A* < 0.05) of the compound and the standard were recorded under exactly the same experimental conditions. The quantum yield of the compound (Φ_F_) was calculated from
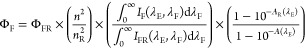
where the subscript *R* refers to the reference solution (standard), *I*_F_ and *I*_FR_ are the corrected fluorescence spectra, and *n* and *n*_R_ are the refractive indexes of solvents. The integrals represent the area under the fluorescence spectra.^31^

Single crystals for **3d** were obtained by dissolving 30 mg of **3d** (an oil solidified upon standing in the fridge) in about 0.7 mL of Et_2_O and 0.3 mL of hexane in an NMR tube. After 1 h of storage in the fridge, yellowish crystals appeared. The obtained crystals were isolated and dried for 24 h at rt.

Single crystals for **5n** were obtained by dissolving 30 mg of **5n** (a foam solid from the chromatography column) in about 0.7 mL of AcOEt and 0.3 mL of hexane in an NMR tube. Then, after 48 h of storage in the fridge, colorless crystals appeared. The obtained crystals were isolated and dried for 24 h at rt.

Single crystals for **6n** were obtained by dissolving 30 mg of **6n** (a solid from the chromatography column) in about 1 mL of AcOEt and 0.1 mL of MeOH in the NMR tube. Then, after a week at rt, colorless crystals appeared. The obtained crystals were isolated and dried for 24 h at rt.

The X-ray intensity data for **3d**, **5n**, and **6n** were measured with an IPDS2T diffractometer equipped with an STOE image plate detector system and microfocus X-ray sources providing Kα radiation by high-grade multilayer X-ray mirror optics for Mo (λ = 0.71073 Å) wavelengths. The measurements were carried out at 120 K. The structures of the compounds were solved by direct methods and refined against *F*^2^ with the Shelxs-2008 and Shelxl-2008 programs^32^ run under WinGX.^33^ Non-hydrogen atoms were refined with anisotropic displacement parameters. The isotropic displacement parameters of all hydrogens were fixed to 1.2 *U*_eq_ for aromatic (1.5 times for methyl) groups.

To calculate the HOMO–LUMO gaps, the quantum-chemical calculations were carried out at the DFT/B3LYP/6-31+G(d,p) level of theory. The ground-state (S_0_) molecular geometries were fully optimized by restricted closed-shell formalism and without any symmetry restriction. A subsequent vibration analysis was also performed to confirm that the structure of each molecule corresponds at least to a local minimum on its potential energy surface. In the next step, the UV–VIS spectra calculations (vertical excitation energy for the S_0_ → S_1_ electronic transitions) were performed for the selected structures with the TD-DFT/B3LYP/6-31+G(d,p) formalism. The GaussSum 3.0 software package^34^ was employed to deconvolute the computed electronic transitions and to determine the contribution of the main electronic transitions. The results are listed in Table S4. Additionally, on the basis of fully optimized ground-state structure, TD-DFT/ B3LYP/6-31+g-dp calculations have been performed to determine the low-lying excited states (S_1_) of **5a**, **5e**, **5h, 5j**, and **5n** compounds. All calculations were performed by the Gaussian 09 package.^35^

The starting compounds were prepared according to reported methods: phenyl(methyl)phosphine oxide,^36^ methyl 2-iodobenzoate,^37^ methyl 2-iodo-6-methylbenzoate,^38^ methyl 2-iodo-5-chlorobenzoate,^39^ 2-iodo-4-methoxybenzoic acid,^40^ methyl 2-iodo-4-methoxybenzoate,^41^ (2-methoxycarbonylphenyl)(methyl)phenylphosphine oxide (**1a**),^15^ [(2-methoxycarbonyl)-6-methylphenyl]methylphenylphosphine oxide (**1b**),^15^ [(2-methoxycarbonyl)-5-chlorophenyl]methylphenylphosphine oxide (**1c**),^15^ [(2-methoxycarbonyl)-4-methoxyphenyl]methylphenylphosphine oxide (**1d**),^15^ 1-phenylbenzophospholan-3-one oxide (**2a**),^15,42^ 1-phenyl-7-methylbenzophospholan-3-one oxide (**2b**),^15^ 1-phenyl-6-chlorobenzophospholan-3-one oxide (**2c**),^15^ and 1-phenyl-5-methoxybenzophospholan-3-one oxide (**2d**).^15^

### A. Procedure for the Synthesis of Triflates **3** (Scheme 3c)

To a Schlenk tube (100 mL) equipped with a magnetic stirrer and an argon inlet, phosphine oxide **2** (0.58 g, 2.4 mmol) in anhydrous THF (25 mL) was added. The reaction mixture was cooled to −78 °C with a dry ice/acetone bath. Then, NaH (0.105 g, 2.63 mmol, 60% in mineral oil) was added, followed by PhN(OTf)_2_ (0.944 g, 2.5 mmol). Then, the reaction mixture was allowed to warm to rt for 2 h. The crude reaction mixture was checked using the ^31^P{^1^H} NMR technique. After completion of the reaction, the mixture was quenched by the addition of water (5 mL), and THF was evaporated. Then, the residue was extracted with DCM (5 × 10 mL). The collected organic phases were dried over Na_2_SO_4_, the solid was filtered off, and the filtrate was evaporated under reduced pressure. The crude product was purified by column chromatography on silica gel using CHCl_3_/acetone (10:1 v/v) as an eluent.

#### 1-Oxido-1-phenyl-1*H*-phosphindol-3-yl Trifluoromethanesulfonate (**3a**)^15^

**2a** (0.58 g, 2.4 mmol) was reacted according to general procedure A to afford **3a** (74%, 0.665 g, 1.78 mmol). ^1^H NMR (500 MHz, CDCl_3_): *δ* 7.68–7.75 (m, 3H), 7.62–7.66 (m, 1H), 7.56–7.61 (m, 1H), 7.52–7.57 (m, 2H), 7.46–7.50 (m, 2H), 6.31 (d, *J*_H-P_ = 14.82 Hz, 1H). ^13^C{^1^H} NMR (125 MHz, CDCl_3_): *δ* 156.7 (d, *J*_C-P_ = 34.5 Hz, C), 135.8 (d, *J*_C-P_ = 19.1 Hz, C), 133.4 (d, *J*_C-P_ = 1.8 Hz, CH), 133.1 (d, *J*_C-P_ = 2.7 Hz, CH), 132.2 (d, *J*_C-P_ = 104.5 Hz, C), 131.5 (d, *J*_C-P_ = 10.9 Hz, CH), 130.8 (d, *J*_C-P_ = 10.9 Hz, CH), 129.5 (d, *J*_C-P_ = 8.2 Hz, CH), 129.1 (d, *J*_C-P_ = 12.7 Hz, CH), 127.5 (d, *J*_C-P_ = 108.1 Hz, C), 121.0 (d, *J*_C-P_ = 10.9 Hz, CH), 118.5 (q, *J*_C-P_ = 321.5 Hz, CF_3_), 108.9 (d, *J*_C-P_ = 95.4 Hz, CH). ^31^P{^1^H} NMR (202 MHz, CDCl_3_): *δ* 32.05 (s).

#### 1-Oxido-1-phenyl-1*H*-7-methylphosphindol-3-yl Trifluoromethanesulfonate (**3b**)^15^

**2b** (0.077 g, 3 mmol) was reacted according to general procedure A to afford **3b** (84%, 0.783 g, 2.01 mmol). ^1^H NMR (500 MHz, CDCl_3_): *δ* 7.73–7.77 (m, 2H), 7.58–7.61 (m, 1H), 7.47–7.53 (m, 3H), 7.35–7.37 (m, 1H), 7.27–7.29 (m, 2H), 6.25 (d, *J*_H-P_ = 15.13 Hz, 1H), 2.37 (s, 3H). ^13^C{^1^H} NMR (125 MHz, CDCl_3_): *δ* 156.8 (d, *J*_C-P_ = 34.5 Hz, C), 142.2 (d, *J*_C-P_ = 8.2 Hz, C), 135.8 (d, *J*_C-P_ = 19.1 Hz, C), 133.5 (d, *J*_C-P_ = 2.7 Hz, CH), 133.1 (d, *J*_C-P_ = 10.0 Hz, CH), 132.9 (d, *J*_C-P_ = 2.7 Hz, CH), 130.8 (d, *J*_C-P_ = 10.9 Hz, CH), 130.3 (d, *J*_C-P_ = 103.5 Hz, C), 129.2 (d, *J*_C-P_ = 13.6 Hz, CH), 127.3 (d, *J*_C-P_ = 106.3 Hz, C), 118.5 (q, *J*_C-P_ = 321.5 Hz, CF_3_), 118.48 (d, *J*_C-P_ = 10.9 Hz, CH), 108.9 (d, *J*_C-P_ = 95.4 Hz, CH), 19.4 (s, CH_3_). ^31^P{^1^H} NMR (202 MHz, CDCl_3_): *δ* 32.43 (s).

#### 1-Oxido-1-phenyl-1*H*-6-chlorophosphindol-3-yl Trifluoromethanesulfonate (**3c**)^15^

**2c** (0.664 g, 2.4 mmol) was reacted according to general procedure A to afford **3c** (60%, 0.589 g, 1.44 mmol). ^1^H NMR (500 MHz, CDCl_3_): *δ* 7.71–7.75 (m, 2H), 7.58–7.65 (m, 3H), 7.45–7.52 (m, 3H), 6.31 (d, *J*_H-P_ = 15.29 Hz, 1H). ^13^C{^1^H} NMR (125 MHz, CDCl_3_): *δ* 156.2 (d, *J*_C-P_ = 33.6 Hz, C), 138.4 (d, *J*_C-P_ = 14.3 Hz, C), 134.6 (d, *J*_C-P_ = 100.8 Hz, C), 133.9 (d, *J*_C-P_ = 19.1 Hz, C), 133.4 (d, *J*_C-P_ = 2.7 Hz, CH), 133.3 (d, *J*_C-P_ = 1.8 Hz, CH), 130.8 (d, *J*_C-P_ = 10.9 Hz, 2CH), 128.9 (d, *J*_C-P_ = 10.0 Hz, 2CH), 128.4 (d, *J*_C-P_ = 13.6 Hz, CH), 126.7 (d, *J*_C-P_ = 108.9 Hz, C), 122.2 (d, *J*_C-P_ = 11.8 Hz, *C*H), 118.5 (q, *J*_C-P_ = 320.6 Hz, CF_3_), 109.9 (d, *J*_C-P_ = 98.6 Hz, CH). ^31^P{^1^H} NMR (202 MHz, CDCl_3_): *δ* 31.17 (s).

#### 1-Oxido-1-phenyl-1*H*-5-methoxyphosphindol-3-yl Trifluoromethanesulfonate (**3d**)^15^

**2d** (0.653 g, 2.4 mmol) was reacted according to general procedure A to afford **3d** (83%, 0.805 g, 1.99 mmol). ^1^H NMR (500 MHz, CDCl_3_): *δ* 7.68–7.72 (m, 2H), 7.57–7.62 (m, 2H), 7.46–7.48 (m, 2H), 7.04–7.05 (m, 1H), 6.97–7.00 (m, 1H), 6.31 (d, *J*_H-P_ = 14.66 Hz, 1H), 3.91 (s, 3H). ^13^C{^1^H} NMR (125 MHz, CDCl_3_): *δ* 164.0 (*J*_P-C_ = 1.8 Hz, C), 155.9 (d, *J*_P-C_ = 33.6 Hz, C), 138.2 (d, *J*_P-C_ = 20.9 Hz, C), 132.9 (d, *J*_P-C_ = 2.7 Hz, CH), 131.1 (d, *J*_P-C_ = 10.0 Hz, CH), 130.8 (d, *J*_P-C_ = 11.8 Hz, CH), 129.1 (d, *J*_P-C_ = 13.6 Hz, CH), 129.7 (d, *J*_P-C_ = 108.9 Hz, C), 122.7 (d, *J*_P-C_ = 110.8 Hz, C), 118.6 (q, *J*_P-C_ = 320.6 Hz, CF_3_), 115.5 (d, *J*_P-C_ = 12.7 Hz, CH), 110.5 (d, *J*_P-C_ = 94.5 Hz, CH), 108.0 (d, *J*_P-C_ = 11.8 Hz, CH), 55.8 (s, CH_3_). ^31^P{^1^H} NMR (202 MHz, CDCl_3_): *δ* 30.90 (s).

### B: General Procedure for the Reaction of **3** with Aryl Boronic Acids (Suzuki Coupling) (Tables 2 and 3)

To a reaction vial (5 mL) equipped with a magnetic stirrer and an argon inlet, **3** (0.134 mmol), aryl boronic acid **4** (0.16 mmol), K_2_CO_3_ (0.037 g, 0.27 mmol), and Pd(PPh_3_)_4_ (7.7 mg, 0.0067 mmol) were added followed by DME (1 mL). The vial was closed using an aluminum cap and heated at 110 °C using heating transfer blocks for 24 h. After that time, the reaction mixture was cooled to rt, and the solvent was evaporated. To the residue, a saturated solution of NaHCO_3_ (5 mL) or NH_4_Cl (5 mL, for **5h**, **6h**, and **8h**) and DCM (10 mL) was added, and the mixture was transferred to an extraction funnel. The mixture was extracted with DCM (3 × 10 mL), and the collected organic phases were dried over Na_2_SO_4_. The solid was filtered off, and the filtrate evaporated under reduced pressure. The crude reaction mixture was checked using the NMR technique. The crude product was purified by column chromatography on silica gel using CHCl_3_/MTBE (30:1 v/v) as an eluent.

#### 1,3-Diphenylbenzophosphole Oxide (**5a**)^13^

**3a** (0.05 g, 0.134 mmol) was reacted with PhB(OH)_2_ (0.0195 g, 0.16 mmol) according to general procedure B to afford **5a** as a yellowish oil (88%, 0.0355 g, 0.117 mmol). *R*_*f*_ = 0.28 (30:1 CHCl_3_/MTBE). ^1^H NMR (500 MHz, CDCl_3_): *δ* 7.77–7.82 (m, 2H), 7.68–7.72 (m, 1H), 7.54–7.57 (m, 2H), 7.51–7.54 (m, 1H), 7.46–7.51 (m, 4H), 7.43–7.48 (m, 3H), 7.40–7.43 (m, 1H), 6.37 (d, *J*_H-P_ = 23.96 Hz, 1H). ^31^P{^1^H} NMR (202 MHz, CDCl_3_): *δ* 37.13 (s); ^13^C{^1^H} NMR (125 MHz, CDCl_3_): *δ* 158.0 (d, *J*_C-P_ = 15.4 Hz, C), 142.1 (d, *J*_C-P_ = 27.3 Hz, C), 134.9 (d, *J*_C-P_ = 16.4 Hz, C), 134.8 (d, *J*_C-P_ = 105.4 Hz, 2C), 132.6 (d, *J*_C-P_ = 1.8 Hz, CH), 132.3 (d, *J*_C-P_ = 2.7 Hz, CH), 130.9 (d, *J*_C-P_ = 10.9 Hz, 2CH), 129.5 (d, *J*_C-P_ = 10.0 Hz, CH), 129.4 (d, *J*_C-P_ = 103.5 Hz, C), 129.3 (d, *J*_C-P_ = 10.0 Hz, CH), 128.1 (d, *J*_C-P_ = 11.8 Hz, CH), 127.8 (s, 2CH), 124.0 (d, *J*_C-P_ = 10.9 Hz, CH), 122.8 (d, *J*_C-P_ = 99.9 Hz, CH). GC-MS (EI) *m*/*z*: 303 (21), 302 (100) (M)^+^, 301 (91). HRMS (ESI/Q-TOF) *m*/*z*: calcd for C_20_H_15_OP [M + Na]^+^, 325.0753, found: 325.0752.

#### 1-Phenyl-3-*p*-tolylbenzophosphole Oxide (**5b**)

**3a** (0.05 g, 0.134 mmol) was reacted with *p*-TolB(OH)_2_ (0.0218 g, 0.16 mmol) according to general procedure B to afford **5b** as a yellowish oil (96%, 0.0406 g, 0.128 mmol). *R*_*f*_ = 0.29 (30:1 CHCl_3_/MTBE). ^1^H NMR (500 MHz, CDCl_3_): *δ* 7.77–7.81 (m, 2H), 7.67–7.71 (m, 1H), 7.52–7.55 (m, 1H), 7.49–7.50 (m, 2H), 7.43–7.47 (m, 4H), 7.37–7.43 (m, 1H), 7.30–7.32 (m, 2H), 6.34 (d, *J*_H-P_ = 24.28 Hz, 1H), 2.44 (s, 3H); ^31^P{^1^H} NMR (202 MHz, CDCl_3_): *δ* 37.12 (s); ^13^C{^1^H} NMR (125 MHz, CDCl_3_): *δ* 158.1 (d, *J*_C-P_ = 15.4 Hz, C), 142.1 (d, *J*_C-P_ = 28.2 Hz, C), 139.8 (s, C), 134.8 (d, *J*_C-P_ = 105.4 Hz, C), 132.5 (d, *J*_C-P_ = 1.8 Hz, CH), 132.2 (d, *J*_C-P_ = 2.7 Hz, CH), 132.0 (d, *J*_C-P_ = 16.4 Hz, C), 130.9 (d, *J*_C-P_ = 10.9 Hz, CH), 129.6 (d, *J*_C-P_ = 102.6 Hz, C), 129.5 (s, 2CH), 129.4 (d, *J*_C-P_ = 10.0 Hz, CH), 129.2 (d, *J*_C-P_ = 10.0 Hz, CH), 128.8 (d, *J*_C-P_ = 12.7 Hz, CH), 127.7 (s, 2CH), 124.0 (d, *J*_C-P_ = 11.8 Hz, CH), 122.2 (d, *J*_C-P_ = 99.9 Hz, CH), 21.4 (s, CH_3_). GC-MS (EI) *m*/*z*: 317 (21), 316 (100) (M)^+^, 315 (84), 301 (12), 300 (31), 299 (13), 297 (12), 269 (23), 268 (22), 254 (18), 253 (26), 252 (24), 239 (24), 223 (19), 196 (14), 191 (13), 189 (14), 178 (13), 165 (16), 77 (10). HRMS (ESI/Q-TOF) *m*/*z*: calcd for C_21_H_17_OP [M + H]^+^, 317.1090 found: 317.1095.

#### 1-Phenyl-3-*m*-tolylbenzophosphole Oxide (**5c**)

**3a** (0.05 g, 0.134 mmol) was reacted with *m*-TolB(OH)_2_ (0.0218 g, 0.16 mmol) according to general procedure B to afford **5c** (74% according to the ^1^H NMR spectrum, 0.0367 g) as a mixture with 7% of Ph_3_P(O). *R*_*f*_ = 0.3 (30:1 CHCl_3_/MTBE). ^1^H NMR (500 MHz, CDCl_3_): *δ* 7.77–7.81 (m, 2H), 7.67–7.71 (m, 1H), 7.52–7.55 (m, 1H), 7.44–7.50 (m, 4H), 7.40–7.44 (m, 1H), 7.34–7.39 (m, 3H), 7.29–7.31 (m, 1H), 6.36 (d, *J*_H-P_ = 24.28 Hz, 1H), 2.44 (s, 3H); ^31^P{^1^H} NMR (202 MHz, CDCl_3_): *δ* 37.17 (s); ^13^C{^1^H} NMR (125 MHz, CDCl_3_): *δ* 158.2 (d, *J*_C-P_ = 15.4 Hz, C), 142.1 (d, *J*_C-P_ = 28.2 Hz, C), 138.6 (s, C), 134.9 (d, *J*_C-P_ = 16.4 Hz, C), 133.8 (d, *J*_C-P_ = 105.4 Hz, C), 132.6 (d, *J*_C-P_ = 1.8 Hz, CH), 132.2 (d, *J*_C-P_ = 3.4 Hz, CH), 130.9 (d, *J*_C-P_ = 10.9 Hz, 2CH), 130.3 (s, CH), 129.46 (d, *J*_C-P_ = 103.5 Hz, C), 129.45 (d, *J*_C-P_ = 10.9 Hz, CH), 129.2 (d, *J*_C-P_ = 9.1 Hz, CH), 128.8 (d, *J*_C-P_ = 12.7 Hz, CH), 128.6 (s, CH), 128.4 (d, *J*_C-P_ = 1.2 Hz, CH), 124.9 (s, CH), 124.1 (d, *J*_C-P_ = 10.8 Hz, CH), 122.5 (d, *J*_C-P_ = 99.9 Hz, CH), 21.5 (s, CH_3_). GC-MS (EI) *m*/*z*: 317 (21), 316 (100) (M)^+^, 315 (76), 301 (18), 300 (46), 299 (19), 297 (13), 283 (10), 269 (25), 268 (23), 254 (21), 253 (27), 252 (27), 239 (21), 223 (18), 196 (14), 191 (14), 189 (16), 178 (14), 165 (19), 77 (11). HRMS (ESI/Q-TOF) *m*/*z*: calcd for C_21_H_17_OPNa [M + Na]^+^, 339.0909, found: 339.0907.

#### 1-Phenyl-3-*o*-tolylbenzophosphole Oxide (**5d**)

**3a** (0.05 g, 0.134 mmol) was reacted with *o*-TolB(OH)_2_ (0.0218 g, 0.16 mmol) according to general procedure B to afford **5d** as a yellowish oil (87%, 0.0367 g, 0.116 mmol). *R*_*f*_ = 0.27 (30:1 CHCl_3_/MTBE). ^1^H NMR (500 MHz, CDCl_3_): *δ* 7.80–7.84 (m, 2H), 7.68–7.72 (m, 1H), 7.55–7.58 (m, 1H), 7.43–7.49 (m, 3H), 7.29–7.41 (m, 5H), 7.00–7.02 (m, 1H), 6.31 (d, *J*_H-P_ = 25.22 Hz, 1H), 2.29 (s, 3H); ^31^P{^1^H} NMR (202 MHz, CDCl_3_): *δ* 38.16 (s); ^13^C{^1^H} NMR (125 MHz, CDCl_3_): *δ* 158.3 (d, *J*_C-P_ = 14.5 Hz, C), 142.9 (d, *J*_C-P_ = 21.8 Hz, C), 134.8 (d, *J*_C-P_ = 18.7 Hz, C), 132.8 (d, *J*_C-P_ = 1.8 Hz, CH), 132.7 (d, *J*_C-P_ = 104.1 Hz, C), 132.3 (d, *J*_C-P_ = 2.7 Hz, CH), 130.9 (d, *J*_C-P_ = 10.9 Hz, CH), 129.5 (d, *J*_C-P_ = 10.0 Hz, CH), 128.9 (d, *J*_C-P_ = 10.0 Hz, CH), 128.89 (s, CH), 128.88 (d, *J*_C-P_ = 12.7 Hz, CH), 124.0 (d, *J*_C-P_ = 10.9 Hz, CH), 123.6 (d, *J*_C-P_ = 98.1 Hz, CH), 20.6 (s, CH_3_). GC-MS (EI) *m*/*z*: 317 (22), 316 (100) (M)^+^, 315 (69), 301 (17), 300 (46), 299 (22), 267 (11), 265 (11), 253 (15), 252 (17), 239 (14), 221 (29), 220 (32), 193 (17), 192 (94), 191 (53), 190 (12), 189 (27), 179 (22), 165 (22), 115 (11), 91 (10), 77 (14). HRMS (ESI/Q-TOF) *m*/*z*: calcd for C_21_H_17_OP [2M + Na]^+^, 655.1926; found: 655.1918.

#### 3-*p*-Anisyl-1-phenylbenzophosphole Oxide (**5e**)

**3a** (0.05 g, 0.134 mmol) was reacted with *p*-AnB(OH)_2_ (0.0243 g, 0.16 mmol) according to general procedure B to afford **5e** as a yellowish oil (99%, 0.0436 g, 0.131 mmol). *R*_*f*_ = 0.26 (30:1 CHCl_3_/MTBE). ^1^H NMR (500 MHz, CDCl_3_): *δ* 7.76–7.80 (m, 2H), 7.65–7.70 (m, 1H), 7.48–7.55 (m, 5H), 7.37–7.47 (m, 3H), 7.00–7.03 (m, 2H), 6.30 (d, *J*_H-P_ = 24.12 Hz, 1H), 3.87 (s, 3H); ^31^P{^1^H} NMR (202 MHz, CDCl_3_): *δ* 36.87 (s); ^13^C{^1^H} NMR (125 MHz, CDCl_3_): *δ* 160.2 (s, C), 157.7 (d, *J*_C-P_ = 15.4 Hz, C), 142.2 (d, *J*_C-P_ = 27.3 Hz, C), 134.0 (d, *J*_C-P_ = 105.4 Hz, C), 132.5 (d, *J*_C-P_ = 1.8 Hz, CH), 132.2 (d, *J*_C-P_ = 2.7 Hz, CH), 132.0 (d, *J*_C-P_ = 10.0 Hz, CH), 130.9 (d, *J*_C-P_ = 10.9 Hz, 2CH), 129.6 (d, *J*_C-P_ = 103.5 Hz, C), 129.4 (d, *J*_C-P_ = 10.9 Hz, CH), 129.3 (s, 2CH), 129.2 (d, *J*_C-P_ = 10.0 Hz, CH), 128.8 (d, *J*_C-P_ = 10.0 Hz, 2CH), 127.2 (d, *J*_C-P_ = 16.3 Hz, C), 124.8 (d, *J*_C-P_ = 11.7 Hz, CH), 121.4 (d, *J*_C-P_ = 100.8 Hz, CH), 114.1 (s, 2CH), 55.4 (s, CH_3_). GC-MS (EI) *m*/*z*: 333 (21), 332 (92) (M)^+^, 331 (24), 316 (100), 315 (22), 301 (15), 285 (21), 270 (15), 241 (12), 240 (17), 239 (41), 183 (14), 152 (20), 138 (10). HRMS (ESI/Q-TOF) *m*/*z*: calcd for C_21_H_17_O_2_PNa [M + Na]^+^: 355.0858, found: 355.0848.

#### 1-Phenyl-3-(*m*-anisyl)benzophosphole Oxide (**5f**)

**3a** (0.05 g, 0.134 mmol) was reacted with *m*-AnB(OH)_2_ (0.0243 g, 0.16 mmol) according to general procedure B to afford **5f** (69% according to the ^1^H NMR spectrum, 0.0315 g) as a mixture with 3% of Ph_3_P(O). *R*_*f*_ = 0.30 (30:1 CHCl_3_/MTBE). ^1^H NMR (500 MHz, CDCl_3_): *δ* 7.77–7.81 (m, 2H), 7.69–7.72 (m, 1H), 7.40–7.54 (m, 7H), 7.02–7.14 (m, 3H), 6.38 (d, *J*_H-P_ = 23.96 Hz, 1H), 3.86 (s, 3H); ^31^P{^1^H} NMR (202 MHz, CDCl_3_): *δ* 37.07 (s); ^13^C{^1^H} NMR (125 MHz, CDCl_3_): *δ* 159.8 (s, C), 157.9 (d, *J*_C-P_ = 15.4 Hz, C), 141.9 (d, *J*_C-P_ = 27.3 Hz, C), 136.3 (d, *J*_C-P_ = 16.4 Hz, C), 133.8 (d, *J*_C-P_ = 105.4 Hz, C), 132.6 (d, *J*_C-P_ = 1.8 Hz, CH), 132.2 (d, *J*_C-P_ = 3.6 Hz, CH), 130.9 (d, *J*_C-P_ = 10.9 Hz, 2CH), 129.9 (s, 2CH), 129.5 (d, *J*_C-P_ = 10.9 Hz, CH), 129.2 (d, *J*_C-P_ = 10.0 Hz, CH), 128.8 (d, *J*_C-P_ = 12.7 Hz, 2CH), 128.5 (d, *J*_C-P_ = 11.8, CH), 124.0 (d, *J*_C-P_ = 10.9 Hz, CH), 122.8 (d, *J*_C-P_ = 99.9 Hz, CH), 120.1 (s, CH), 114.9 (s, CH), 113.4 (s, CH), 55.4 (s, CH_3_). GC-MS (EI) *m*/*z*: 333 (25), 332 (100) (M)^+^, 331 (72), 317 (29), 316 (95), 315 (29), 301 (16), 285 (35), 284 (30), 283 (11), 271 (13), 270 (30), 269 (12), 255 (19), 253 (18), 252 (24), 241 (15), 240 (15), 239 (43), 226 (10), 223 (12), 195 (12), 194 (12), 183 (19), 165 (39), 152 (23), 126 (11), 77 (18). HRMS (ESI/Q-TOF) *m*/*z*: calcd for C_42_H_34_O_4_P_2_ [2M + Na]^+^, 687.1825; found: 687.1814.

#### 1-Phenyl-3-(*o*-anisyl)benzophosphole Oxide (**5g**)

**3a** (0.05 g, 0.134 mmol) was reacted with *o*-AnB(OH)_2_ (0.0243 g, 0.16 mmol) according to general procedure B to afford **5g** (58% according to the ^1^H NMR spectrum, 0.0275 g) as a mixture with 5% of Ph_3_P(O). *R*_*f*_ = 0.3 (30:1 CHCl_3_/MTBE). ^1^H NMR (500 MHz, CDCl_3_): *δ* 7.83–7.88 (m, 2H), 7.63–7.67 (m, 1H), 7.52–7.53 (m, 1H), 7.42–7.47 (m, 4H), 7.33–7.36 (m, 2H), 7.03–7.11 (m, 3H), 6.35 (d, *J*_H-P_ = 24.91 Hz, 1H), 3.80 (s, 3H); ^31^P{^1^H} NMR (202 MHz, CDCl_3_): *δ* 37.53 (s); ^13^C{^1^H} NMR (125 MHz, CDCl_3_): *δ* 156.7 (s, C), 157.9 (d, *J*_C-P_ = 15.4 Hz, C), 133.0 (d, *J*_C-P_ = 106.3 Hz, C), 132.4 (d, *J*_C-P_ = 1.8 Hz, CH), 132.1 (d, *J*_C-P_ = 2.7 Hz, CH), 131.0 (d, *J*_C-P_ = 10.9 Hz, 2CH), 130.5 (s, CH), 129.8 (d, *J*_C-P_ = 1.8 Hz, CH), 129.7 (d, *J*_C-P_ = 102.8 Hz, C), 129.0 (d, *J*_C-P_ = 10.9 Hz, CH), 128.7 (d, *J*_C-P_ = 12.7 Hz, 2CH), 128.5 (d, *J*_C-P_ = 10.0 Hz, CH), 124.7 (d, *J*_C-P_ = 11.8 Hz, CH), 123.9 (d, *J*_C-P_ = 98.1 Hz, CH), 120.8 (s, CH), 111.1 (s, CH), 55.4 (s, CH_3_). GC-MS (EI) *m*/*z*: 333 (23), 332 (100), 331 (67), 317 (44), 316 (59), 315 (35), 301 (14), 299 (17), 270 (17), 255 (11), 254 (12), 253 (26), 252 (28), 241 (11), 240 (11), 239 (40), 236 (11), 223 (28), 194 (15), 183 (17), 179 (14), 178 (23), 165 (50), 152 (18), 126 (13), 107 (10), 77 (20). HRMS (ESI/Q-TOF) *m*/*z*: calcd for C_21_H_17_O_2_P [M + Na]^+^, 355.0858; found: 355.0852.

#### 3-(4-Hydroxyphenyl)-1-phenylbenzophosphole Oxide (**5h**)

**3a** (0.05 g, 0.134 mmol) was reacted with *p*-OH-C_6_H_4_B(OH)_2_ (0.0221 g, 0.16 mmol) according to general procedure B to afford **5h** (92%, 0.0392 g, 0.123 mmol) as pale yellow solid, mp = 256.5–257.5 °C. *R*_*f*_ = 0.34 (30:5:1 CHCl_3_/AcOEt/MeOH). ^1^H NMR (500 MHz, CDCl_3_): *δ* 9.26 (bs, 1H), 7.76–7.80 (m, 2H), 7.66–7.70 (m, 1H), 7.48–7.55 (m, 3H), 7.38–7.43 (m, 2H), 7.37–7.47 (m, 1H), 7.34–7.37 (m, 2H), 7.00–7.03 (m, 2H), 6.23 (d, *J*_H-P_ = 24.43 Hz, 1H); ^31^P{^1^H} NMR (202 MHz, CDCl_3_): *δ* 38.71 (s); ^13^C{^1^H} NMR (125 MHz, CDCl_3_): *δ* 159.9 (s, C). 159.8 (d, *J*_C-P_ = 16.4 Hz, C), 142.2 (d, *J*_C-P_ = 28.2 Hz, C), 133.5 (d, *J*_C-P_ = 106.3 Hz, C), 132.7 (d, *J*_C-P_ = 1.8 Hz, CH), 132.5 (d, *J*_C-P_ = 2.7 Hz, CH), 131.0 (d, *J*_C-P_ = 10.9 Hz, 2CH), 129.5 (d, *J*_C-P_ = 12.7 Hz, CH), 129.3 (s, 2CH), 129.2 (d, *J*_C-P_ = 9.1 Hz, CH), 128.9 (d, *J*_C-P_ = 12.7 Hz, 2CH), 125.8 (d, *J*_C-P_ = 17.3 Hz, C), 124.4 (d, *J*_C-P_ = 12.7 Hz, CH), 119.6 (d, *J*_C-P_ = 101.7 Hz, CH), 116.1 (s, 2CH). HRMS (ESI/Q-TOF) *m*/*z*: calcd for C_20_H_16_O_2_P [M + H]^+^, 319.0888, found: 319.0889.

#### 3-(3-Aminophenyl)-1-phenylbenzophosphole Oxide (**5i**)

**3a** (0.05 g, 0.134 mmol) was reacted with *m*-H_2_N-C_6_H_4_B(OH)_2_-H_2_O (0.0248 g, 0.16 mmol) according to general procedure B to afford **5i** (67%, 0.0284 g, 0.0875 mmol) as a pale yellow solid, mp = 223.6–224.6 °C. *R*_*f*_ = 0.32 (30:5:1 CHCl_3_/AcOEt/MeOH). ^1^H NMR (500 MHz, CDCl_3_): *δ* 7.75–7.80 (m, 2H), 7.65–7.69 (m, 1H), 7.48–7.55 (m, 3H), 7.42–7.47 (m, 2H), 7.37–7.41 (m, 1H), 7.25–7.27 (m, 1H), 6.91–6.92 (m, 1H), 6.83 (bs, 1H), 6.78–6.80 (m, 1H), 6.33 (d, *J*_H-P_ = 24.28 Hz, 1H), 3.86 (m, 2H). ^31^P{^1^H} NMR (202 MHz, CDCl_3_): *δ* 37.04 (s); ^13^C{^1^H} NMR (125 MHz, CDCl_3_): *δ* 158.3 (d, *J*_C-P_ = 15.4 Hz, C), 146.8 (s, C), 142.1 (d, *J*_C-P_ = 27.3 Hz, C), 136.0 (d, *J*_C-P_ = 15.4 Hz, C), 133.9 (d, *J*_C-P_ = 104.5 Hz, 2C), 132.5 (d, *J*_C-P_ = 1.8 Hz, CH), 132.2 (d, *J*_C-P_ = 2.7 Hz, CH), 130.9 (d, *J*_C-P_ = 10.9 Hz, 2CH), 129.7 (s, CH), 129.65 (d, *J*_C-P_ = 102.6 Hz, C), 129.5 (d, *J*_C-P_ = 10.0 Hz, CH), 129.2 (d, *J*_C-P_ = 10.0 Hz, CH), 128.8 (d, *J*_C-P_ = 12.7 Hz, 2CH), 124.0 (d, *J*_C-P_ = 10.9 Hz, CH), 122.3 (d, *J*_C-P_ = 99.9 Hz, CH), 117.9 (s, CH), 116.1 (s, CH), 114.0 (s, CH). HRMS (ESI/Q-TOF) *m*/*z*: calcd for C_20_H_17_ONP [M + H]^+^, 318.1048, found: 318.1049.

#### 3-(*p*-Fluorophenyl)-1-phenylbenzophosphole Oxide (**5j**)

**3a** (0.05 g, 0.134 mmol) was reacted with *p*-F-C_6_H_4_B(OH)_2_ (0.0223 g, 0.16 mmol) according to general procedure B to afford **5j** as an orange oil (94%, 0.0403 g, 0.126 mmol). *R*_*f*_ = 0.26 (30:1 CHCl_3_/MTBE). ^1^H NMR (500 MHz, CDCl_3_): *δ* 7.76–7.80 (m, 2H), 7.67–7.72 (m, 1H), 7.53–7.56 (m, 2H), 7.50–7.51 (m, 1H), 7.40–7.47 (m, 5H), 7.18–7.21 (m, 2H), 6.35 (d, *J*_H-P_ = 23.80 Hz, 1H). ^31^P{^1^H} NMR (202 MHz, CDCl_3_): *δ* 36.81 (s); ^13^C{^1^H} NMR (125 MHz, CDCl_3_): *δ* 163.4 (d, *J*_C-F_ = 249.8 Hz, C-F), 156.9 (d, *J*_C-P_ = 15.4 Hz, C), 141.8 (d, *J*_C-P_ = 27.3 Hz, C), 133.7 (d, *J*_C-P_ = 105.4 Hz, 2C), 132.6 (d, *J*_C-P_ = 1.8 Hz, CH), 132.3 (d, *J*_C-P_ = 3.6 Hz, CH), 132.0 (d, *J*_C-P_ = 10.0 Hz, 2CH), 130.95 (dd, *J*_C-F_ = 3.6 Hz, *J*_C-P_ = 17.3 Hz, C), 130.9 (d, *J*_C-P_ = 10.9 Hz, 2CH), 129.7 (d, *J*_C-P_ = 8.7 Hz, CH), 129.5 (dd, *J*_C-F_ = 32.7 Hz, *J*_C-P_ = 9.1 Hz, CH), 128.8 (d, *J*_C-P_ = 12.7 Hz, CH), 128.5 (d, *J*_C-P_ = 11.8 Hz, CH), 123.9 (d, *J*_C-P_ = 11.9 Hz, CH), 123.1 (d, *J*_C-P_ = 99.0 Hz, CH), 115.9 (d, *J*_C-F_ = 21.8 Hz, CH). GC-MS (EI) *m*/*z*: 321 (22), 320 (100) (M)^+^, 319 (83), 304 (38), 302 (17), 300 (18), 281 (17), 273 (32), 272 (36), 271 (35), 270 (29), 253 (24), 243 (22), 227 (21), 225 (15), 207 (40), 196 (26), 194 (14), 183 (25), 170 (10), 165 (15), 107 (11), 77 (13). HRMS (ESI/Q-TOF) *m*/*z*: calcd for C_20_H_15_OFP [M + H]^+^, 321.0845, found: 321.0846.

#### 1-Phenyl-3-(*m*-fluorophenyl)benzophosphole Oxide (**5k**)

**3a** (0.05 g, 0.134 mmol) was reacted with *m*-F-C_6_H_4_B(OH)_2_ (0.0223 g, 0.16 mmol) according to general procedure B to afford **5k** as a yellowish oil (94%, 0.0402 g, 0.125 mmol). *R*_*f*_ = 0.3 (30:1 CHCl_3_/MTBE). ^1^H NMR (500 MHz, CDCl_3_): *δ* 7.75–7.80 (m, 2H), 7.70–7.72 (m, 1H), 7.51–7.57 (m, 2H), 7.43–7.47 (m, 5H), 7.33–7.35 (m, 1H), 7.24–7.25 (m, 1H), 7.17–7.20 (m, 1H), 6.40 (d, *J*_H-P_ = 23.64 Hz, 1H). ^31^P{^1^H} NMR (202 MHz, CDCl_3_): *δ* 36.86 (s); ^13^C{^1^H} NMR (125 MHz, CDCl_3_): *δ* 162.7 (d, *J*_C-F_ = 247.9 Hz, C-F), 156.6 (d, *J*_C-P_ = 16.4 Hz, *J*_C-F_ = 2.7 Hz, C), 141.6 (d, *J*_C-P_ = 26.4 Hz, C), 136.9 (d, *J*_C-P_ = 7.26 Hz, *J*_C-F_ = 16.35 Hz, C), 133.6 (d, *J*_C-P_ = 105.4 Hz, C), 132.7 (d, *J*_C-P_ = 1.8 Hz, CH), 132.4 (d, *J*_C-P_ = 2.7 Hz, CH), 131.9 (d, *J*_C-P_ = 10.9 Hz, 2CH), 130.6 (d, *J*_C-P_ = 10.9 Hz, CH), 129.7 (d, *J*_C-F_ = 9.1 Hz, CH), 129.4 (d, *J*_C-F_ = 9.1 Hz, CH), 129.1 (d, *J*_C-P_ = 103.5 Hz, C), 123.83 (d, *J*_C-P_ = 11.8 Hz, CH), 123.74 (d, *J*_C-P_ = 99.0 Hz, CH), 123.6 (d, *J*_C-P_ = 2.7 Hz, CH), 116.5 (d, *J*_C-F_ = 21.8 Hz, CH), 114.9 (d, *J*_C-F_ = 22.7 Hz, CH). GC-MS (EI) *m*/*z*: 321 (5), 320 (21) (M)^+^, 319 (14), 304 (13), 303 (6), 283 (10), 282 (12), 291 (38), 253 (22), 209 (15), 208 (21), 207 (100), 191 (14), 135 (10), 133 (15), 96 (12), 73 (30). HRMS (ESI/Q-TOF) *m*/*z*: calcd for C_40_H_28_F_2_O_2_P_2_ [2M + Na]^+^, 663.1425; found: 663.1440.

#### 1-Phenyl-3-(*o*-fluorophenyl)benzophosphole Oxide (**5l**)

**3a** (0.05 g, 0.134 mmol) was reacted with *o*-F-C_6_H_4_B(OH)_2_ (0.0223 g, 0.16 mmol) according to general procedure B to afford **5l** (67% according to the ^1^H NMR spectrum, 0.0308 g) as a mixture with 5% of Ph_3_P(O). *R*_*f*_ = 0.26 (30:1 CHCl_3_/MTBE). ^1^H NMR (500 MHz, CDCl_3_): *δ* 7.79–7.84 (m, 2H), 7.67–7.71 (m, 1H), 7.54–7.57 (m, 1H), 7.44–7.50 (m, 5H), 7.40–7.44 (m, 1H), 7.20–7.30 (m, 3H), 6.45 (d, *J*_H-P_ = 23.96 Hz, 1H). ^31^P{^1^H} NMR (202 MHz, CDCl_3_): *δ* 37.26 (s); ^13^C{^1^H} NMR (125 MHz, CDCl_3_): *δ* 159.4 (d, *J*_C-F_ = 247.8 Hz, C-F), 152.5 (d, *J*_C-P_ = 16.4 Hz, C), 141.9 (d, *J*_C-P_ = 27.3 Hz, C), 132.9 (d, *J*_C-P_ = 105.4 Hz, C), 132.8 (d, *J*_C-P_ = 1.8 Hz, CH), 132.3 (d, *J*_C-P_ = 2.7 Hz, CH), 131.2 (d, *J*_C-P_ = 8.7 Hz, CH), 131.0 (d, *J*_C-P_ = 10.9 Hz, 2CH), 130.2 (d, *J*_C-P_ = 1.8 Hz, CH), 129.6 (d, *J*_C-P_ = 10.9 Hz, CH), 129.2 (d, *J*_C-P_ = 102.6 Hz, CH), 128.9 (d, *J*_C-P_ = 10.3 Hz, CH), 128.89 (d, *J*_C-P_ = 10.3 Hz, CH), 129.7 (d, *J*_C-P_ = 12.7 Hz, 2CH), 125.6 (d, *J*_C-P_ = 98.1 Hz, CH), 124.6 (d, *J*_C-P_ = 3.6 Hz, CH), 124.0 (d, *J*_C-P_ = 1.82 Hz, *J*_C-F_ = 10.9 Hz, CH), 122.8 (dd, *J*_C-P_ = 16.4 Hz, *J*_C-F_ = 16.4 Hz, CH), 116.9 (d, *J*_C-F_ = 21.8 Hz, CH), GC-MS (EI) *m*/*z*: 321 (22), 320 (100) (M)^+^, 319 (66), 304 (25), 303 (10), 301 (10), 273 (28), 272 (21), 271 (19), 270 (18), 254 (18), 253 (46), 252 (38), 243 (20), 227 (20), 207 (24), 194 (12), 186 (18), 165 (12), 151 (10), 77 (12). HRMS (ESI/Q-TOF) *m*/*z*: calcd for C_20_H_14_FOP [M + Na]^+^, 343.0659; found: 343.0654.

#### 1-Phenyl-3-(*p*-chlorophenyl)benzophosphole Oxide (**5m**)

**3a** (0.05 g, 0.134 mmol) was reacted with *p*-Cl-C_6_H_4_B(OH)_2_ (0.025 g, 0.16 mmol) according to general procedure B to afford **5m** as a yellowish oil (82% according to the ^1^H NMR spectrum, 0.0414 g) as a mixture with 7% of Ph_3_P(O). *R*_*f*_ = 0.32 (30:1 CHCl_3_/MTBE). ^1^H NMR (500 MHz, CDCl_3_): *δ* 7.75–7.79 (m, 2H), 7.68–7.70 (m, 1H), 7.41–7.55 (m, 10H), 6.38 (d, *J*_H-P_ = 23.64 Hz, 1H); ^31^P{^1^H} NMR (202 MHz, CDCl_3_): *δ* 36.83 (s); ^13^C{^1^H} NMR (125 MHz, CDCl_3_): *δ* 156.7 (d, *J*_C-P_ = 15.4 Hz, C), 141.7 (d, *J*_C-P_ = 27.3 Hz, C), 135.6 (s, C), 133.7 (d, *J*_C-P_ = 105.4 Hz, C), 133.3 (d, *J*_C-P_ = 17.3 Hz, C), 132.7 (d, *J*_C-P_ = 1.8 Hz, CH), 132.4 (d, *J*_C-P_ = 2.7 Hz, CH), 130.9 (d, *J*_C-P_ = 10.9 Hz, 2CH), 129.7 (d, *J*_C-P_ = 10.9 Hz, CH), 129.4 (d, *J*_C-P_ = 10.0 Hz, CH), 129.3 (d, *J*_C-P_ = 103.5 Hz, C), 129.1 (d, *J*_C-P_ = 7.2 Hz, 2CH), 128.9 (d, *J*_C-P_ = 12.7 Hz, CH), 123.8 (d, *J*_C-P_ = 11.8 Hz, CH), 123.5 (d, *J*_C-P_ = 99.0 Hz, CH). GC-MS (EI) *m*/*z*: 339 (8), 338 (35), 337 (48), 336 (100) (M)^+^, 335 (88), 332 (11), 321 (11), 320 (34), 319 (14), 289 (18), 288 (21), 285 (10), 283 (24), 281 (26), 259 (21), 255 (11), 254 (49), 253 (62), 252 (10), 243 (19), 212 (14), 209 (16), 208 (18), 207 (79), 196 (23), 194 (12), 178 (10), 177 (16), 176 (31), 165 (21), 152 (10), 151 (14), 150 (11), 139 (11), 126 (14), 107 (12), 77 (19). HRMS (ESI/Q-TOF) *m*/*z*: calcd for C_20_H_14_ClOPNa [M + Na]^+^, 359.0363; found: 359.0358.

#### 3-(*m*-Nitrophenyl)-1-phenylbenzophosphole Oxide (**5n**)

**3a** (0.05 g, 0.134 mmol) was reacted with *m*-O_2_N-C_6_H_4_B(OH)_2_ (0.0268 g, 0.16 mmol) according to general procedure B to afford **5n** (83%, 0.0416 g, 0.111 mmol) as yellow solid, mp = 142.5–143.5 °C. *R*_*f*_ = 0.28 (30:1 CHCl_3_/MTBE). ^1^H NMR (500 MHz, CDCl_3_): *δ* 8.41–8.42 (m, 1H), 8.34–8.36 (m, 1H), 7.87–7.90 (m, 1H), 7.77–7.80 (m, 1H), 7.72–7.77 (m, 2H), 7.64–7.69 (m, 1H), 7.53–7.58 (m, 2H), 7.44–7.49 (m, 3H), 7.37–7.39 (m, 1H), 6.50 (d, *J*_H-P_ = 22.9 Hz, 1H). ^31^P{^1^H} NMR (202 MHz, CDCl_3_): *δ* 36.92 (s); ^13^C{^1^H} NMR (125 MHz, CDCl_3_): *δ* 155.2 (d, *J*_C-P_ = 16.4 Hz, C), 148.4 (s, C), 142.1 (d, *J*_C-P_ = 26.3 Hz, C), 136.5 (d, *J*_C-P_ = 16.4 Hz, C), 133.8 (s, CH), 133.4 (d, *J*_C-P_ = 105.4 Hz, C), 133.0 (d, *J*_C-P_ = 1.8 Hz, CH), 132.6 (d, *J*_C-P_ = 2.7 Hz, CH), 130.9 (d, *J*_C-P_ = 10.9 Hz, 2CH), 130.1 (s, CH), 130.0 (d, *J*_C-P_ = 10.9 Hz, CH), 129.7 (d, *J*_C-P_ = 10.0 Hz, CH), 128.9 (d, *J*_C-P_ = 10.0 Hz, 2CH), 128.7 (d, *J*_C-P_ = 103.5 Hz, C), 125.4 (d, *J*_C-P_ = 98.1 Hz, CH), 124.3 (s, CH), 123.5 (d, *J*_C-P_ = 11.8 Hz, CH), 122.7 (s, CH). HRMS (ESI/Q-TOF) *m*/*z*: calcd for C_20_H_15_O_3_NP[M + H]^+^, 348.0789, found: 348.0790.

#### 1,3-Diphenyl-7-methylbenzophosphole Oxide (**6a**)

**3b** (0.052 g, 0.134 mmol) was reacted with PhB(OH)_2_ (0.0195 g, 0.16 mmol) according to general procedure B to afford **6a** (85% according to the ^1^H NMR spectrum, 0.0373 g) as a mixture with 5% of Ph_3_P(O). *R*_*f*_ = 0.31 (30:1 CHCl_3_/MTBE). ^1^H NMR (500 MHz, CDCl_3_): *δ* 7.79–7.83 (m, 2H), 7.52–7.55 (m, 3H), 7.43–7.50 (m, 5H), 7.36–7.40 (m, 1H), 7.26–7.28 (m, 1H), 7.14–7.16 (m, 1H), 6.31 (d, *J*_H-P_ = 24.28 Hz, 1H), 2.39 (s, 3H). ^31^P{^1^H} NMR (202 MHz, CDCl_3_): *δ* 37.44 (s); ^13^C{^1^H} NMR (125 MHz, CDCl_3_): *δ* 157.9 (d, *J*_C-P_ = 15.4 Hz, C), 142.3 (d, *J*_C-P_ = 27.3 Hz, C), 141.5 (d, *J*_C-P_ = 9.1 Hz, C), 135.2 (d, *J*_C-P_ = 16.4 Hz, C), 132.8 (d, *J*_C-P_ = 1.8 Hz, CH), 132.1 (d, *J*_C-P_ = 3.6 Hz, CH), 131.1 (d, *J*_C-P_ = 105.4 Hz, C), 133.4 (d, *J*_C-P_ = 10.0 Hz, CH), 130.9 (d, *J*_C-P_ = 10.9 Hz, 2CH), 129.4 (s, CH), 129.0 (d, *J*_C-P_ = 103.5 Hz, C), 128.8 (d, *J*_C-P_ = 12.7 Hz, 2CH), 128.7 (s, 2CH), 127.8 (s, 2CH), 122.9 (d, *J*_C-P_ = 99.9 Hz, CH), 121.6 (d, *J*_C-P_ = 11.8 Hz, CH), 19.3 (d, *J*_C-P_ = 4.5 Hz, CH_3_). GC-MS (EI) *m*/*z*: 317 (23), 316 (100) (M)^+^, 315 (56), 300 (11), 270 (12), 269 (48), 268 (36), 276 (10), 254 (22), 253 (26), 252 (21), 239 (18), 191 (15), 189 (15), 165 (16), 77 (14). HRMS (ESI/Q-TOF) *m*/*z*: calcd for C_21_H_17_OPNa [M + Na]^+^, 339.0909, found: 339.0907.

#### 7-Methyl-1-phenyl-3-(*p*-tolyl)benzophosphole Oxide (**6b**)

**3b** (0.052 g, 0.134 mmol) was reacted with *p*-TolB(OH)_2_ (0.0218 g, 0.16 mmol) according to general procedure B to afford **6b** (70% according to the ^1^H NMR spectrum, 0.0336 g) as a mixture with 5% of Ph_3_P(O). *R*_*f*_ = 0.28 (30:1 CHCl_3_/MTBE). ^1^H NMR (500 MHz, CDCl_3_): *δ* 7.78–7.82 (m, 2H), 7.65–7.70 (m, 1H), 7.52–7.55 (m, 1H), 7.43–7.51 (m, 5H), 7.36–7.39 (m, 1H), 7.29–7.30 (m, 1H), 7.13–7.15 (m, 1H), 6.28 (d, *J*_H-P_ = 24.29 Hz, 1H), 2.43 (s, 3H), 2.39 (s, 3H); ^31^P{^1^H} NMR (202 MHz, CDCl_3_): *δ* 37.37 (s); ^13^C{^1^H} NMR (125 MHz, CDCl_3_): *δ* 158.1 (d, *J*_C-P_ = 15.4 Hz, C), 142.4 (d, *J*_C-P_ = 27.3 Hz, C), 141.5 (d, *J*_C-P_ = 9.1 Hz, C), 139.6 (s, C), 132.8 (d, *J*_C-P_ = 1.8 Hz, CH), 132.2 (d, *J*_C-P_ = 2.7 Hz, CH), 131.03 (d, *J*_C-P_ = 10.0 Hz, CH), 131.0 (d, *J*_C-P_ = 10.9 Hz, CH), 129.4 (s, 2CH), 128.8 (d, *J*_C-P_ = 10.0 Hz, CH), 128.5 (d, *J*_C-P_ = 11.8 Hz, CH), 127.8 (s, CH), 122.0 (d, *J*_C-P_ = 99.9 Hz, CH), 121.7 (d, *J*_C-P_ = 10.9 Hz, CH), 21.4 (s, CH_3_), 19.3 (d, *J*_C-P_ = 4.5 Hz, CH_3_). GC-MS (EI) *m*/*z*: 331 (23), 330 (100) (M)^+^, 329 (59), 314 (19), 284 (11), 283 (43), 282 (35), 268 (18), 267 (13), 253 (16), 252 (17), 189 (16), 165 (11). HRMS (ESI/Q-TOF) *m*/*z*: calcd for C_22_H_19_OP [M + Na]^+^, 353.1066, found: 353.1066.

#### 7-Methyl-1-phenyl-3-(*p-*anisyl)benzophosphole Oxide (**6e**)

**3b** (0.052 g, 0.134 mmol) was reacted with *p*-AnB(OH)_2_ (0.0243 g, 0.16 mmol) according to general procedure B to afford **6e** as a yellowish oil (90%, 0.0418 g, 0.121 mmol). *R*_*f*_ = 0.26 (30:1 CHCl_3_/MTBE). ^1^H NMR (500 MHz, CDCl_3_): *δ* 7.77–7.82 (m, 2H), 7.48–7.52 (m, 3H), 7.41–7.47 (m, 2H), 7.36–7.40 (m, 1H), 7.31–7.33 (m, 1H), 7.13–7.15 (m, 1H), 7.00–7.01 (m, 2H), 6.25 (d, *J*_H-P_ = 24.28 Hz, 1H), 3.89 (s, 3H), 2.39 (s, 3H). ^31^P{^1^H} NMR (202 MHz, CDCl_3_): *δ* 37.34 (s); ^13^C{^1^H} NMR (125 MHz, CDCl_3_): *δ* 160.6 (s, C), 157.6 (d, *J*_C-P_ = 15.4 Hz, C), 142.2 (d, *J*_C-P_ = 27.3 Hz, C), 141.4 (d, *J*_C-P_ = 9.1 Hz, C), 132.7 (d, *J*_C-P_ = 1.2 Hz, CH), 132.0 (d, *J*_C-P_ = 2.7 Hz, CH), 131.9 (d, *J*_C-P_ = 104.5 Hz, C), 131.0 (d, *J*_C-P_ = 2.7 Hz, CH), 130.9 (d, *J*_C-P_ = 10.9 Hz, CH), 129.3 (s, CH), 129.2 (d, *J*_C-P_ = 101.7 Hz, C), 128.8 (d, *J*_C-P_ = 11.8 Hz, CH), 127.5 (d, *J*_C-P_ = 16.4 Hz, CH), 121.6 (d, *J*_C-P_ = 10.9 Hz, CH), 121.5 (d, *J*_C-P_ = 100.9 Hz, CH), 114.1 (s, CH), 55.4 (s, CH_3_), 19.3 (d, *J*_C-P_ = 4.5 Hz, CH_3_). GC-MS (EI) *m*/*z*: 347 (25), 346 (100) (M)^+^, 345 (47), 331 (12), 330 (25), 299 (33), 298 (46), 284 (17), 283 (14), 281 (19), 287 (16), 253 (17), 252 (11), 207 (47), 178 (12), 165 (13), 134 (11), 73 (13). HRMS (ESI/Q-TOF) *m*/*z*: calcd for C_22_H_20_O_2_P: 347.1201; found: 347.1202.

#### 3-(*p*-Hydroxyphenyl)-7-methyl-1-phenylbenzophosphole Oxide (**6h**)

**3b** (0.052 g, 0.134 mmol) was reacted with *p*-OH-C_6_H_4_B(OH)_2_ (0.0221 g, 0.16 mmol) according to general procedure B to afford **6h** (84%, 0.0374 g, 0.112 mmol) as a brownish solid, mp = 135–136 °C. *R*_*f*_ = 0.21 (30:1 CHCl_3_/MTBE). ^1^H NMR (500 MHz, CDCl_3_): *δ* 9.36 (bs, 1H), 7.76–7.80 (m, 2H), 7.49–7.55 (m, 1H), 7.41–7.47 (m, 2H), 7.29–7.40 (m, 4H), 7.09–7.12 (m, 1H), 7.00–7.02 (m, 2H), 6.14 (d, *J*_H-P_ = 24.59 Hz, 1H), 2.36 (s, 3H); ^31^P{^1^H} NMR (202 MHz, CDCl_3_): *δ* 38.98 (s); ^13^C{^1^H} NMR (125 MHz, CDCl_3_): *δ* 158.9 (s, C), 158.8 (d, *J*_C-P_ = 15.4 Hz, C), 142.5 (d, *J*_C-P_ = 28.16 Hz, C), 141.4 (d, *J*_C-P_ = 9.1 Hz, C), 132.9 (s, CH), 132.3 (d, *J*_C-P_ = 1.8 Hz, CH), 131.1 (d, *J*_C-P_ = 7.2 Hz, CH), 131.0 (d, *J*_C-P_ = 10.9 Hz, CH), 129.3 (s, CH), 128.9 (d, *J*_C-P_ = 11.8 Hz, CH), 125.8 (d, *J*_C-P_ = 16.4 Hz, CH), 122.0 (d, *J*_C-P_ = 10.9 Hz, CH), 119.7 (d, *J*_C-P_ = 101.7 Hz, CH), 116.0 (s, CH), 19.6 (d, *J*_C-P_ = 4.5 Hz, CH_3_). HRMS (ESI/Q-TOF) *m*/*z*: calcd for C_21_H_18_O_2_P [M + H]^+^, 333.1044, found: 333.1044.

#### 3-(*m*-Aminophenyl)-7-methyl-1-phenylbenzophosphole Oxide (**6i**)

**3b** (0.052 g, 0.134 mmol) was reacted with *m*-H_2_N-C_6_H_4_B(OH)_2_-H_2_O (0.0248 g, 0.16 mmol) according to general procedure B to afford **6i** (96%, 0.0424 g, 0.128 mmol) as a brownish solid, mp = 171.3–172.1 °C. *R*_*f*_ = 0.43 (30:5:1 CHCl_3_/AcOEt/MeOH). ^1^H NMR (500 MHz, CDCl_3_): *δ* 7.77–7.81 (m, 2H), 7.51–7.54 (m, 1H), 7.42–7.46 (m, 2H), 7.30–7.38 (m, 2H), 7.23–7.26 (m, 1H), 7.11–7.14 (m, 1H), 6.89–6.92 (m, 1H), 6.76–6.82 (m, 2H), 6.26 (d, *J*_H-P_ = 24.43 Hz, 1H), 3.85 (m, 2H), 2.38 (s, 3H). ^31^P{^1^H} NMR (202 MHz, CDCl_3_): *δ* 37.37 (s); ^13^C{^1^H} NMR (125 MHz, CDCl_3_): *δ* 158.2 (d, *J*_C-P_ = 15.4 Hz, C), 146.8 (s, C), 142.3 (d, *J*_C-P_ = 28.2 Hz, C), 141.3 (d, *J*_C-P_ = 9.1 Hz, C), 136.2 (d, *J*_C-P_ = 16.4 Hz, C), 132.7 (d, *J*_C-P_ = 1.8 Hz, CH), 132.2 (d, *J*_C-P_ = 2.7 Hz, CH), 131.8 (d, *J*_C-P_ = 104.5 Hz, C), 130.96 (d, *J*_C-P_ = 9.1 Hz, CH), 130.9 (d, *J*_C-P_ = 10.9 Hz, CH), 129.6 (s, CH), 129.2 (d, *J*_C-P_ = 101.7 Hz, C), 128.8 (d, *J*_C-P_ = 12.7 Hz, CH), 122.4 (d, *J*_C-P_ = 99.9 Hz, CH), 121.8 (d, *J*_C-P_ = 11.8 Hz, CH), 117.9 (s, CH), 115.9 (s, CH), 114.1 (s, CH), 19.3 (d, *J*_C-P_ = 4.5 Hz, CH_3_). GC-MS (EI) *m*/*z*: 332 (18), 331 (75) (M)^+^, 330 (32), 316 (8), 315 (27), 285 (14), 284 (55), 283 (22), 282 (18), 281 (41), 269 (15), 268 (15), 267 (21), 253 (16), 252 (12), 209 (15), 208 (22), 207 (100), 193 (12), 191 (17), 165 (13), 135 (13), 134 (18), 96 (12), 77 (11). HRMS (ESI/Q-TOF) *m*/*z*: calcd for C_21_H_19_ONP [M + H]^+^, 332.1204, found: 332.1206.

#### 3-(*p*-Fluorophenyl)-7-methyl-1-phenylbenzophosphole Oxide (**6j**)

**3b** (0.052 g, 0.134 mmol) was reacted with *p*-F-C_6_H_4_B(OH)_2_ (0.0223 g, 0.16 mmol) according to general procedure B to afford **6j** (84% according to the ^1^H NMR spectrum, 0.0394 g) as a mixture with 5% of Ph_3_P(O). *R*_*f*_ = 0.21 (30:1 CHCl_3_/MTBE). ^1^H NMR (500 MHz, CDCl_3_): *δ* 7.77–7.81 (m, 2H), 7.50–7.56 (m, 3H), 7.43–7.47 (m, 2H), 7.37–7.40 (m, 1H), 7.22–7.24 (m, 1H), 7.15–7.20 (m, 3H), 6.29 (d, *J*_H-P_ = 23.7 Hz, 1H), 2.39 (s, 3H); ^31^P{^1^H} NMR (202 MHz, CDCl_3_): *δ* 37.23 (s); ^13^C{^1^H} NMR (125 MHz, CDCl_3_): *δ* 163.2 (d, *J*_C-F_ = 248.9 Hz, C-F), 156.8 (d, *J*_C-P_ = 15.4 Hz, C), 142.1 (d, *J*_C-P_ = 27.3 Hz, C), 141.6 (d, *J*_C-P_ = 9.1 Hz, C), 132.8 (d, *J*_C-P_ = 1.8 Hz, CH), 132.2 (d, *J*_C-P_ = 3.6 Hz, CH), 131.6 (d, *J*_C-P_ = 103.6 Hz, C), 131.2 (d, *J*_C-P_ = 10.0 Hz, CH), 130.9 (d, *J*_C-P_ = 10.9 Hz, 2CH), 129.7 (d, *J*_C-F_ = 7.2 Hz, 2CH), 129.1 (d, *J*_C-P_ = 101.7 Hz, C), 128.8 (d, *J*_C-P_ = 11.8 Hz, 2CH), 123.2 (d, *J*_C-P_ = 99.0 Hz, CH), 121.4 (d, *J*_C-P_ = 10.9 Hz, CH), 115.8 (d, *J*_C-F_ = 21.8 Hz, 2CH), 19.3 (d, *J*_C-P_ = 4.5 Hz, CH_3_). GC-MS (EI) *m*/*z*: 335 (20), 334 (90) (M)^+^, 333 (47), 318 (17), 288 (11), 287 (43), 286 (40), 285 (11), 283 (14), 282 (12), 281 (37), 272 (19), 271 (23), 270 (19), 257 (17), 253 (13), 214 (14), 210 (10), 209 (23), 208 (24), 207 (100), 191 (19), 189 (11), 183 (13), 135 (11), 133 (19), 96 (14), 77 (12), 73 (29). HRMS (ESI/Q-TOF) *m*/*z*: calcd for C_21_H_17_OFP [M + H]^+^, 335.1001, found: 335.1002.

#### 3-(*m*-Nitrophenyl)-7-methyl-1-phenylbenzophosphole Oxide (**6n**)

**3b** (0.052 g, 0.134 mmol) was reacted with *m*-O_2_N-C_6_H_4_B(OH)_2_ (0.0268 g, 0.16 mmol) according to general procedure B to afford **6n** (99%, 0.0479 g, 0.133 mmol) as a yellowish solid, mp = 207–208 °C. *R*_*f*_ = 0.23 (30:1 CHCl_3_/MTBE). ^1^H NMR (500 MHz, CDCl_3_): *δ* 8.39–8.41 (m, 1H), 8.33–8.36 (m, 1H), 7.86–7.88 (m, 1H), 7.78–7.82 (m, 2H), 7.69–7.72 (m, 1H), 7.56–7.60 (m, 1H), 7.46–7.50 (m, 2H), 7.41–7.46 (m, 1H), 7.16–7.23 (m, 2H), 6.44 (d, *J*_H-P_ = 23.01 Hz, 1H), 2.41 (s, 3H). ^31^P{^1^H} NMR (202 MHz, CDCl_3_): *δ* 37.15 (s); ^13^C{^1^H} NMR (125 MHz, CDCl_3_): *δ* 155.1 (d, *J*_C-P_ = 16.4 Hz, C), 148.4 (s, C), 142.1 (d, *J*_C-P_ = 9.1 Hz, C), 141.3 (d, *J*_C-P_ = 27.2 Hz, C), 136.8 (d, *J*_C-P_ = 17.2 Hz, C), 133.8 (s, CH), 133.1 (d, *J*_C-P_ = 1.8 Hz, CH), 132.5 (d, *J*_C-P_ = 3.6 Hz, CH), 131.6 (d, *J*_C-P_ = 10.0 Hz, CH), 130.9 (d, *J*_C-P_ = 10.9 Hz, CH), 130.1 (s, CH), 129.0 (d, *J*_C-P_ = 12.7 Hz, CH), 125.5 (d, *J*_C-P_ = 98.1 Hz, CH), 124.2 (s, CH), 122.8 (s, CH), 121.7 (d, *J*_C-P_ = 10.9 Hz, CH), 19.4 (d, *J*_C-P_ = 4.5 Hz, CH_3_). HRMS (ESI/Q-TOF) *m*/*z*: calcd for C_21_H_17_NO_3_ [M + H]^+^, 331.1208, found: 331.1208.

#### 6-Chloro-1,3-diphenylbenzophosphole Oxide (**7a**)

**3c** (0.0547 g, 0.134 mmol) was reacted with PhB(OH)_2_ (0.0195 g, 0.16 mmol) according to general procedure B to afford **7a** as a yellowish oil (68%, 0.0307 g, 0.0911 mmol). *R*_*f*_ = 0.31 (30:1 CHCl_3_/MTBE). ^1^H NMR (500 MHz, CDCl_3_): *δ* 7.77–7.82 (m, 2H), 7.63–7.65 (m, 1H), 7.55–7.60 (m, 1H), 7.49–7.55 (m, 8H), 7.40–7.42 (m, 1H), 6.39 (d, *J*_H-P_ = 24.28 Hz, 1H). ^31^P{^1^H} NMR (202 MHz, CDCl_3_): *δ* 36.04 (s); ^13^C{^1^H} NMR (125 MHz, CDCl_3_): *δ* 157.6 (d, *J*_C-P_ = 14.5 Hz, C), 140.2 (d, *J*_C-P_ = 26.4 Hz, C), 136.3 (d, *J*_C-P_ = 100.8 Hz, C), 136.1 (d, *J*_C-P_ = 14.5 Hz, CH), 134.5 (d, *J*_C-P_ = 16.5 Hz, CH), 133.9 (d, *J*_C-P_ = 10.9 Hz, CH), 132.6 (d, *J*_C-P_ = 2.7 Hz, CH), 132.4 (d, *J*_C-P_ = 1.8 Hz, CH), 130.9 (d, *J*_C-P_ = 10.9 Hz, CH), 129.8 (s, CH), 129.5 (d, *J*_C-P_ = 10.9 Hz, CH), 129.0 (d, *J*_C-P_ = 12.7 Hz, CH), 128.9 (s, 2CH), 127.7 (s, 2CH), 125.1 (d, *J*_C-P_ = 11.8 Hz, CH), 122.8 (d, *J*_C-P_ = 99.9 Hz, CH). HRMS (ESI/Q-TOF) *m*/*z*: calcd for C_20_H_15_ClOP [M + H]^+^, 337.0549; found: 337.0548.

#### 1,3-Diphenyl-5-methoxybenzophosphole Oxide (**8a**)

**3d** (0.0542 g, 0.134 mmol) was reacted with PhB(OH)_2_ (0.0195 g, 0.16 mmol) according to general procedure B to afford **8a** (90% according to the ^1^H NMR spectrum, 0.0436 g) as a mixture with 7% of Ph_3_P(O). *R*_*f*_ = 0.22 (30:1 CHCl_3_/MTBE). ^1^H NMR (500 MHz, CDCl_3_): *δ* 7.76–7.80 (m, 2H), 7.62–7.64 (m, 1H), 7.44–7.55 (m, 8H), 7.00–7.01 (m, 1H), 6.88–6.89 (m, 1H), 6.39 (d, *J*_H-P_ = 23.64 Hz, 1H), 3.82 (s, 3H). ^31^P{^1^H} NMR (202 MHz, CDCl_3_): *δ* 36.07 (s); ^13^C{^1^H} NMR (125 MHz, CDCl_3_): *δ* 163.5 (s, C), 157.2 (d, *J*_C-P_ = 14.5 Hz, C), 144.5 (d, *J*_C-P_ = 29.1 Hz, C), 134.8 (d, *J*_C-P_ = 16.4 Hz, C), 132.1 (d, *J*_C-P_ = 2.7 Hz, CH), 130.9 (d, *J*_C-P_ = 10.9 Hz, 2CH), 130.8 (d, *J*_C-P_ = 10.9 Hz, CH), 129.1 (d, *J*_C-P_ = 104.5 Hz, C), 129.5 (s, CH), 128.8 (s, CH), 127.7 (d, *J*_C-P_ = 12.7 Hz, 2CH), 127.8 (s, 2CH), 124.4 (d, *J*_C-P_ = 111.7 Hz, C), 125.3 (d, *J*_C-P_ = 99.0 Hz, CH), 112.9 (d, *J*_C-P_ = 11.8 Hz, CH), 111.9 (d, *J*_C-P_ = 12.7 Hz, CH), 55.6 (s, CH_3_). GC-MS (EI) *m*/*z*: 333 (4), 332 (100) (M)^+^, 331 (44), 317 (16), 316 (67), 315 (14), 301 (14), 285 (31), 271 (11), 270 (31), 255 (35), 252 (17), 241 (14), 240 (13), 239 (50), 220 (11), 183 (19), 165 (32), 152 (13), 139 (10), 77 (15). HRMS (ESI/Q-TOF) *m*/*z*: calcd for C_21_H_18_O_2_P [M + H]^+^, 333.1044, found: 333.1044.

#### 1-Phenyl-5-methoxy-3-(*p*-tolyl)benzophosphole Oxide (**8b**)

**3d** (0.0542 g, 0.134 mmol) was reacted with *p*-TolB(OH)_2_ (0.0218 g, 0.16 mmol) according to general procedure B to afford **8b** (81% according to the ^1^H NMR spectrum, 0.0418 g) as a mixture with 7% of Ph_3_P(O). *R*_*f*_ = 0.53 (30:5:1 CHCl_3_/AcOEt/MeOH). ^1^H NMR (500 MHz, CDCl_3_): *δ* 7.75–7.79 (m, 2H), 7.59–7.63 (m, 1H), 7.51–7.54 (m, 1H), 7.42–7.45 (m, 4H), 7.29–7.31 (m, 2H), 7.02–7.03 (m, 1H), 6.88–6.89 (m, 1H), 6.36 (d, *J*_H-P_ = 23.80 Hz, 1H), 3.82 (s, 3H), 2.44 (s, 3H). ^31^P{^1^H} NMR (202 MHz, CDCl_3_): *δ* 36.07 (s); ^13^C{^1^H} NMR (125 MHz, CDCl_3_): *δ* 163.5 (d, *J*_C-P_ = 1.8 Hz, C), 157.3 (d, *J*_C-P_ = 14.5 Hz, C), 144.6 (d, *J*_C-P_ = 29.1 Hz, C), 139.7 (s, CH), 132.1 (d, *J*_C-P_ = 2.7 Hz, CH), 132.0 (d, *J*_C-P_ = 4.5 Hz, CH), 130.9 (d, *J*_C-P_ = 10.9 Hz, 2CH), 130.8 (d, *J*_C-P_ = 11.8 Hz, CH), 129.9 (d, *J*_C-P_ = 104.5 Hz, C), 129.5 (s, 2CH), 128.7 (d, *J*_C-P_ = 12.7 Hz, 2CH), 127.7 (s, 2CH), 124.5 (d, *J*_C-P_ = 111.7 Hz, C), 123.7 (d, *J*_C-P_ = 99.0 Hz, CH), 112.9 (d, *J*_C-P_ = 11.8 Hz, CH), 111.9 (d, *J*_C-P_ = 11.8 Hz, CH), 55.5 (s, CH_3_), 21.3 (s, CH_3_). GC-MS (EI) *m*/*z*: 347 (5), 346 (19) (M)^+^, 345 (12), 330 (19), 283 (11), 282 (13), 281 (46), 267 (12), 253 (19), 209 (15), 208 (23), 207 (100), 193 (10), 191 (13), 147 (12), 135 (12), 133 (14), 95 (13), 73 (36). HRMS (ESI/Q-TOF) *m*/*z*: calcd for C_22_H_20_O_2_P [M + H]^+^, 347.1201, found: 347.1202.

#### 3-(*p*-Hydroxyphenyl)-1-phenyl-5-methoxybenzophosphole Oxide (**8h**)

**3d** (0.0542 g, 0.134 mmol) was reacted with *p*-OH-C_6_H_4_B(OH)_2_ (0.0221 g, 0.16 mmol) according to general procedure B to afford **8h** (73%, according to the ^1^H NMR spectrum, 0.035 g) as a pale yellow solid, mp = 248.4–249.6 °C. *R*_*f*_ = 0.26 (30:5:1 CHCl_3_/AcOEt/MeOH). ^1^H NMR (500 MHz, CDCl_3_): *δ* 7.74–7.79 (m, 2H), 7.58–7.61, (m, 1H), 7.52–7.54 (m, 1H), 7.42–7.46 (m, 2H), 7.34–7.36 (m, 2H), 7.07–7.08 (m, 1H), 7.01–7.03 (m, 2H), 6.85–6.89 (m, 1H), 6.24 (d, *J*_H-P_ = 24.12 Hz, 1H), 3.81 (s, 3H). ^31^P{^1^H} NMR (202 MHz, CDCl_3_): *δ* 37.76 (s); ^13^C{^1^H} NMR (125 MHz, CDCl_3_): *δ* 163.4 (s, C), 159.1 (s, C), 158.2 (d, *J*_C-P_ = 15.4 Hz, C), 144.8 (d, *J*_C-P_ = 29.9 Hz, C), 132.3 (d, *J*_C-P_ = 2.7 Hz, CH), 130.9 (d, *J*_C-P_ = 10.9 Hz, CH), 130.8 (d, *J*_C-P_ = 11.8 Hz, CH), 129.3 (d, *J*_C-P_ = 103.5 Hz, C), 129.2 (s, CH), 128.8 (d, *J*_C-P_ = 12.7 Hz, CH), 125.4 (d, *J*_C-P_ = 17.3 Hz, C), 123.8 (d, *J*_C-P_ = 112.6 Hz, C), 120.0 (d, *J*_C-P_ = 101.7 Hz, CH), 116.1 (s, CH), 113.2 (d, *J*_C-P_ = 11.8 Hz, CH), 112.1 (d, *J*_C-P_ = 12.7 Hz, CH), 55.6 (s, CH_3_). HRMS (ESI/Q-TOF) *m*/*z*: calcd for C_21_H_18_O_3_P [M + H]^+^, 349.0994, found: 349.0995.

#### 3-(*m*-Aminophenyl)-5-methoxy-1-phenylbenzophosphole Oxide (**8i**)

**3d** (0.0542 g, 0.134 mmol) was reacted with *m*-H_2_N-C_6_H_4_B(OH)_2_-H_2_O (0.0248 g, 0.16 mmol) according to general procedure B to afford **8i** (85%, 0.0395 g, 0.114 mmol) as an orange oil. *R*_*f*_ = 0.33 (30:5:1 CHCl_3_/AcOEt/MeOH). ^1^H NMR (500 MHz, CDCl_3_): *δ* 7.72–7.77 (m, 2H), 7.57–7.59 (m, 1H), 7.49–7.53 (m, 1H), 7.40–7.43 (m, 2H), 7.23–7.25 (m, 1H), 7.03–7.04 (m, 1H), 6.79–6.91 (m, 4H), 6.32 (d, d, *J*_H-P_ = 23.96 Hz, 1H), 4.07 (bs, 1H), 3.80 (s, 3H). ^31^P{^1^H} NMR (202 MHz, CDCl_3_): *δ* 35.89 (s); ^13^C{^1^H} NMR (125 MHz, CDCl_3_): *δ* 163.5 (d, *J*_C-P_ = 1.8 Hz, C), 157.4 (d, *J*_C-P_ = 14.5 Hz, C), 146.3 (s, C), 144.6 (d, *J*_C-P_ = 28.2 Hz, C), 135.9 (d, *J*_C-P_ = 16.4 Hz, C), 132.0 (d, *J*_C-P_ = 2.7 Hz, CH), 130.9 (d, *J*_C-P_ = 10.9 Hz, 2CH), 130.6 (d, *J*_C-P_ = 10.9 Hz, CH), 129.6 (s, CH), 128.7 (d, *J*_C-P_ = 10.9 Hz, 2CH), 124.4 (d, *J*_C-P_ = 111.7 Hz, C), 123.9 (d, *J*_C-P_ = 99 Hz, CH), 118.2 (s, CH), 116.4 (s, CH), 112.9 (d, *J*_C-P_ = 11.8 Hz, CH), 111.9 (d, d, *J*_C-P_ = 11.8 Hz, CH), 55.6 (s, CH_3_). HRMS (ESI/Q-TOF) *m*/*z*: calcd for C_21_H_19_O_2_NP [M + H]^+^, 348.1204, found: 348.1204.

#### 1-Phenyl-5-methoxy-3-(*p*-nitrophenyl)benzophosphole Oxide (**8n**)

**3d** (0.0542 g, 0.134 mmol) was reacted with *m*-O_2_N-C_6_H_4_B(OH)_2_ (0.0268 g, 0.16 mmol) according to general procedure B to afford **8n** (88% according to the ^1^H NMR spectrum, 0.047 g) as a mixture with 5% of Ph_3_P(O). *R*_*f*_ = 0.21 (30:1 CHCl_3_/MTBE). ^1^H NMR (500 MHz, CDCl_3_): *δ* 8.34–8.40 (m, 2H), 7.86–7.88 (m, 1H), 7.73–7.79 (m, 2H), 7.66–7.71 (m, 2H), 7.55–7.59 (m, 1H), 7.45–7.49 (m, 2H), 6.92–6.94 (m, 1H), 6.87–6.88 (m, 1H), 6.52 (d, *J*_H-P_ = 22.54 Hz, 1H), 3.85 (s, 3H). ^31^P{^1^H} NMR (202 MHz, CDCl_3_): *δ* 35.58 (s); ^13^C{^1^H} NMR (125 MHz, CDCl_3_): *δ* 163.8 (d, *J*_C-P_ = 1.8 Hz, C), 154.3 (d, *J*_C-P_ = 16.4 Hz, C), 148.5 (s, C), 143.5 (d, *J*_C-P_ = 28.2 Hz, C), 136.5 (d, *J*_C-P_ = 16.4 Hz, C), 133.8 (s, CH), 132.4 (d, *J*_C-P_ = 2.7 Hz, CH), 132.1 (d, *J*_C-P_ = 10.0 Hz, CH), 131.4 (d, *J*_C-P_ = 11.8 Hz, CH), 131.0 (d, *J*_C-P_ = 10.9 Hz, 2CH), 130.1 (s, CH), 128.9 (d, *J*_C-P_ = 12.7 Hz, 2CH), 127.0 (d, *J*_C-P_ = 97.2 Hz, CH), 124.2 (s, CH), 123.9 (d, *J*_C-P_ = 112.6 Hz, C), 122.8 (s, CH), 113.2 (d, *J*_C-P_ = 11.8 Hz, CH), 111.5 (d, *J*_C-P_ = 11.8 Hz, CH), 55.7 (s, CH_3_). HRMS (ESI/LQT) *m*/*z*: calcd for C_21_H_17_O_4_NP [M + H]^+^, 378.0895, found: 378.0894.

### C: Procedure for the Reaction of **3b** with **4n** in a Higher Scale

To a reaction vial (30 mL) equipped with a magnetic stirrer and an argon inlet, **3b** (0.389 g, 1 mmol), *m*-NO_2_-C_6_H_4_B(OH)_2_ (**4n**) (0.2 g, 1.2 mmol), K_2_CO_3_ (0.276 g, 2.0 mmol), and Pd(PPh_3_)_4_ (58.0 mg, 0.05 mmol) were added followed by DME (10 mL). The vial was closed using an aluminum cap and heated at 110 °C using heating transfer blocks for 24 h. After that time, the reaction was cooled to rt, and the solvent was evaporated. To the residue, a saturated solution of NaHCO_3_ (10 mL) and DCM (10 mL) was added, and the mixture was transferred to an extraction funnel. The mixture was extracted with DCM (3 × 15 mL), and the collected organic phases were dried over Na_2_SO_4_. The solid was filtered off, and the filtrate evaporated under reduced pressure. The crude reaction mixture was checked using the NMR technique. The crude product was purified by column chromatography on silica gel using CHCl_3_/MTBE (30:1 v/v) as the eluent.

#### 3-(*m*-Nitrophenyl)-7-methyl-1-phenylbenzophosphole Oxide (**6n**)

**3b** was reacted according to general procedure C to afford **6n** (91%, 0.329 g, 0.91 mmol).

### D: Procedure for the Reaction of **3a** with **4a** Using Pd(PPh_3_)_4_ in Different Solvents (Table 1, Entries 1–11)

To a reaction vial (5 mL) equipped with a magnetic stirrer and an argon inlet, **3a** (0.0375 g, 0.1 mmol), PhB(OH)_2_**4a** (0.0195 g, 0.12 mmol), base (0.2 mmol), and Pd(PPh_3_)_4_ (7.7 mg, 5.0 μmol) were added followed by the solvent (1 mL). The vial was closed using an aluminum cap and heated at indicated temperature using heating blocks for 24 h. After that time, the reaction was cooled to rt, and the solvent was evaporated. To the residue, a saturated solution of NaHCO_3_ (5 mL) and DCM (10 mL) were added, and the mixture was transferred to an extraction funnel. The mixture was extracted with DCM (3 × 15 mL), and the collected organic phases were dried over Na_2_SO_4_. The solid was filtered off, and the filtrate evaporated under reduced pressure. The crude reaction mixture was checked using the NMR technique. The crude product was purified by column chromatography on silica gel using CHCl_3_/MTBE (30:1 v/v) as an eluent.

#### 1,3-Diphenylbenzophosphole Oxide (**5a**)^13^

**3a** was reacted according to procedure D using K_2_CO_3_ (0.0276 g, 0.2 mmol) as a base in DMF at 110 °C to afford **5a** (85%, 0.026 g, 0.085 mmol).

#### 1,3-Diphenylbenzophosphole Oxide (**5a**)^13^

**3a** was reacted according to procedure D using K_2_CO_3_ (0.0276 g, 0.2 mmol) as a base in THF at 60 °C to afford **5a** (57%, 0.0172 g, 0.057 mmol).

#### 1,3-Diphenylbenzophosphole Oxide (**5a**)^13^

**3a** was reacted according to procedure D using K_2_CO_3_ (0.0276 g, 0.2 mmol) as a base in toluene at 80 °C to afford **5a** (52%, 0.0157 g, 0.052 mmol).

#### 1,3-Diphenylbenzophosphole Oxide (**5a**)^13^

**3a** was reacted according to procedure D using K_2_CO_3_ (0.0276 g, 0.2 mmol) as a base in toluene at 110 °C to afford **5a** (87%, 0.0263 g, 0.087 mmol).

#### 1,3-Diphenylbenzophosphole Oxide (**5a**)^13^

**3a** was reacted according to procedure D using K_2_CO_3_ (0.0276 g, 0.2 mmol) as a base in 1,4-dioxane at rt to afford **5a** (79%, 0.0239 g, 0.079 mmol).

#### 1,3-Diphenylbenzophosphole Oxide (**5a**)^13^

**3a** was reacted according to procedure D using K_2_CO_3_ (0.0276 g, 0.2 mmol) as a base in 1,4-dioxane at 80 °C to afford **5a** (74%, 0.0224 g, 0.074 mmol).

#### 1,3-Diphenylbenzophosphole Oxide (**5a**)^13^

**3a** was reacted according to procedure D using K_2_CO_3_ (0.0276 g, 0.2 mmol) as a base in DME at 50 °C to afford **5a** (55%, 0.0166 g, 0.055 mmol).

#### 1,3-Diphenylbenzophosphole Oxide (**5a**)^13^

**3a** was reacted according to procedure D using K_2_CO_3_ (0.0276 g, 0.2 mmol) as a base in DME at 80 °C to afford **5a** (61%, 0.0184 g, 0.061 mmol).

#### 1,3-Diphenylbenzophosphole Oxide (**5a**)^13^

**3a** was reacted according to procedure D using Na_2_CO_3_ (0.1 mL, 0.2 mmol, 2 M) as a base in DME at 110 °C to afford **5a** (62%, 0.0187 g, 0.062 mmol).

#### 1,3-Diphenylbenzophosphole Oxide (**5a**)^13^

**3a** was reacted according to procedure D using Na_2_CO_3_ (0.1 mL, 0.2 mmol, 2 M) as a base and LiCl (0.0127 g, 0.3 mmol) in DME at 110 °C to afford **5a** (60%, 0.0181 g, 0.06 mmol).

### E: Procedure for the Reaction of **3a** with **4a** Using Pd(OAc)_2_ as a Catalyst (Table 1, Entries 12–13)

To a reaction vial (5 mL) equipped with a magnetic stirrer and an argon inlet, **3a** (0.1 g, 0.268 mmol), PhB(OH)_2_ (**4a**) (0.039 g, 0.32 mmol), KF (0.031 g, 0.543 mmol), Pd(OAc)_2_ (1.2 mg, 5.3 μmol), and ligand (6.4 μmol) were added followed by the solvent (1 mL). The vial was closed using an aluminum cap and heated at 85 °C using heating transfer blocks for 24 h or stirred at rt for 24 h. After that time, the solvent was evaporated. To the residue, a saturated solution of NaHCO_3_ (5 mL) and DCM (10 mL) was added, and the mixture was transferred to an extraction funnel. The mixture was extracted with DCM (3 × 10 mL), and the collected organic phases were dried over Na_2_SO_4_. The solid was filtered off and the filtrate was evaporated under reduced pressure. The crude reaction mixture was checked using the NMR technique. The crude product was purified by column chromatography on silica gel using CHCl_3_/MTBE (30:1 v/v) as an eluent.

#### 1,3-Diphenylbenzophosphole Oxide (**5a**)^13^

**3a** was reacted according to procedure E using PCy_3_ (1.8 mg, 6.4 μmol) as a ligand in DME at 85 °C for 24 h to afford **5a** (21%, 0.0167 g, 0.056 mmol).

#### 1,3-Diphenylbenzophosphole Oxide (**5a**)^13^

**3a** was reacted according to procedure E using PTol_3_ (2.0 mg, 6.4 μmol) as a ligand in THF at rt for 24 h to afford **5a** (17%, 0.0137 g, 0.0454 mmol) and **2a** (24%, 0.011 g, 0.045 mmol).

#### 1-Phenylbenzophosphole Oxide (**2a**)^42^

^1^H NMR (500 MHz, CDCl_3_): *δ* 8.05–8.07 (m, 1H), 7.85–7.90 (m, 1H), 7.77–7.83 (m, 2H), 7.55–7.60 (m, 3H), 7.45–7.47 (m, 2H), 3.12–3.31 (m, 2H). ^31^ P NMR (202 MHz, CDCl_3_): *δ* 29.92 (s). ^13^C NMR (125 MHz, CDCl_3_): *δ* 194.3 (d, *J*_C-P_ = 12.7 Hz, C), 141.5 (d, *J*_C-P_ = 80.8 Hz, C), 141.3 (d, *J*_C-P_ = 23.6 Hz, C), 135.9 (d, *J*_C-P_ = 10.9 Hz, CH), 133.6 (d, *J*_C-P_ = 2.7 Hz, CH), 132.6 (d, *J*_C-P_ = 2.7 Hz, CH), 130.8 (d, *J*_C-P_ = 105.4 Hz, C), 130.6 (d, *J*_C-P_ = 10.9 Hz, 2CH), 129.4 (d, *J*_C-P_ = 6.4 Hz, CH), 128.9 (d, *J*_C-P_ = 13.6 Hz, 2CH), 124.7 (d, *J*_C-P_ = 10.9 Hz, CH), 40.1 (d, *J*_C-P_ = 71.8 Hz, CH_2_).

### F: Procedure for the Reaction of **3a** with **4a** Using PdCl_2_ as a Catalyst (Table 1, Entry 14)

To a reaction vial (5 mL) equipped with a magnetic stirrer and an argon inlet, **3a** (0.1 g, 0.268 mmol), PhB(OH)_2_ (**4a**) (0.039 g, 0.32 mmol), Na_2_CO_3_ (0.0566 g, 0.543 mmol), PdCl_2_ (2.4 mg, 13.3 μmol), and PTol_3_ (4.1 mg, 13.3 μmol) were added followed by THF (1 mL). The vial was closed and heated at 40 °C using an oil bath for 24 h. After that time, the reaction was cooled to rt, and the solvent was evaporated. To the residue, a saturated solution of NaHCO_3_ (5 mL) and DCM (10 mL) was added, and the mixture was transferred to an extraction funnel. The mixture was extracted with DCM (3 × 10 mL), and the collected organic phases were dried over Na_2_SO_4_. The solid was filtered off, and the filtrate evaporated under reduced pressure. The crude reaction mixture was checked using the NMR technique. The crude product was purified by column chromatography on silica gel using CHCl_3_/MTBE (30:1 v/v) as an eluent.

#### 1,3-Diphenylbenzophosphole Oxide (**5a**)^13^

**3a** was reacted according to procedure F to afford **5a** (13%, 0.0105 g, 0.0345 mmol) and **2a** (13%, 0.008 g, 0.0345 mmol).

## Data Availability

The data underlying this study are available in the published article and its Supporting Information.

## Supporting Information Available

^1^H NMR, ^13^C{^1^H} NMR, and ^31^P{^1^H} NMR spectra for all products (**2a**, **5a–n**, **6a–b**,**e**,**h–j**,**n**, **7a**, **8a–b**,**h–i**,**n**) and starting materials (**3a–d**); crystallographic data (**3d**, **5n**, **6n**); and data/figures obtained from DFT and TD-DFT calculations at the B3LYP/6–31+G(d,p) level of theory (PDF)
