# Supplementary material for: Benzophosphol-3-yl Triflates as Precursors of 1,3-Diarylbenzophosphole Oxides
Source: J Org Chem. 2023 Jun 5;88(13):7901–17. doi: 10.1021/acs.joc.2c02355 (PMC10337042; doi:10.1021/acs.joc.2c02355)
Supplement: Supplementary file 1 — jo2c02355_si_001.pdf [file jo2c02355_si_001.pdf]

Supporting information

Benzophosphol-3-yl triflates as precursors of 1,3-diarylbenzophosphole oxides

Agnieszka Brzyska,[a] Sebastian Majewski,[b] Łukasz Ponikiewski,[c] Monika Zubik-Duda,[d] Agnieszka Lipke,[e] Agnieszka Gładysz-Płaska,[f] Sylwia Sowa[b,*]

[a]*Jerzy Haber Institute of Catalysis and Surface Chemistry, Polish Academy of Sciences, 8 Niezapominajek St., Krakow PL-30-239, Poland*

[b]*Department of Organic Chemistry and Crystallochemistry, Faculty of Chemistry, Institute of Chemical Sciences, Maria Curie-Sklodowska University in Lublin, 33 Gliniana St., Lublin PL-20-614, Poland*

[c]*Department of Inorganic Chemistry, Faculty of Chemistry, Gdańsk University of Technology, 11/12 G. Narutowicza St., Gdańsk PL-80-233, Poland*

[d]*Department of Biophysics, Institute of Physics, Maria Curie-Sklodowska University in Lublin, PL-20-031 Lublin, Poland*

[e]*Institute of Chemical Sciences, Maria Curie-Sklodowska University in Lublin, 2/9 M. Curie-Sklodowska sq., Lublin PL-20-031, Poland*

[f]*Department of Inorganic Chemistry, Faculty of Chemistry, Institute of Chemical Sciences, Maria Curie-Sklodowska University in Lublin, 2/13-15A M. Curie-Sklodowska sq., Lublin PL-20-031, Poland*

**E-mail*: sylwia.sowa@mail.umcs.pl

Table of contents:

Copies of ^{1}H NMR ^{13}C{^{1}H} NMR and ^{31}P{^{1}H} NMR spectra of products	S3-S113
1-Phenylbenzophospholan-3-one oxide (**2a**)	S4-S6
1,3-Diphenylbenzophosphole oxide (**5a**)	S7-S10
1-Phenyl-3-(*p*-tolyl)benzophosphole oxide (**5b**)	S11-S14
1-Phenyl-3-(*m*-tolyl)benzophosphole oxide (**5c**)	S15-S18
1-Phenyl-3-(*o*-tolyl)benzophosphole oxide (**5d**)	S19-S22
1-Phenyl-3-(*p*-anisyl)benzophosphole oxide (**5e**)	S23-S26
1-Phenyl-3-(*m*-anisyl)benzophosphole oxide (**5f**)	S27-S30
1-Phenyl-3-(*o*-anisyl)benzophosphole oxide (**5g**)	S31-S34
1-Phenyl-3-(*p*-hydroxyphenyl)benzophosphole oxide (**5h**)	S35-S38
1-Phenyl-3-(*m*-aminophenyl)benzophosphole oxide (**5i**)	S39-S42
1-Phenyl-3-(*p*-fluorophenyl)benzophosphole oxide (**5j**)	S43-S46
1-Phenyl-3-(*m*-fluorophenyl)benzophosphole oxide (**5k**)	S47-S50
1-Phenyl-3-(*o*-fluorophenyl)benzophosphole oxide (**5l**)	S51-S54
1-Phenyl-3-(*p*-chlorophenyl)benzophosphole oxide (**5m**)	S55-S58
1-Phenyl-3-(*m*-nitrophenyl)benzophosphole oxide (**5n**)	S59-S62
1,3-Diphenyl-7-methylbenzophosphole oxide (**6a**)	S63-S66
7-Methyl-1-phenyl-3-(*p*-tolyl)benzophosphole oxide (**6b**)	S67-S70
1-Phenyl-3-(*p*-anisyl)benzophosphole oxide (**6e**)	S71-S74
3-(*p*-Hydroxyphenyl)-7-methyl-1-phenylbenzophosphole oxide (**6h**)	S75-S78
3-(*p*-Aminophenyl)-7-methyl-1-phenylbenzophosphole oxide (**6i**)	S79-S82
3-(*p*-Fluorophenyl)-7-methyl-1-phenylbenzophosphole oxide (**6j**)	S83-S86
3-(*m*-Nitrophenyl)-7-methyl-1-phenylbenzophosphole oxide (**6n**)	S87-S90
6-Chloro-1,3-diphenylbenzophosphole oxide (**7a**)	S91-S94
1,3-Diphenyl-5-methoxybenzophosphole oxide (**8a**)	S95-S98
1-Phenyl-5-methoxy-3-(*p*-tolyl)benzophosphole oxide (**8b**)	S99-S102
3-(*p*-Hydroxyphenyl)-1-phenyl-5-methoxybenzophosphole oxide (**8h**)	S103-S106
3-(*m*-Aminophenyl)-1-phenyl-5-methoxybenzophosphole oxide (**8i**)	S107-110
1-Phenyl-5-methoxy-3-(*p*-nitrophenyl)benzophosphole oxide (**8n**)	S111-S114
Copies of ^{1}H NMR ^{13}C{^{1}H} NMR and ^{31}P{^{1}H} NMR spectra of starting materials	S115-S126
1-Oxido-1-phenyl-1H-phosphindol-3-yl trifluoromethanesulfonate (**3a**).	S115-S117
1-Oxido-1-phenyl-1H-7-methylphosphindol-3-yl trifluoromethanesulfonate (**3b**)	S118-S120
1-Oxido-1-phenyl-1H-6-chlorophosphindol-3-yl trifluoromethanesulfonate (**3c**)	S121-S123
1-Oxido-1-phenyl-1H-5-methoxyphosphindol-3-yl trifluoromethanesulfonate (**3d**)	S124-S126
Fig. S1a. Molecular structure of **3d**	S127
Fig. S1b. Molecular structure of **3d** showing intramolecular bond	S127
Fig. S1c. Molecular structure of **3d**, showing intra- and intermolecular hydrogen bonds	S128
Table S1. The intra- and intermolecular hydrogen bonds lengths	S128
Fig. S2. Molecular structure of **5n**	S129
Fig. S3. Molecular structure of **6n**	S130
Table S2. Crystallographic data for **3d**, **5n** and **6n**	S131
Fig. S4. a) Normalized absorption spectra of **5e** in different solvents at 10^{-5} M b) Normalized fluorescence spectra of **5e** in different solvents at 10^{-5} M	S132
Fig. S5. a) Normalized absorption spectra of **5j** in different solvents at 10^{-5} M b) Normalized fluorescence spectra of **5j** in different solvents at 10^{-5} M.	S133
Fig. S6. a) Normalized absorption spectra of **5h** in different solvents at 10^{-5} M b) Normalized fluorescence spectra of **5h** in different solvents at 10^{-5} M	S134
Fig. S7. a) Normalized absorption spectra of **5n** in different solvents at 10^{-5} M b) Normalized fluorescence spectra of **5n** in different solvents at 10^{-5} M	S135

Fig. S8. Lippert-Mataga plot for **5a,e,j,h,n.** S136

Fig. S9. a) Normalized absorption spectra of **5a,j** in THF at 10^{-4} M. b) Normalized fluorescence spectra of **5a,j** in THF at 10^{-4} M S137

Fig. S10 The HOMO-LUMO frontier molecular orbitals for **5a-n** obtained at DFT/B3LYP/6-31+G(d,p) level S138

Fig. S11 The correlation diagram (H-L gap vs. excitation energy) S139

Table S3. The calculation of energy gap from excitation energy (S_1-S_0) S139

Fig. S12. HOMO (orange) and LUMO (blue) energies of the **5** compound series vs. Hammet's σ_p of para-substituent S140

Table S4. The experimental and computed UV-Vis parameters and electronic transitions for **5a,e,h,j,n** compounds S140

Fig. S13. Calculated distribution patterns of the HOMOs-1, HOMOs and LUMOs of **5e** and **5h** at the ground states in gas phase and the transitions related to most probable absorption peaks S141

Table S5. The comparison of dipole moments of S_0 and S_1 states for **5a,e,h,j,n** S141

Table S6. Geometries of S_0 vs S_1 state for **5a,e,h,j,n** S142-144

Table S7. Dihedral (tortion) angles (φ_t) for **5a,e,h,j,n** S144

Additional comments to geometries of S_0 and S_1 for **5a,e,h,j,n** S145

Table S8. Some calculated bonds length and Mulliken charges at selected atoms in **5a,e,h,j,n** S146

Fig. S14. The Changes of charge in the conjugated bond system from S_0 to S_1. S146

Table S9. The ground state (minima) geometries for the analyzed compounds (optimized at the DFT/B3LYP/6-31+g(d,p) level) 147-157 S147-157

Table 10. The equilibrium geometry coordinates (XYZ) for S1 states for the analyzed compounds S158-162

References S163

Copies of ^{1}H NMR ^{13}C{^{1}H} NMR and ^{31}P{^{1}H} NMR spectra of compounds

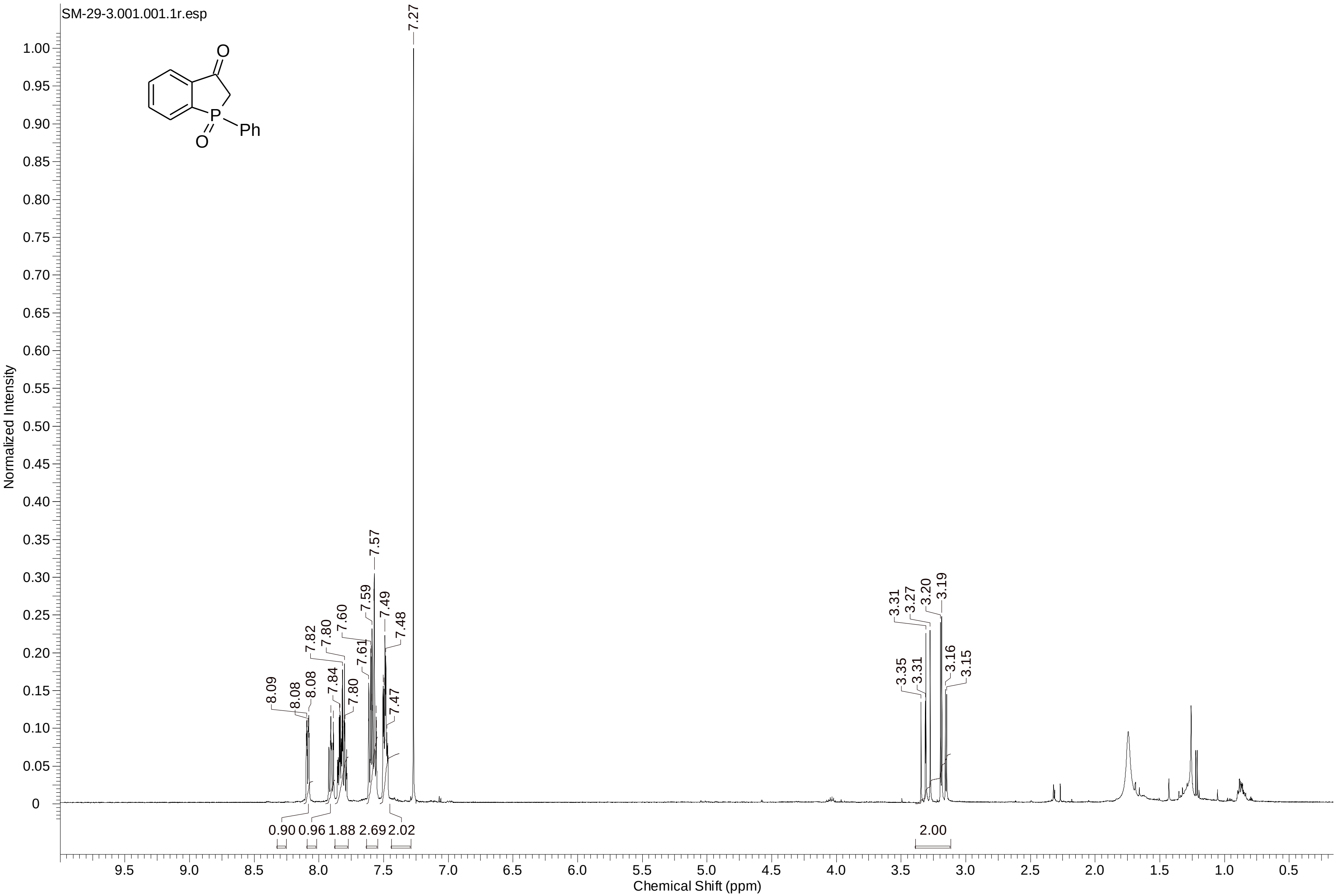

^1H NMR spectrum of 1-phenylbenzophospholan-3-one oxide (**2a**) (500 MHz, $CDCl_3$)[1]

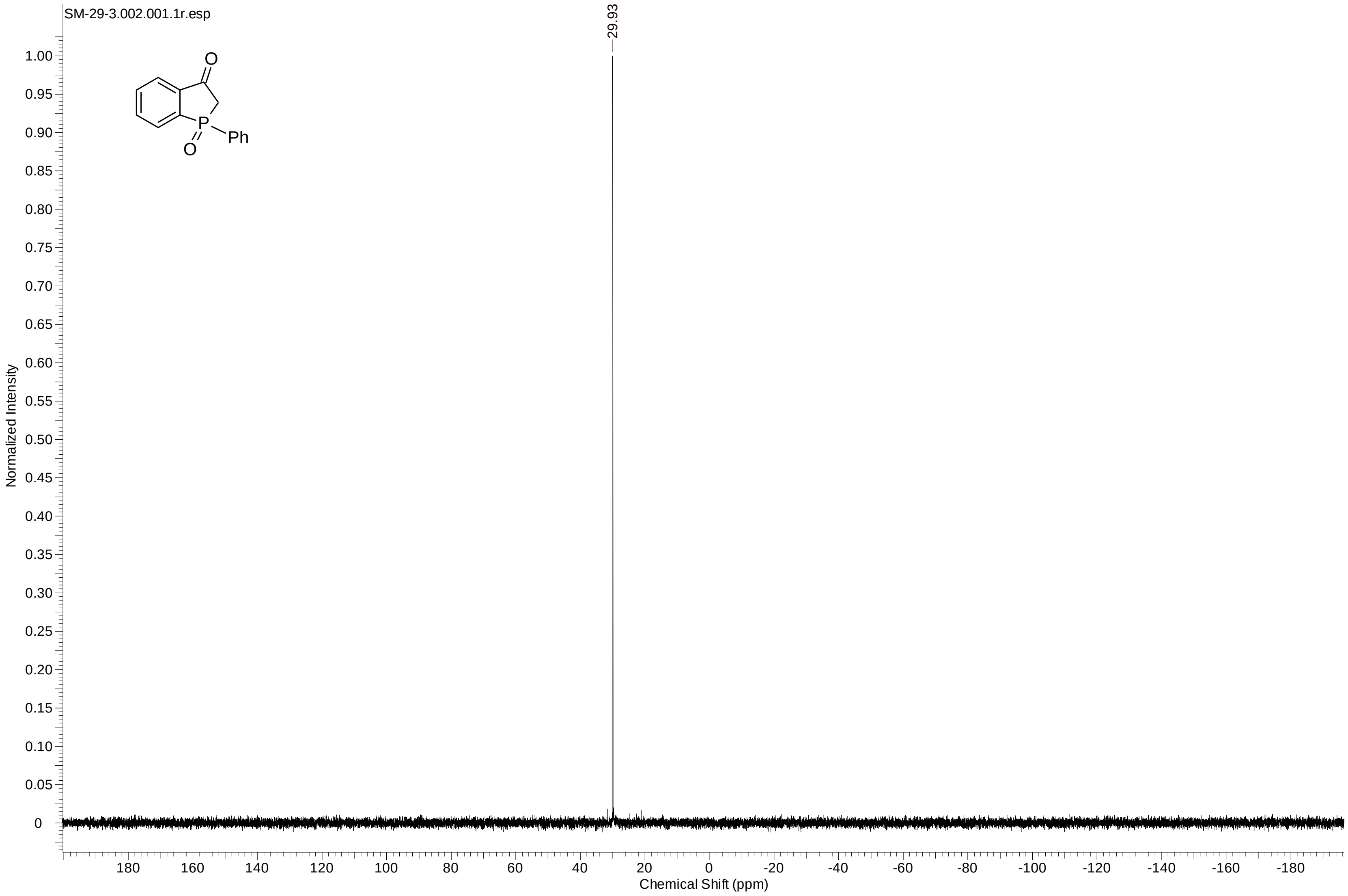

$^{31}P\{^1H\}$ NMR spectrum of 1-phenylbenzophospholan-3-one oxide (**2a**) (202 MHz, $CDCl_3$)[1]

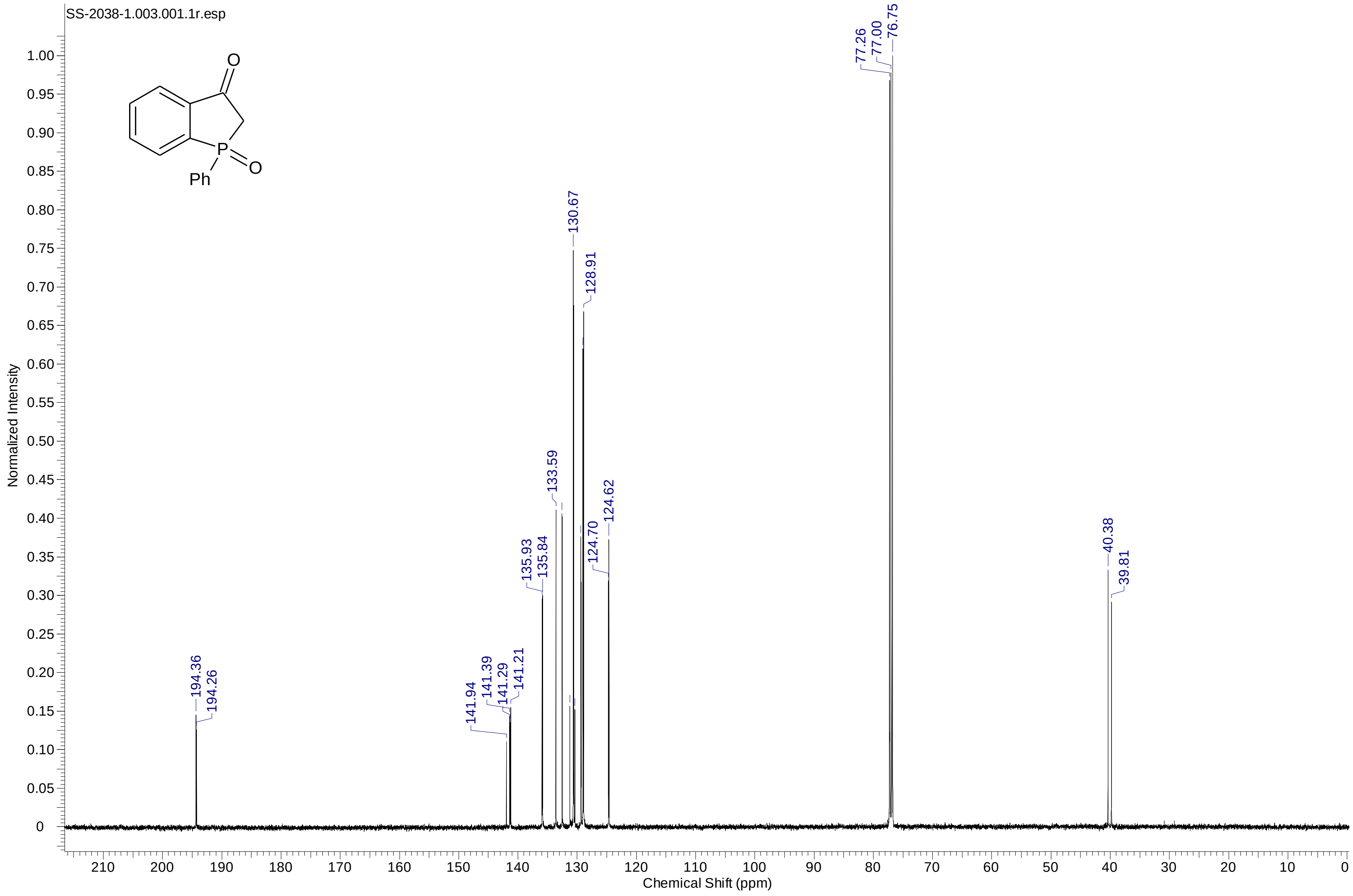

$^{13}C\{^1H\}$ NMR spectrum of 1-phenylbenzophospholan-3-one oxide (**2a**) (202 MHz, $CDCl_3$)[1]

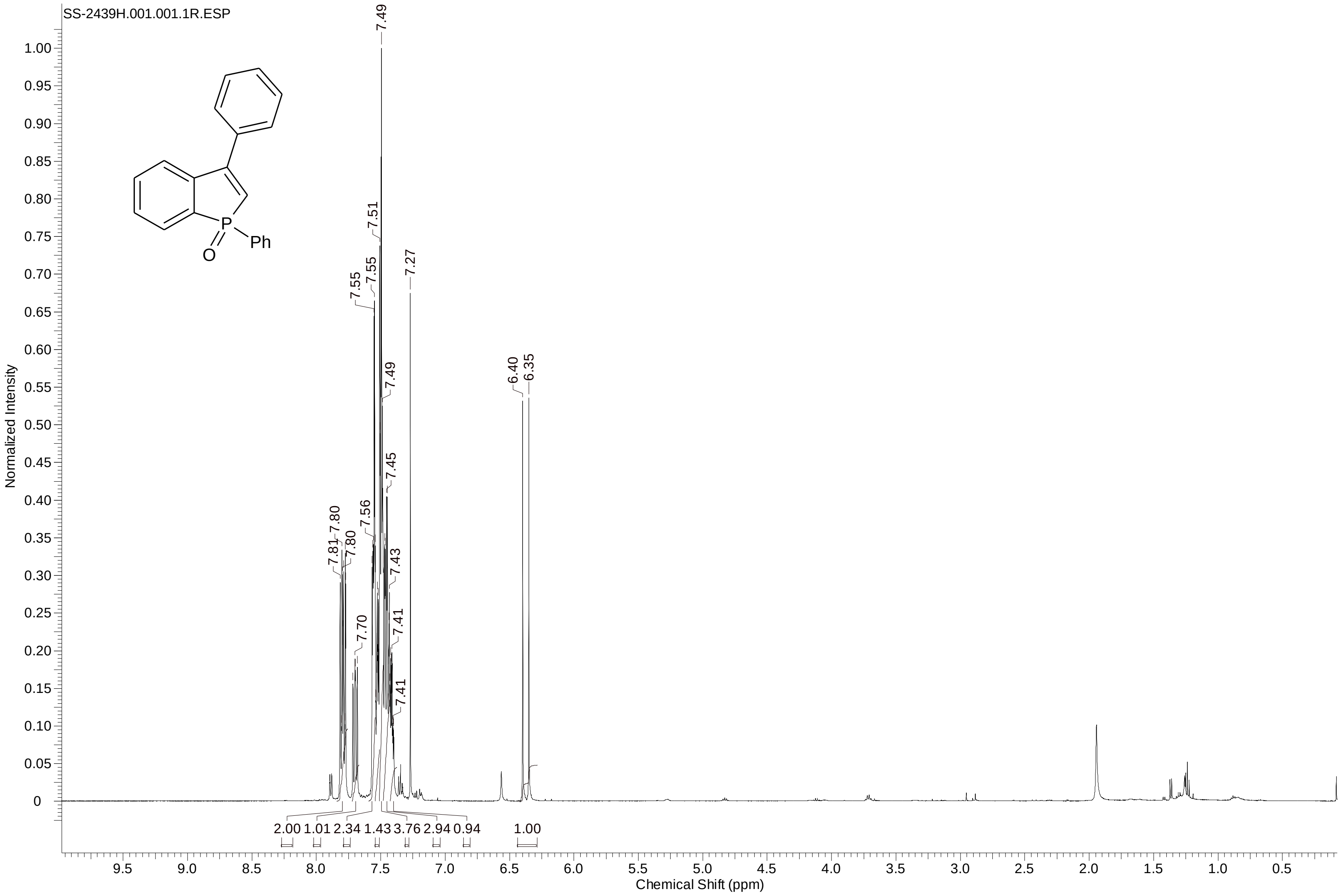

^{1}H NMR spectrum of 1,3-diphenylbenzophosphole oxide (**5a**) (500 MHz, $CDCl_3$)[2]

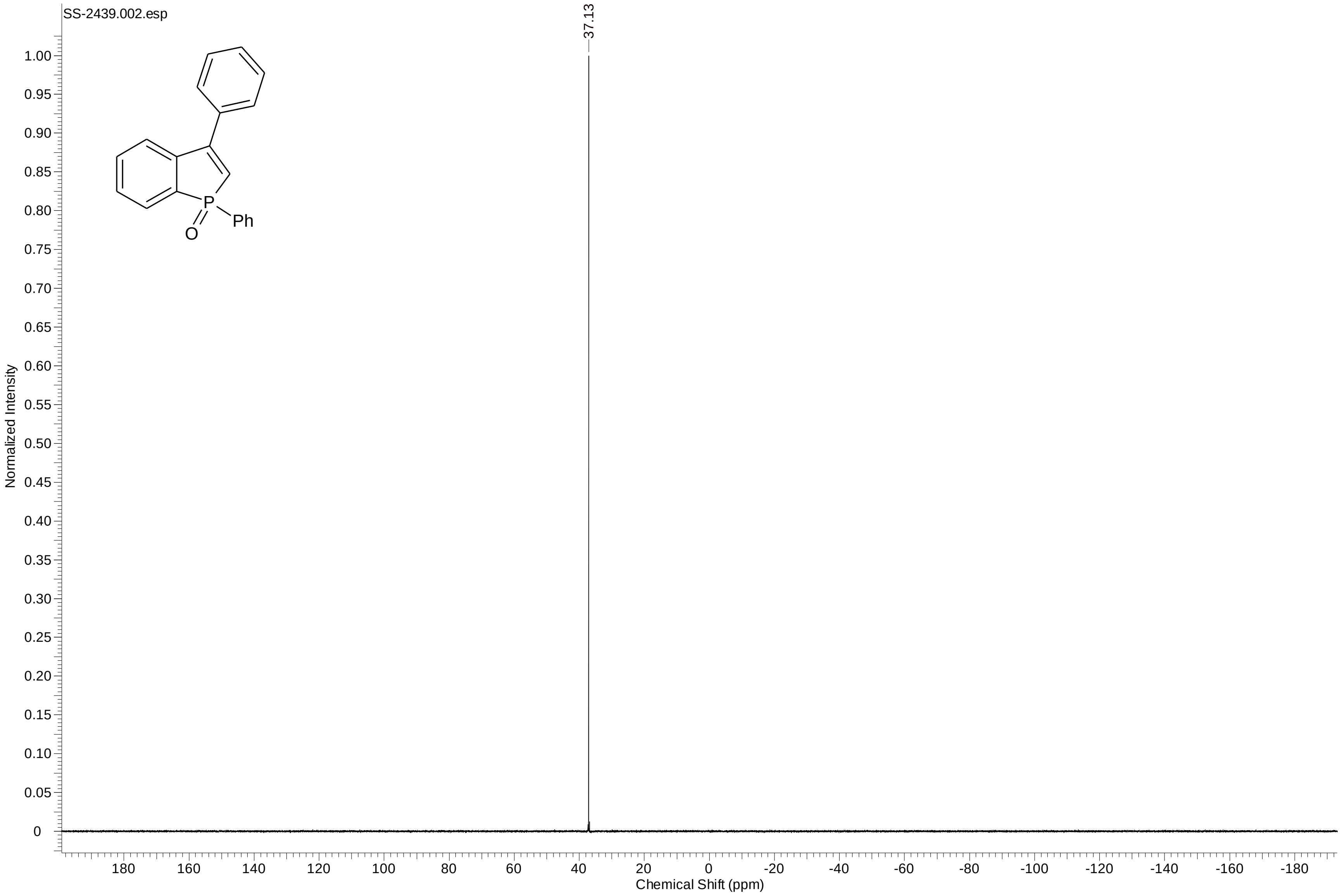

$^{31}P\{^{1}H\}$ NMR spectrum of 1,3-diphenylbenzophosphole oxide (**5a**) (202 MHz, $CDCl_3$)

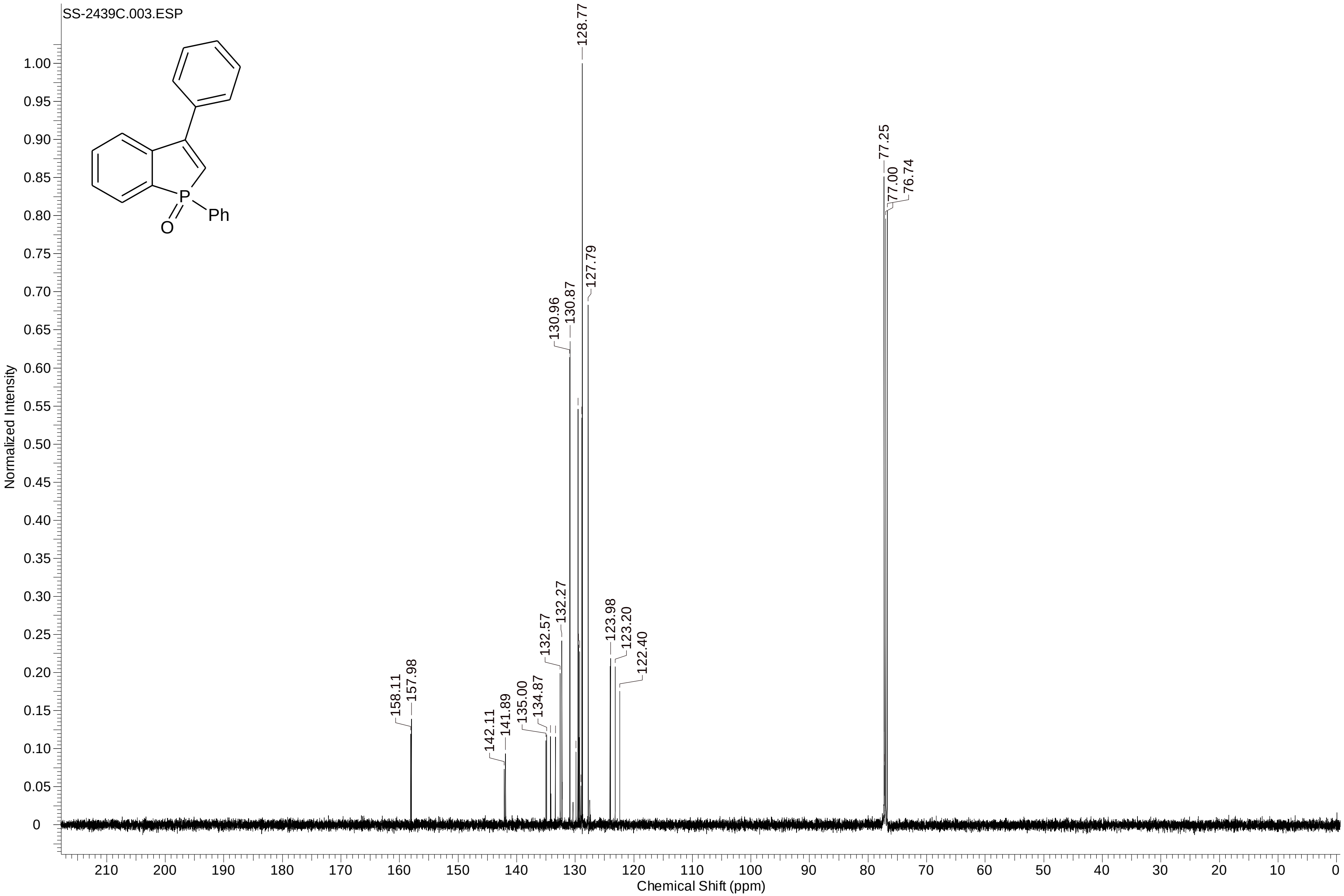

$^{13}C\{^{1}H\}$ NMR spectrum of 1,3-diphenylbenzophosphole oxide (**5a**) (125 MHz, $CDCl_3$)

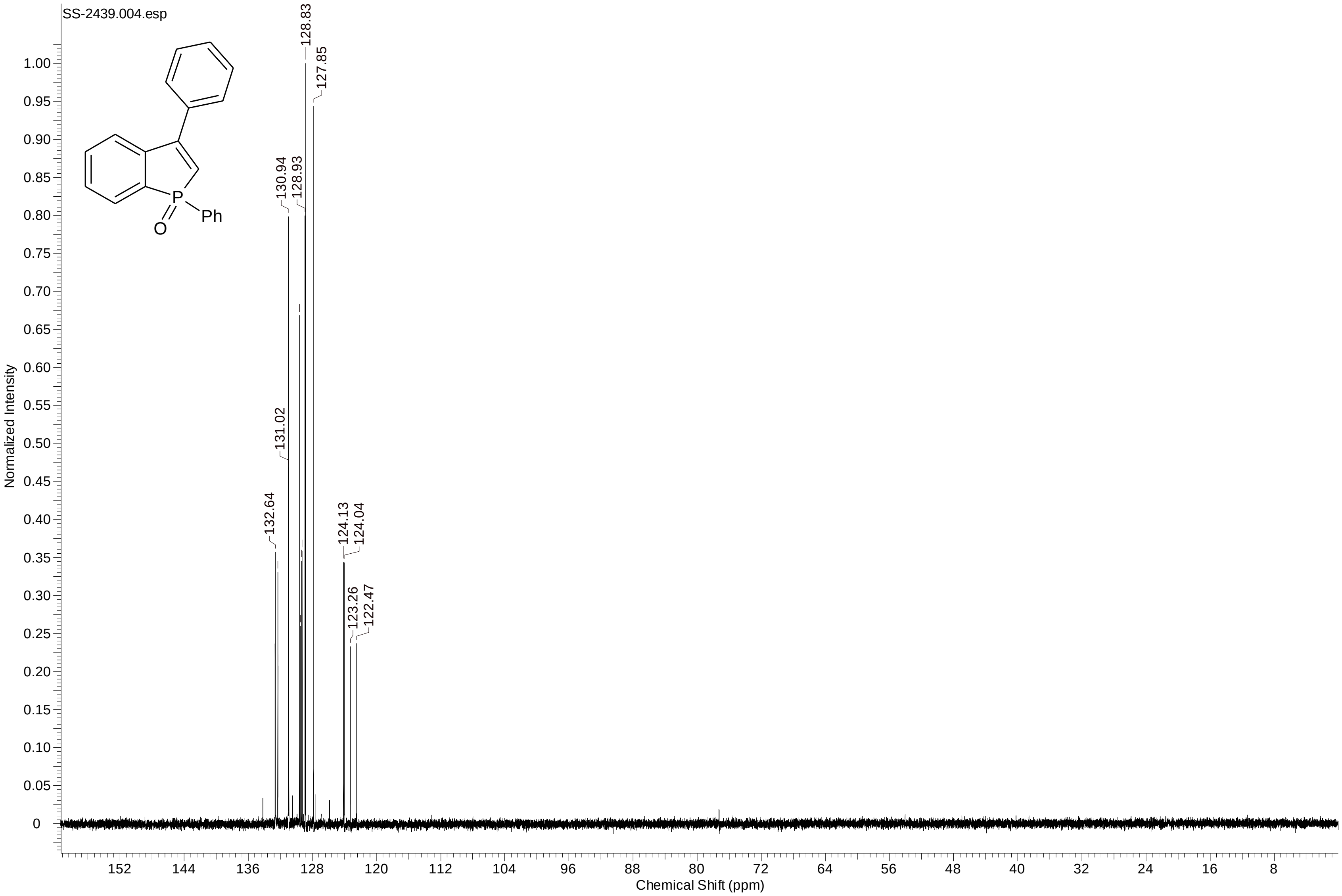

DEPT 135 NMR spectrum of 1,3-diphenylbenzophosphole oxide (**5a**) (125 MHz, $CDCl_3$)

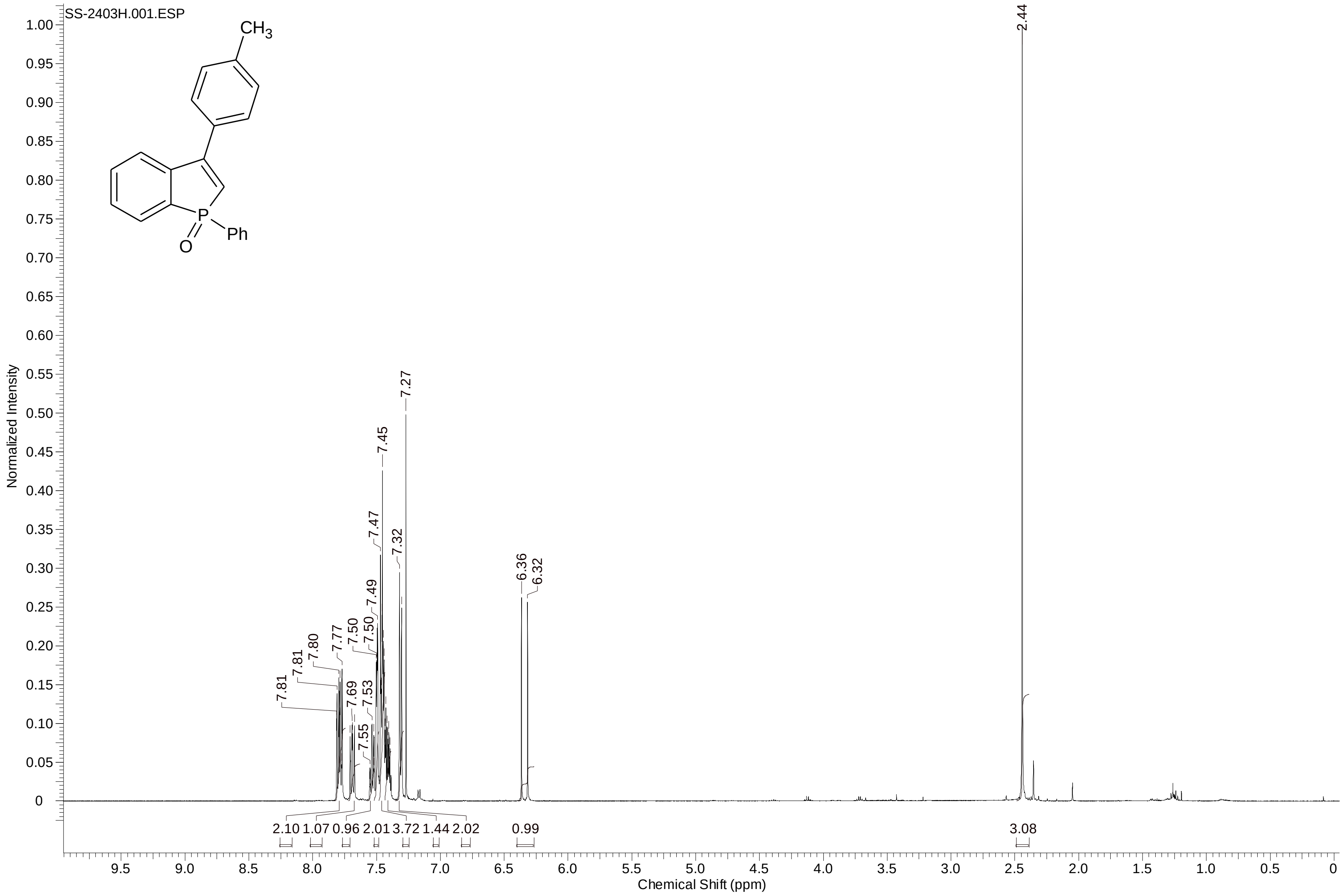

^{1}H NMR spectrum of 1-phenyl-3-(*p*-tolyl)benzophosphole oxide (**5b**) (500 MHz, $CDCl_3$)

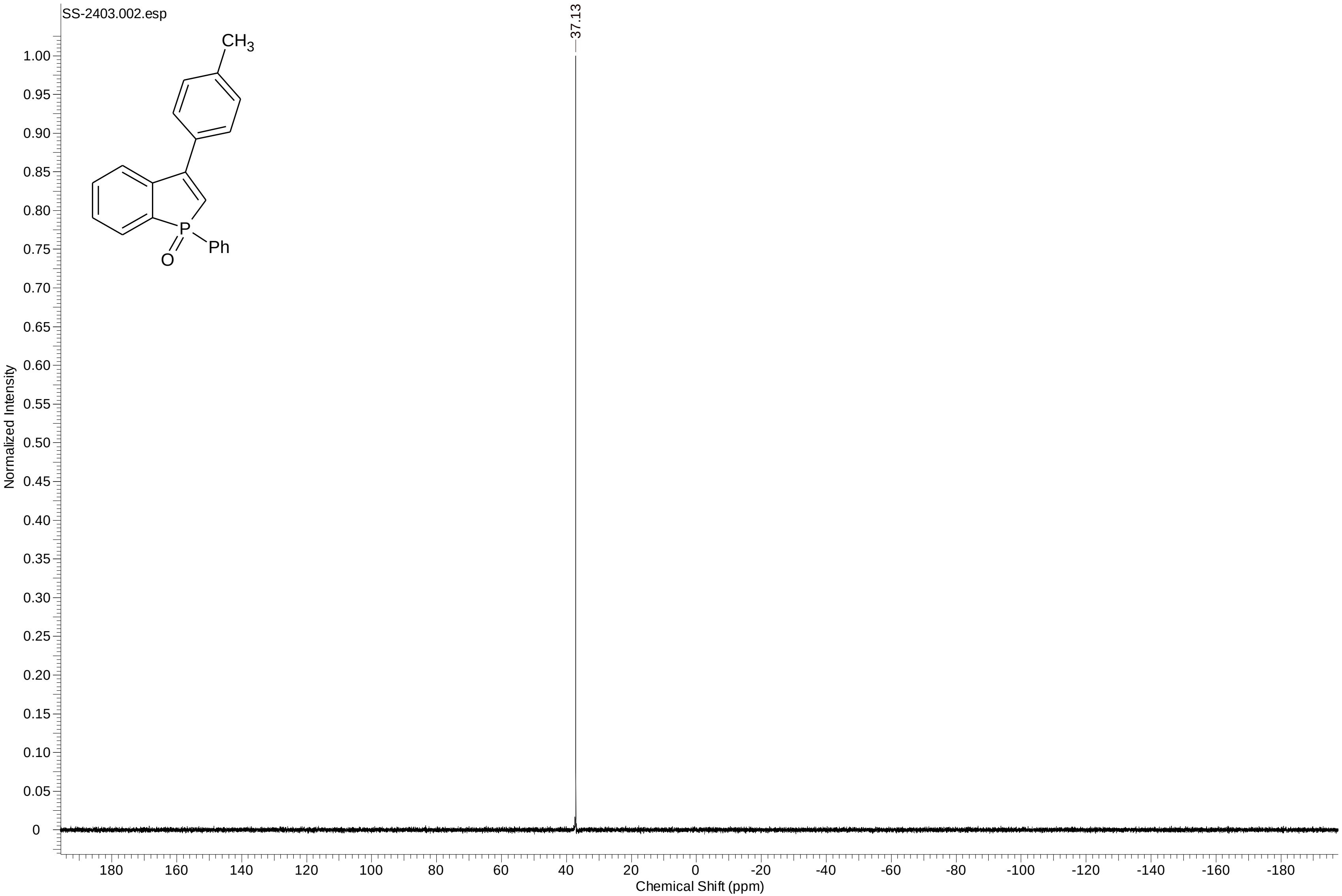

$^{31}P\{^{1}H\}$ NMR spectrum of 1-phenyl-3-(*p*-tolyl)benzophosphole oxide (**5b**) (202 MHz, $CDCl_3$)

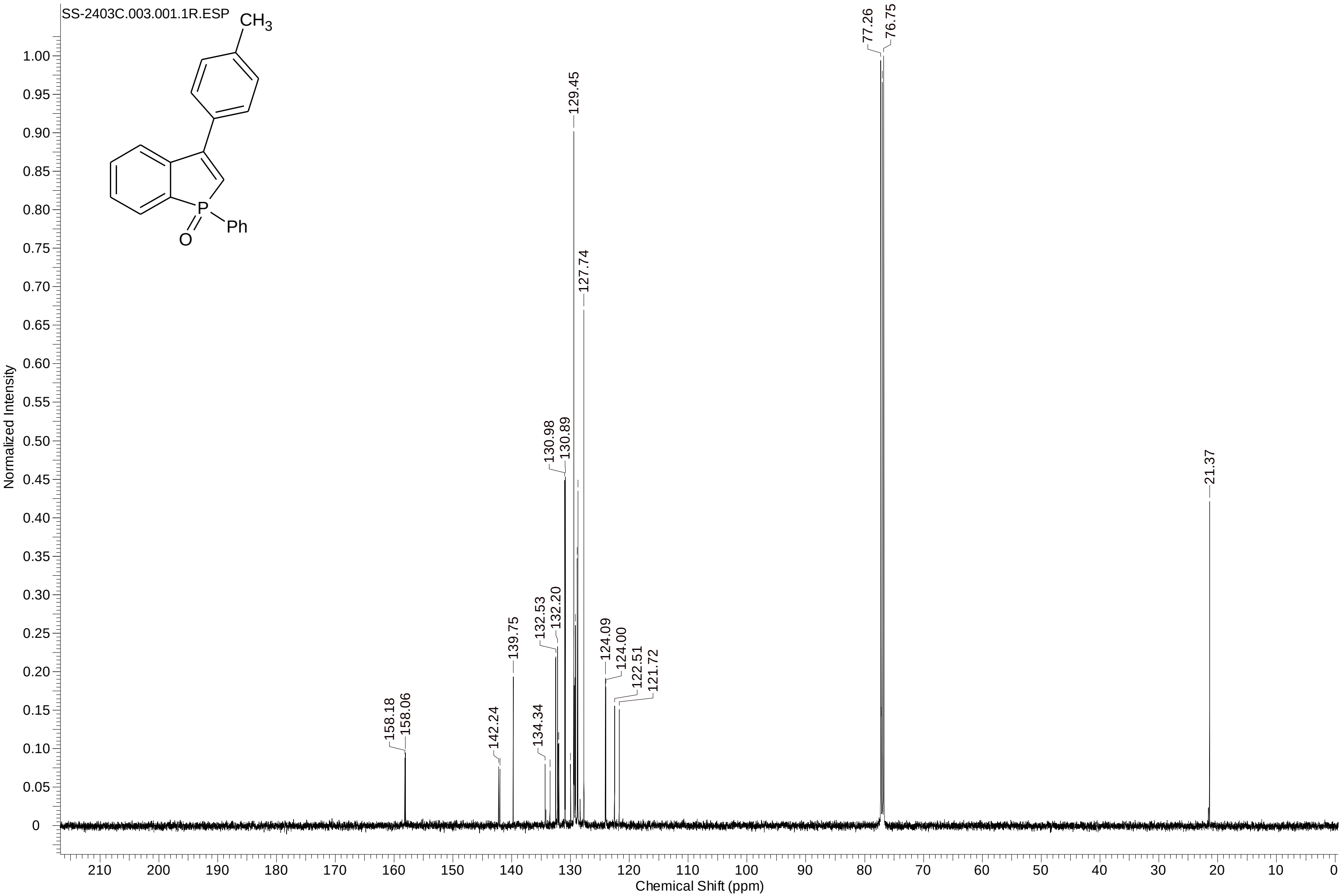

$^{13}C\{^1H\}$ NMR spectrum of 1-phenyl-3-(*p*-tolyl)benzophosphole oxide (**5b**) (125 MHz, $CDCl_3$)

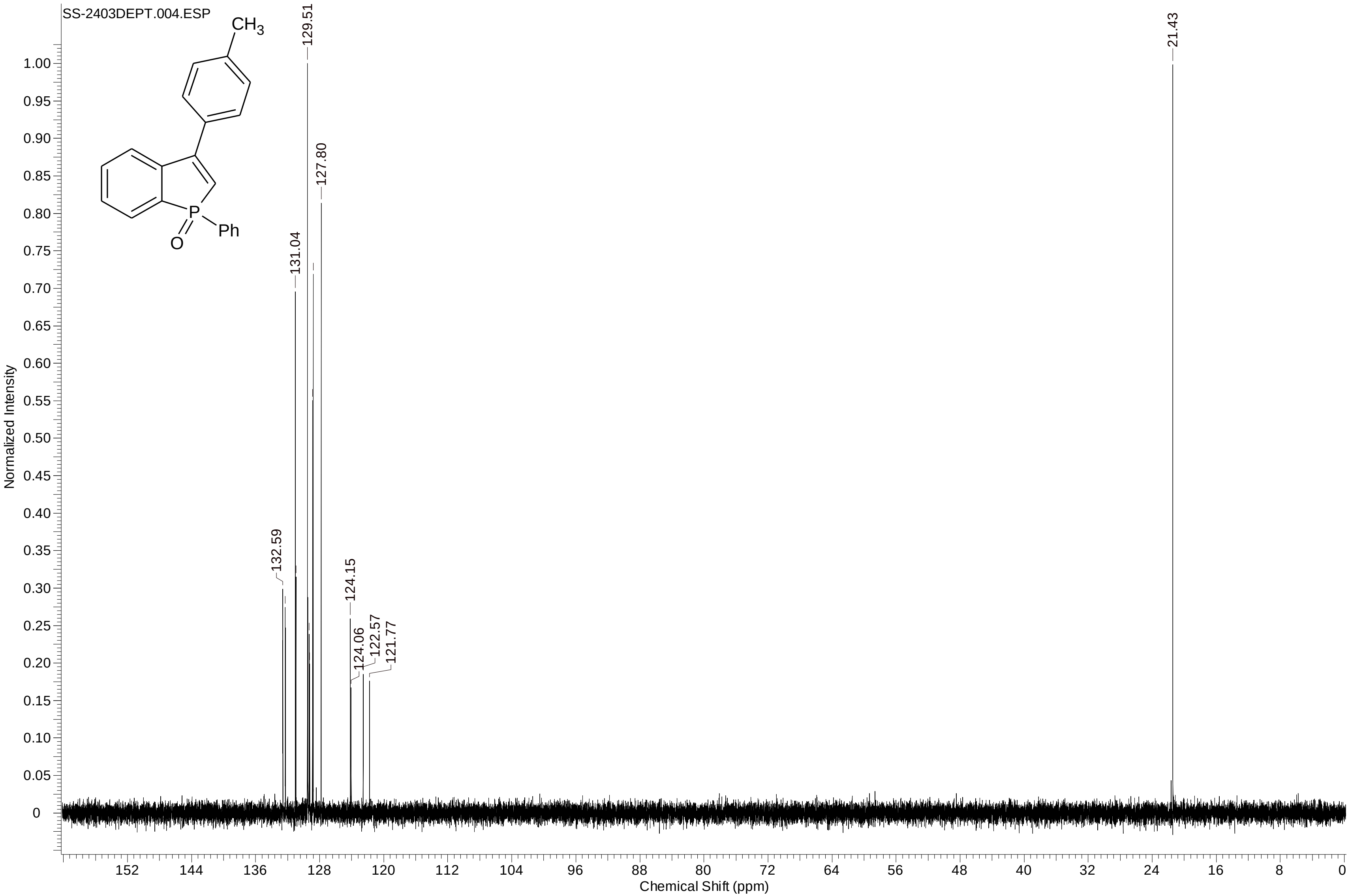

DEPT 135 NMR spectrum of 1-phenyl-3-(*p*-tolyl)benzophosphole oxide (**5b**) (125 MHz, $CDCl_3$)

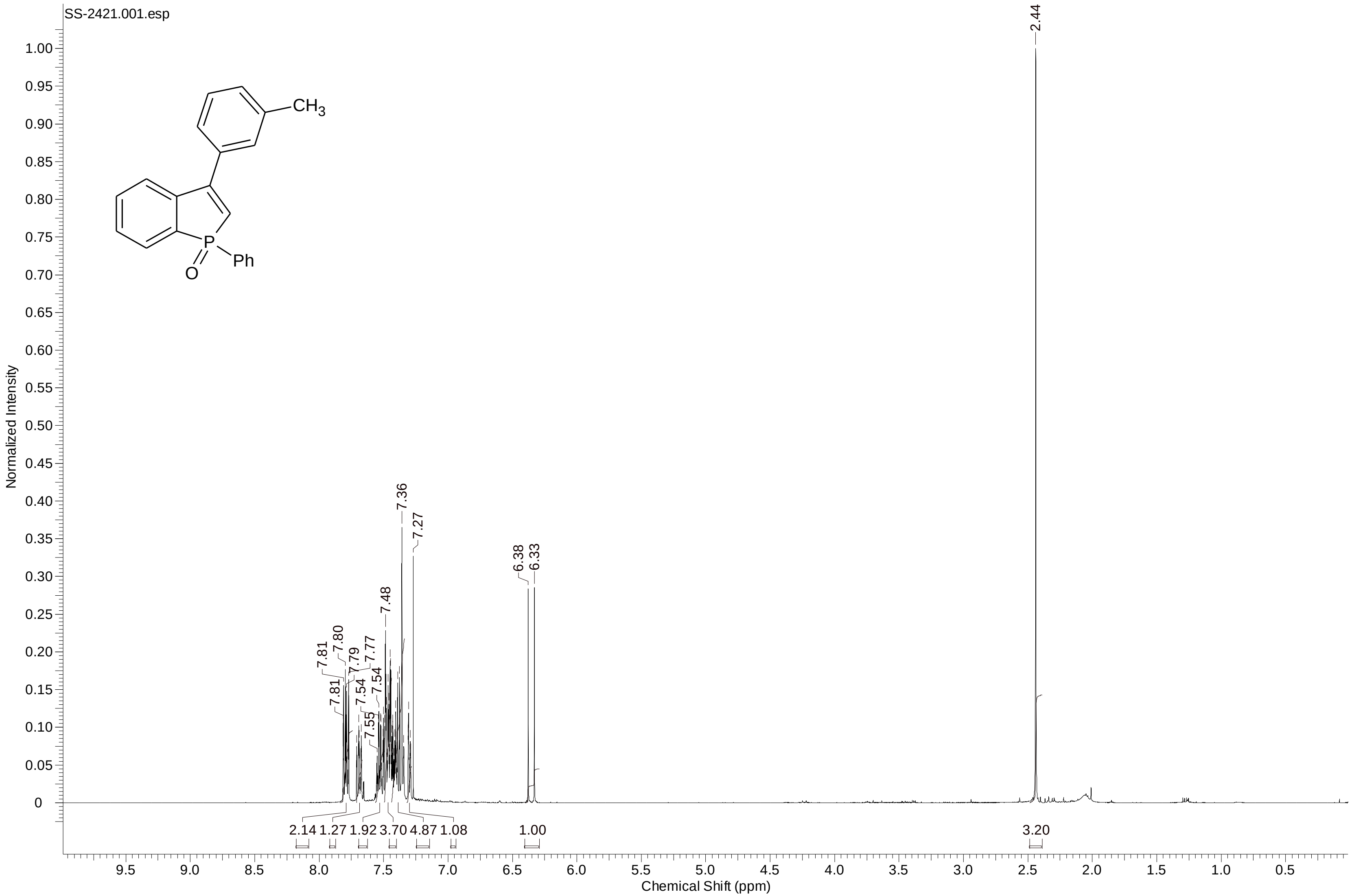

^{1}H NMR spectrum of 1-phenyl-3-(*m*-tolyl)benzophosphole oxide (**5c**) (500 MHz, $CDCl_3$)

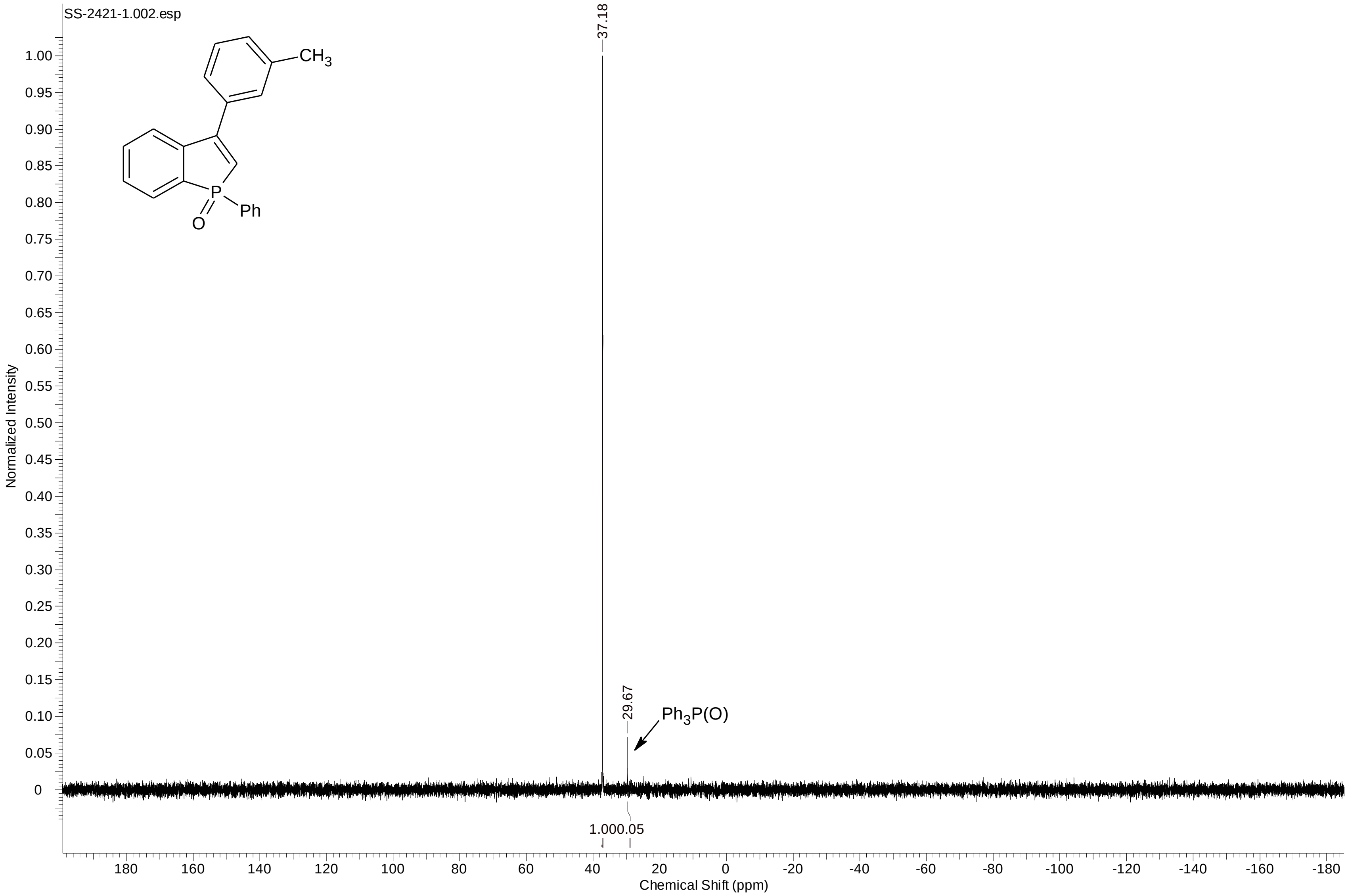

$^{31}P\{^{1}H\}$ NMR spectrum of 1-phenyl-3-(*m*-tolyl)benzophosphole oxide (**5c**) (202 MHz, $CDCl_3$)

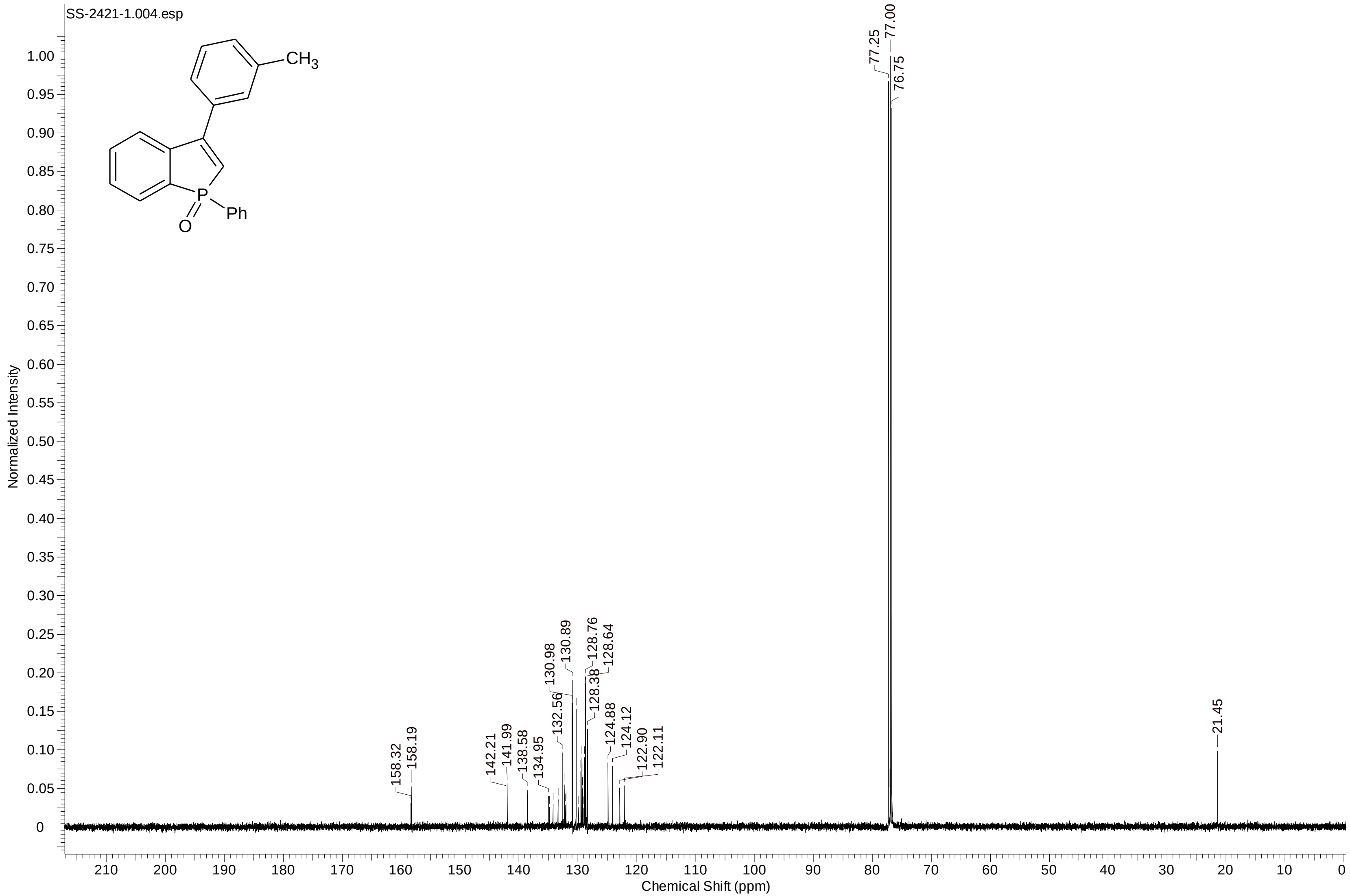

$^{13}C\{^1H\}$ NMR spectrum of 1-phenyl-3-(*m*-tolyl)benzophosphole oxide (**5c**) (125 MHz, $CDCl_3$)

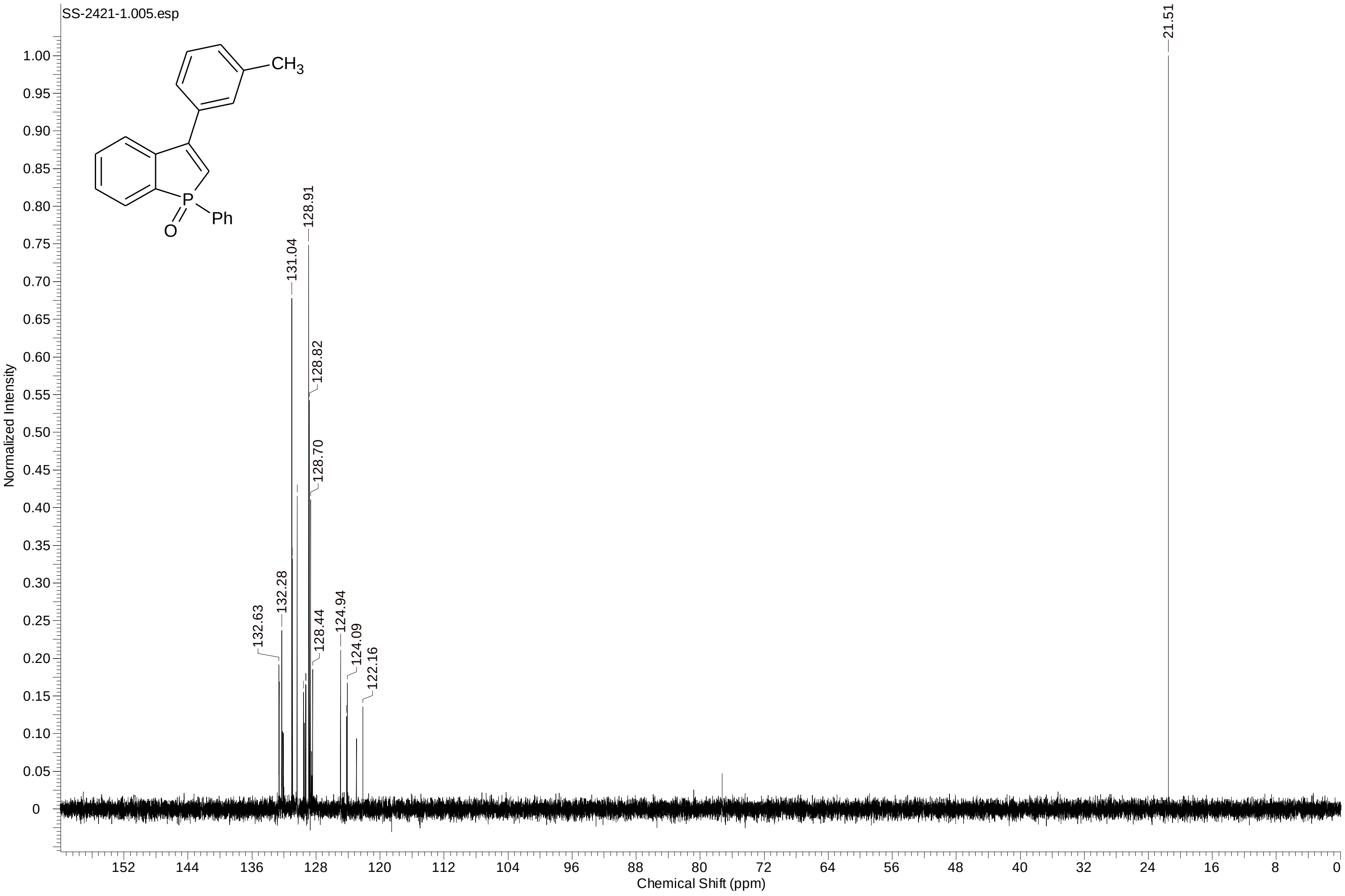

DEPT 135 NMR spectrum of 1-phenyl-3-(*m*-tolyl)benzophosphole oxide (**5c**) (125 MHz, $CDCl_3$)

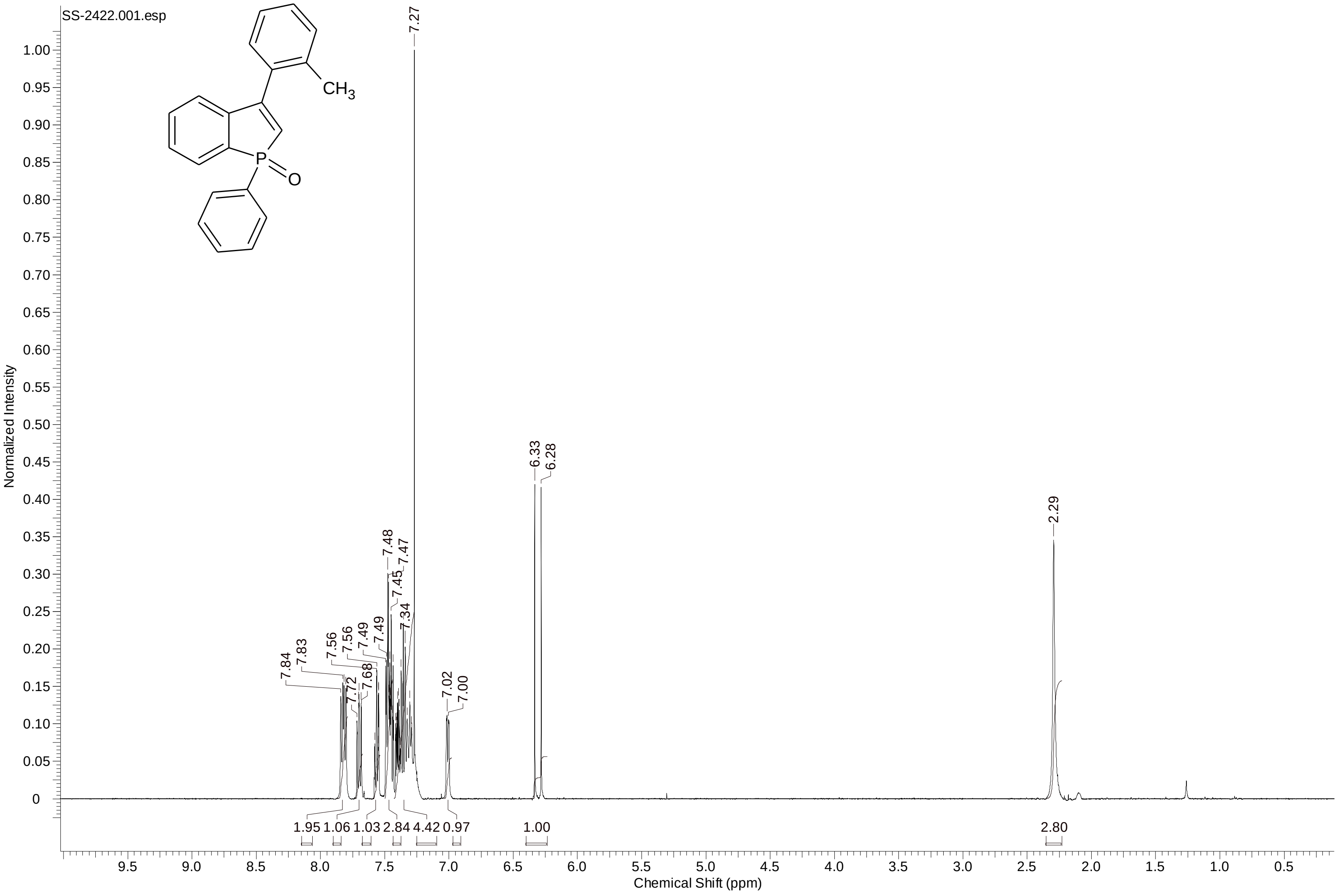

^{1}H NMR spectrum of 1-phenyl-3-(*o*-tolyl)benzophosphole oxide (**5d**) (500 MHz, $CDCl_3$)

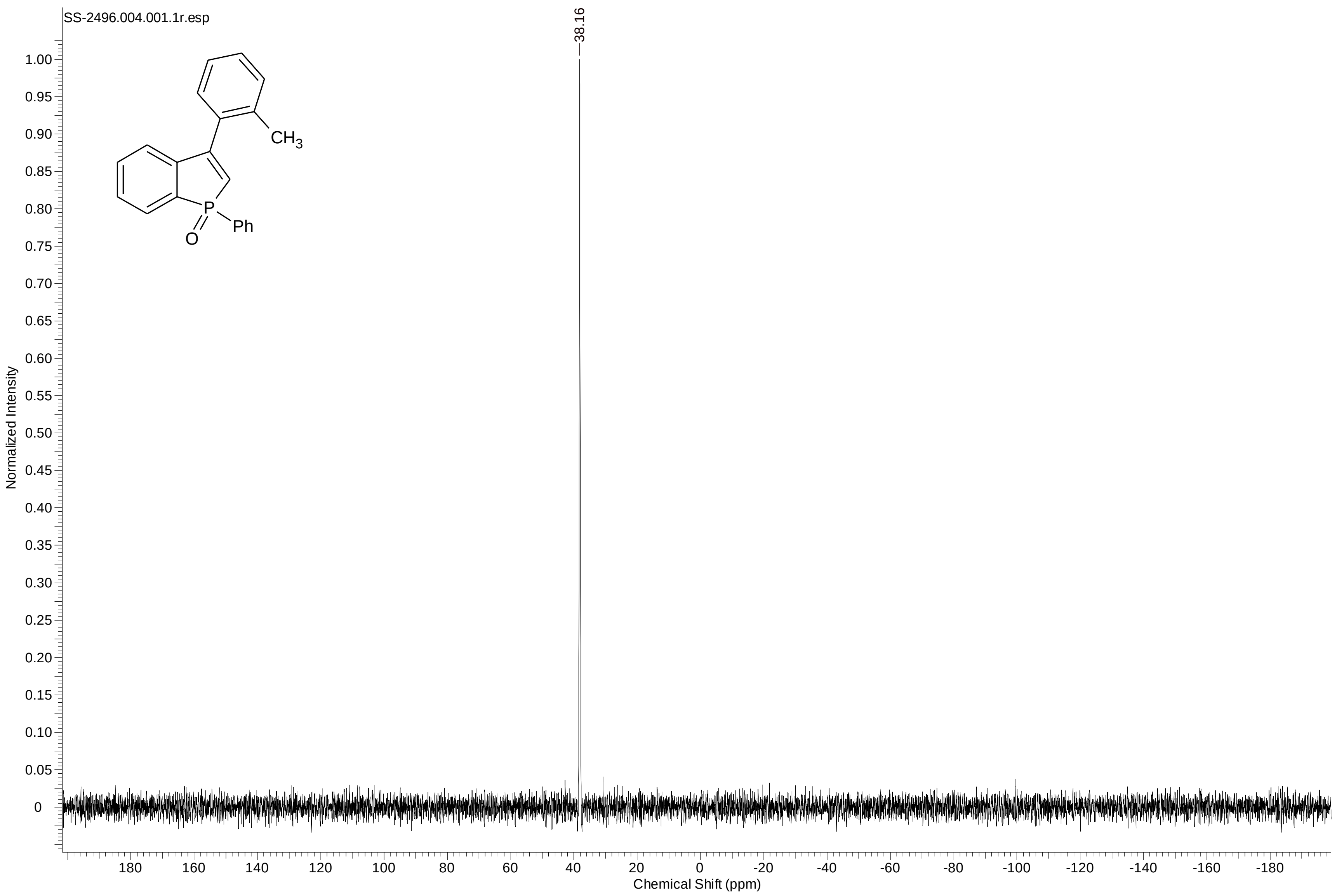

$^{31}P\{^{1}H\}$ NMR spectrum of 1-phenyl-3-(*o*-tolyl)benzophosphole oxide (**5d**) (202 MHz, $CDCl_3$)

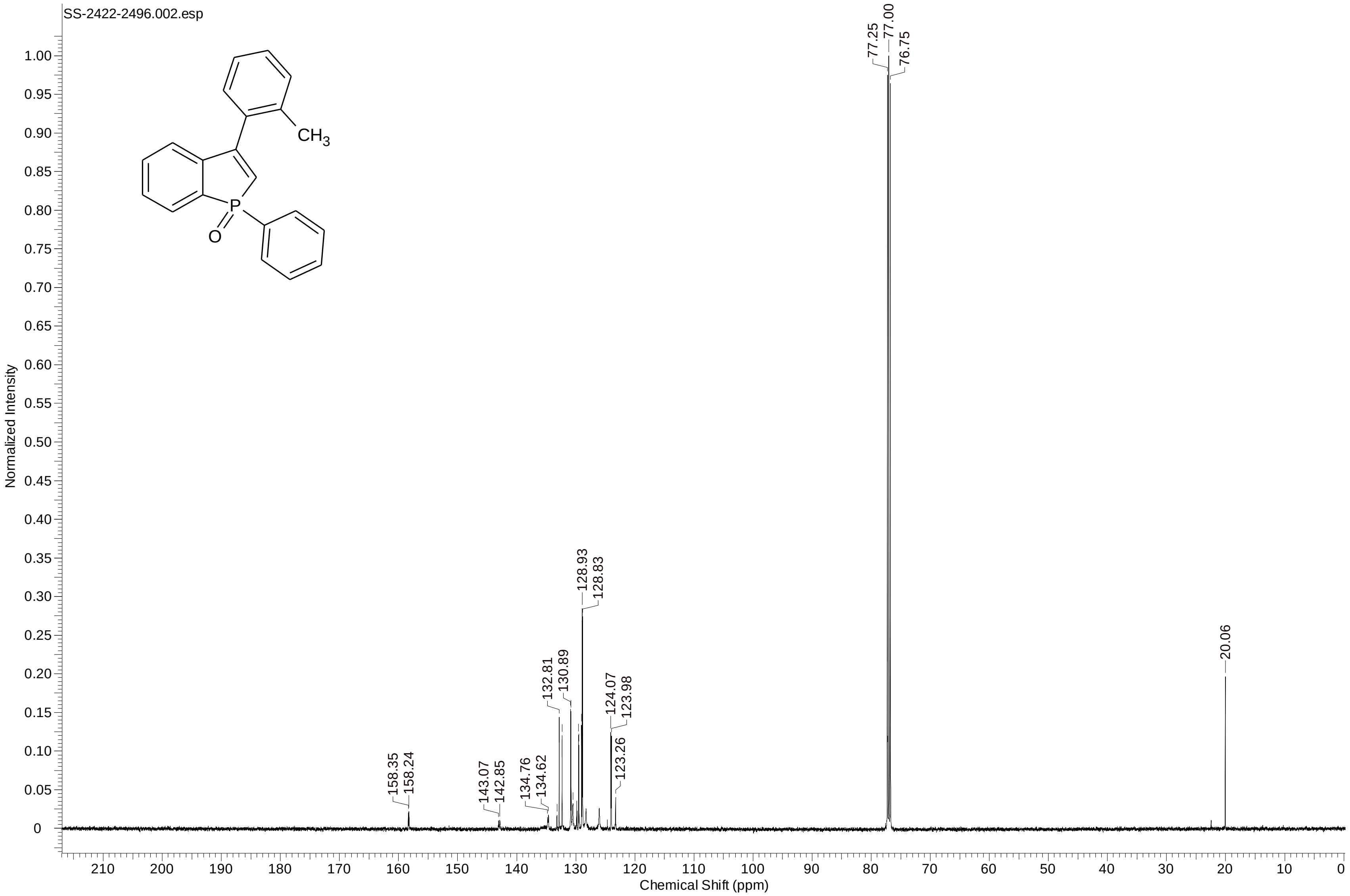

$^{13}C\{^{1}H\}$ NMR spectrum of 1-phenyl-3-(*o*-tolyl)benzophosphole oxide (**5d**) (125 MHz, $CDCl_3$)

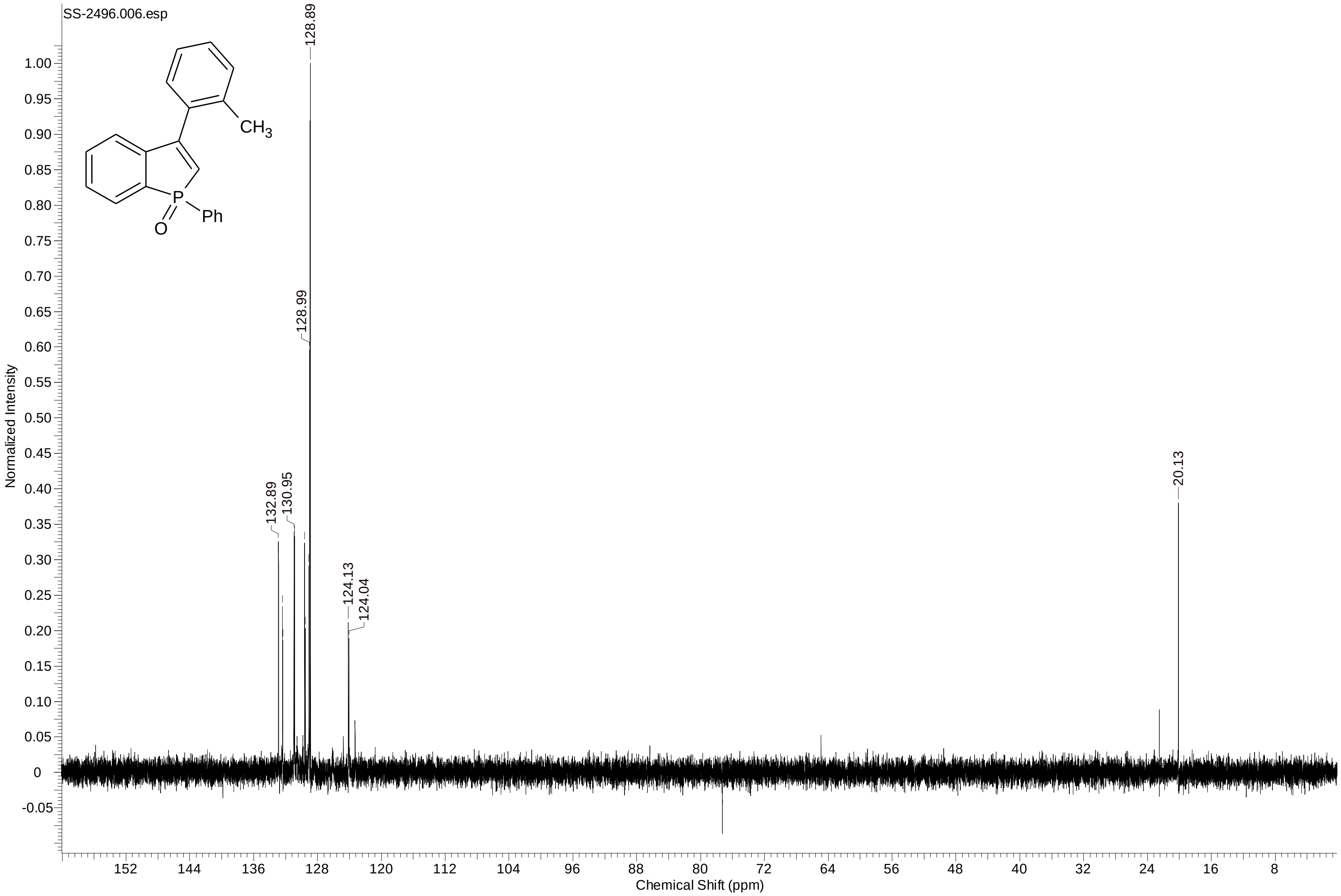

DEPT 135 NMR spectrum of 1-phenyl-3-(*o*-tolyl)benzophosphole oxide (**5d**) (125 MHz, $CDCl_3$)

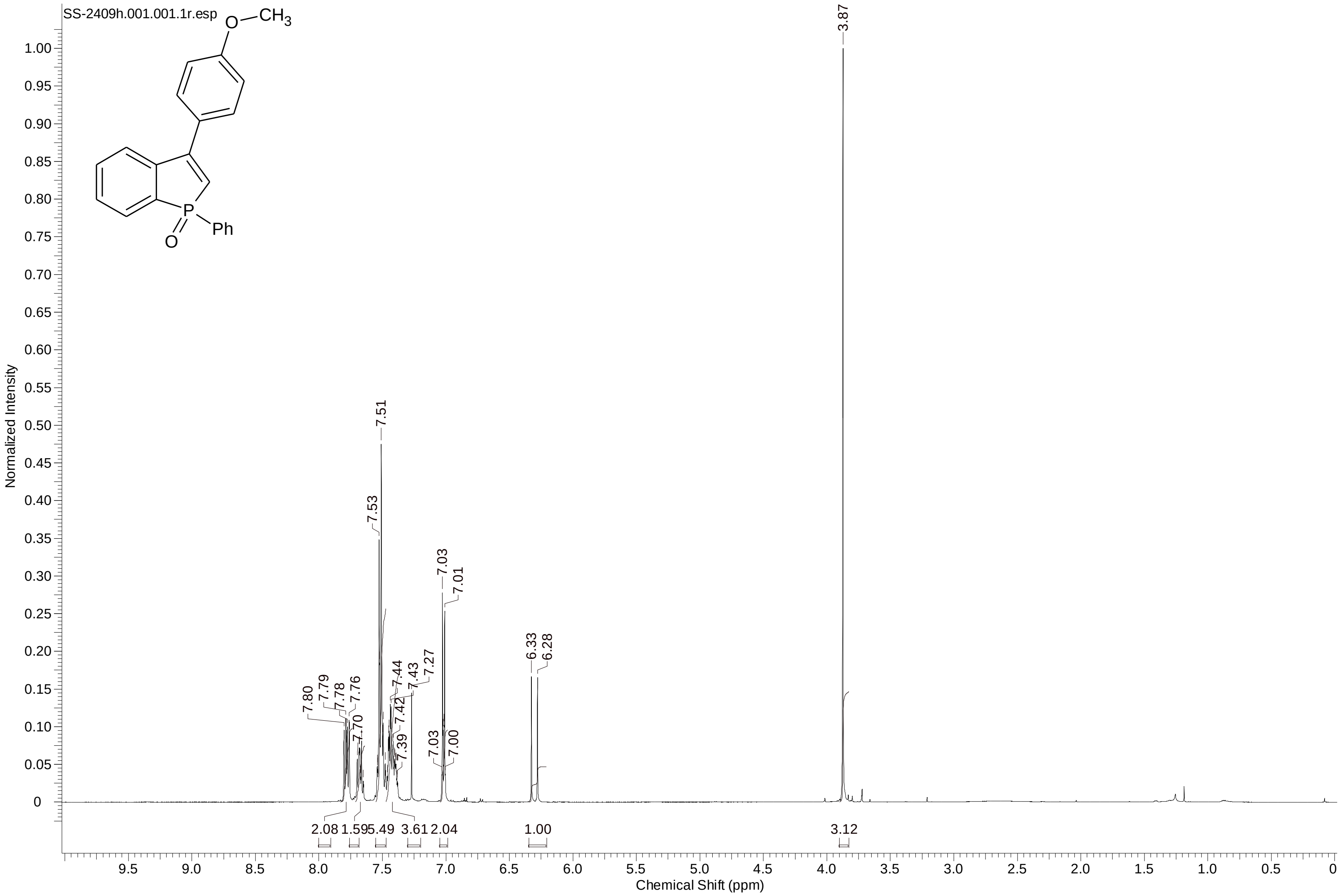

^{1}H NMR spectrum of 1-phenyl-3-(*p*-anisyl)benzophosphole oxide (**5e**) (500 MHz, $CDCl_3$)

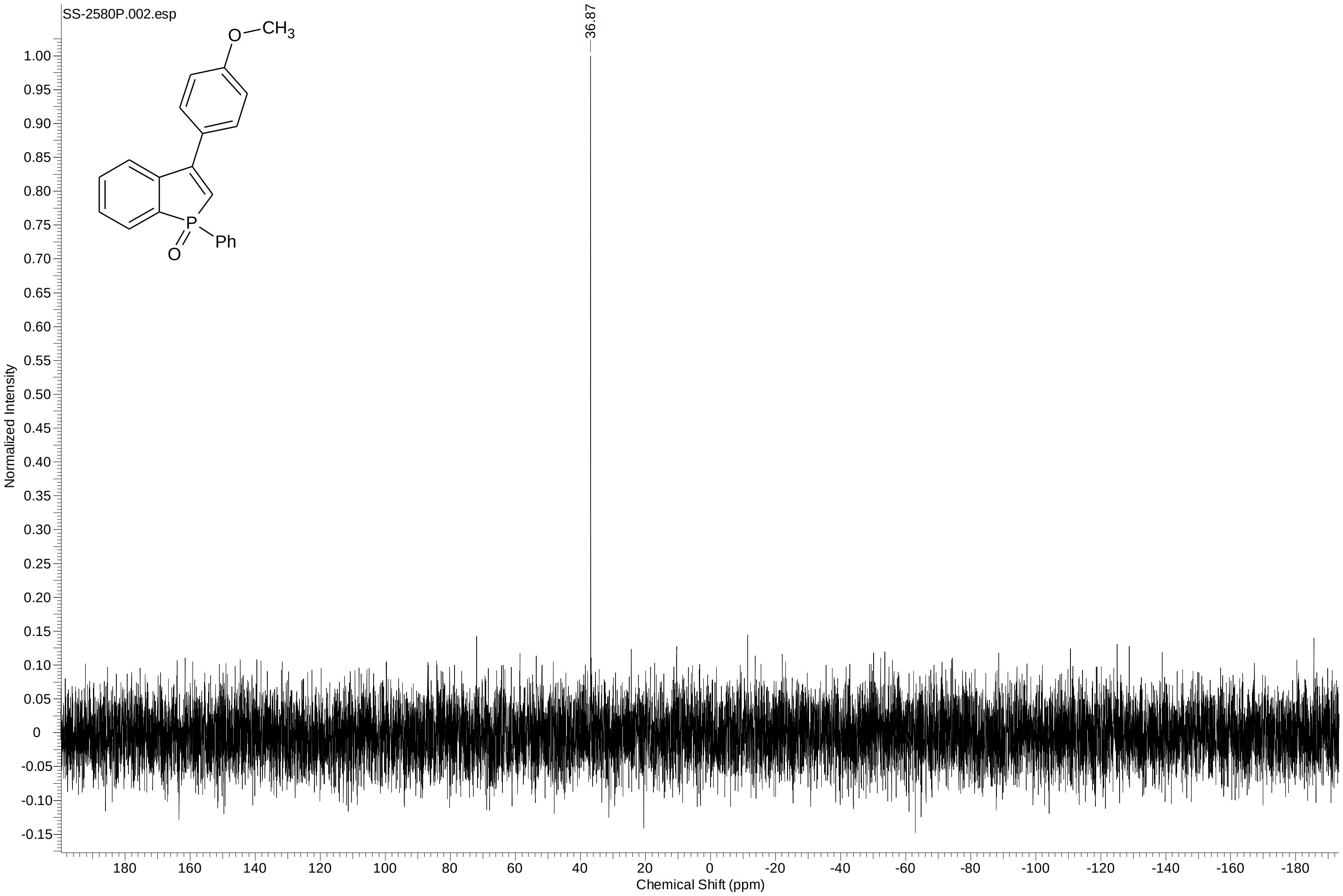

$^{31}P\{^{1}H\}$ NMR spectrum of 1-phenyl-3-(*p*-anisyl)benzophosphole oxide (**5e**) (202 MHz, $CDCl_3$)

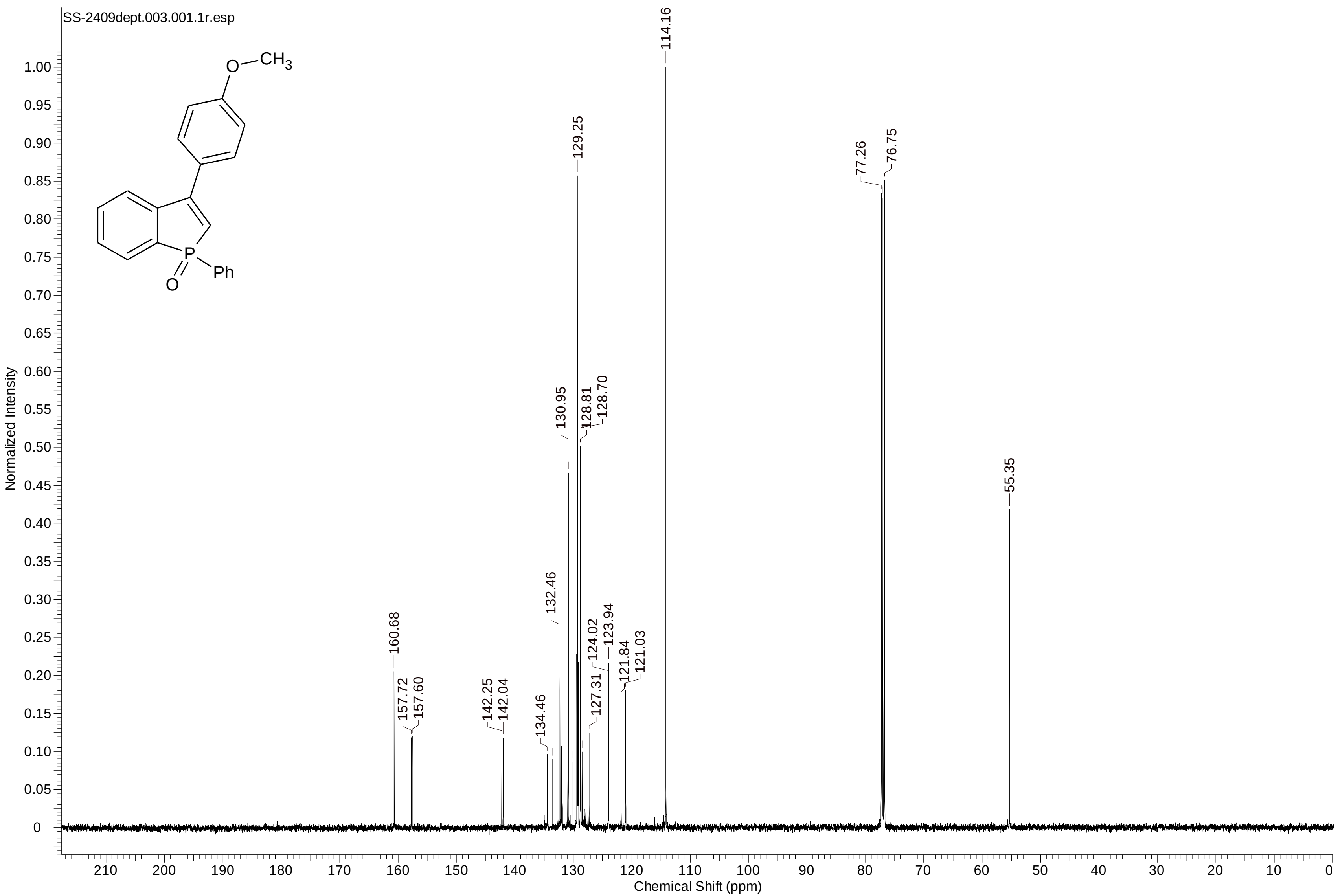

$^{13}C\{^{1}H\}$ NMR spectrum of 1-phenyl-3-(*p*-anisyl)benzophosphole oxide (**5e**) (125 MHz, $CDCl_3$)

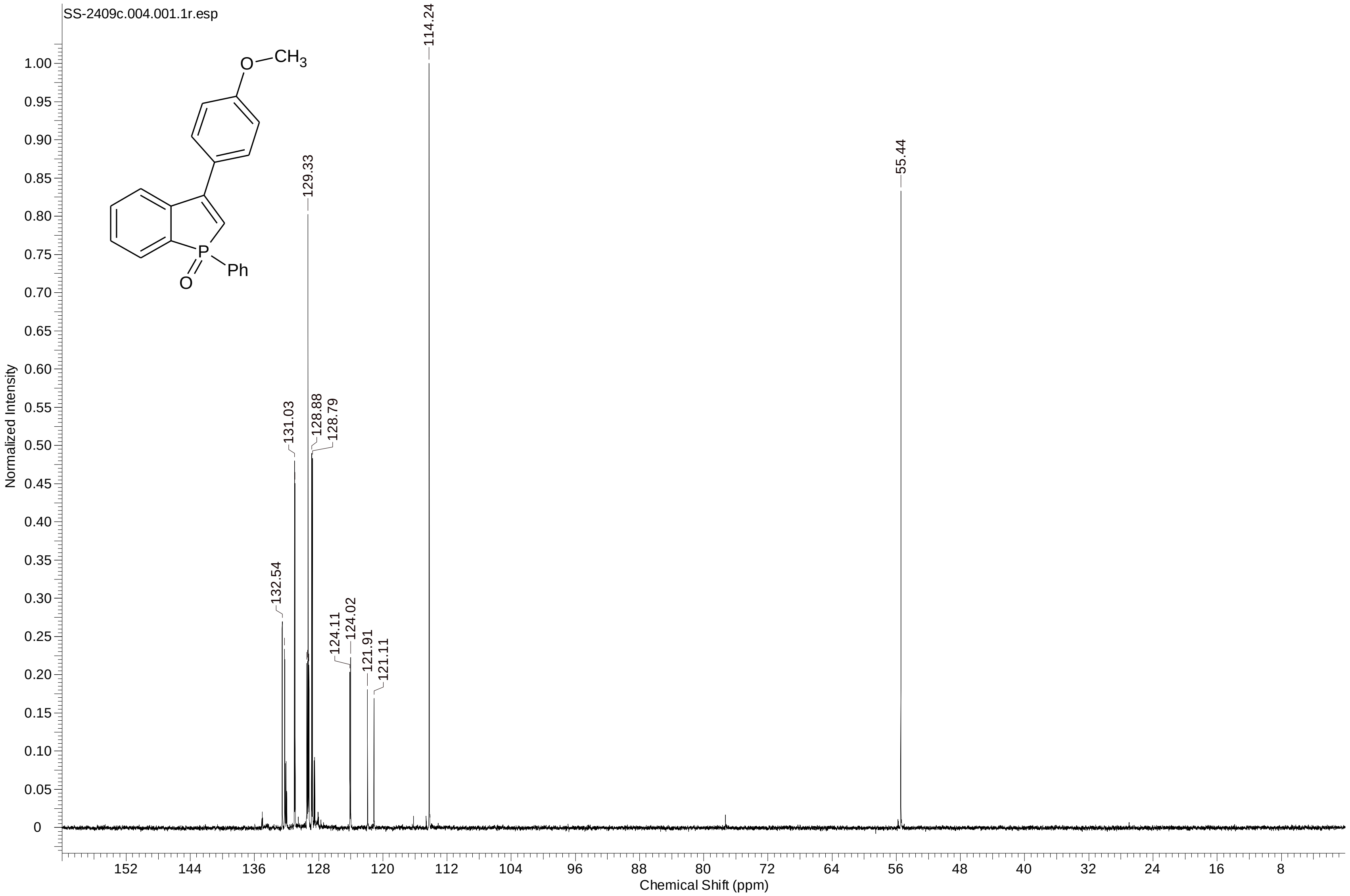

DEPT 135 NMR spectrum of 1-phenyl-3-(*p*-anisyl)benzophosphole oxide (**5e**) (125 MHz, $CDCl_3$)

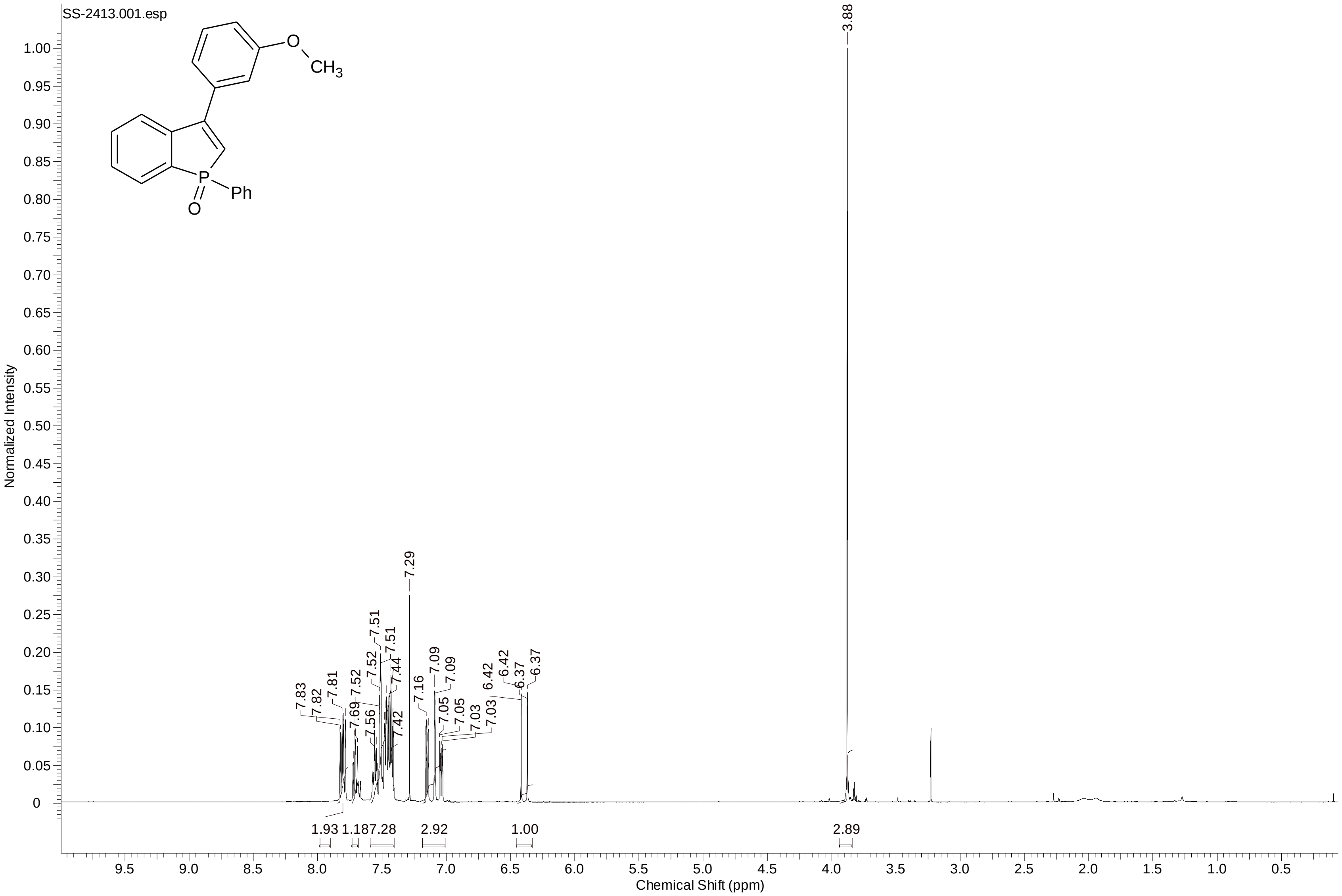

^{1}H NMR spectrum of 1-phenyl-3-(*m*-anisyl)benzophosphole oxide (**5f**) (500 MHz, $CDCl_3$)

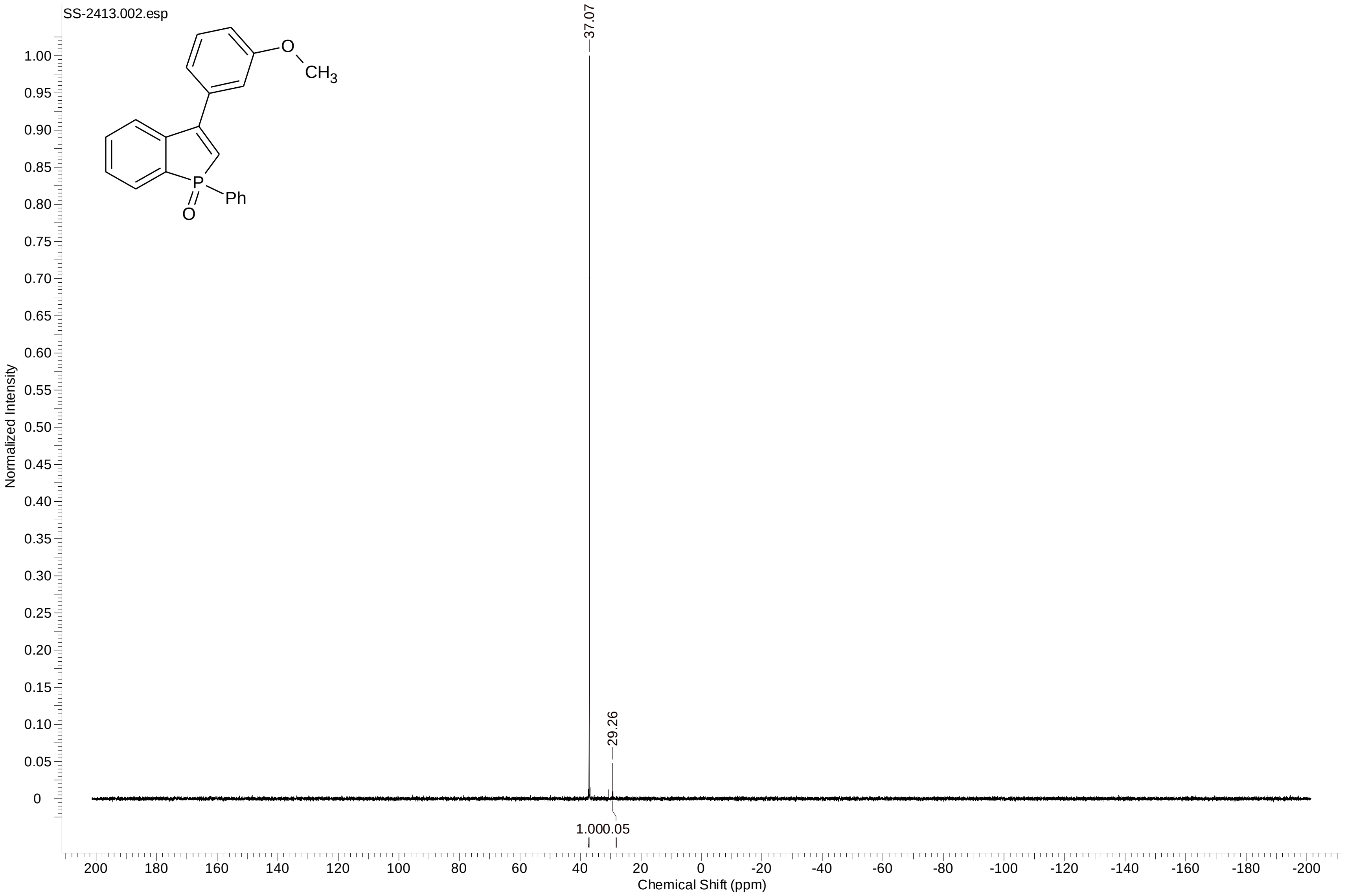

$^{31}P\{^1H\}$ NMR spectrum of 1-phenyl-3-(*m*-anisyl)benzophosphole oxide (**5f**) (202 MHz, $CDCl_3$)

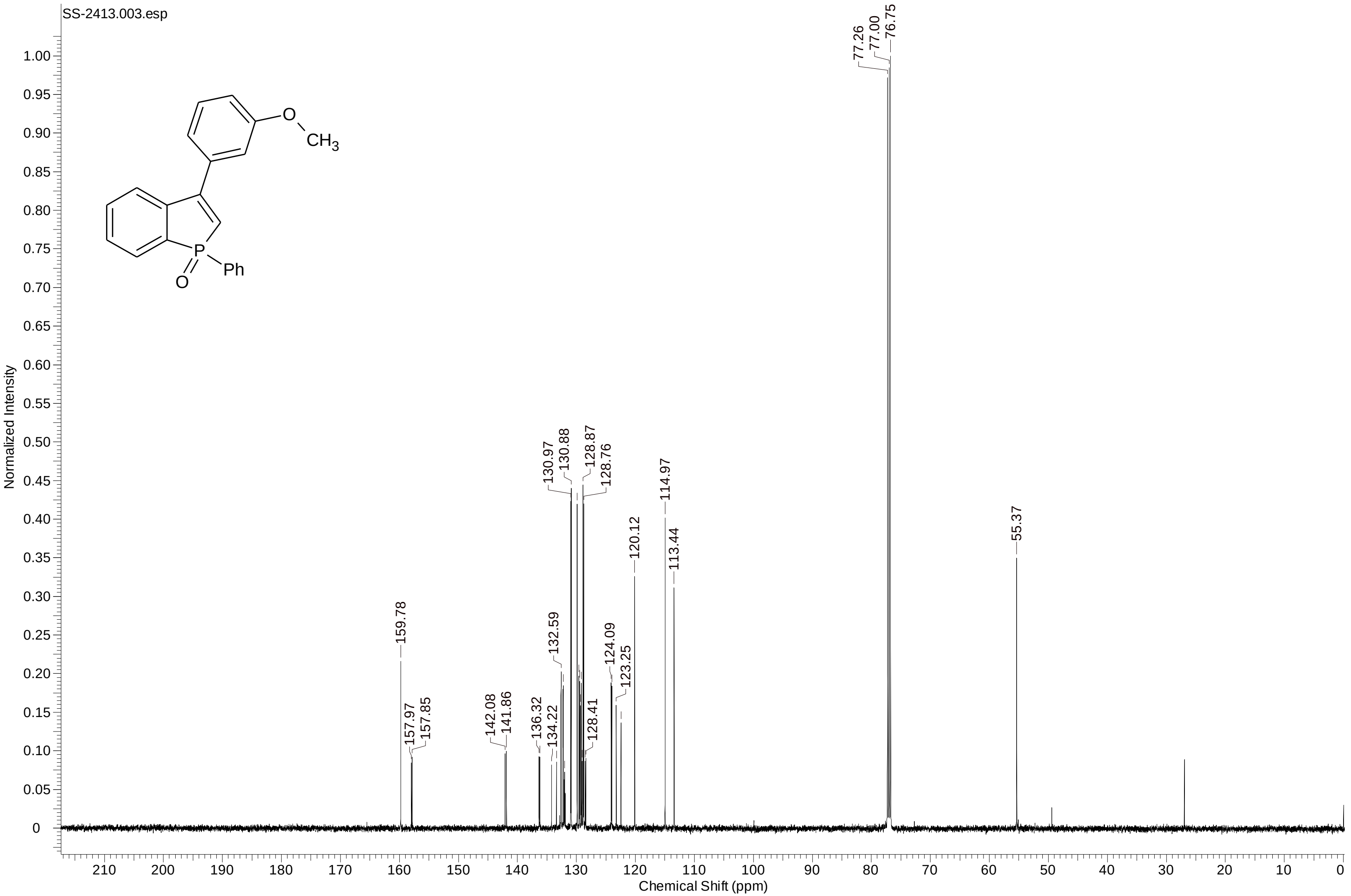

$^{13}C\{^1H\}$ NMR spectrum of 1-phenyl-3-(*m*-anisyl)benzophosphole oxide (**5f**) (125 MHz, $CDCl_3$)

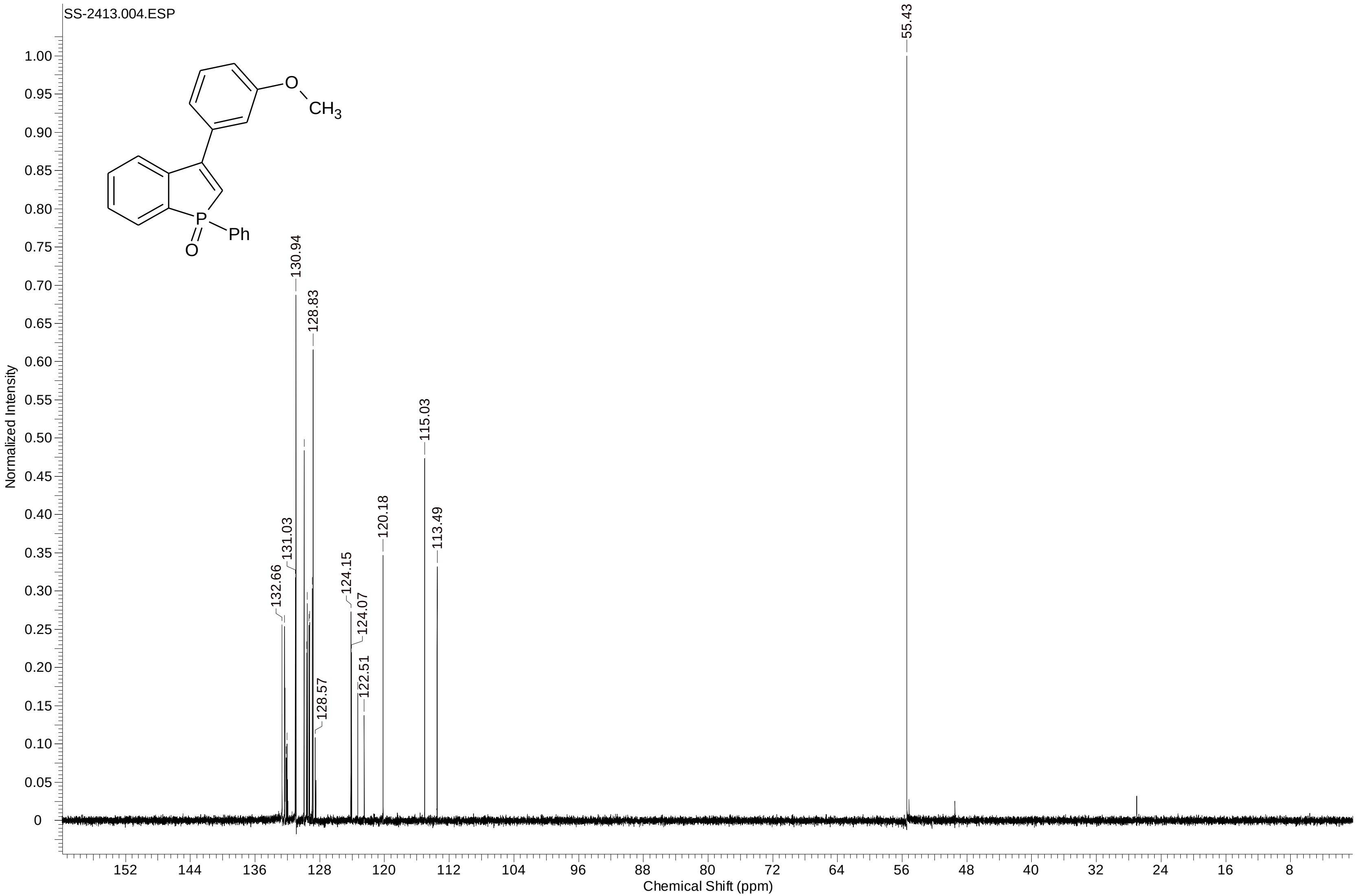

DEPT 135 NMR spectrum of 1-phenyl-3-(*m*-anisyl)benzophosphole oxide (**5f**) (125 MHz, $CDCl_3$)

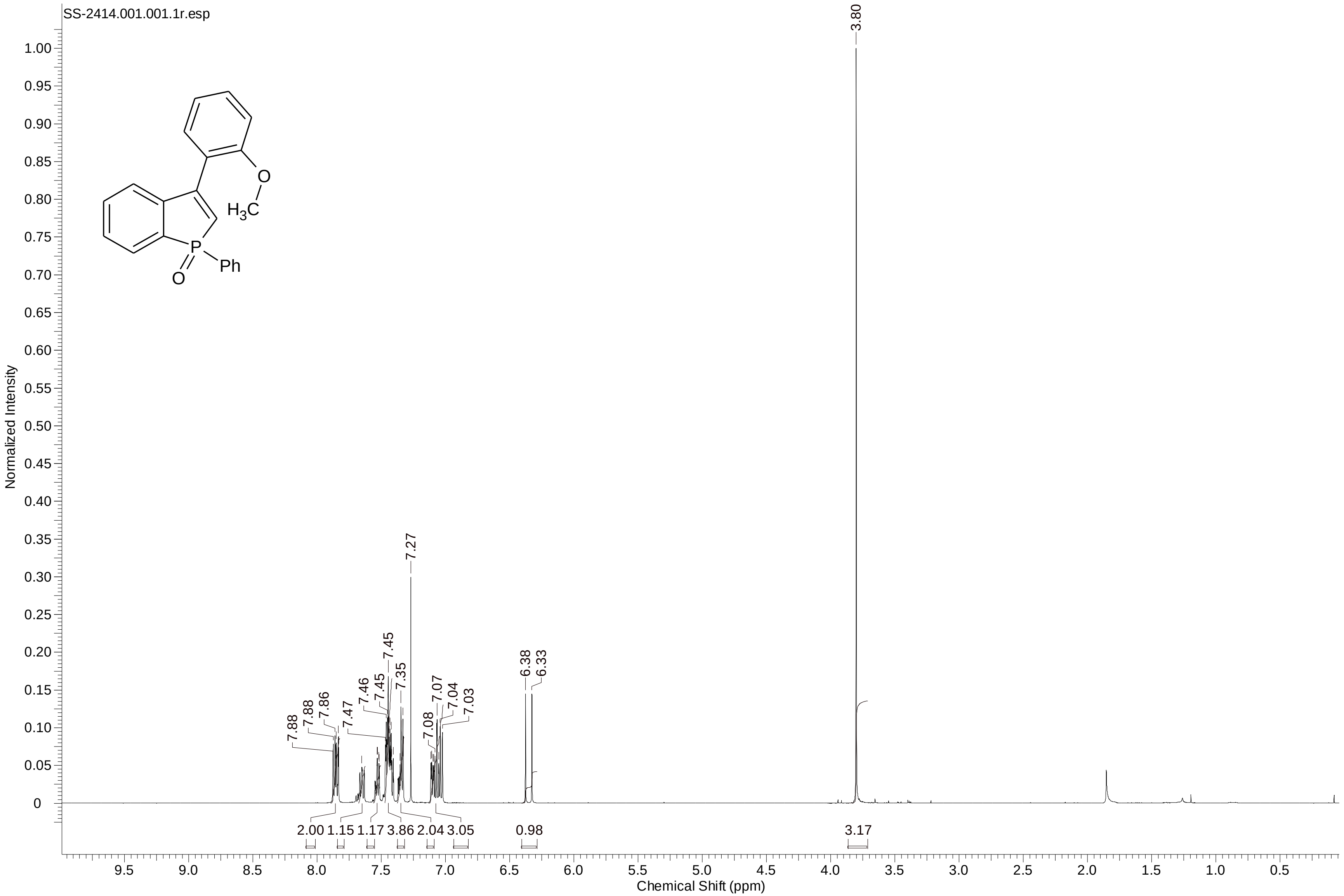

^{1}H NMR spectrum of 1-phenyl-3-(*o*-anisyl)benzophosphole oxide (**5g**) (500 MHz, $CDCl_3$)

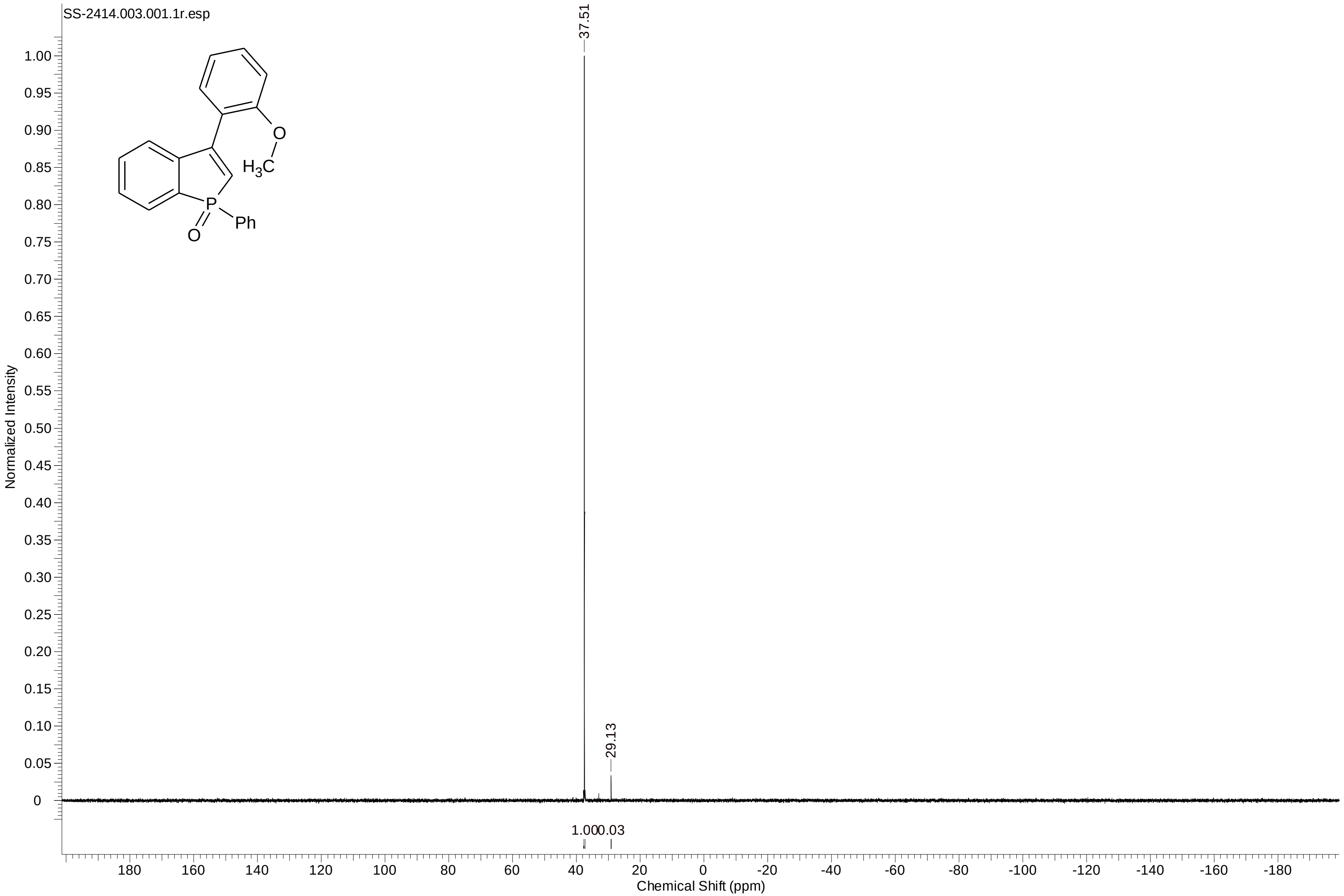

$^{31}P\{^{1}H\}$ NMR spectrum of 1-phenyl-3-(*o*-anisyl)benzophosphole oxide (**5g**) (202 MHz, $CDCl_3$)

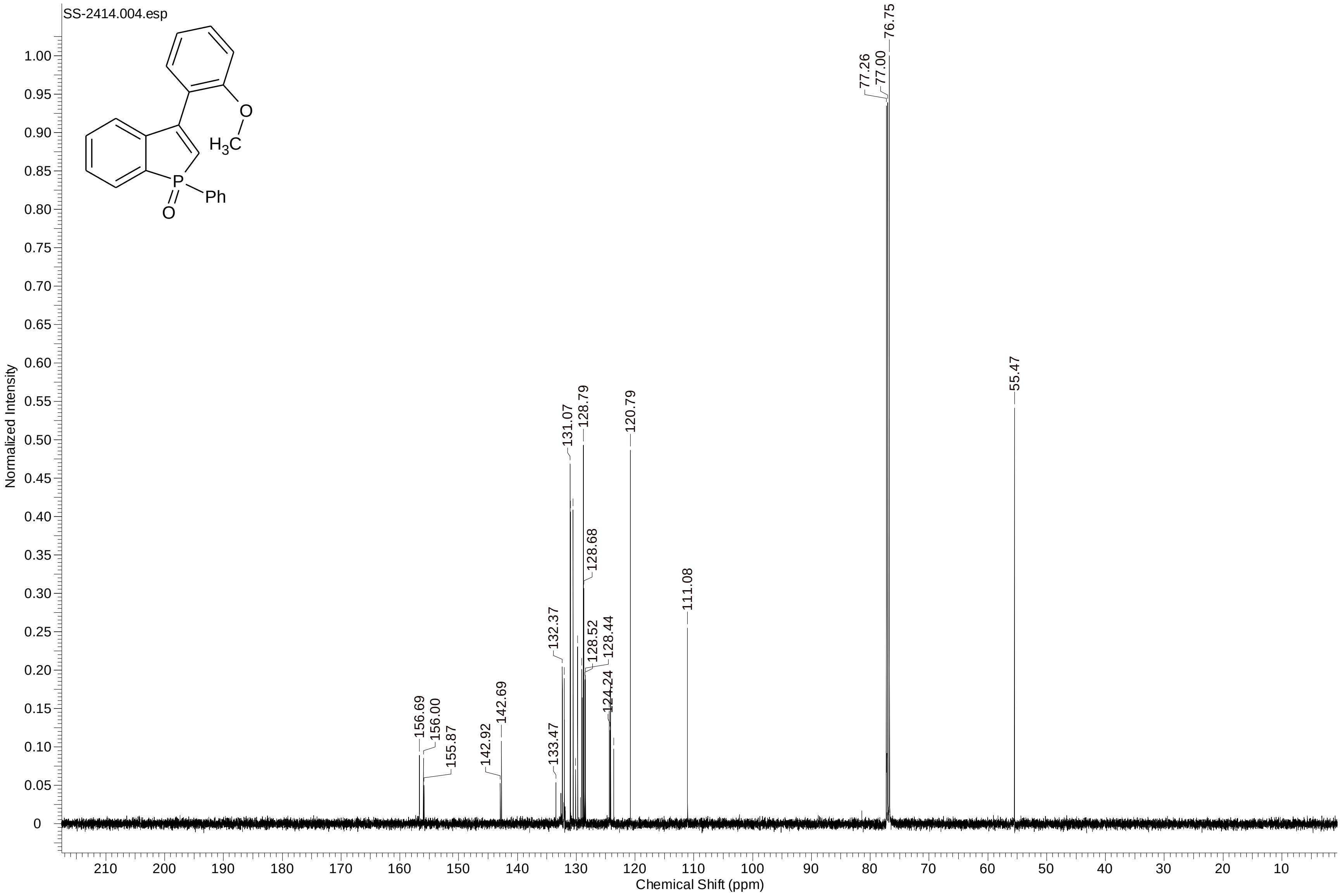

$^{13}C\{^{1}H\}$ NMR spectrum of 1-phenyl-3-(*o*-anisyl)benzophosphole oxide (**5g**) (125 MHz, $CDCl_3$)

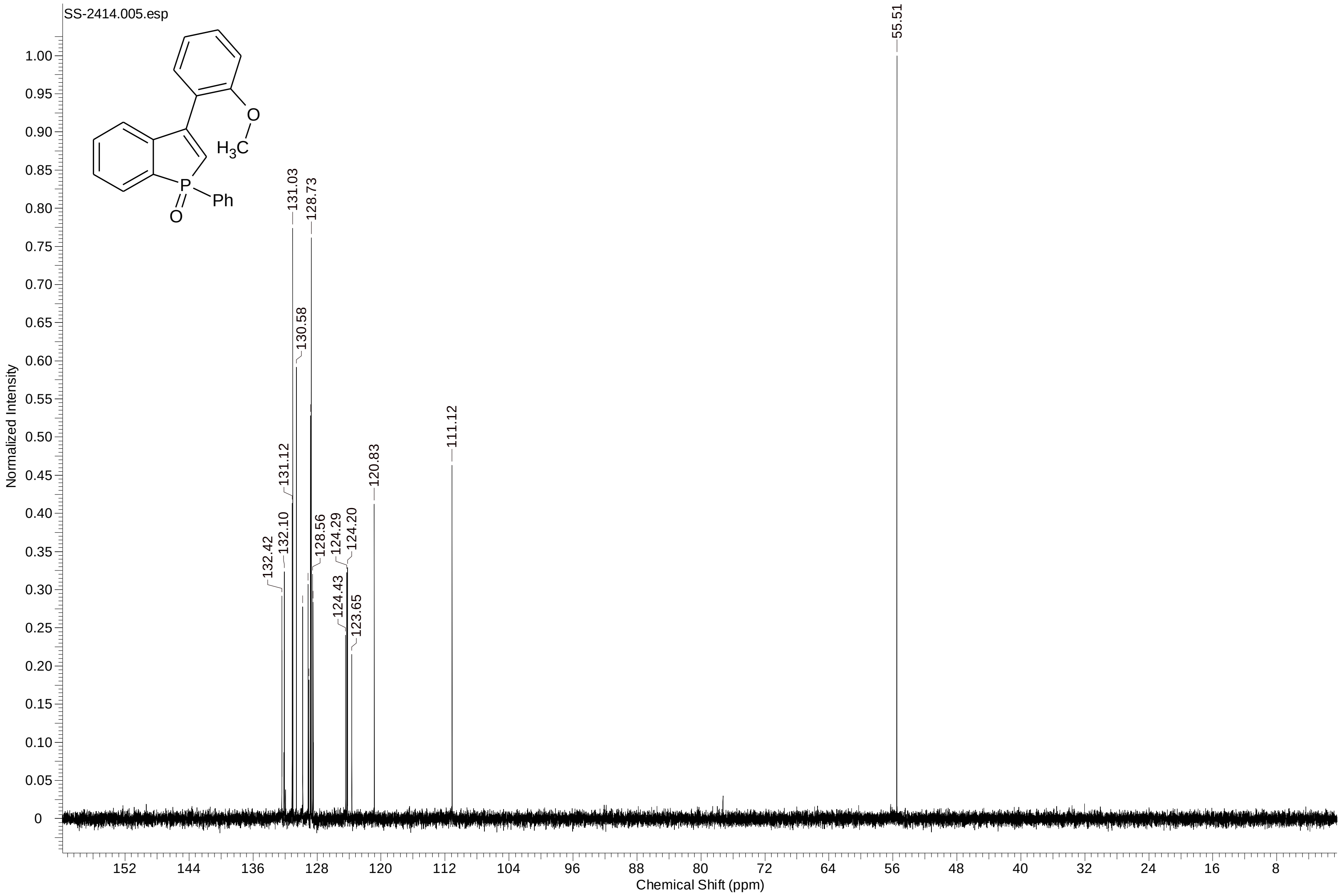

DEPT 135 NMR spectrum of 1-phenyl-3-(*o*-anisyl)benzophosphole oxide (**5g**) (125 MHz, $CDCl_3$)

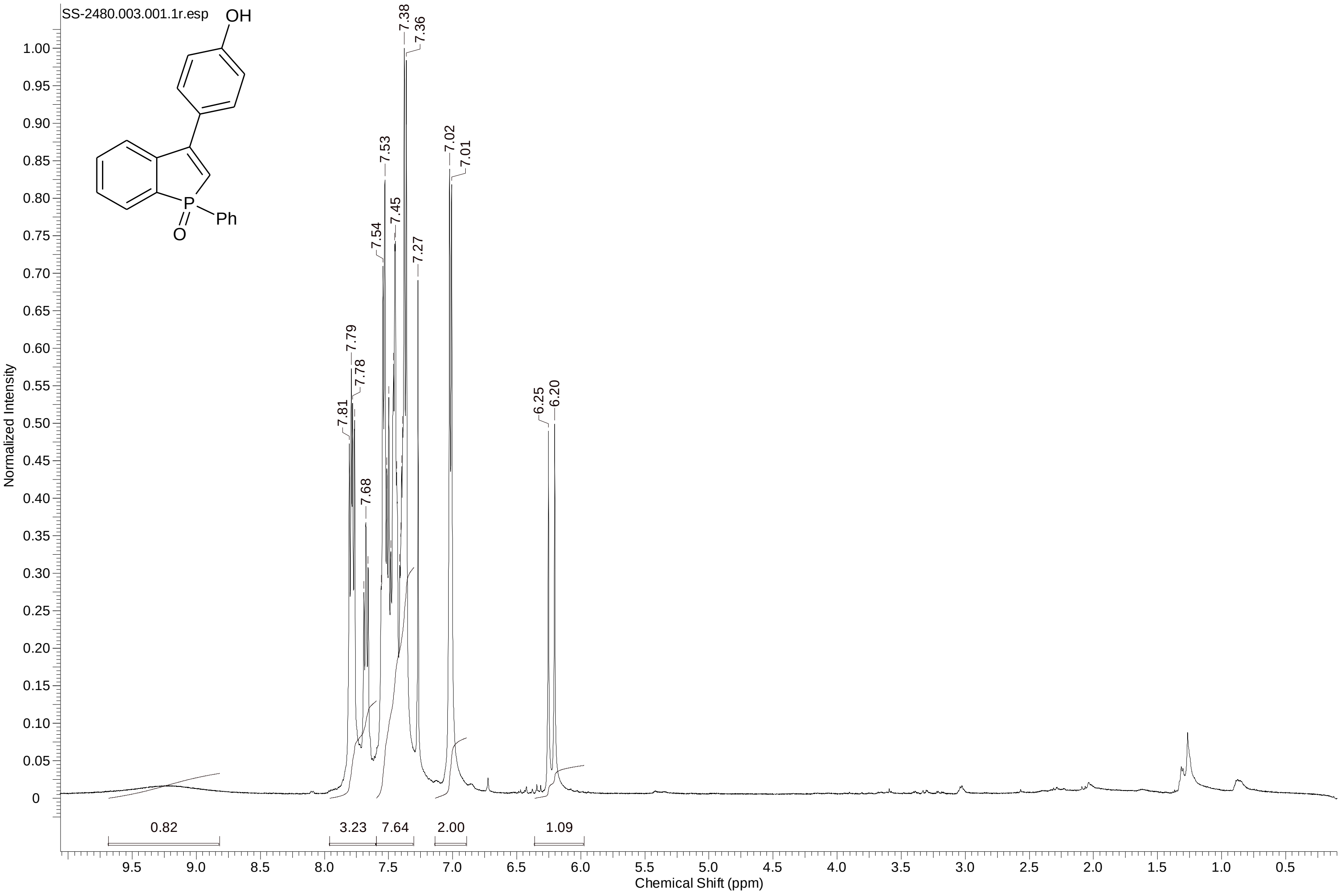

^{1}H NMR spectrum of 1-phenyl-3-(*p*-hydroxyphenyl)benzophosphole oxide (**5h**) (500 MHz, $CDCl_3$)

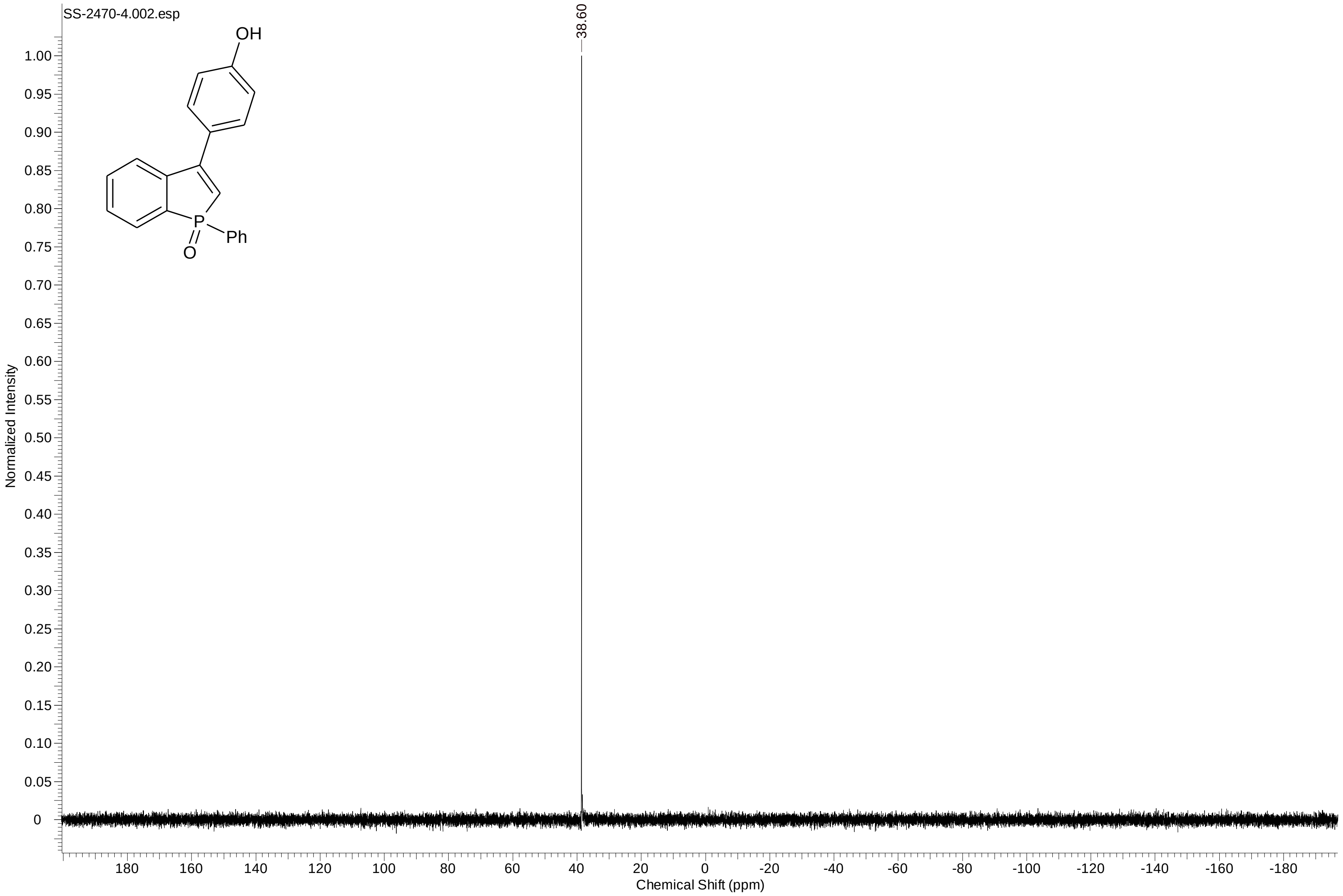

$^{31}P\{^1H\}$ NMR spectrum of 1-phenyl-3-(*p*-hydroxyphenyl)benzophosphole oxide (**5h**) (202 MHz, $CDCl_3$)

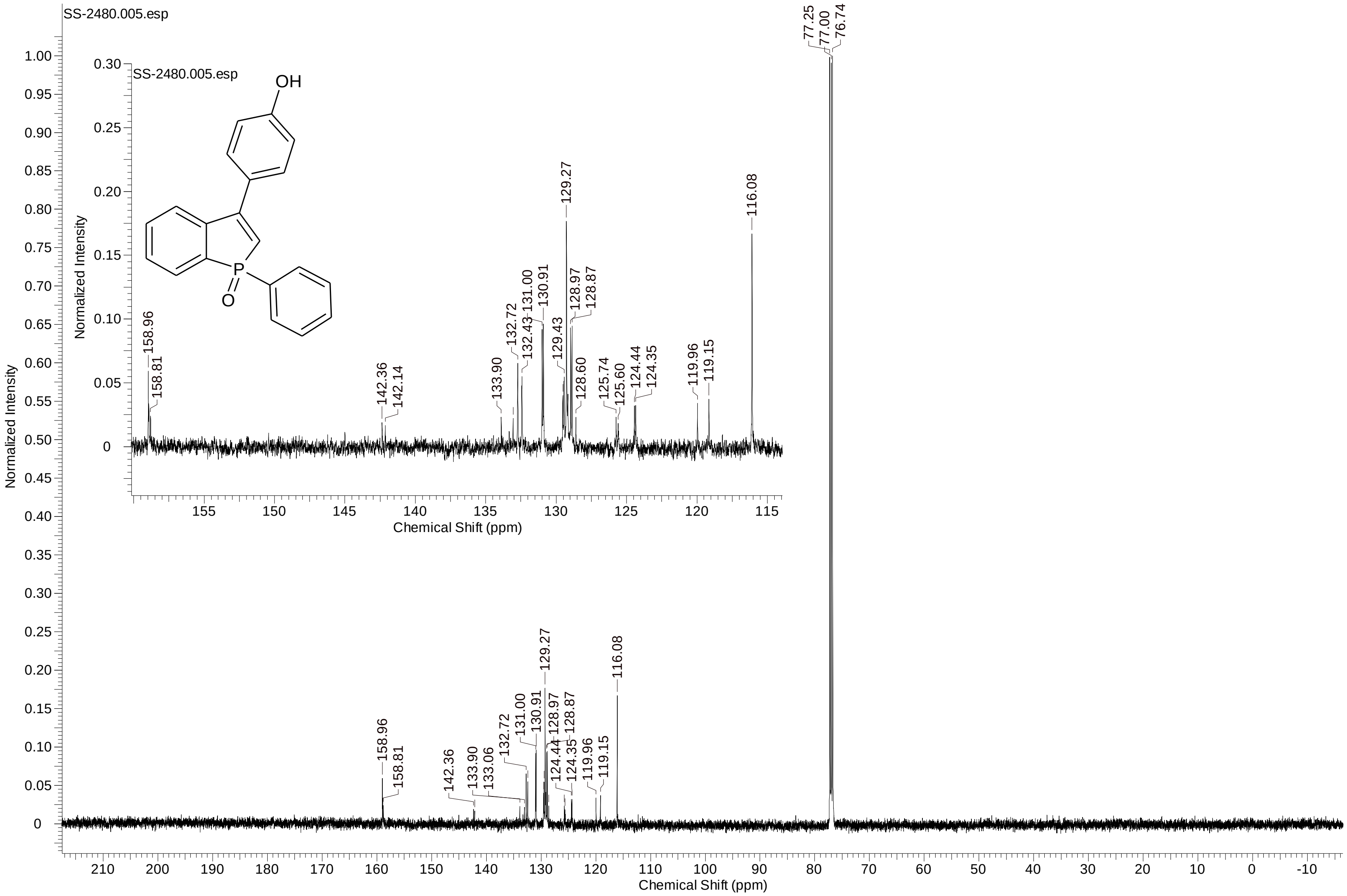

$^{13}C\{^1H\}$ NMR spectrum of 1-phenyl-3-(*p*-hydroxyphenyl)benzophosphole oxide (**5h**) (125 MHz, $CDCl_3$) (low solubility in $CDCl_3$)

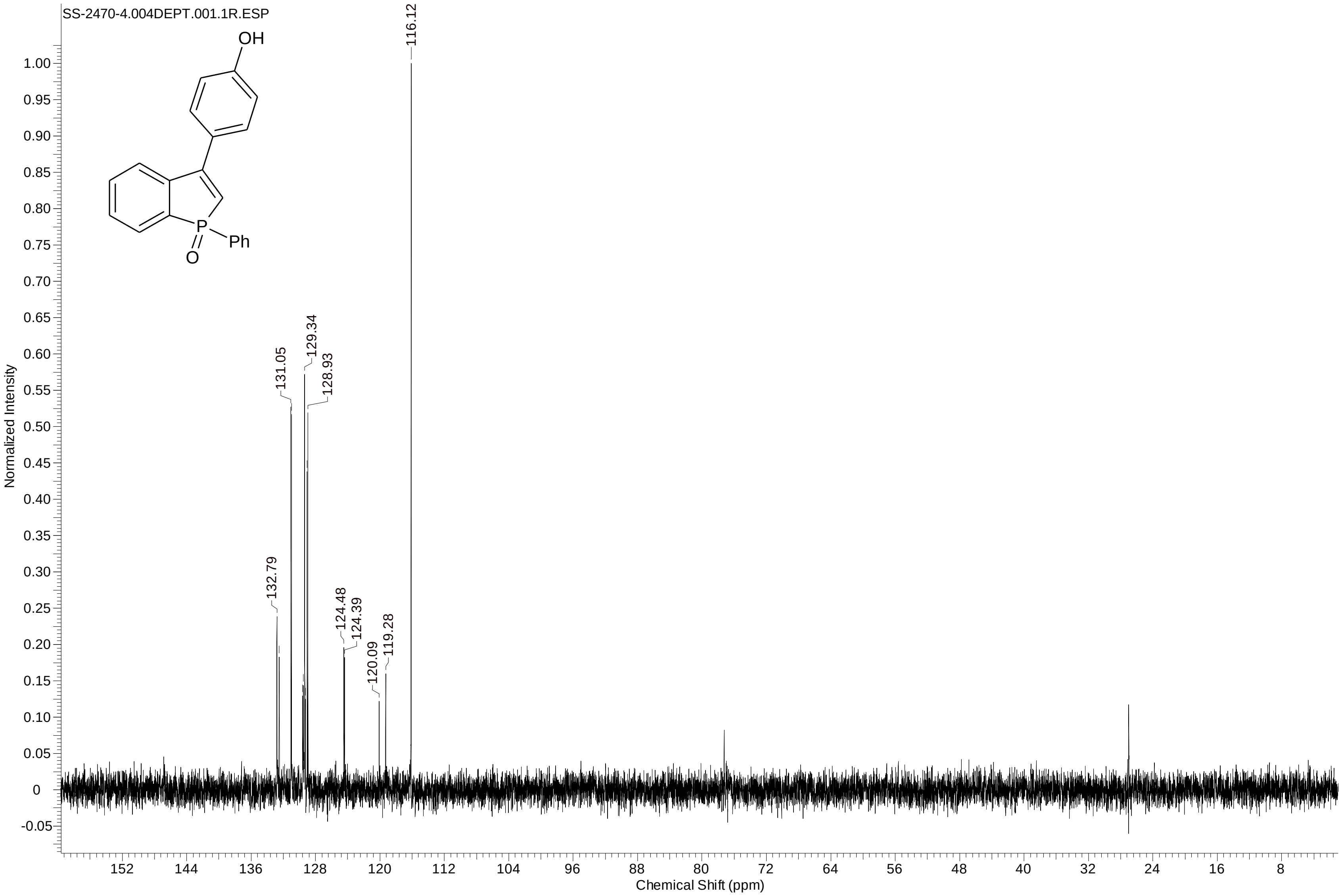

DEPT 135 NMR spectrum of 1-phenyl-3-(*p*-hydroxyphenyl)benzophosphole oxide (**5h**) (125 MHz, $CDCl_3$)

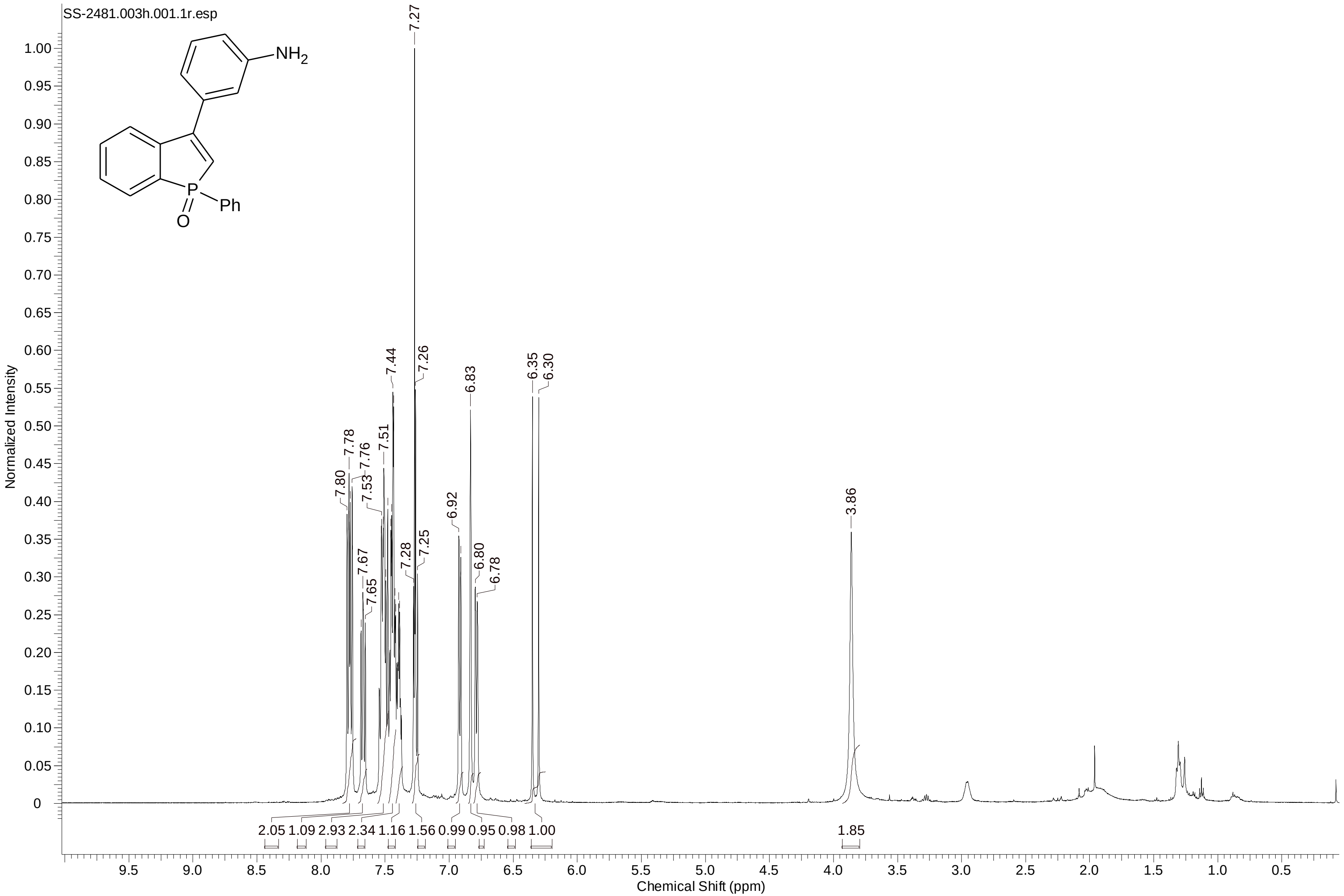

^{1}H NMR spectrum of 1-phenyl-3-(*m*-aminophenyl)benzophosphole oxide (**5i**) (500 MHz, $CDCl_3$)

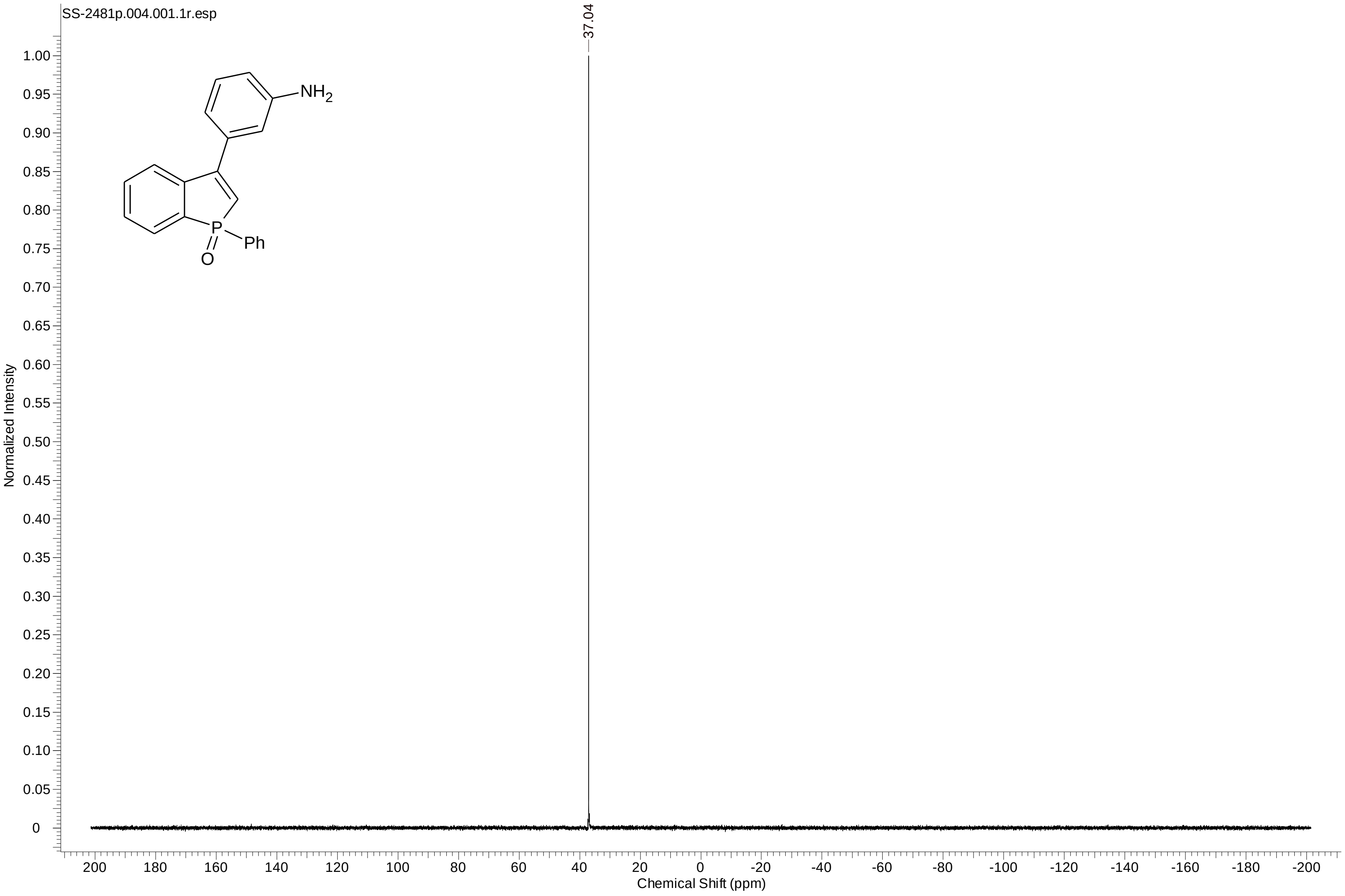

$^{31}P\{^{1}H\}$ NMR spectrum of 1-phenyl-3-(*m*-aminophenyl)benzophosphole oxide (**5i**) (202 MHz, $CDCl_3$)

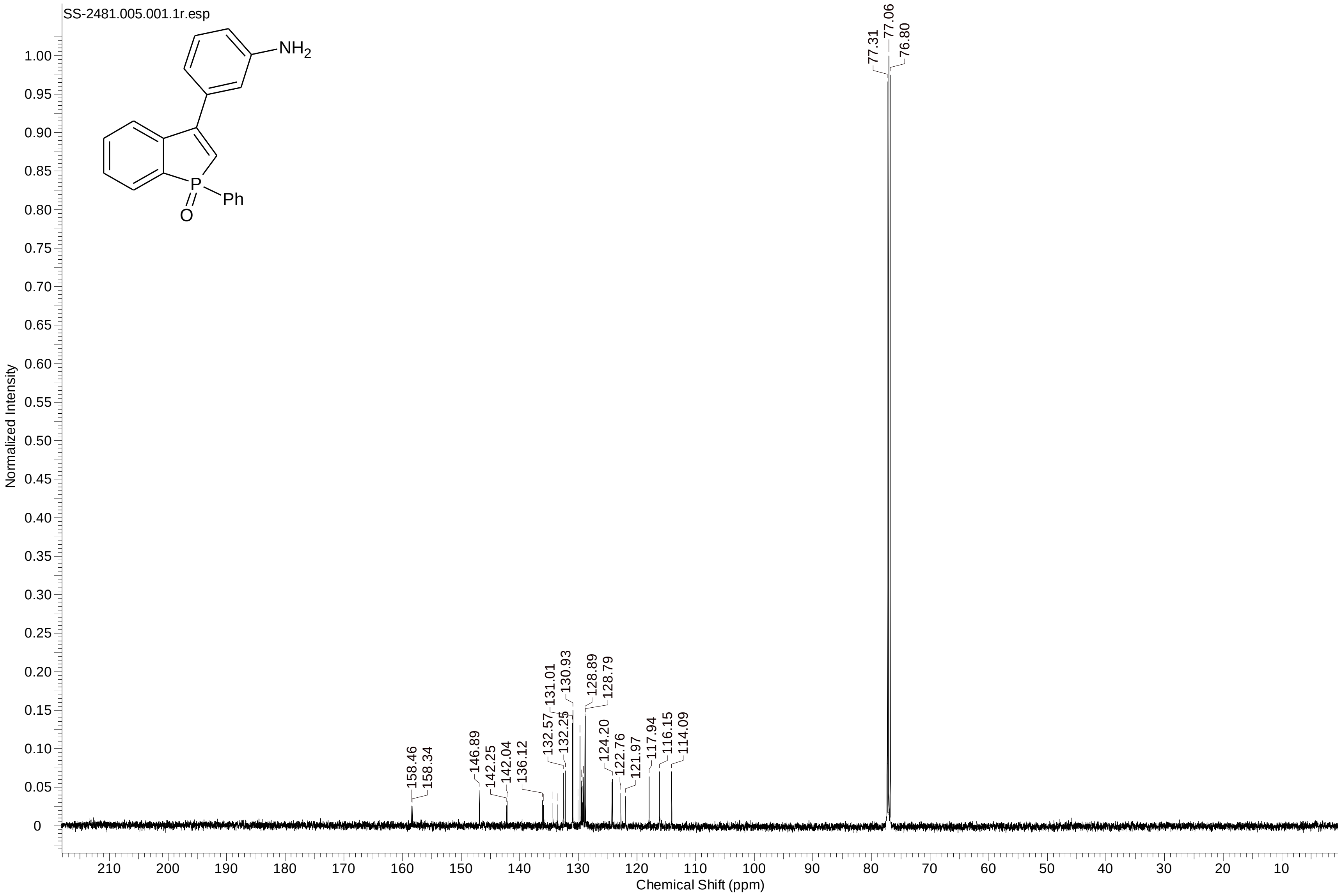

$^{13}C\{^{1}H\}$ NMR spectrum of 1-phenyl-3-(*m*-aminophenyl)benzophosphole oxide (**5i**) (125 MHz, $CDCl_3$)

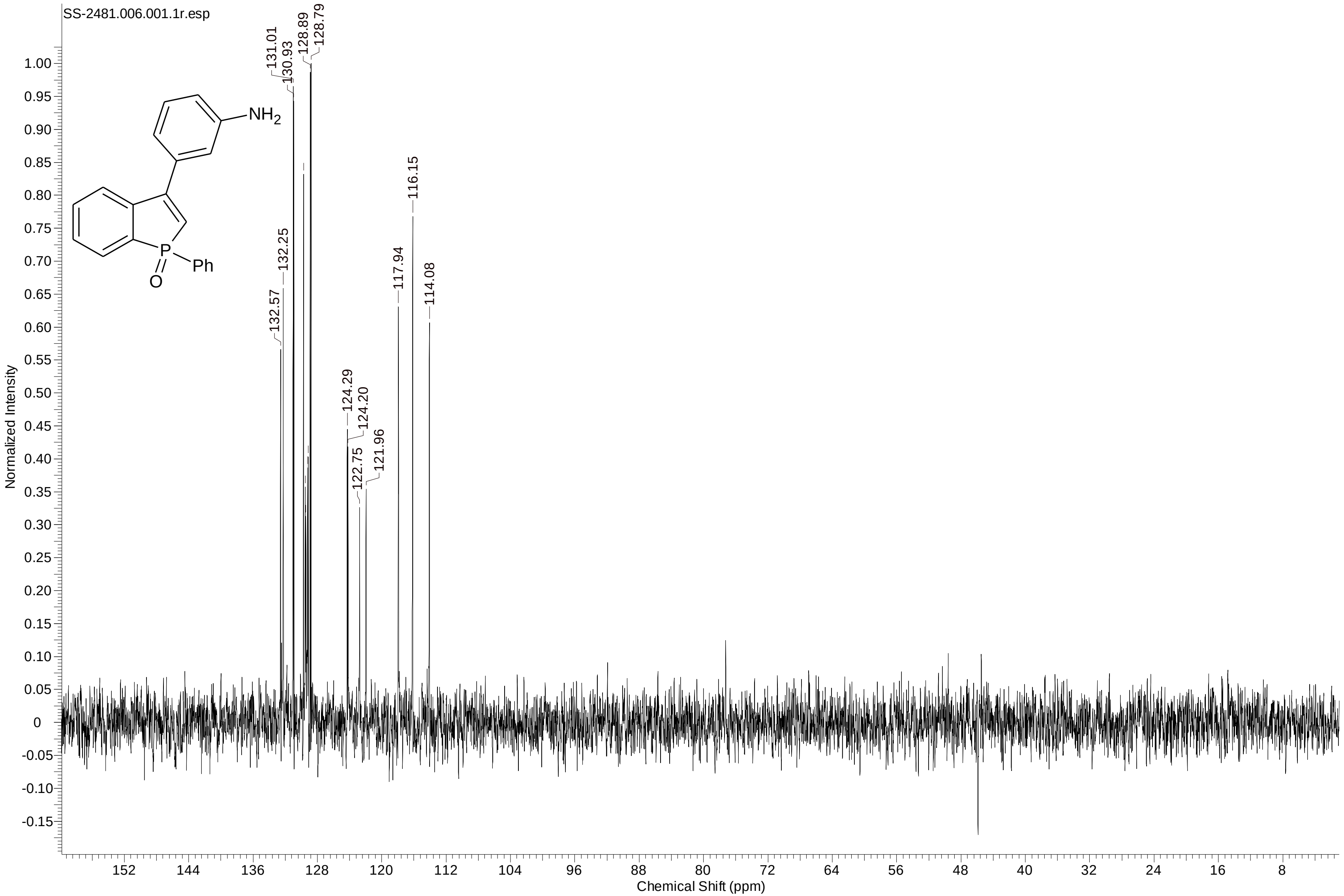

DEPT 135 NMR spectrum of 1-phenyl-3-(*m*-aminophenyl)benzophosphole oxide (**5i**) (125 MHz, $CDCl_3$)

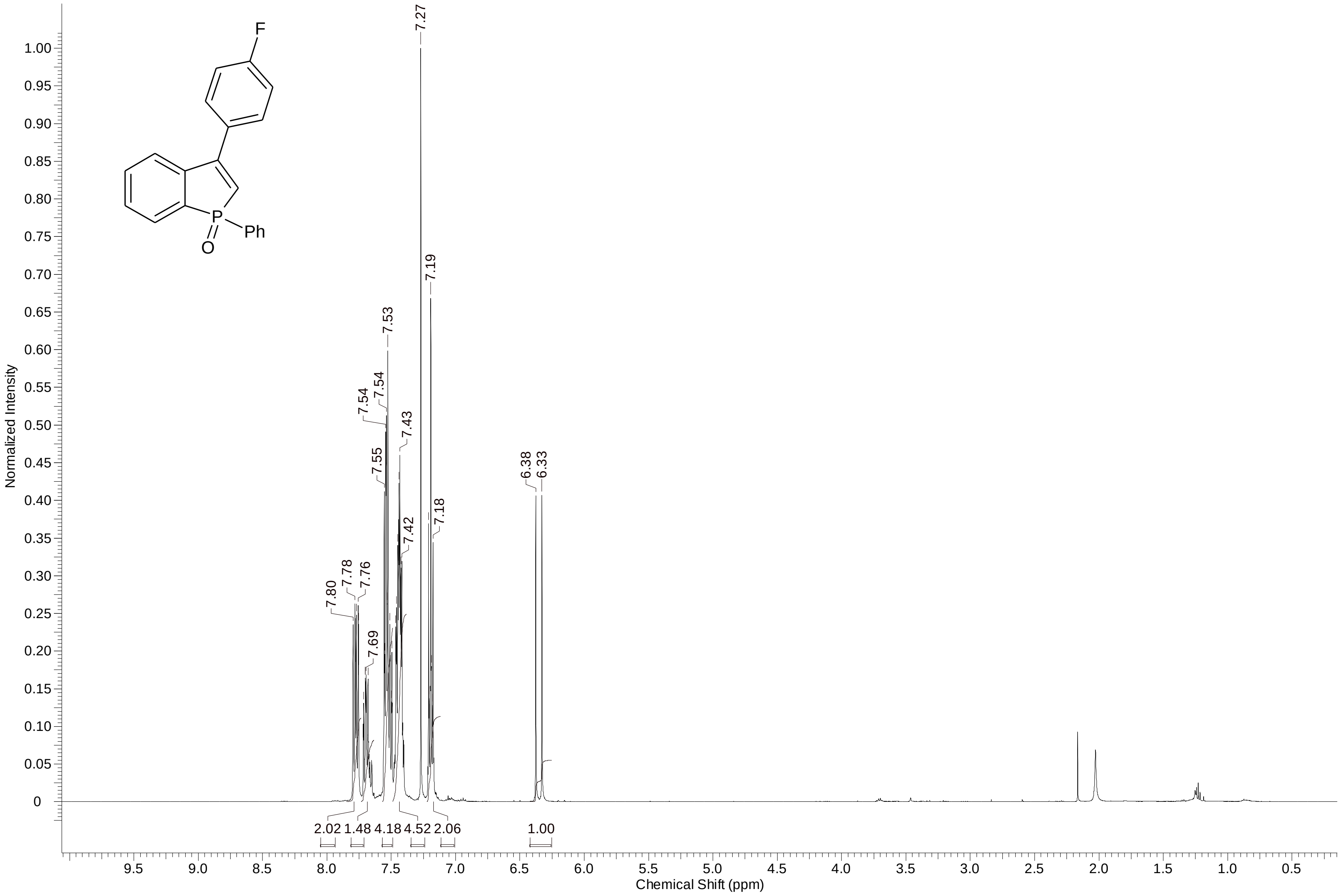

^{1}H NMR spectrum of 1-phenyl-3-(*p*-fluorophenyl)benzophosphole oxide (**5j**) (500 MHz, $CDCl_3$)

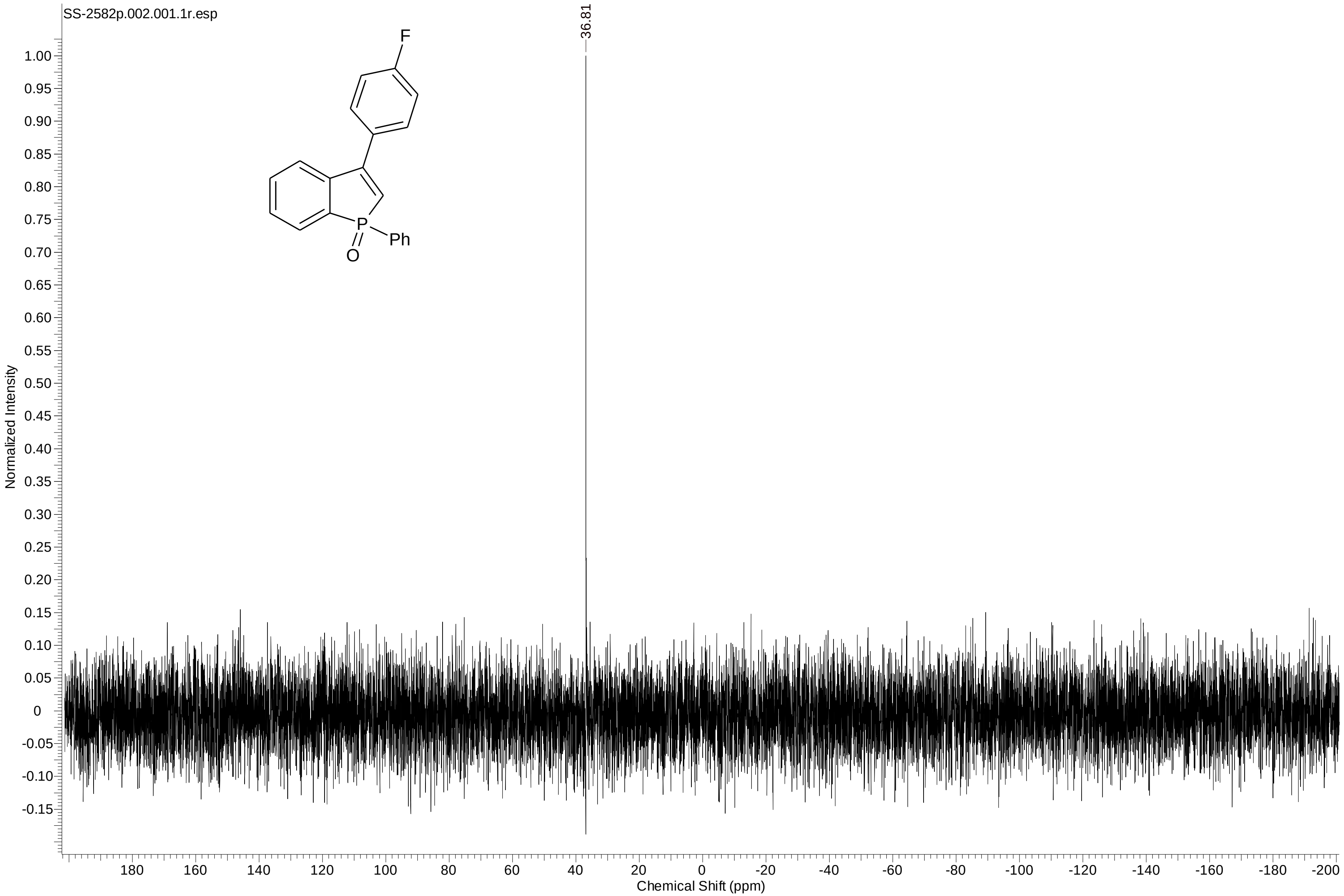

$^{31}P\{^{1}H\}$ NMR spectrum of 1-phenyl-3-(*p*-fluorophenyl)benzophosphole oxide (**5j**) (202 MHz, $CDCl_3$)

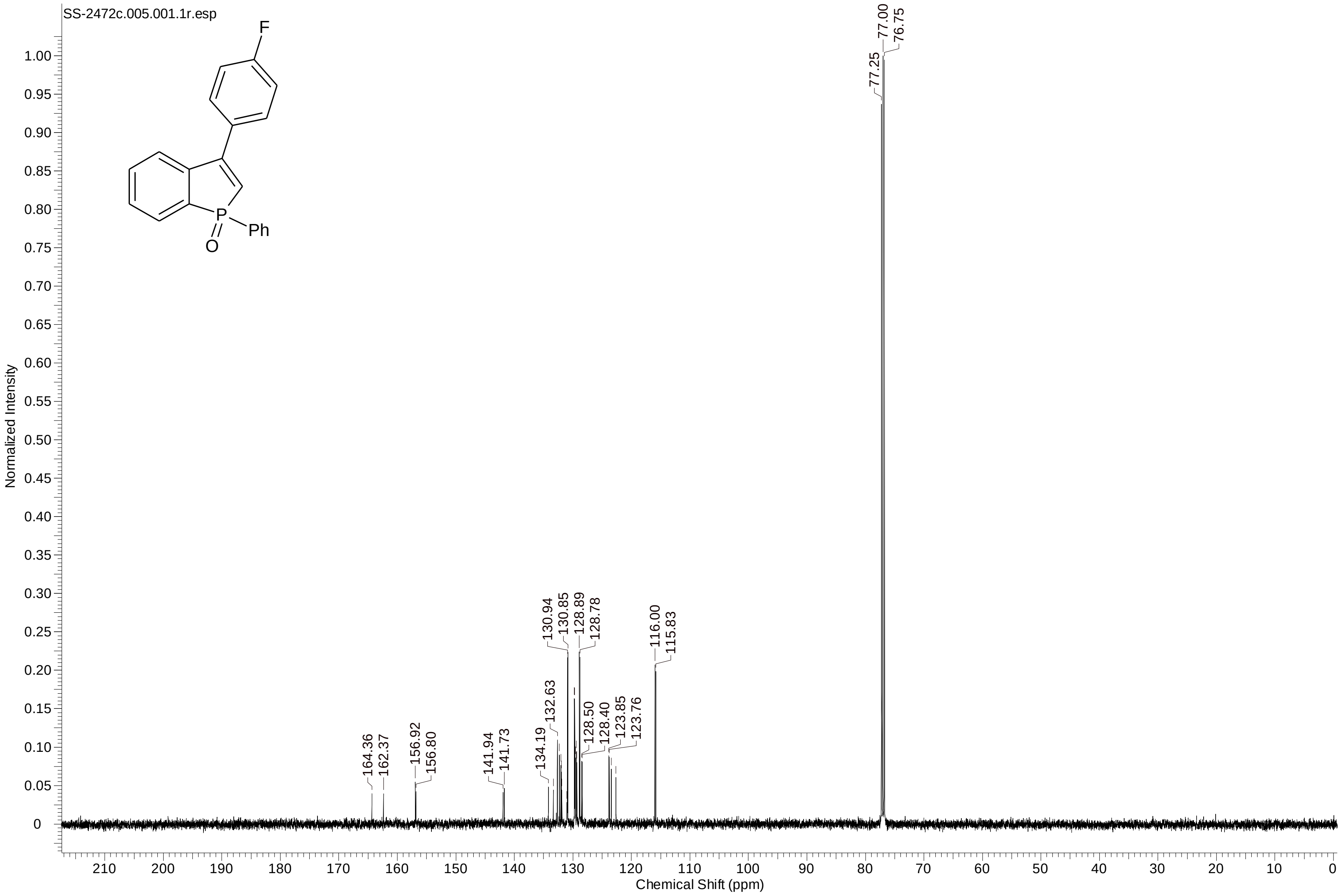

$^{13}C\{^{1}H\}$ NMR spectrum of 1-phenyl-3-(*p*-fluorophenyl)benzophosphole oxide (**5j**) (125 MHz, $CDCl_3$)

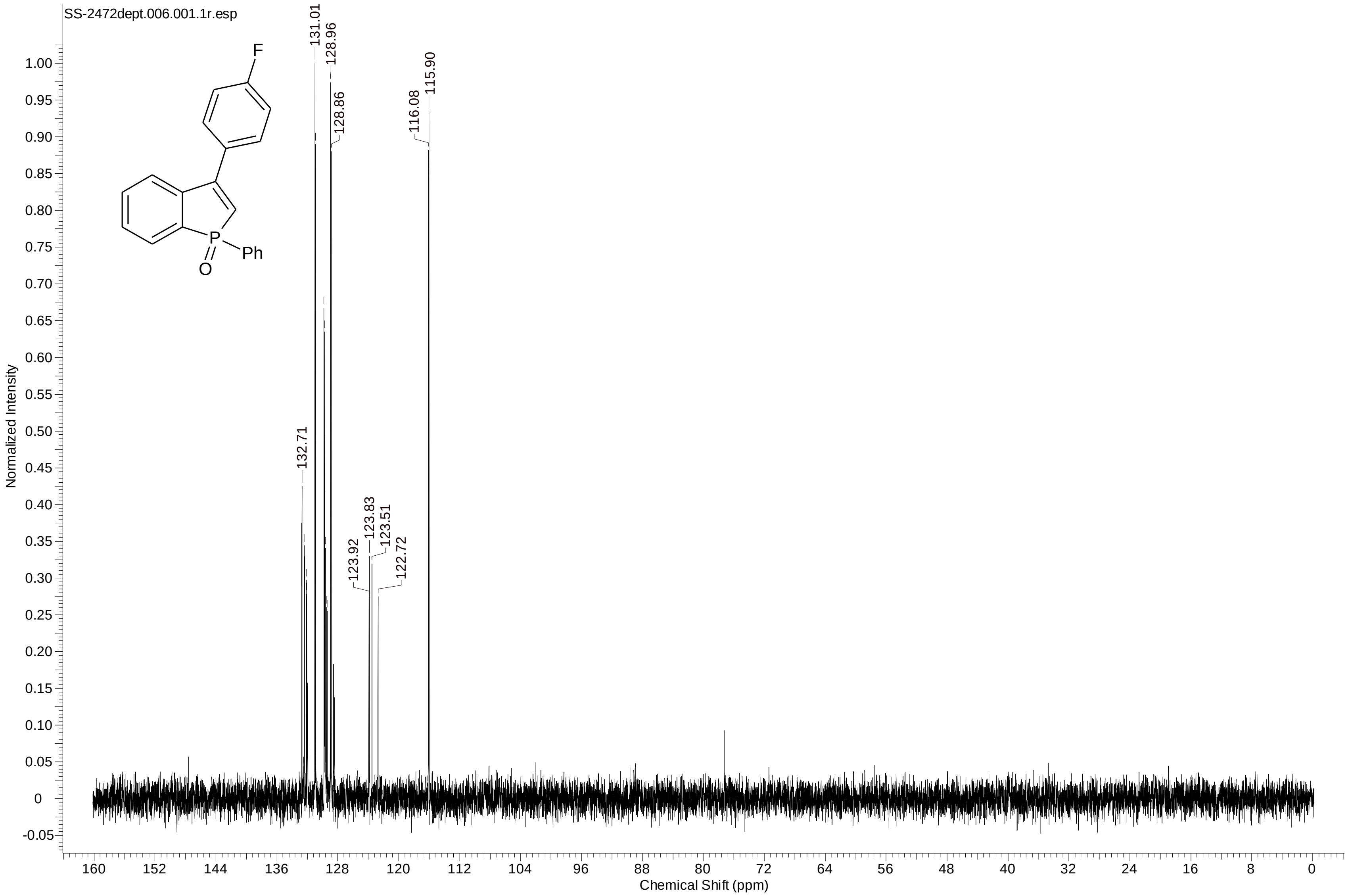

DEPT 135 NMR spectrum of 1-phenyl-3-(*p*-fluorophenyl)benzophosphole oxide (**5j**) (125 MHz, $CDCl_3$)

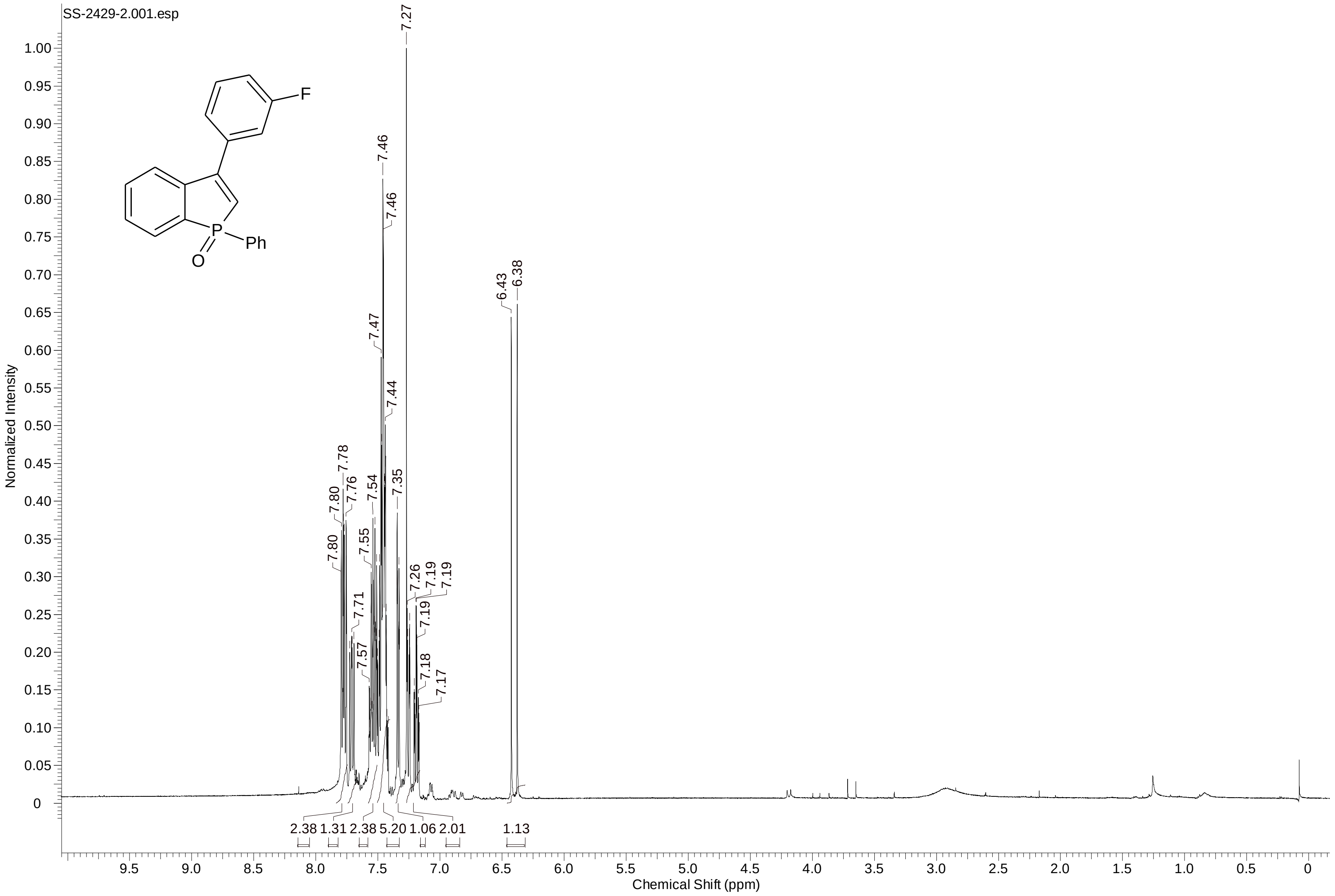

^{1}H NMR spectrum of 1-phenyl-3-(*m*-fluorophenyl)benzophosphole oxide (**5k**) (500 MHz, $CDCl_3$)

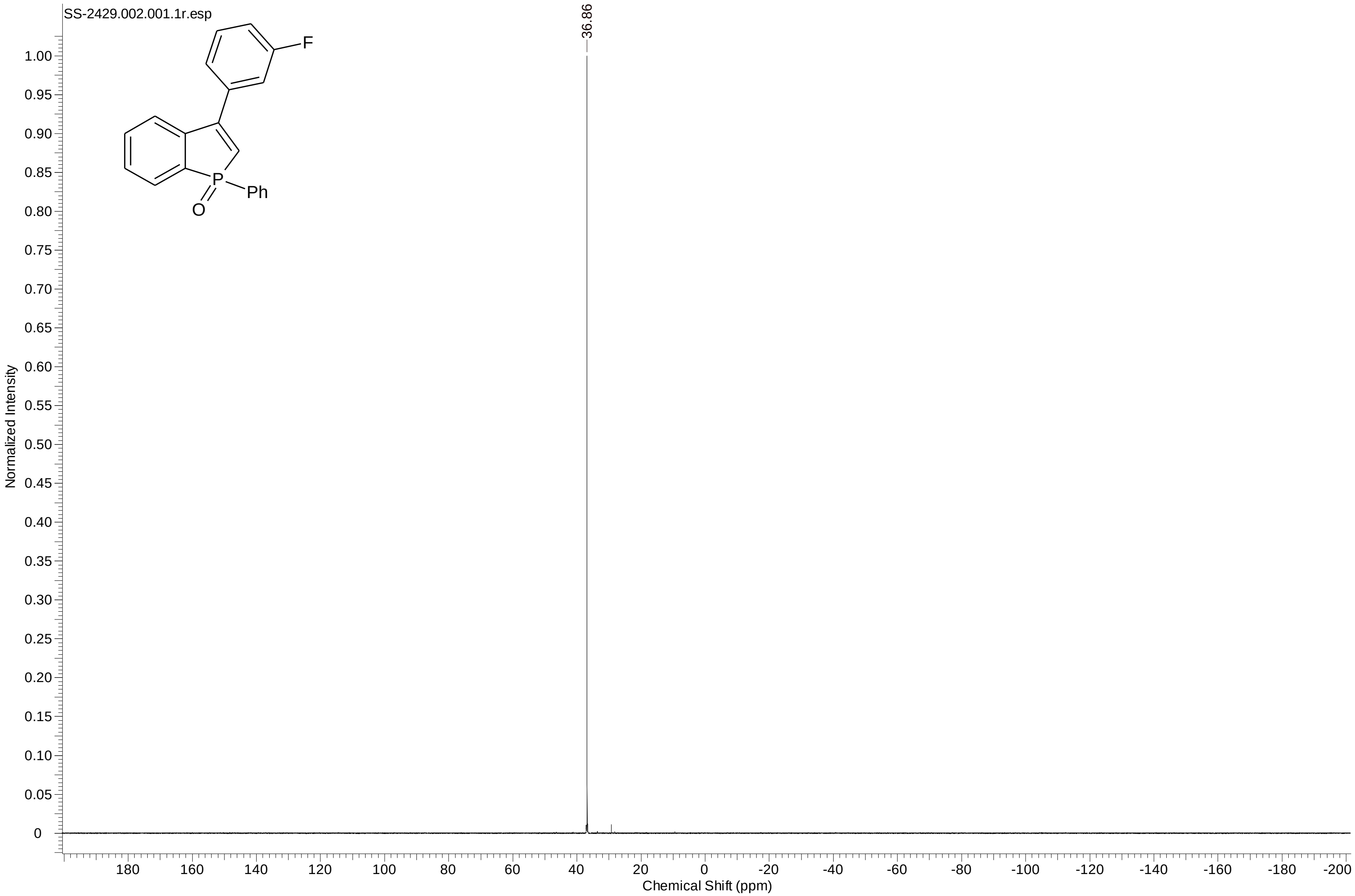

$^{31}P\{^{1}H\}$ NMR spectrum of 1-phenyl-3-(*m*-fluorophenyl)benzophosphole oxide (**5k**) (202 MHz, $CDCl_3$)

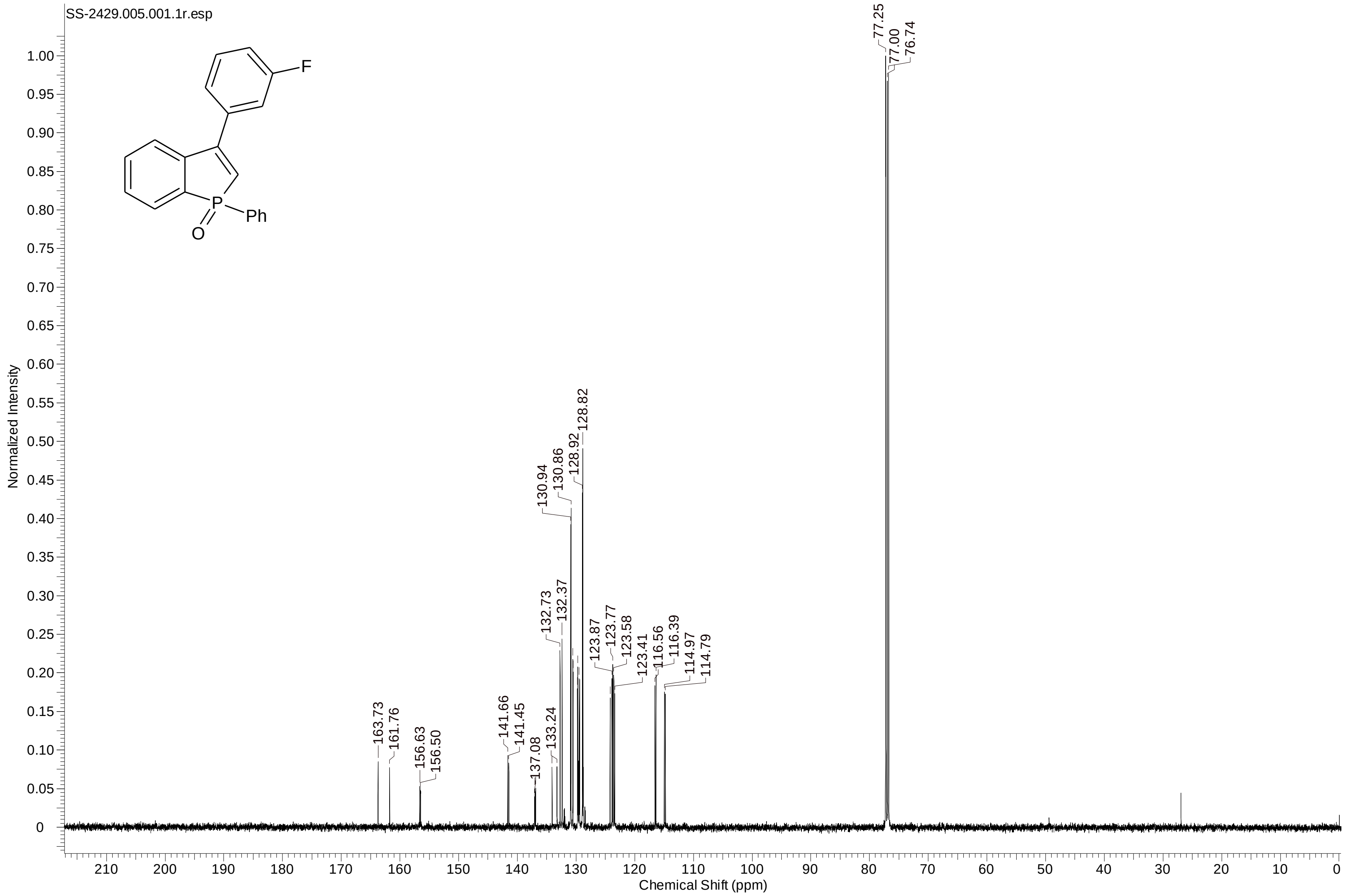

$^{13}C\{^{1}H\}$ NMR spectrum of 1-phenyl-3-(*m*-fluorophenyl)benzophosphole oxide (**5k**) (125 MHz, $CDCl_3$)

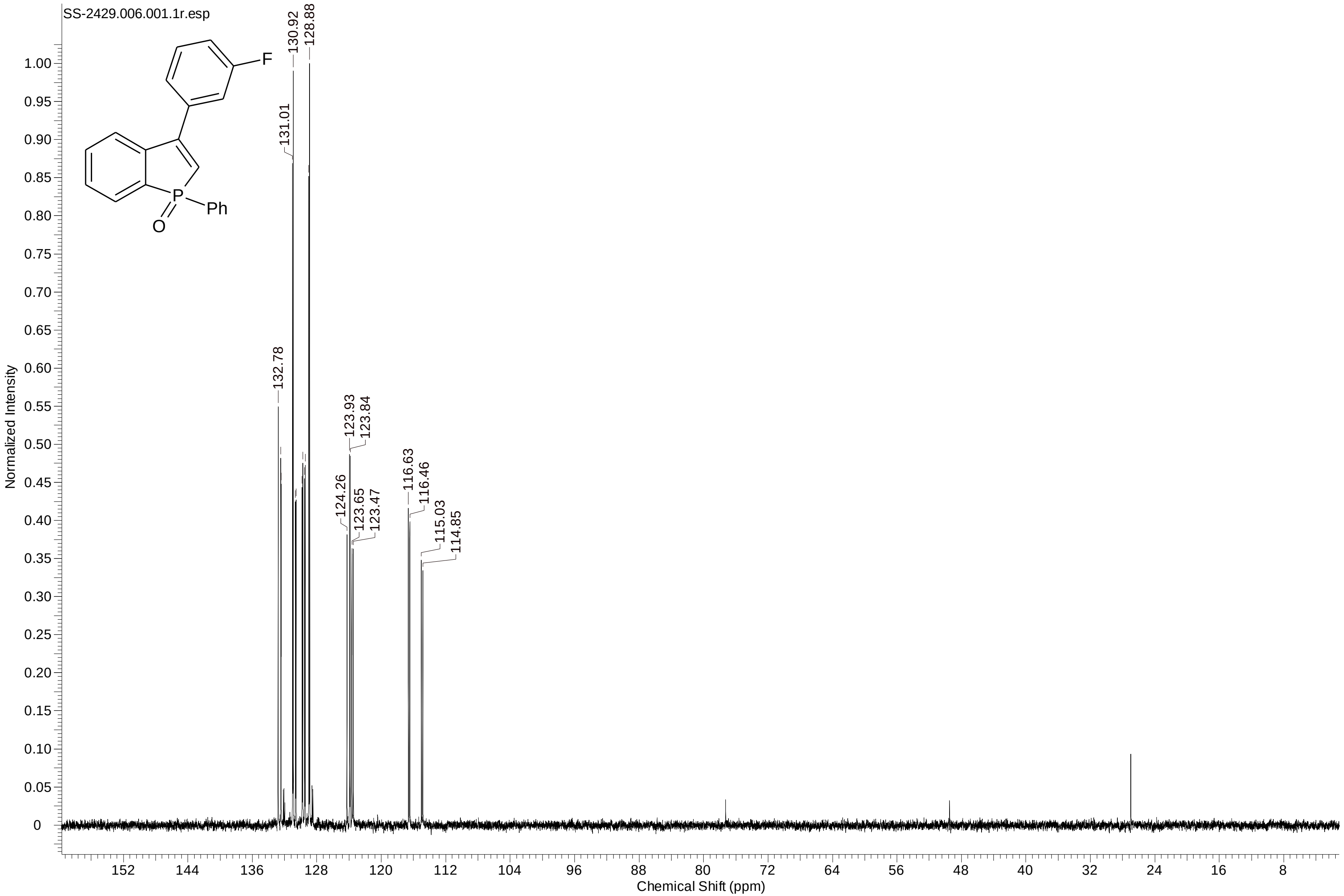

DEPT 135 NMR spectrum of 1-phenyl-3-(*m*-fluorophenyl)benzophosphole oxide (**5k**) (125 MHz, $CDCl_3$)

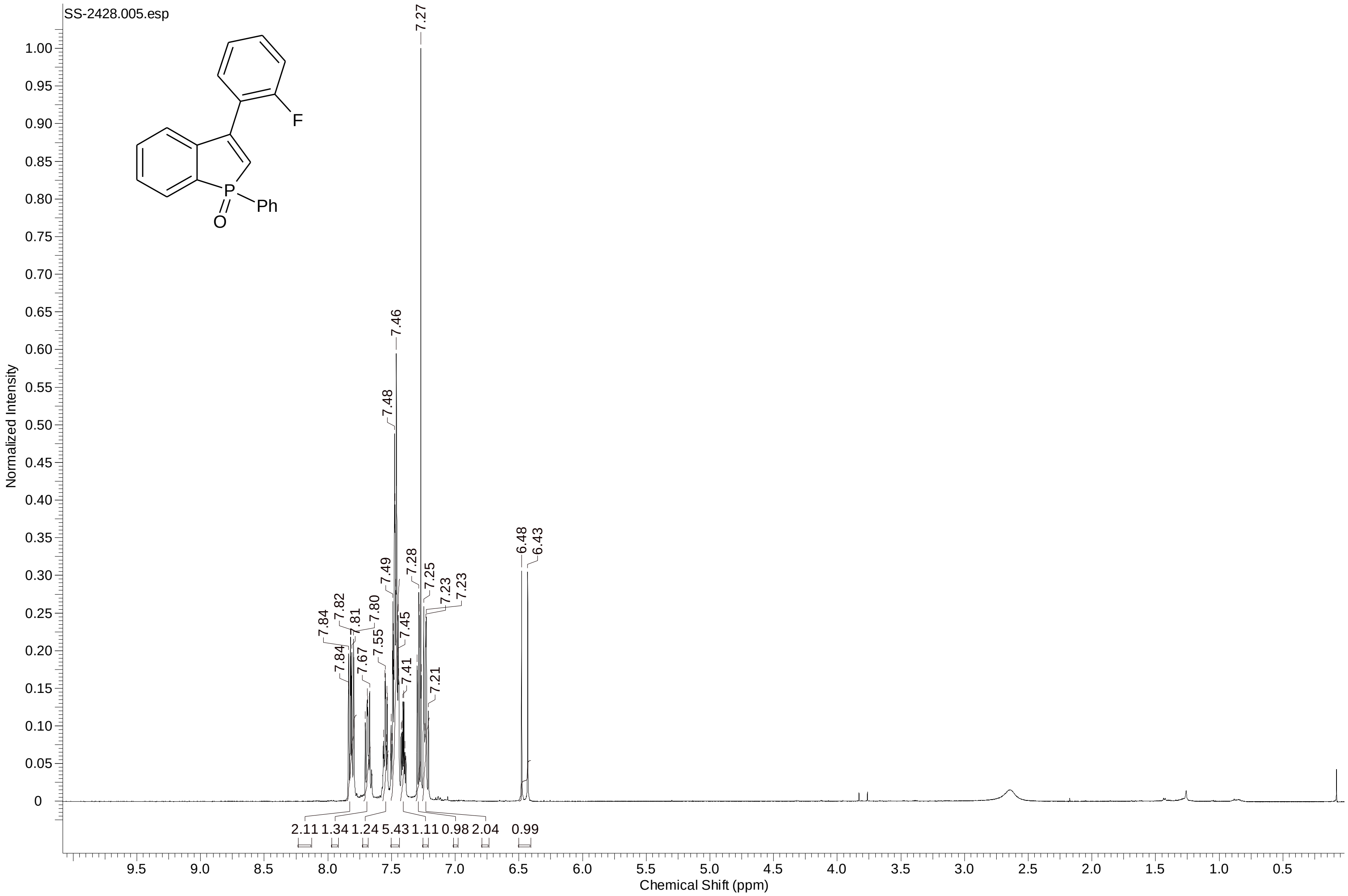

^{1}H NMR spectrum of 1-phenyl-3-(*o*-fluorophenyl)benzophosphole oxide (**5l**) (500 MHz, $CDCl_3$)

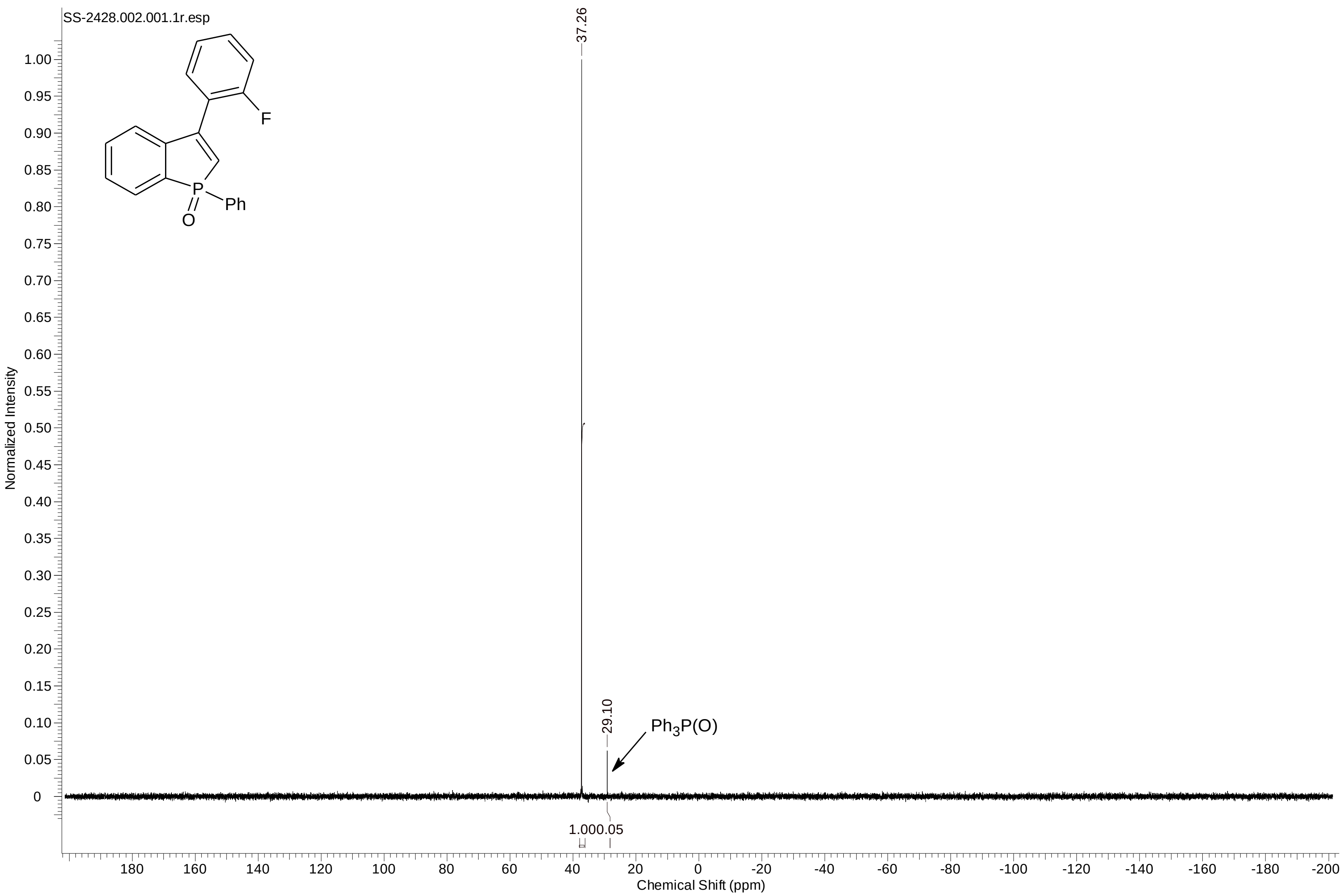

$^{31}P\{^{1}H\}$ NMR spectrum of 1-phenyl-3-(*o*-fluorophenyl)benzophosphole oxide (**5l**) (202 MHz, $CDCl_3$)

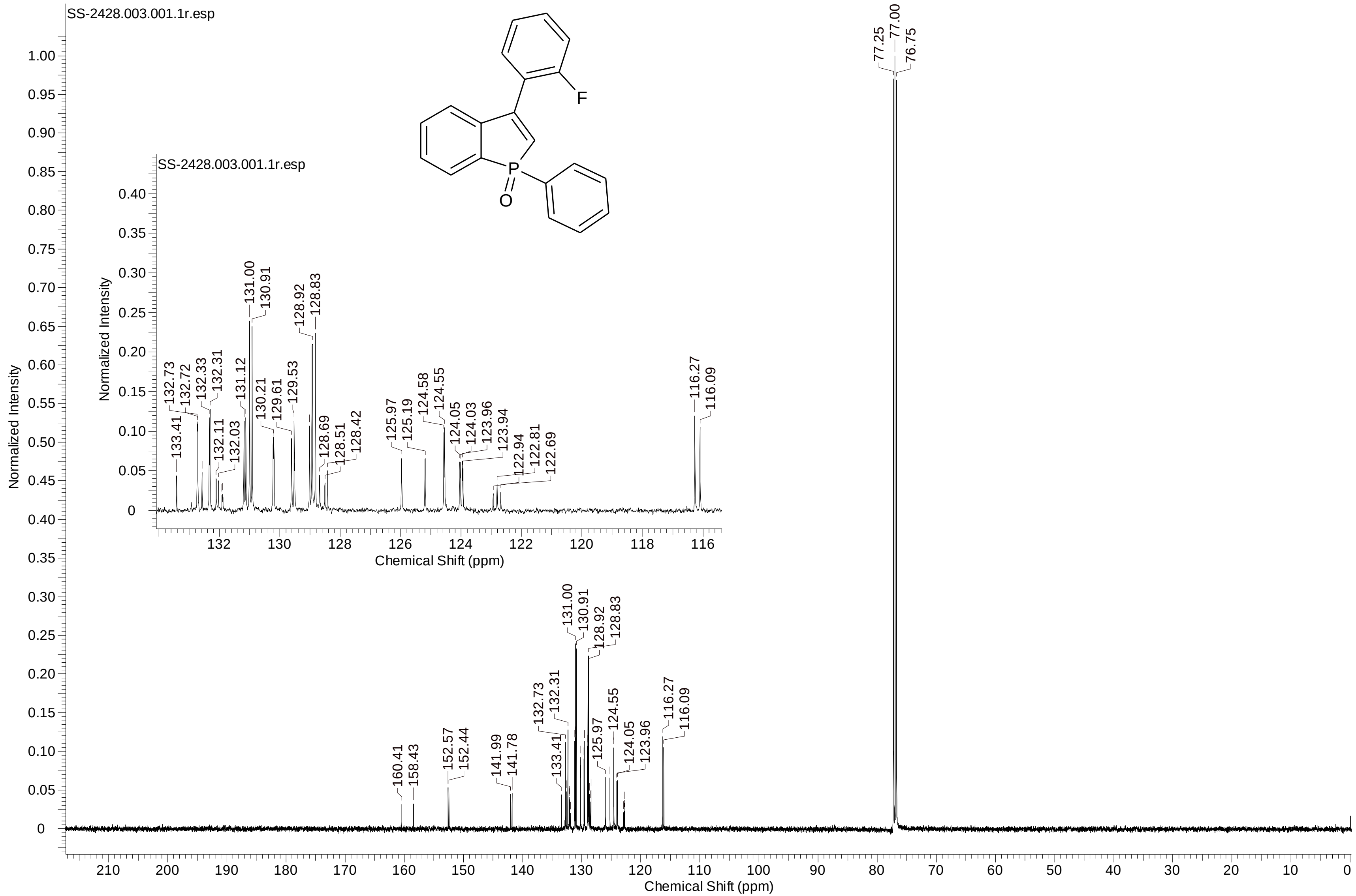

$^{13}C\{^{1}H\}$ NMR spectrum of 1-phenyl-3-(*o*-fluorophenyl)benzophosphole oxide (**5l**) (125 MHz, $CDCl_3$)

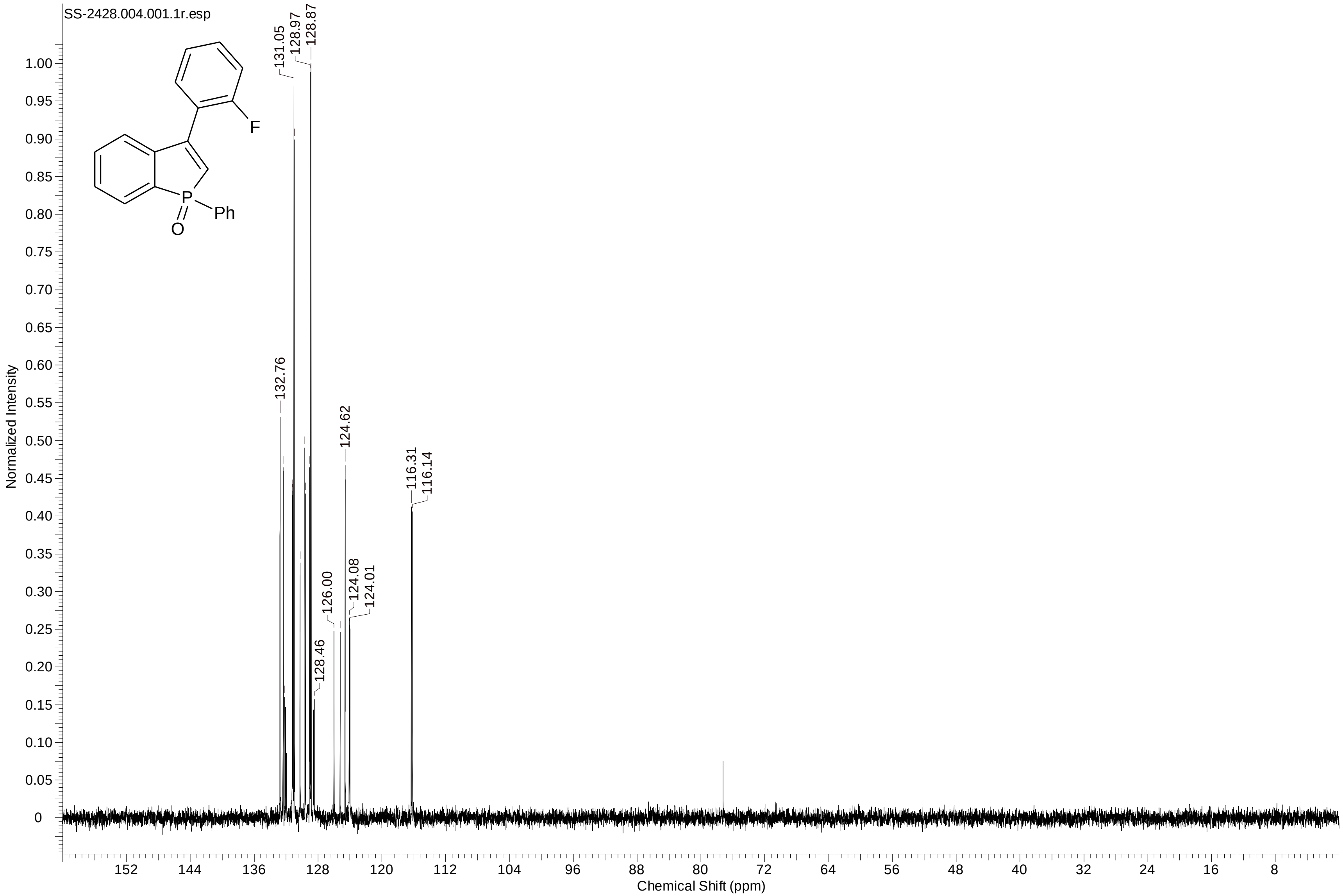

DEPT 135 NMR spectrum of 1-phenyl-3-(*o*-fluorophenyl)benzophosphole oxide (**5l**) (125 MHz, $CDCl_3$)

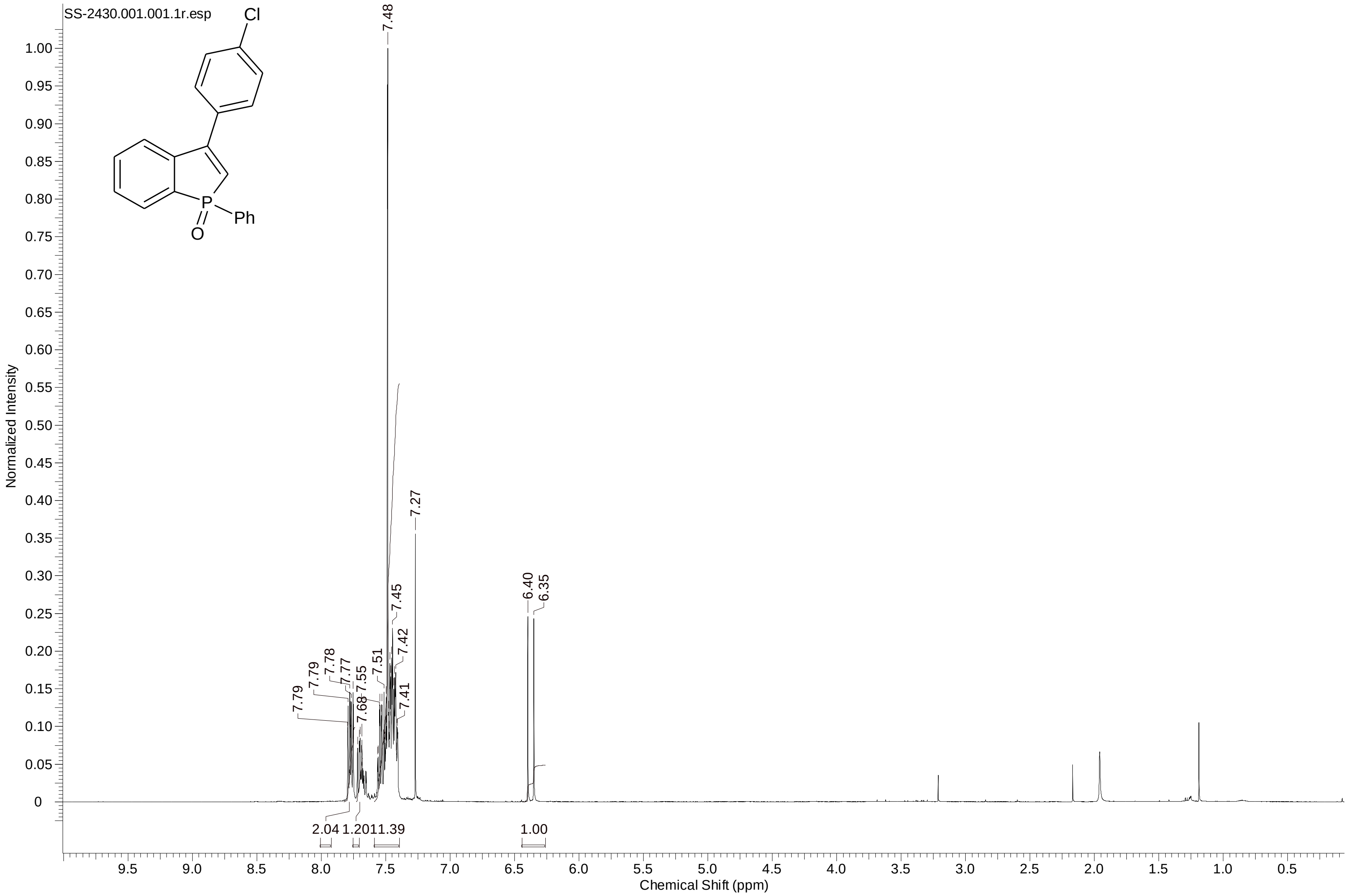

^{1}H NMR spectrum of 1-phenyl-3-(*p*-chlorophenyl)benzophosphole oxide (**5m**) (500 MHz, $CDCl_3$)

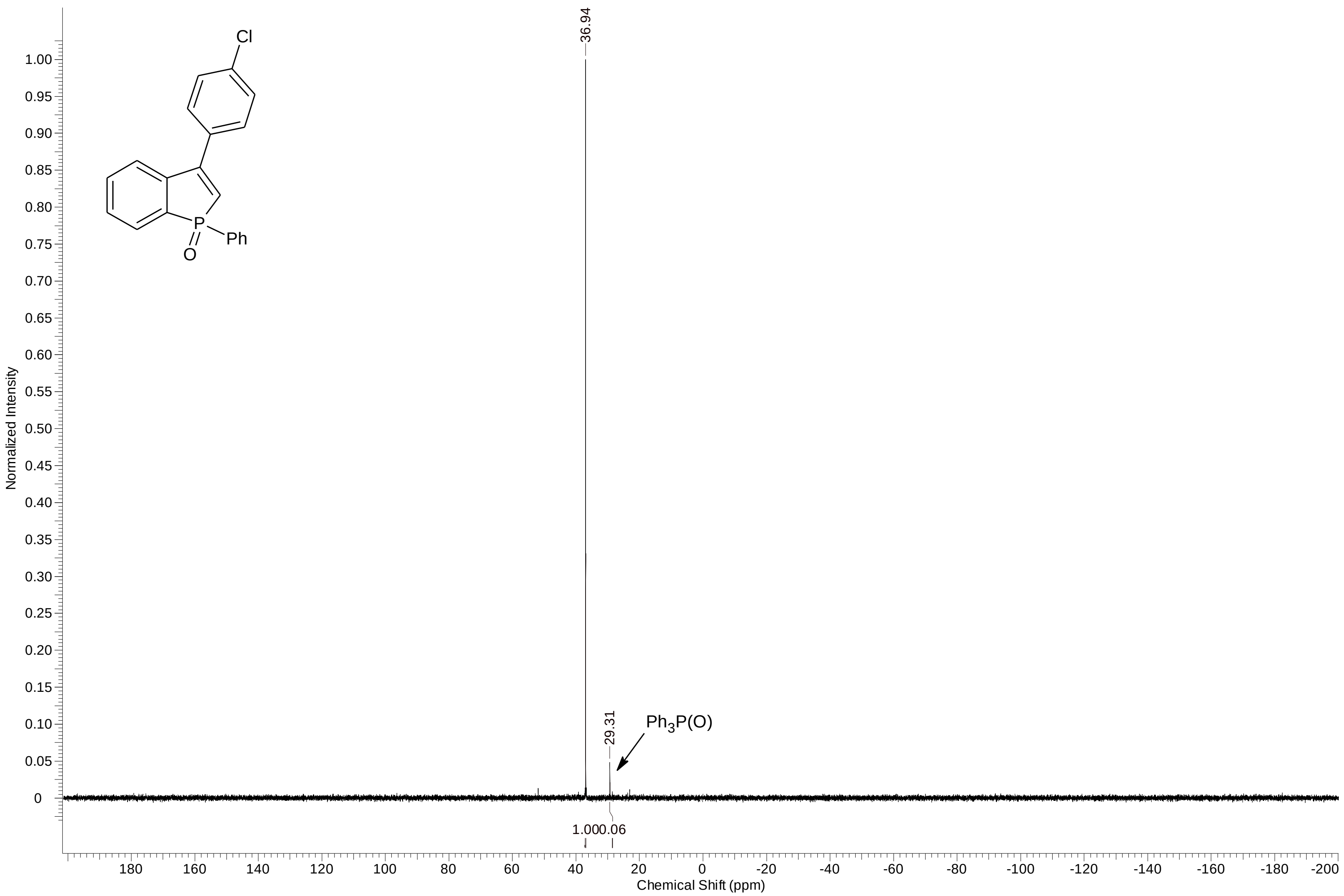

$^{31}P\{^{1}H\}$ NMR spectrum of 1-phenyl-3-(*p*-chlorophenyl)benzophosphole oxide (**5m**) (202 MHz, $CDCl_3$)

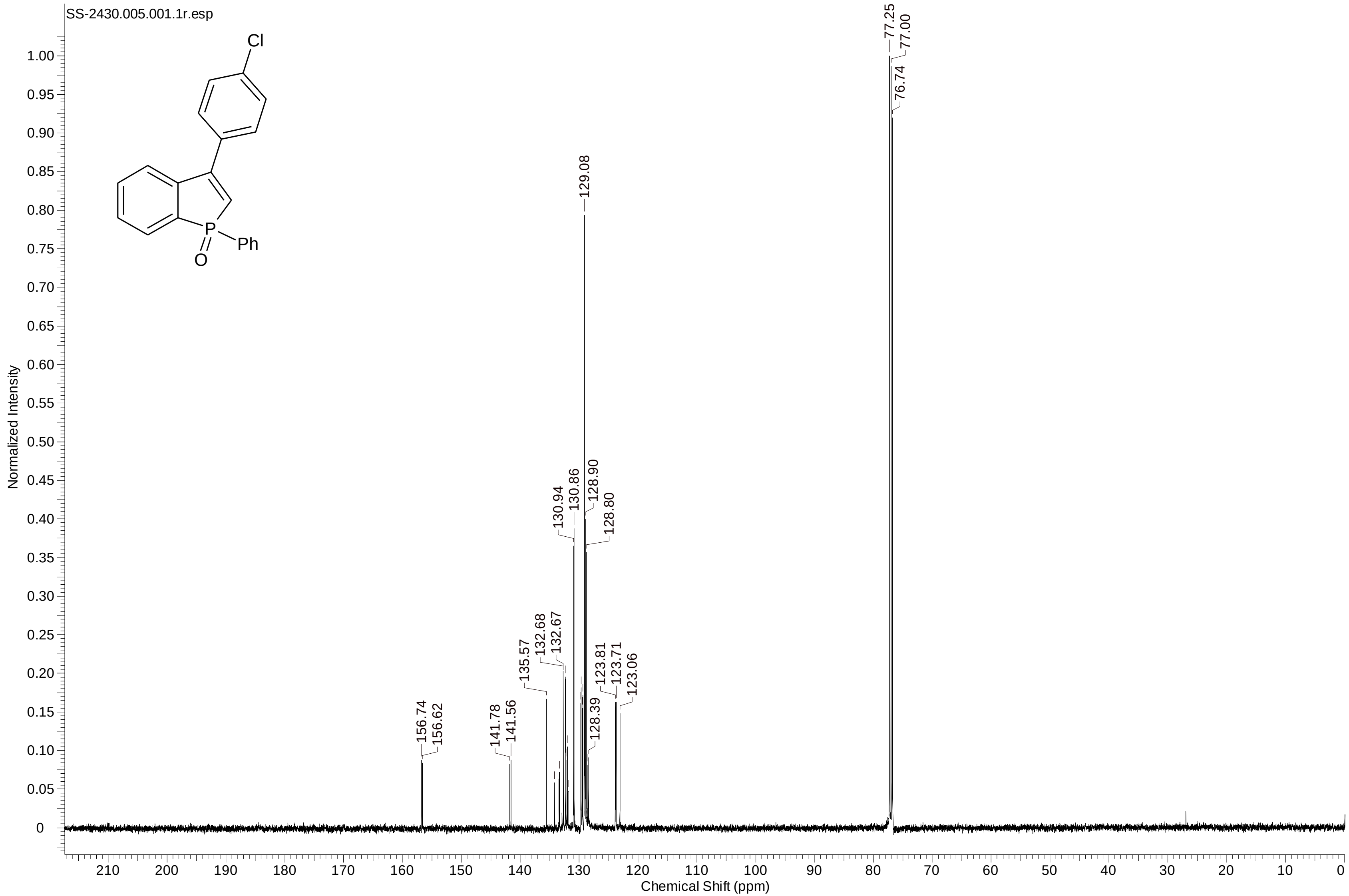

$^{13}C\{^1H\}$ NMR spectrum of 1-phenyl-3-(*p*-chlorophenyl)benzophosphole oxide (**5m**) (125 MHz, $CDCl_3$)

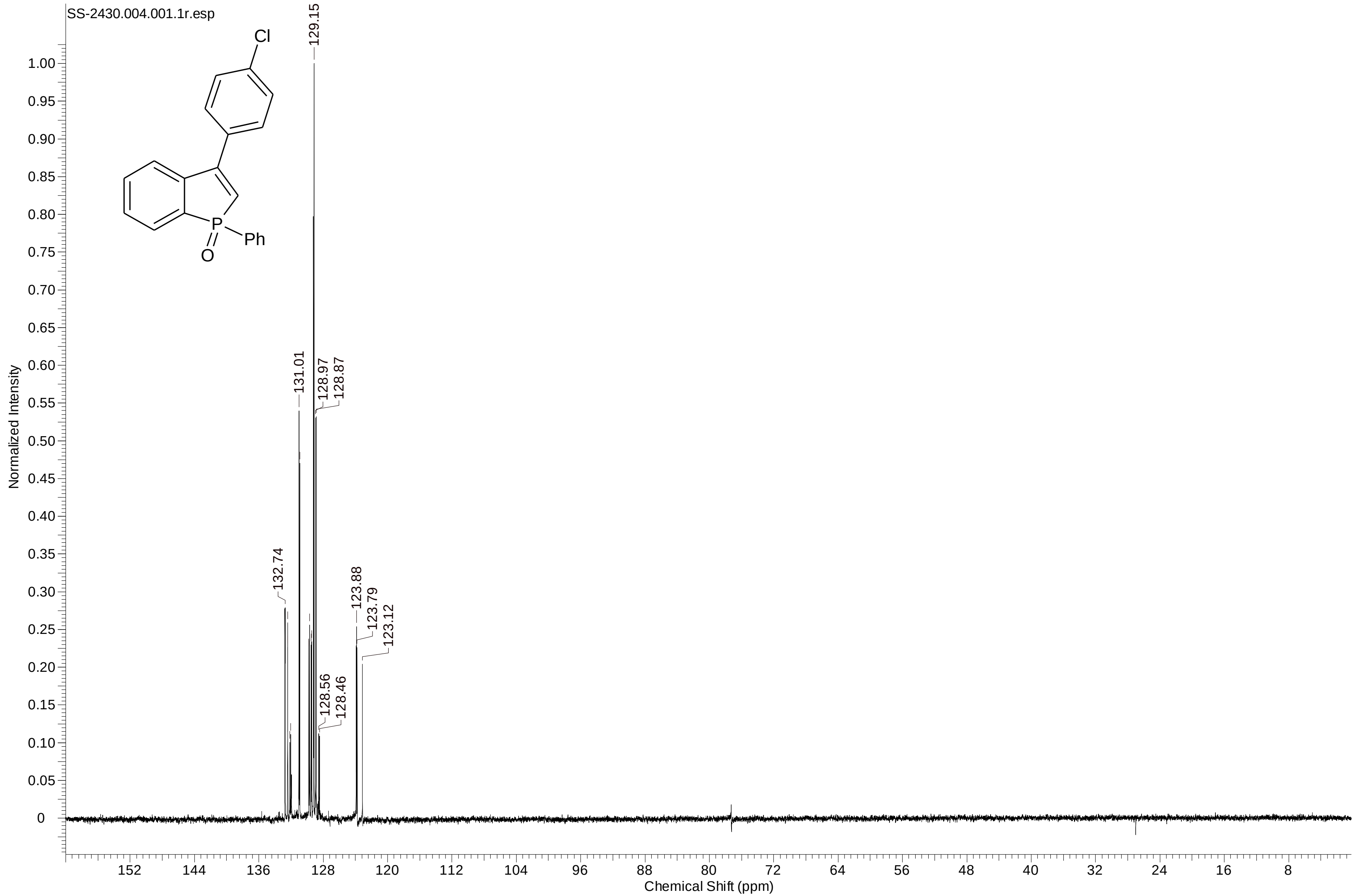

DEPT 135 NMR spectrum of 1-phenyl-3-(*p*-chlorophenyl)benzophosphole oxide (**5m**) (125 MHz, $CDCl_3$)

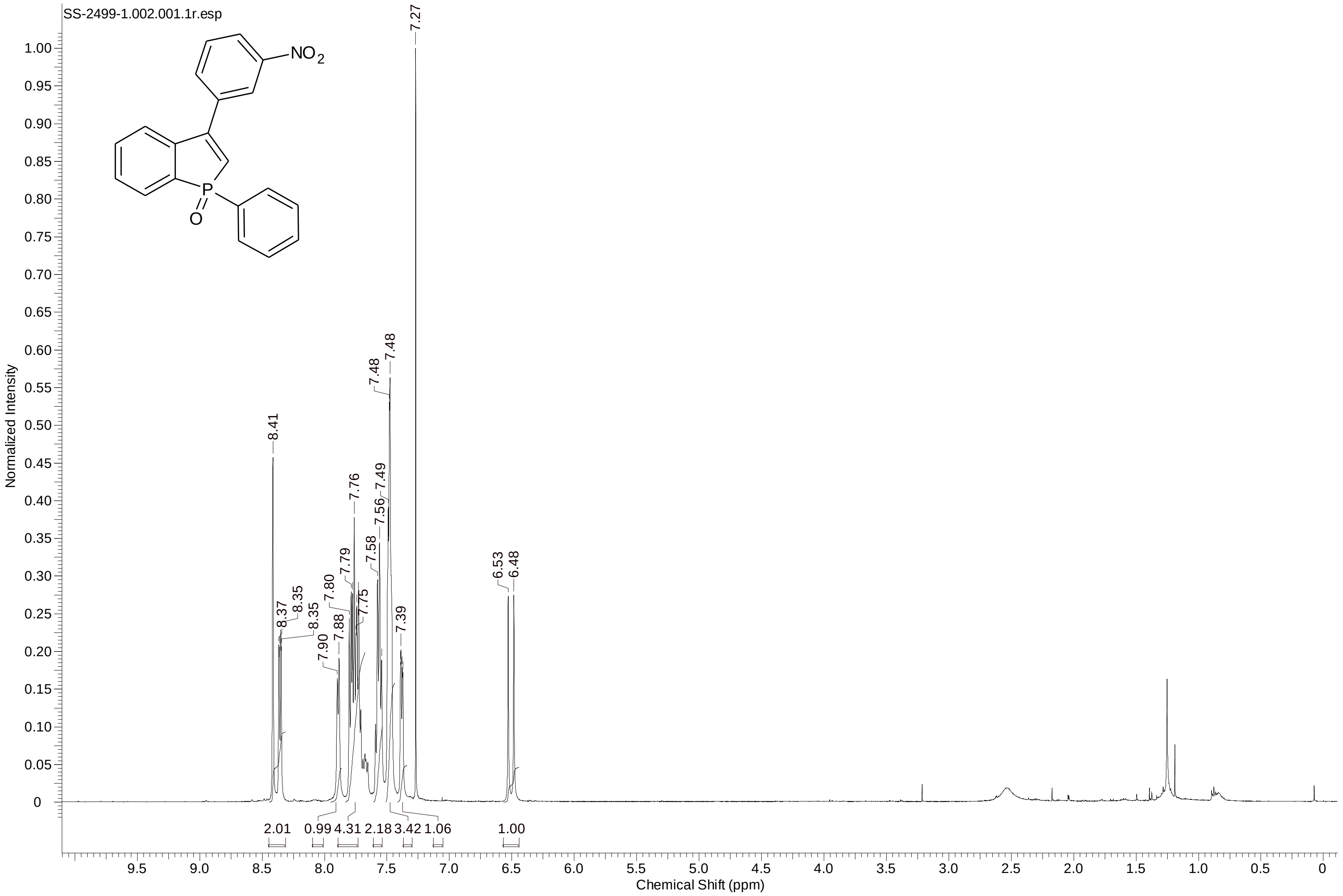

^{1}H NMR spectrum of 1-phenyl-3-(*m*-nitrophenyl)benzophosphole oxide (**5n**) (500 MHz, $CDCl_3$)

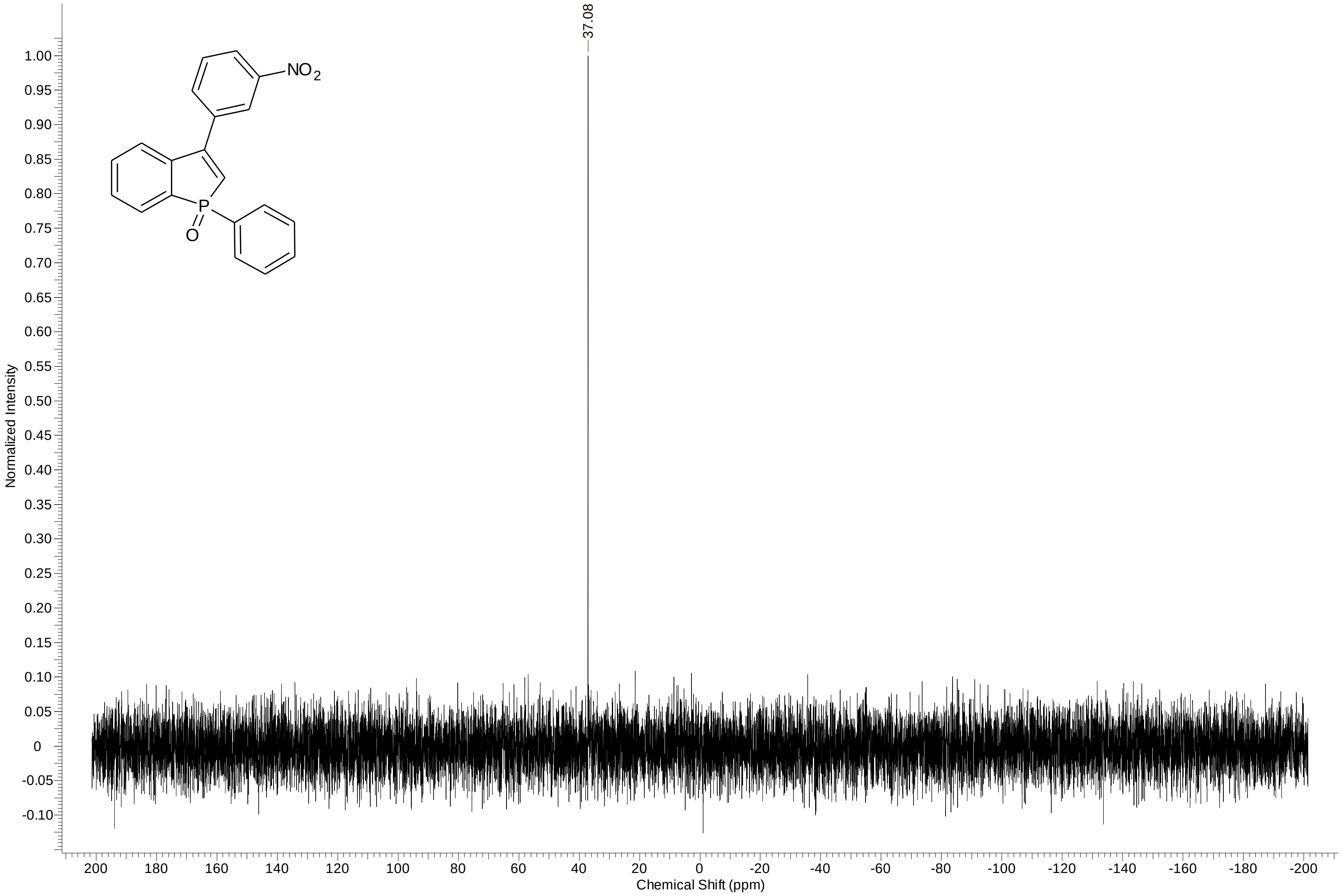

$^{31}P\{^{1}H\}$ NMR spectrum of 1-phenyl-3-(*m*-nitrophenyl)benzophosphole oxide (**5n**) (202 MHz, $CDCl_3$)

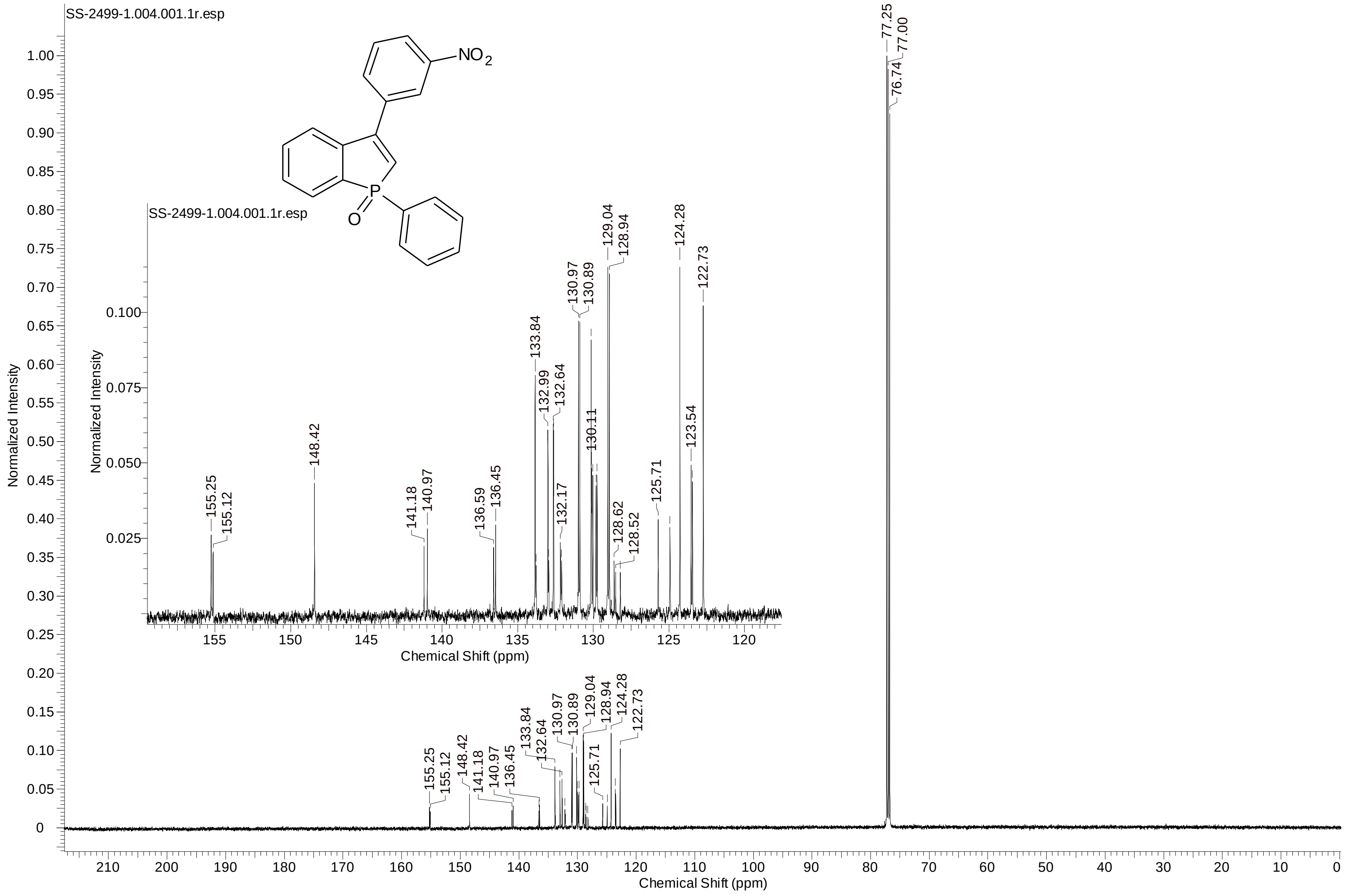

$^{13}C\{^1H\}$ NMR spectrum of 1-phenyl-3-(*m*-nitrophenyl)benzophosphole oxide (**5n**) (125 MHz, $CDCl_3$)

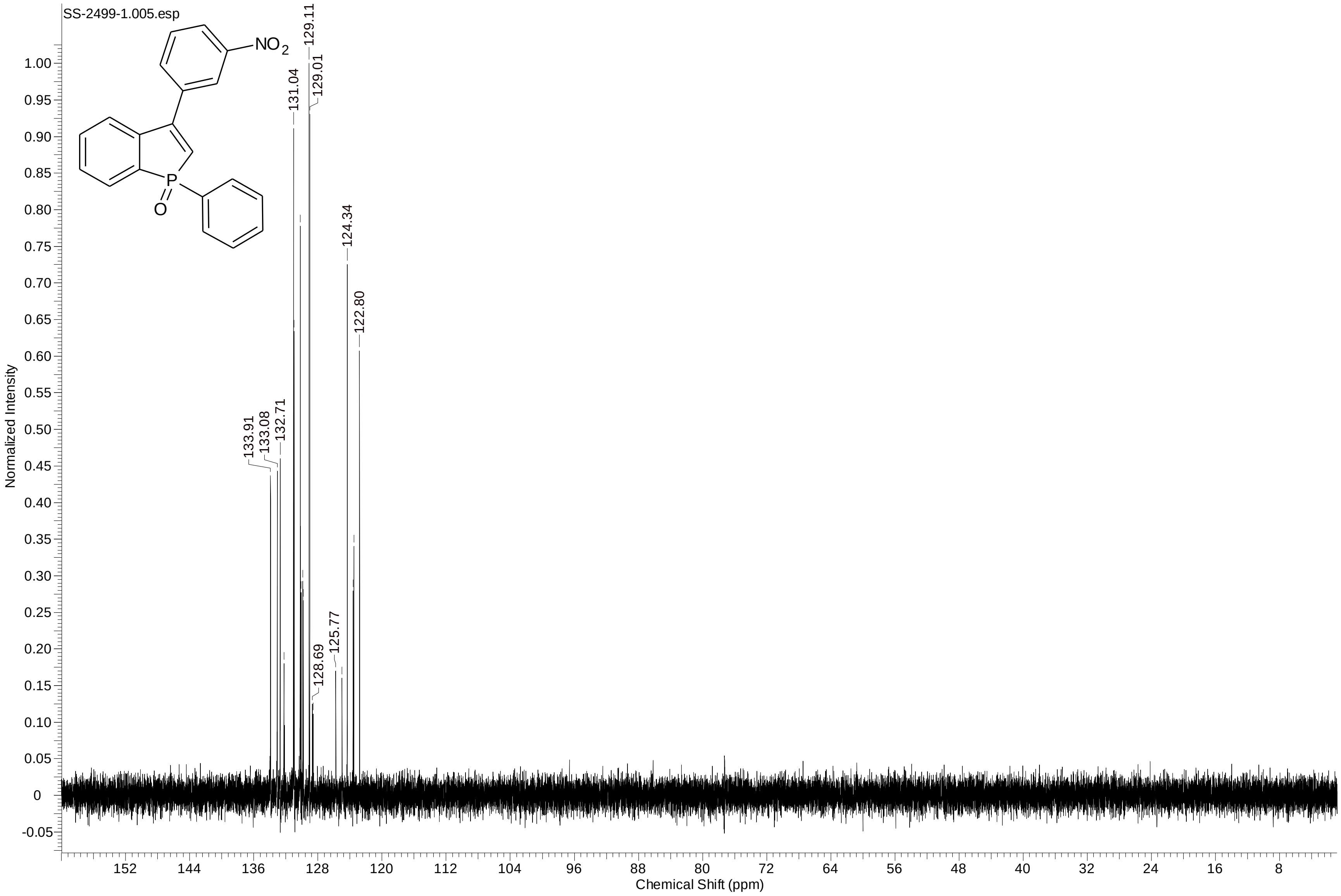

DEPT 135 NMR spectrum of 1-phenyl-3-(*m*-nitrophenyl)benzophosphole oxide (**5n**) (125 MHz, $CDCl_3$)

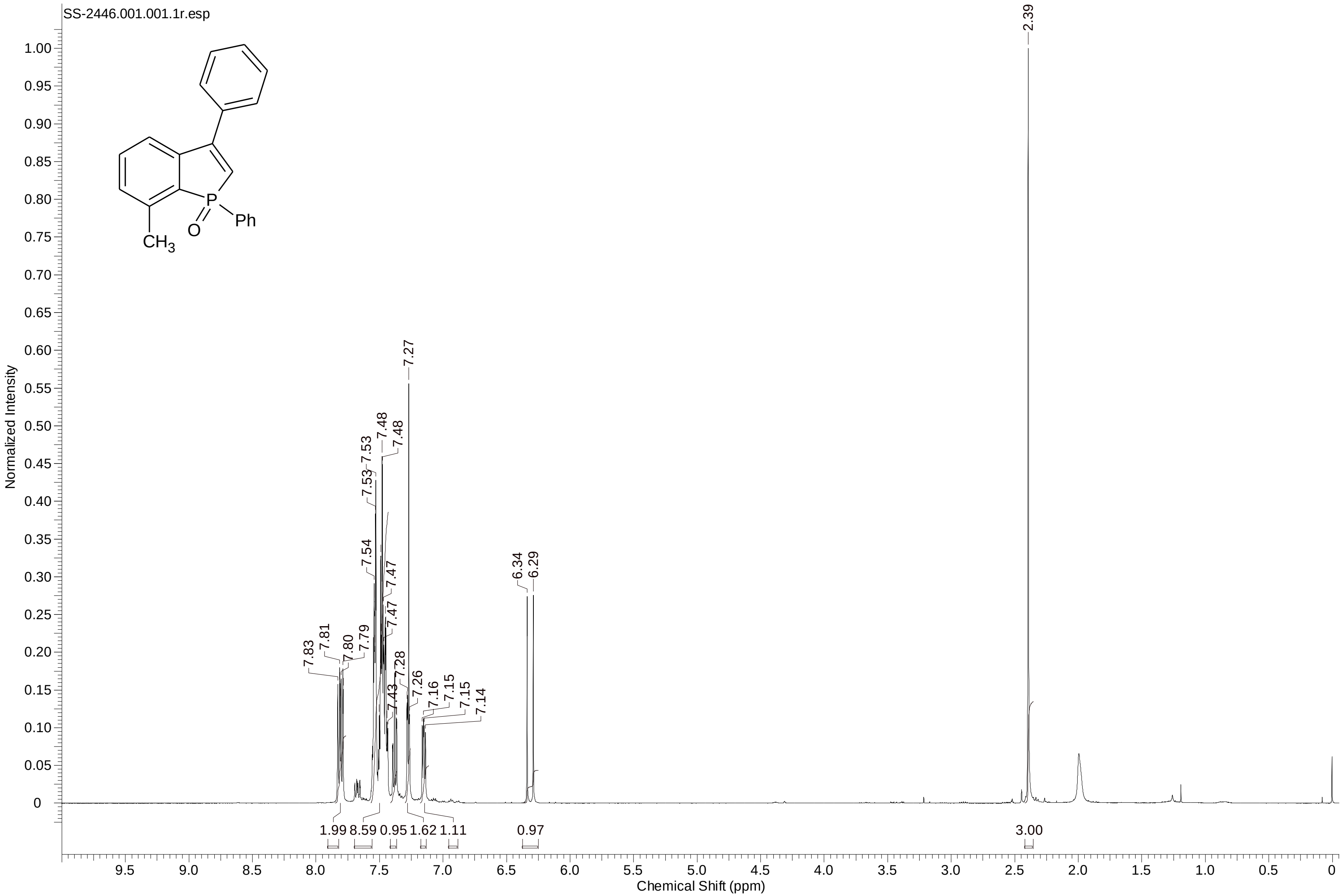

^{1}H NMR spectrum of 1,3-diphenyl-7-methylbenzophosphole oxide (**6a**) (500 MHz, $CDCl_3$)

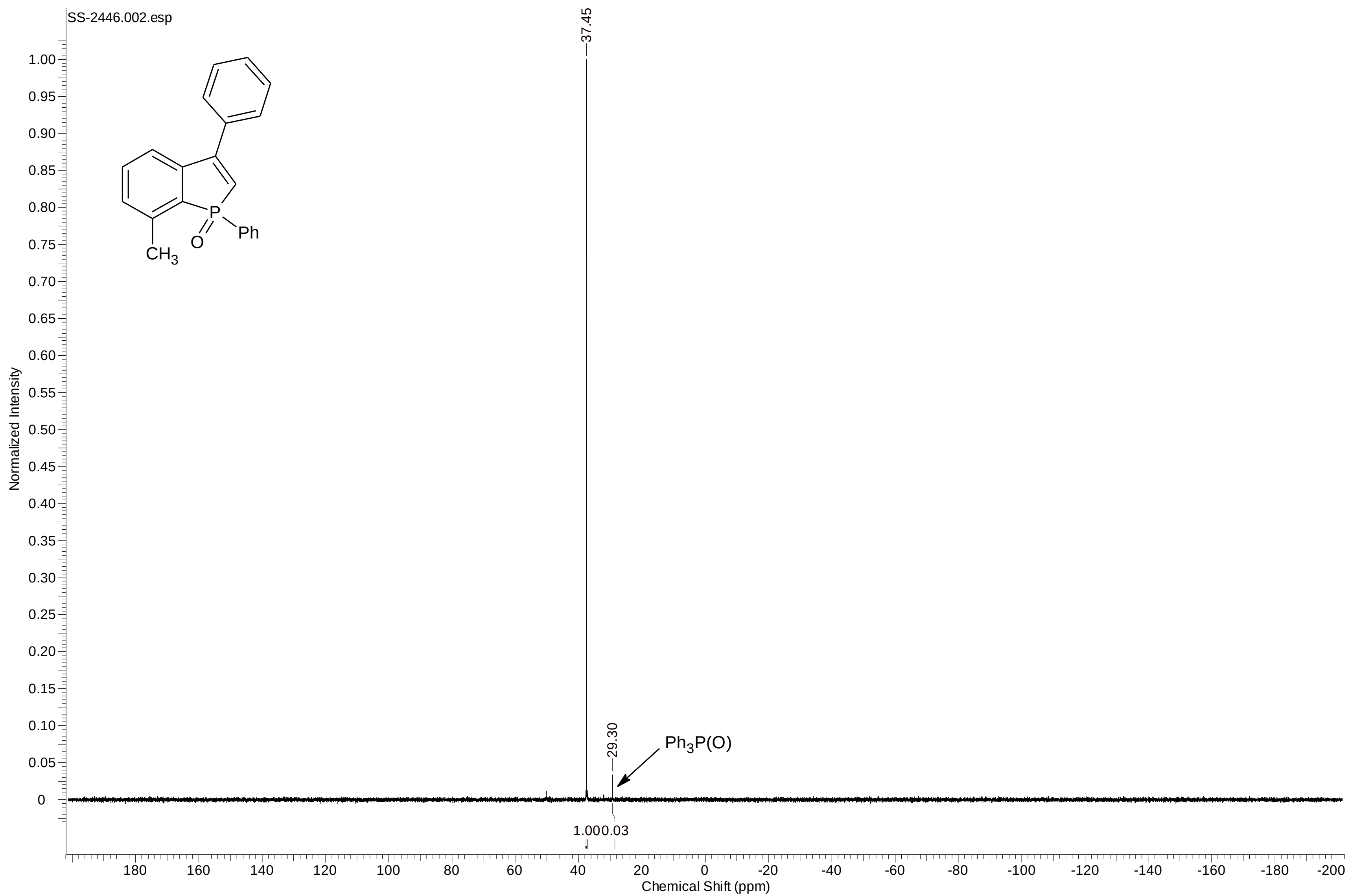

$^{31}P\{^1H\}$ NMR spectrum of 1,3-diphenyl-7-methylbenzophosphole oxide (**6a**) (202 MHz, $CDCl_3$)

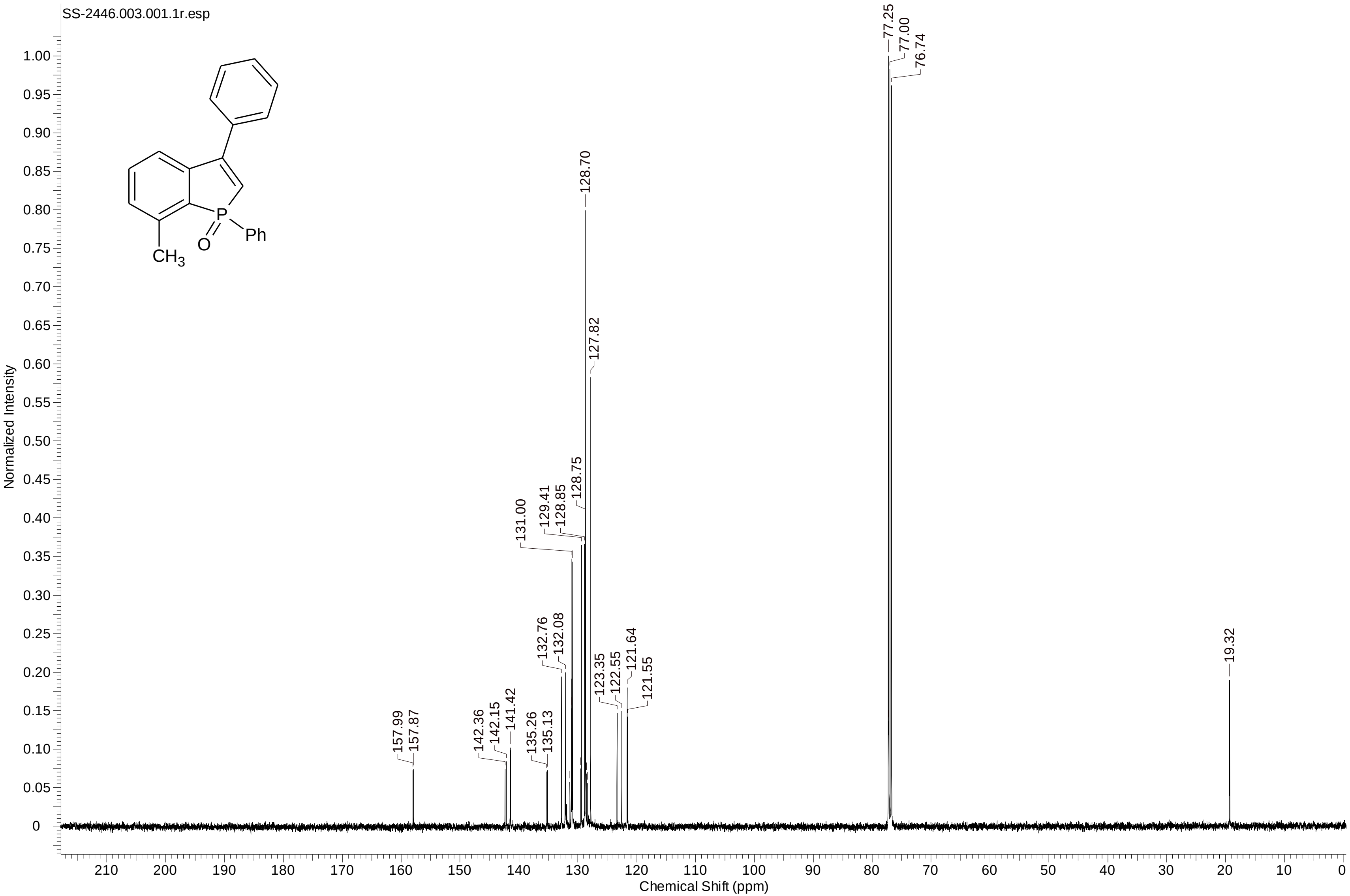

$^{13}C\{^{1}H\}$ NMR spectrum of 1,3-diphenyl-7-methylbenzophosphole oxide (**6a**) (125 MHz, $CDCl_3$)

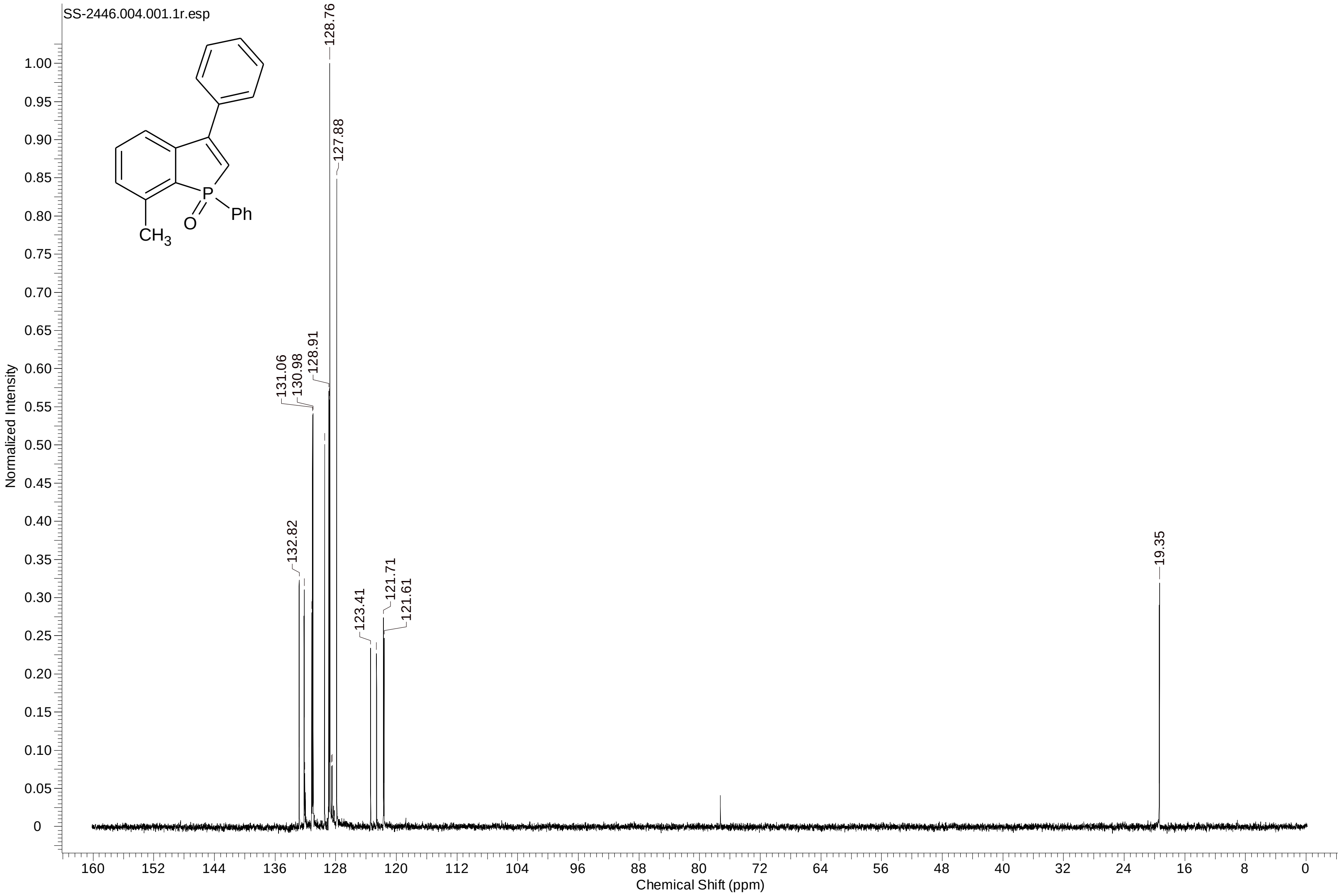

DEPT 135 NMR spectrum of 1,3-diphenyl-7-methylbenzophosphole oxide (**6a**) (125 MHz, $CDCl_3$)

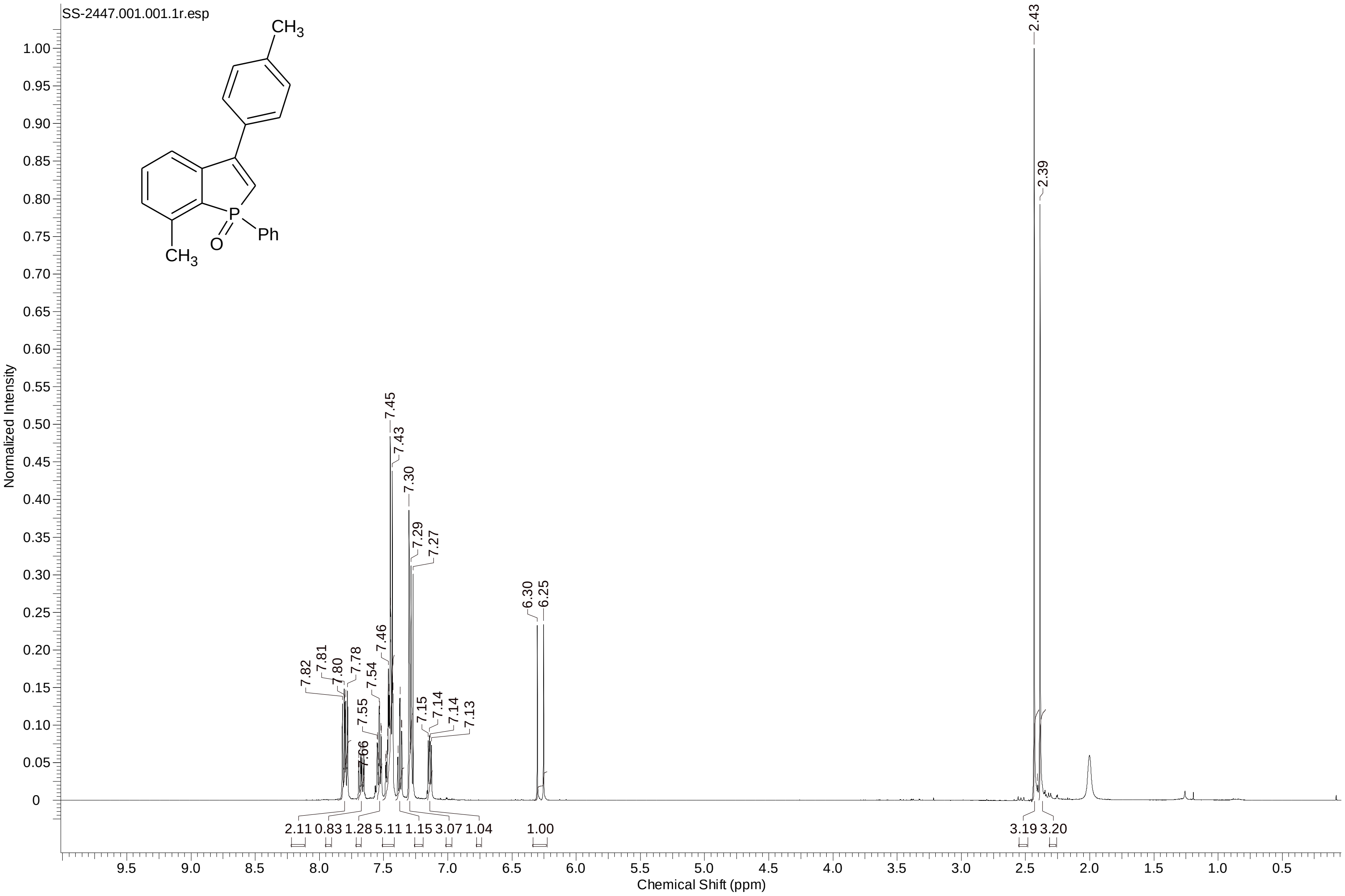

^{1}H NMR spectrum of 7-methyl-1-phenyl-3-(*p*-tolyl)benzophosphole oxide (**6b**) (500 MHz, $CDCl_3$)

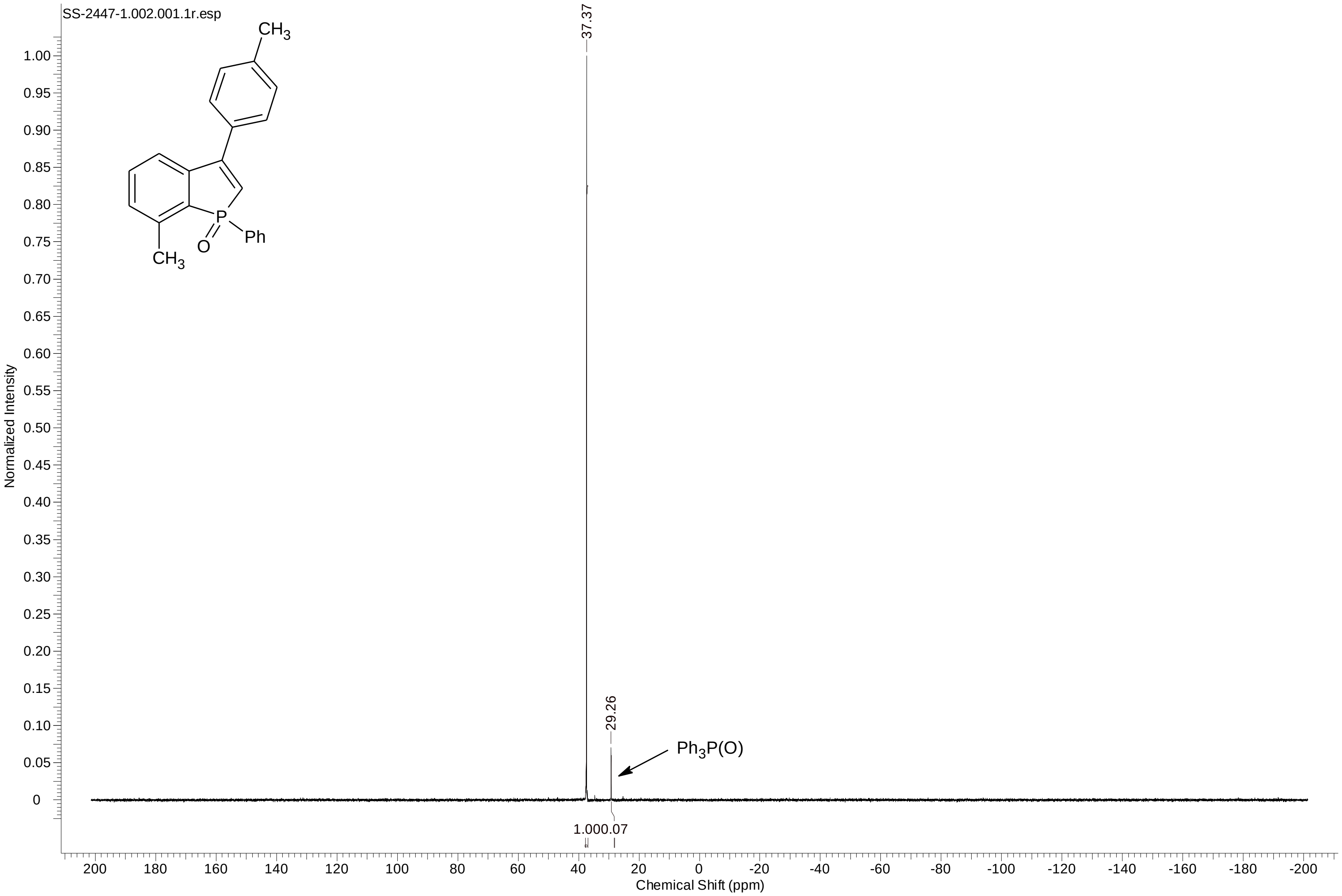

$^{31}P\{^{1}H\}$ NMR spectrum of 7-methyl-1-phenyl-3-(*p*-tolyl)benzophosphole oxide (**6b**) (202 MHz, $CDCl_3$)

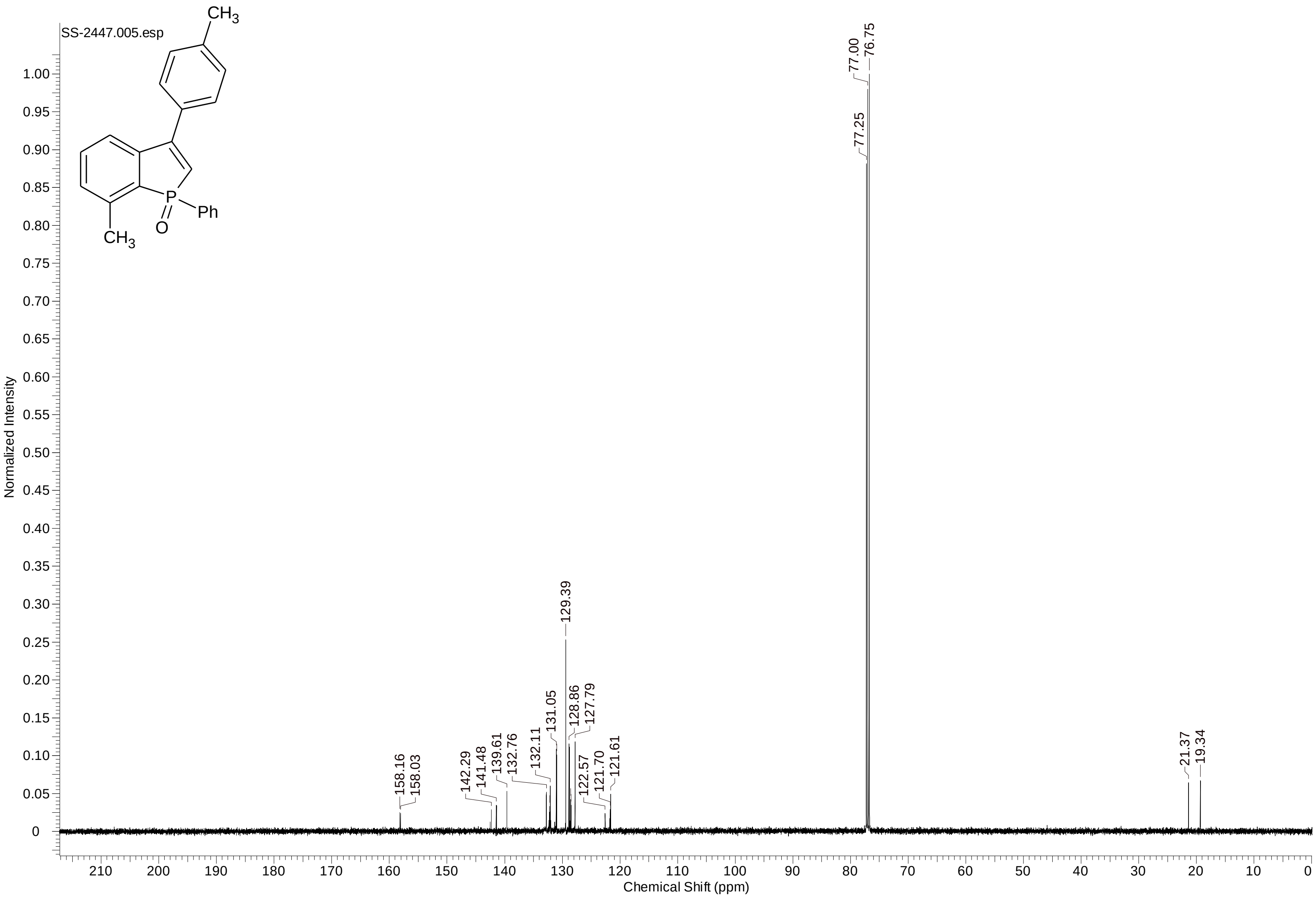

$^{13}C\{^1H\}$ NMR spectrum of 7-methyl-1-phenyl-3-(*p*-tolyl)benzophosphole oxide (**6b**) (125 MHz, $CDCl_3$)

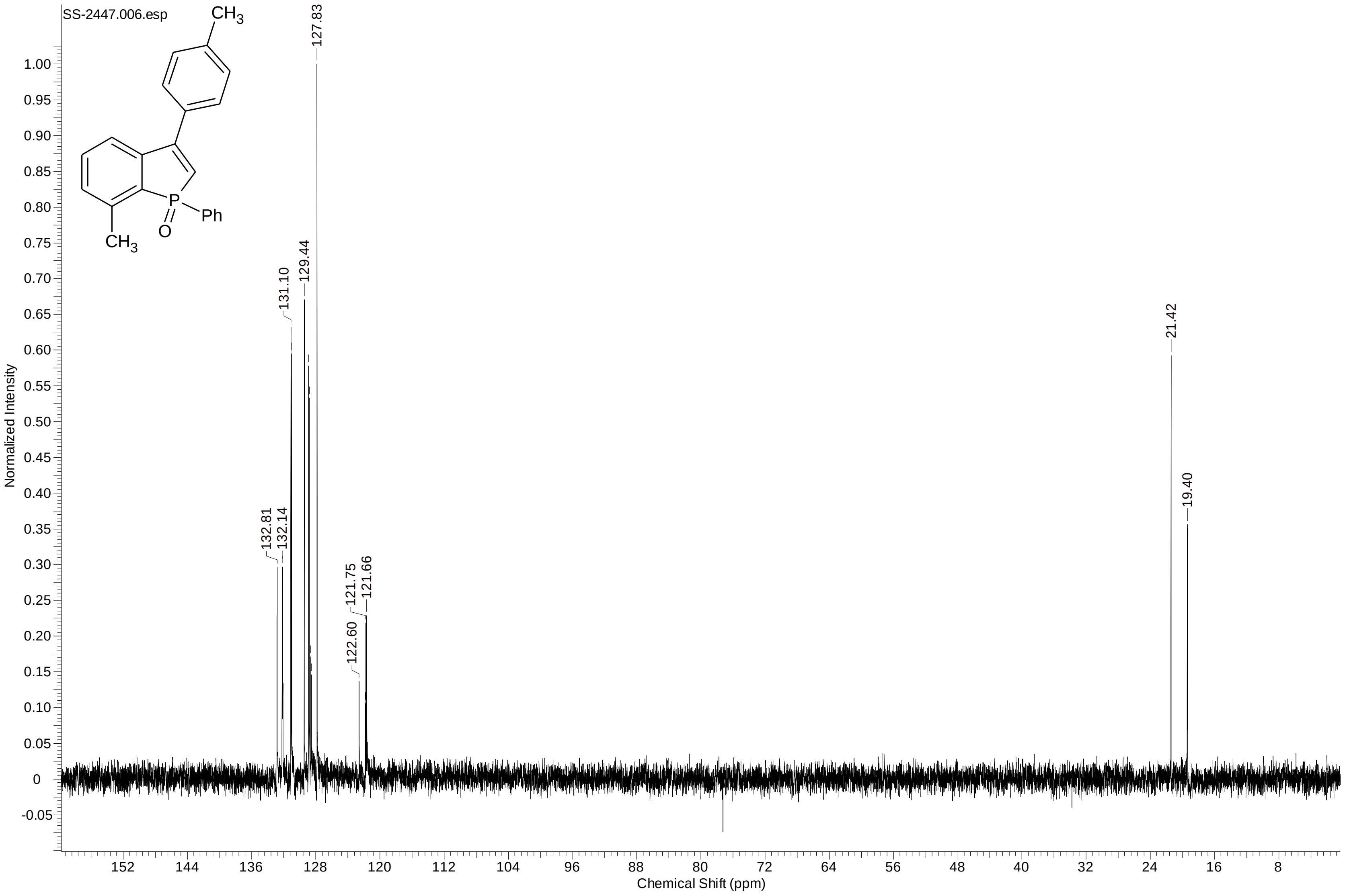

DEPT 135 NMR spectrum of 7-methyl-1-phenyl-3-(*p*-tolyl)benzophosphole oxide (**6b**) (125 MHz, $CDCl_3$)

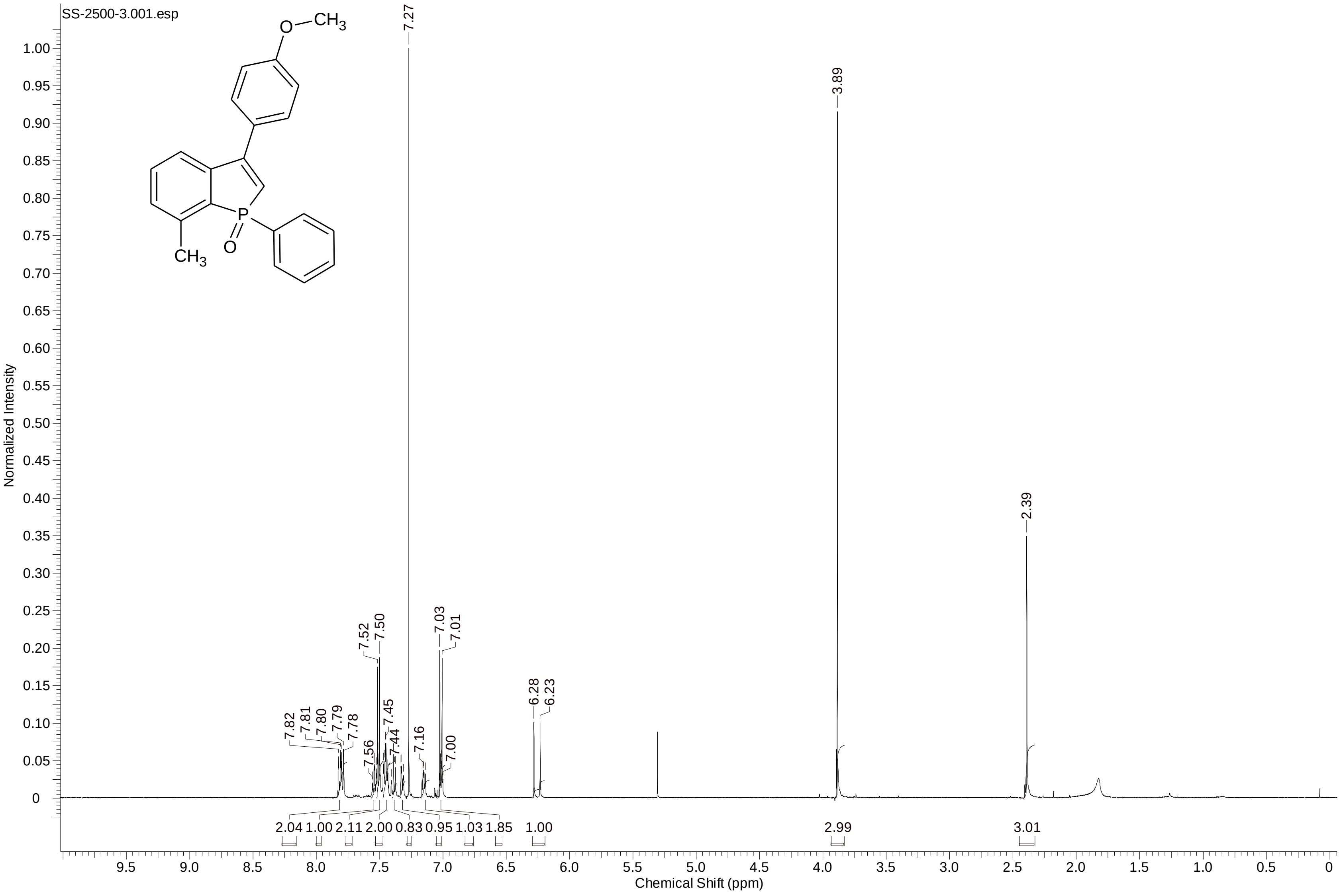

^{1}H NMR spectrum of 1-phenyl-3-(*p*-anisyl)benzophosphole oxide (**6e**) (500 MHz, $CDCl_3$)

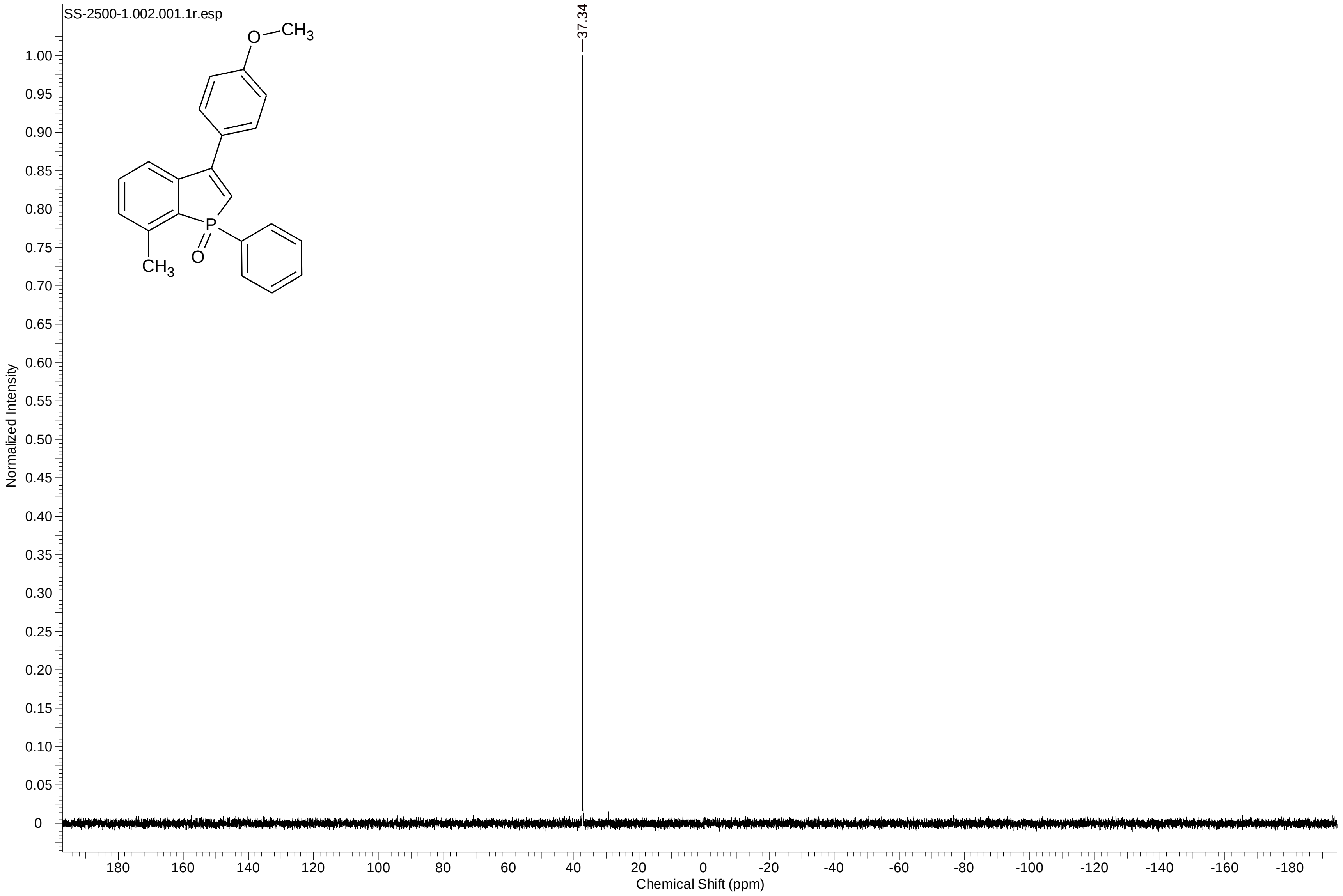

$^{31}P\{^{1}H\}$ NMR spectrum of 1-phenyl-3-(*p*-anisyl)benzophosphole oxide (**6e**) (202 MHz, $CDCl_3$)

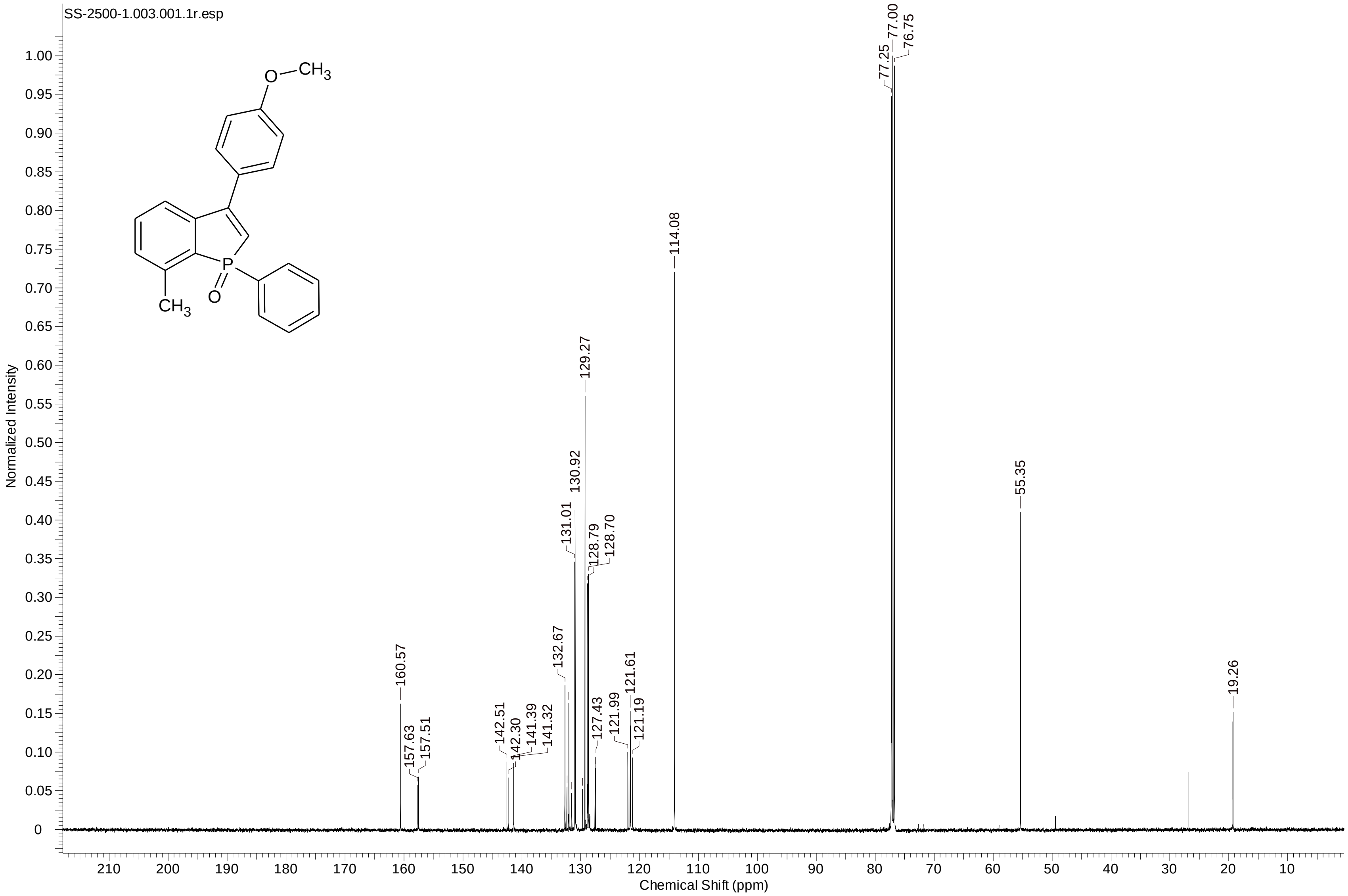

$^{13}C\{^{1}H\}$ NMR spectrum of 1-phenyl-3-(*p*-anisyl)benzophosphole oxide (**6e**) (125 MHz, $CDCl_3$)

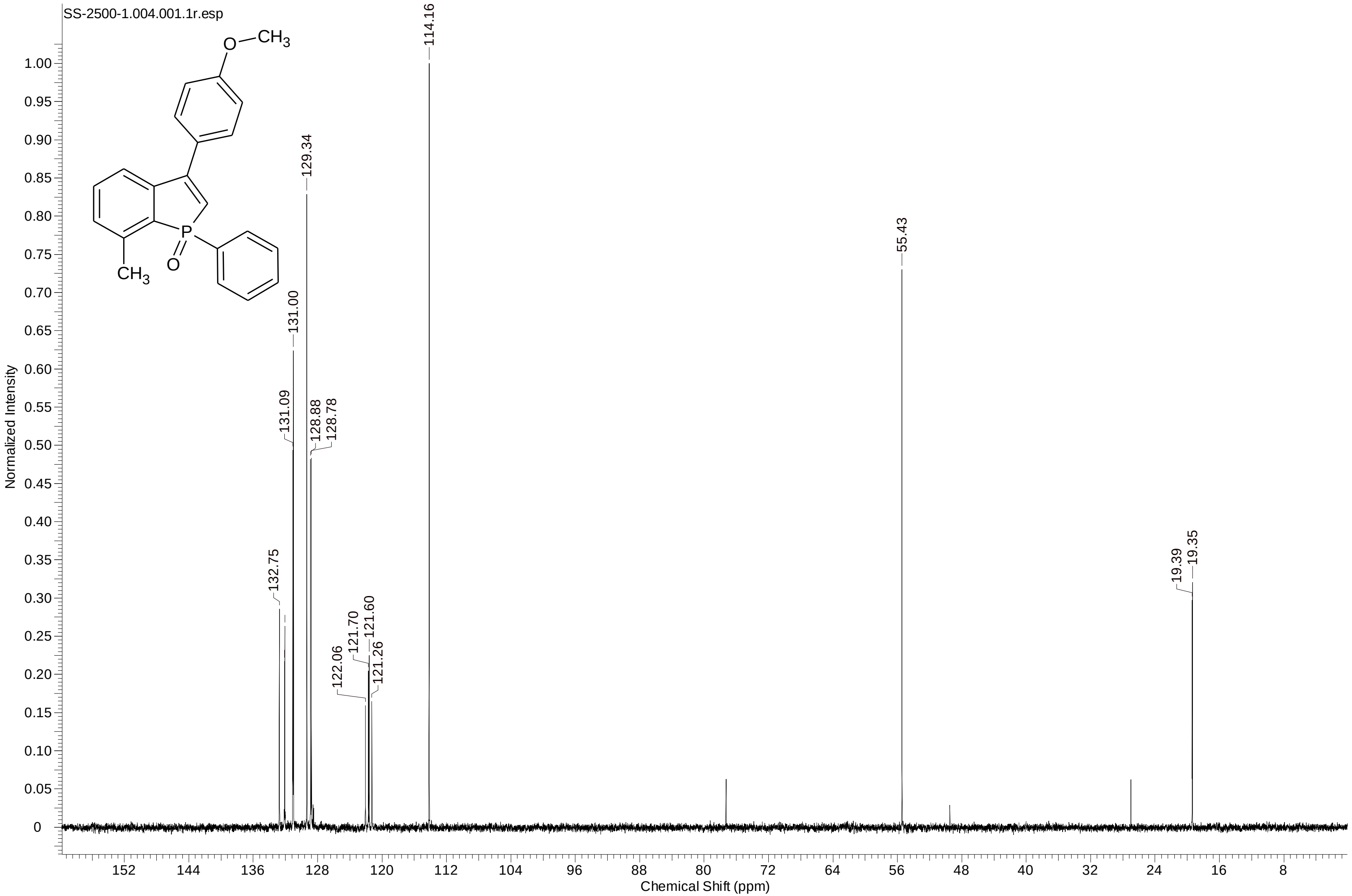

DEPT 135 NMR spectrum of 1-phenyl-3-(*p*-anisyl)benzophosphole oxide (**6e**) (125 MHz, $CDCl_3$)

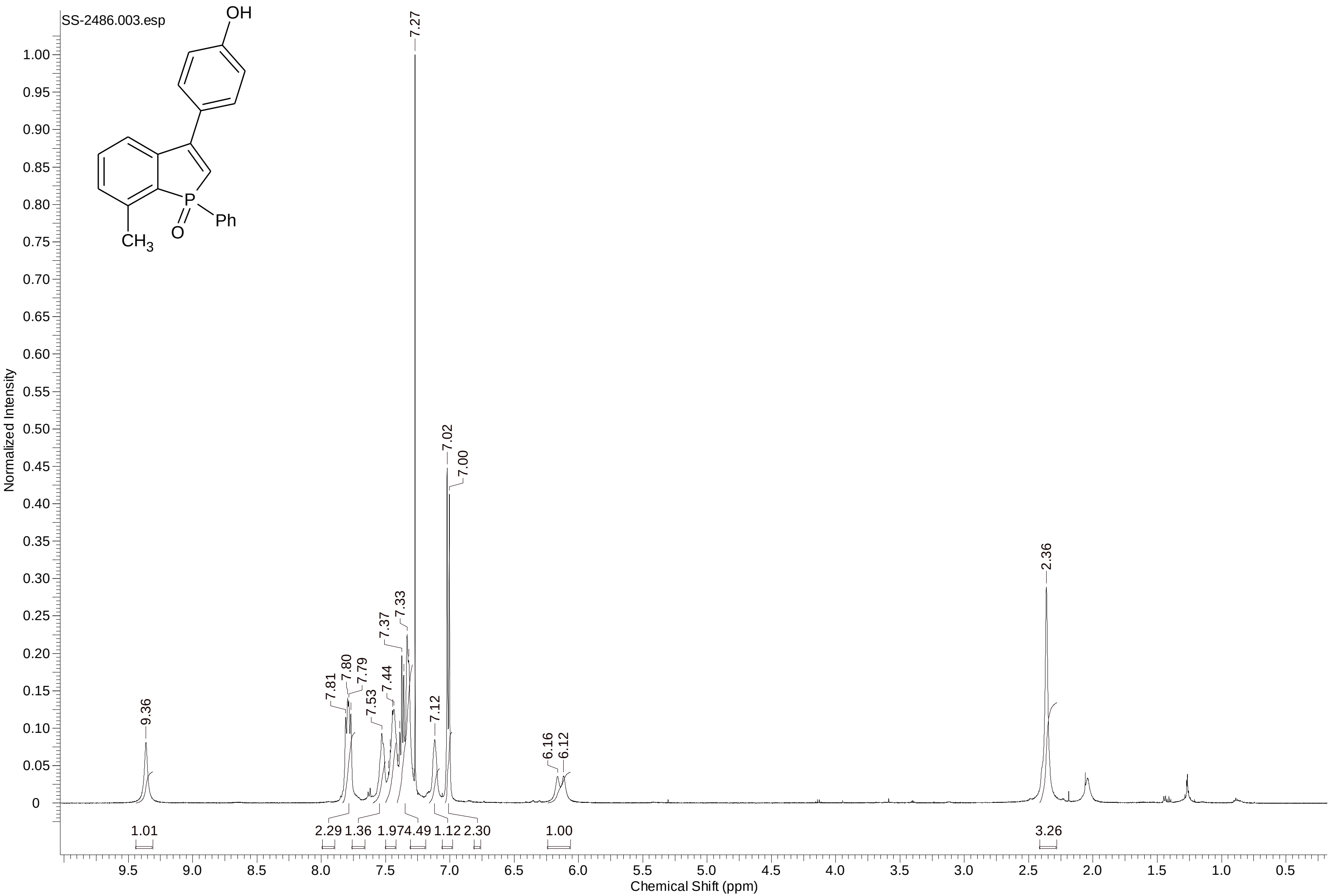

^{1}H NMR spectrum of 3-(*p*-hydroxyphenyl)-7-methyl-1-phenylbenzophosphole oxide (**6h**) (500 MHz, $CDCl_3$)

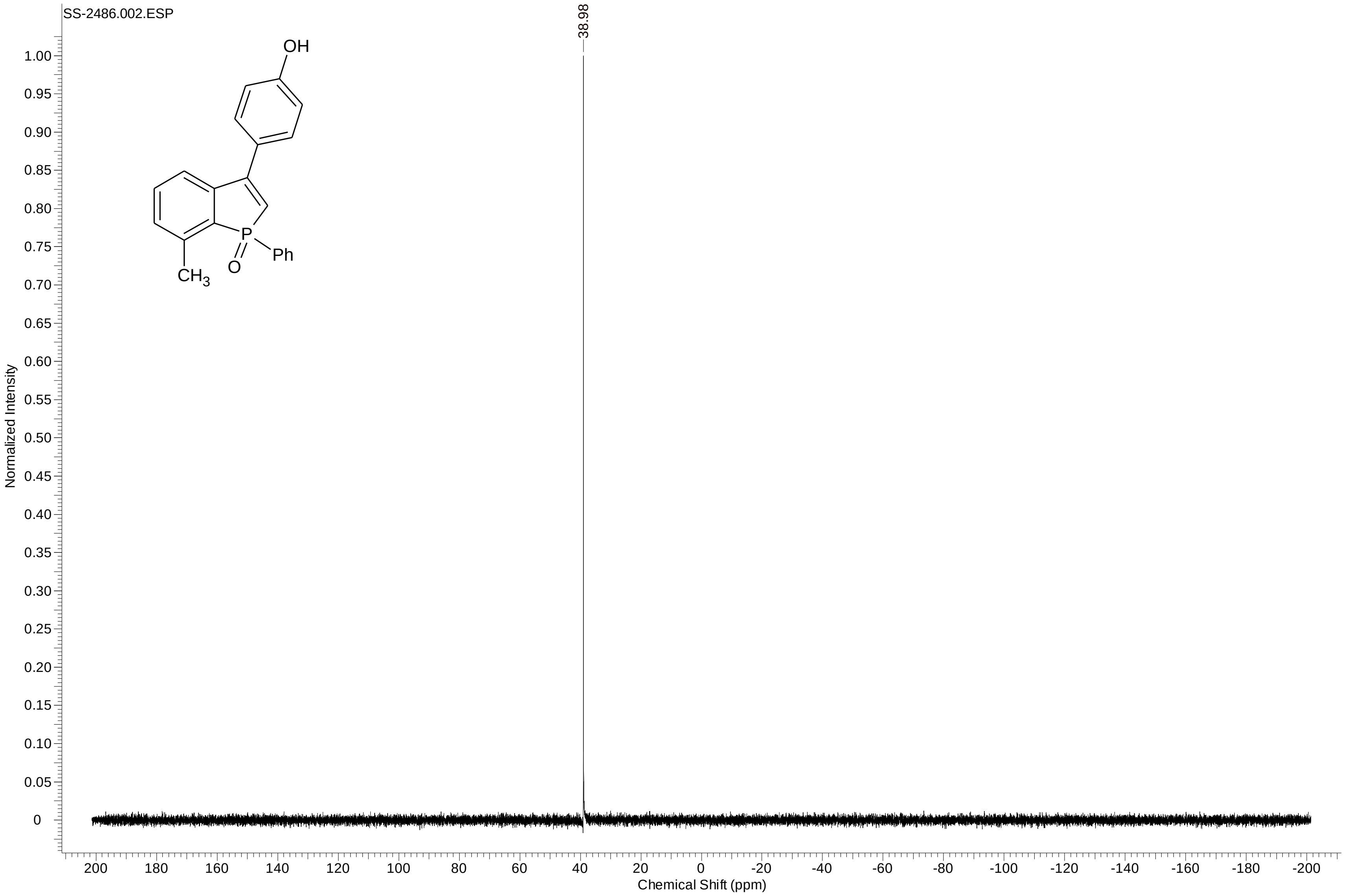

$^{31}P\{^1H\}$ NMR spectrum of 3-(*p*-hydroxyphenyl)-7-methyl-1-phenylbenzophosphole oxide (**6h**) (202 MHz, $CDCl_3$)

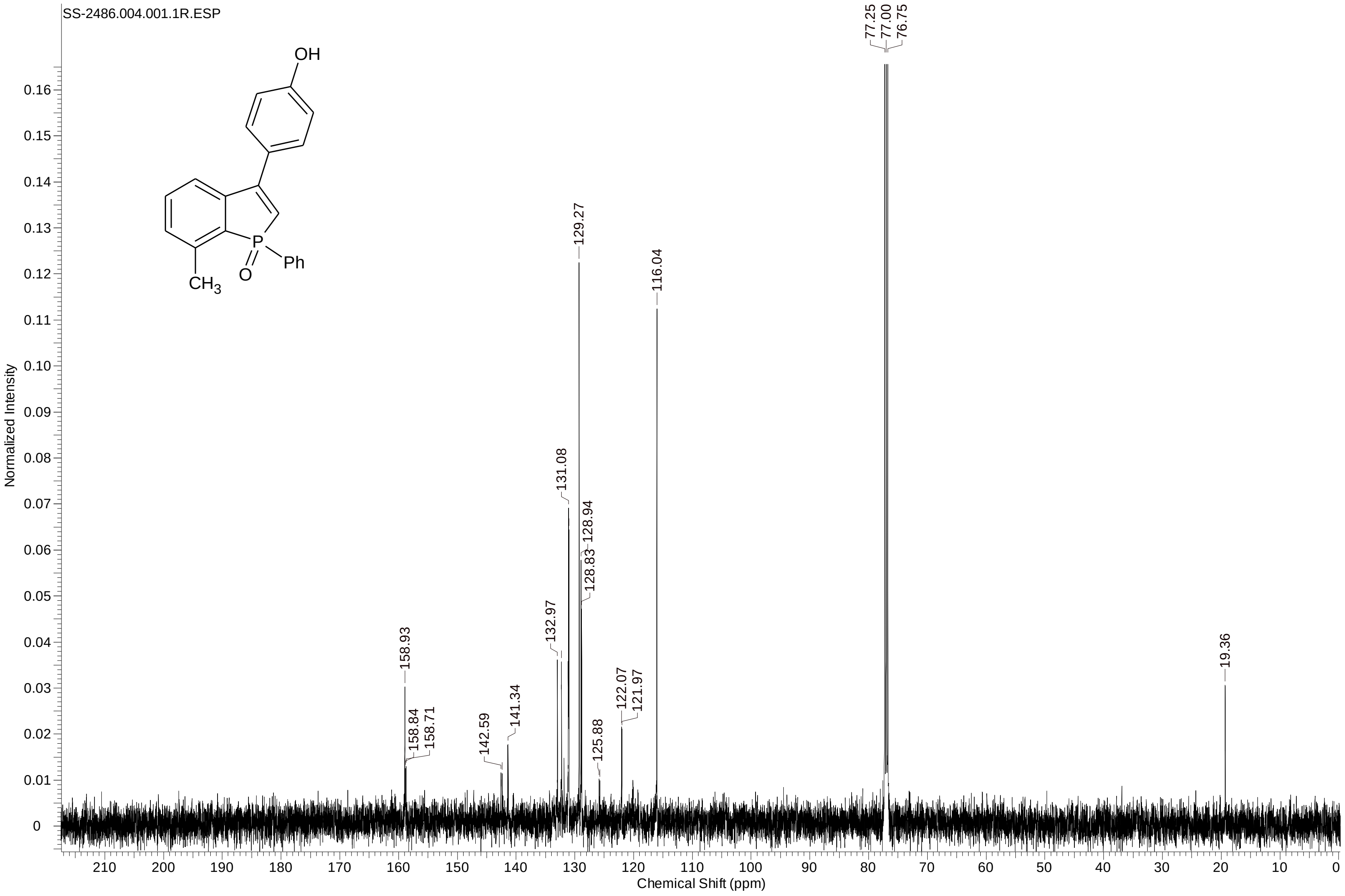

$^{13}C\{^{1}H\}$ NMR spectrum of 3-(*p*-hydroxyphenyl)-7-methyl-1-phenylbenzophosphole oxide (**6h**) (202 MHz, $CDCl_3$) (low solubility in $CDCl_3$)

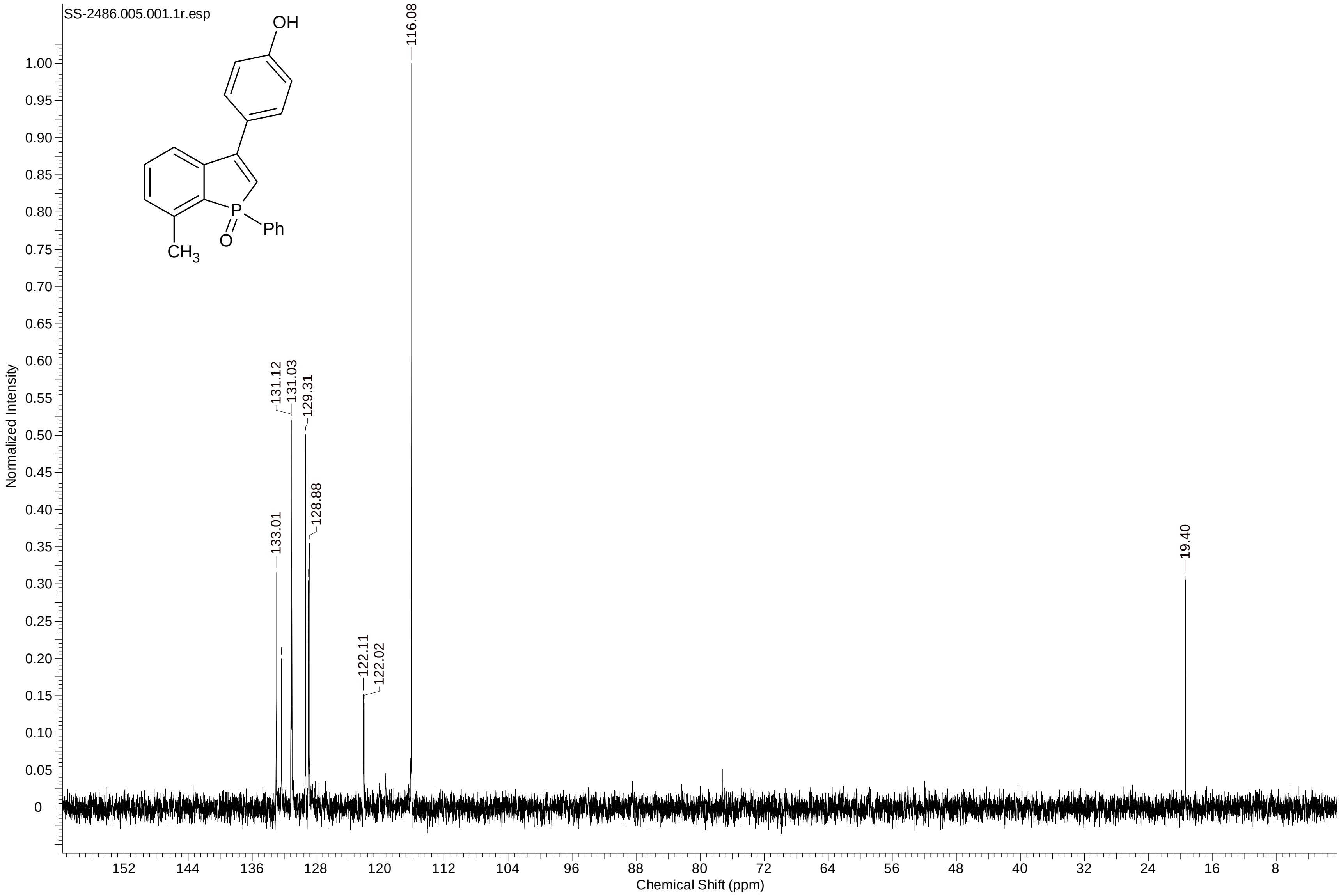

DEPT 135 NMR spectrum of 3-(*p*-hydroxyphenyl)-7-methyl-1-phenylbenzophosphole oxide (**6h**) (125 MHz, $CDCl_3$)

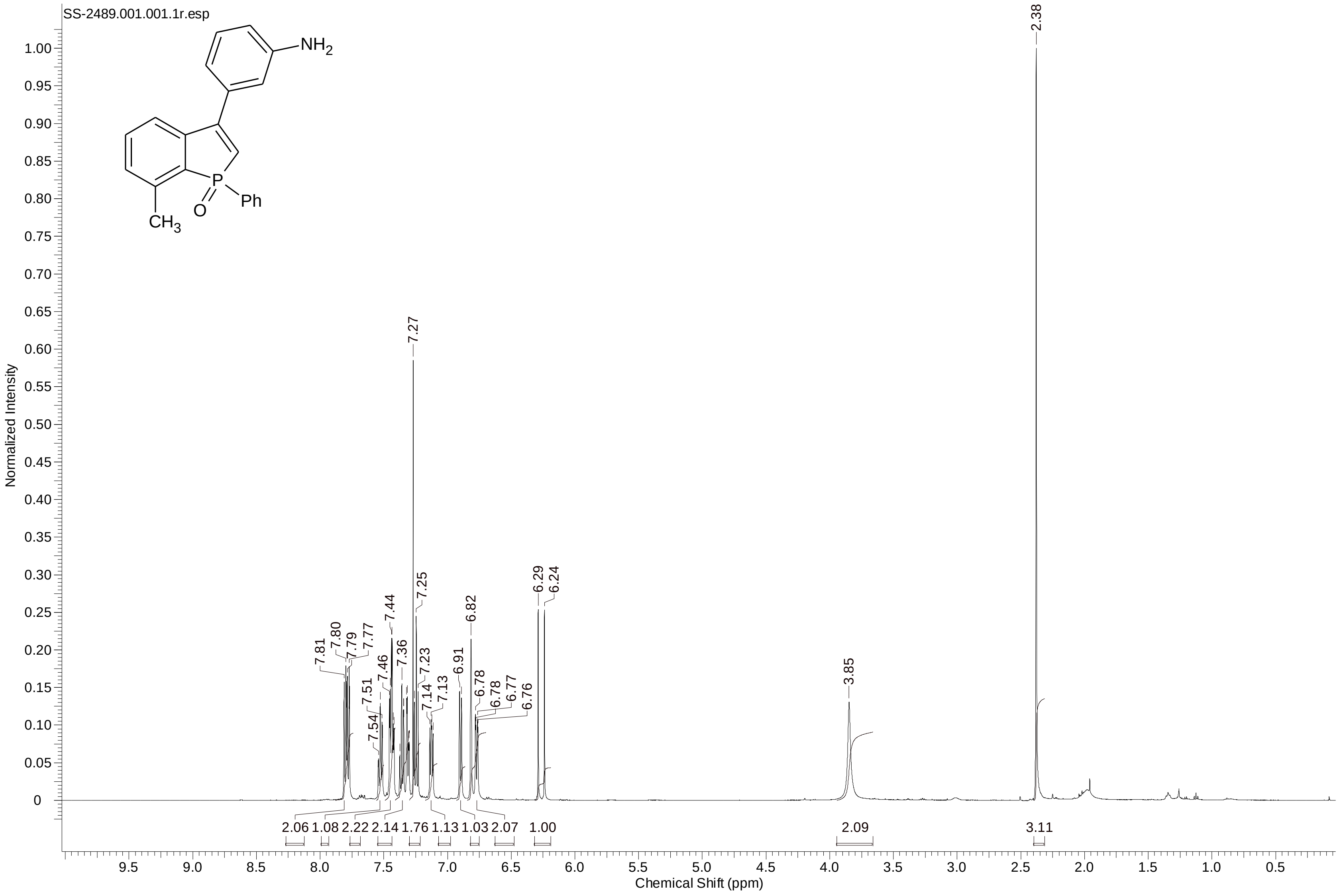

^{1}H NMR spectrum of 3-(*p*-aminophenyl)-7-methyl-1-phenylbenzophosphole oxide (**6i**) (500 MHz, $CDCl_3$)

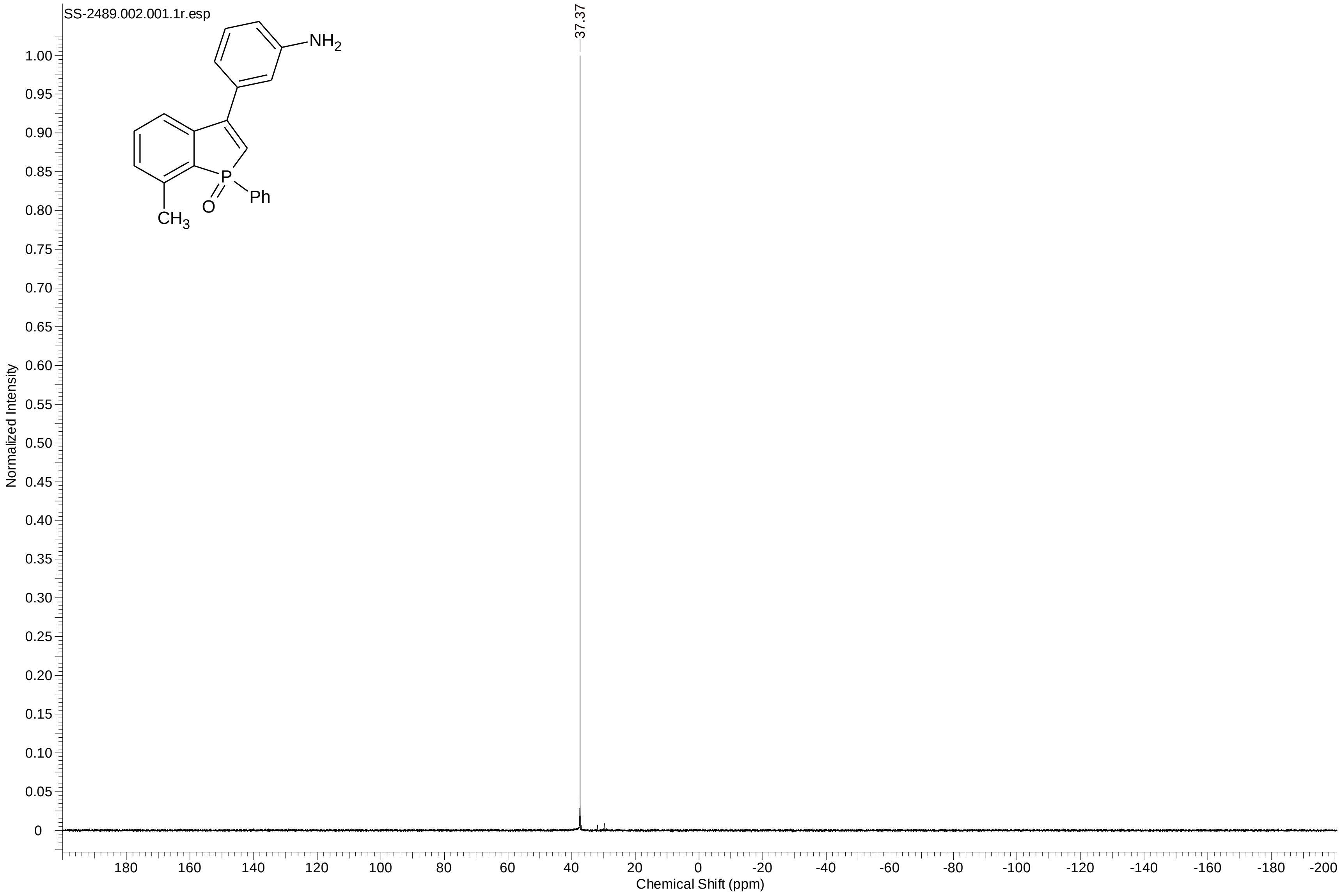

$^{31}P\{^1H\}$ NMR spectrum of 3-(*p*-aminophenyl)-7-methyl-1-phenylbenzophosphole oxide (**6i**) (202 MHz, $CDCl_3$)

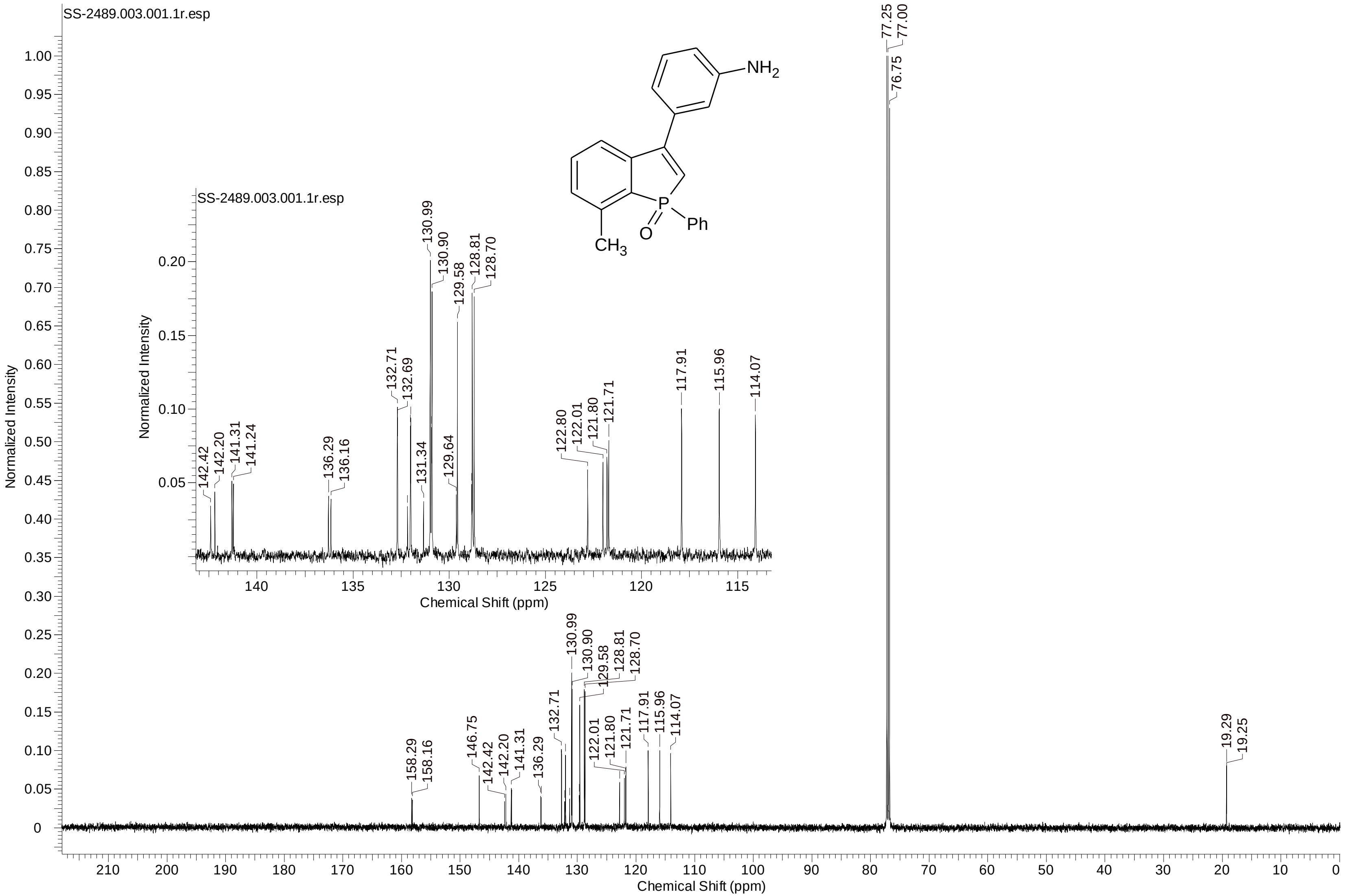

$^{13}C\{^{1}H\}$ NMR spectrum of 3-(*p*-aminophenyl)-7-methyl-1-phenylbenzophosphole oxide (**6i**) (202 MHz, $CDCl_3$)

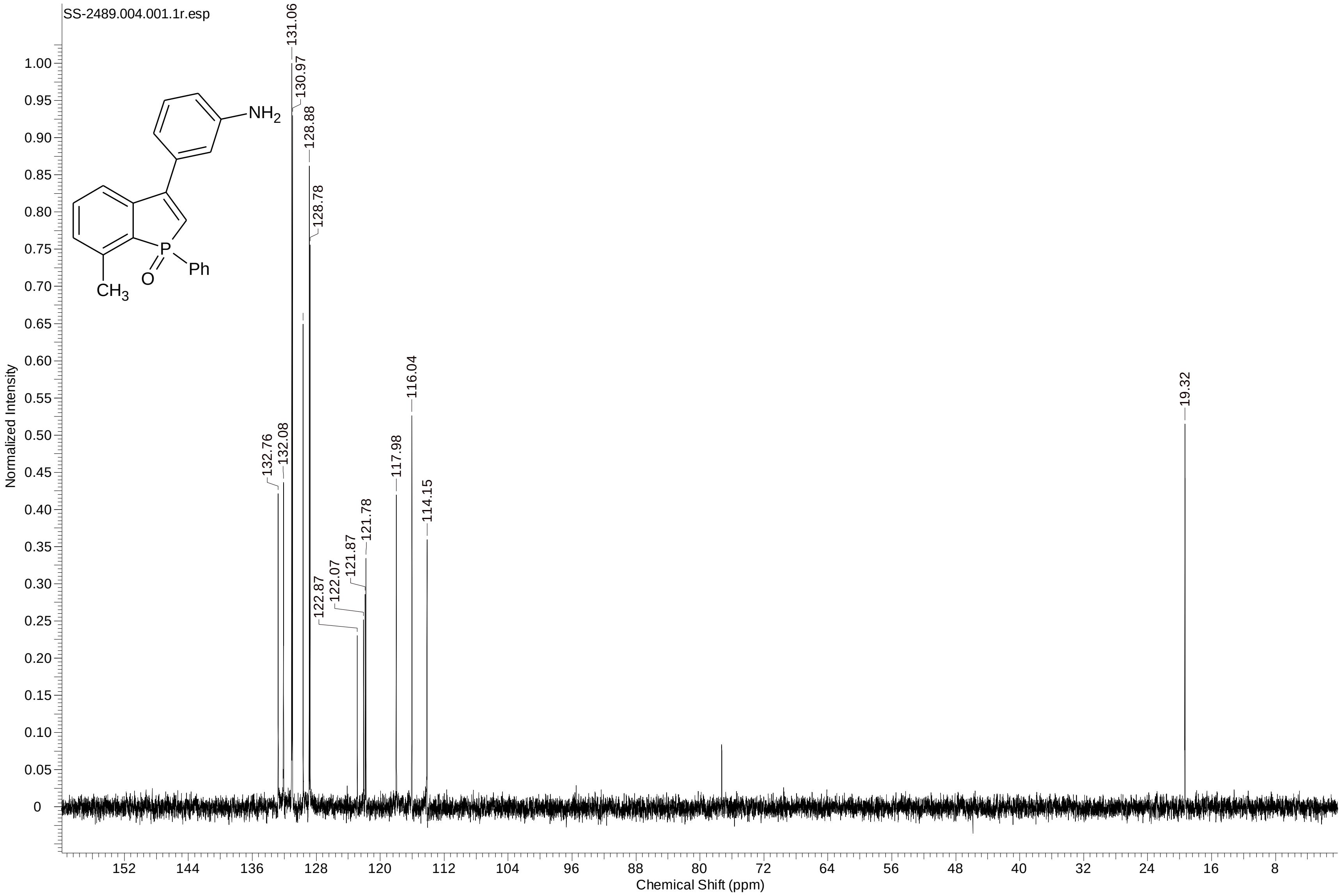

DEPT 135 NMR spectrum of 3-(*p*-aminophenyl)-7-methyl-1-phenylbenzophosphole oxide (**6i**) (202 MHz, $CDCl_3$)

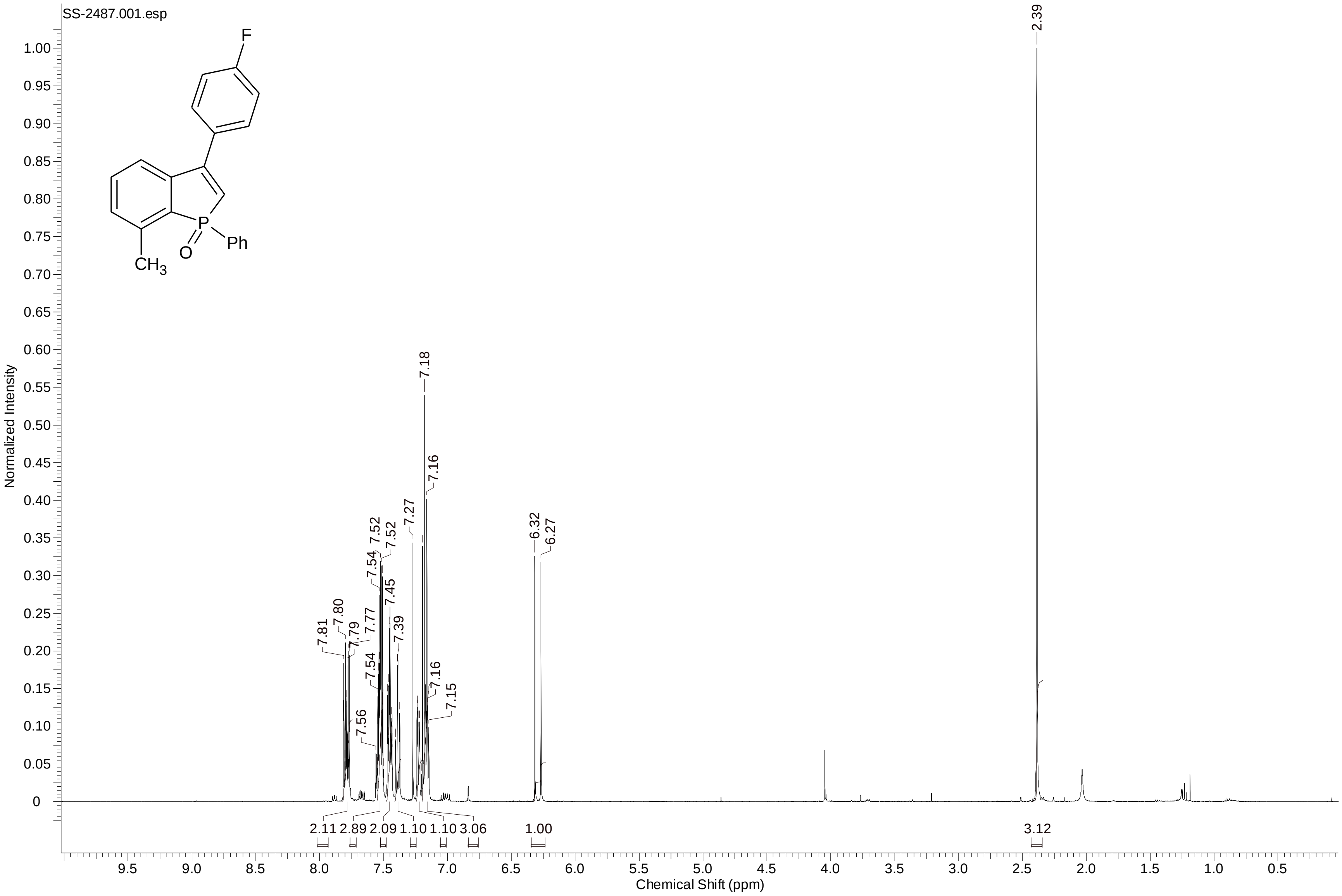

^{1}H NMR spectrum of 3-(*p*-fluorophenyl)-7-methyl-1-phenylbenzophosphole oxide (**6j**) (500 MHz, $CDCl_3$)

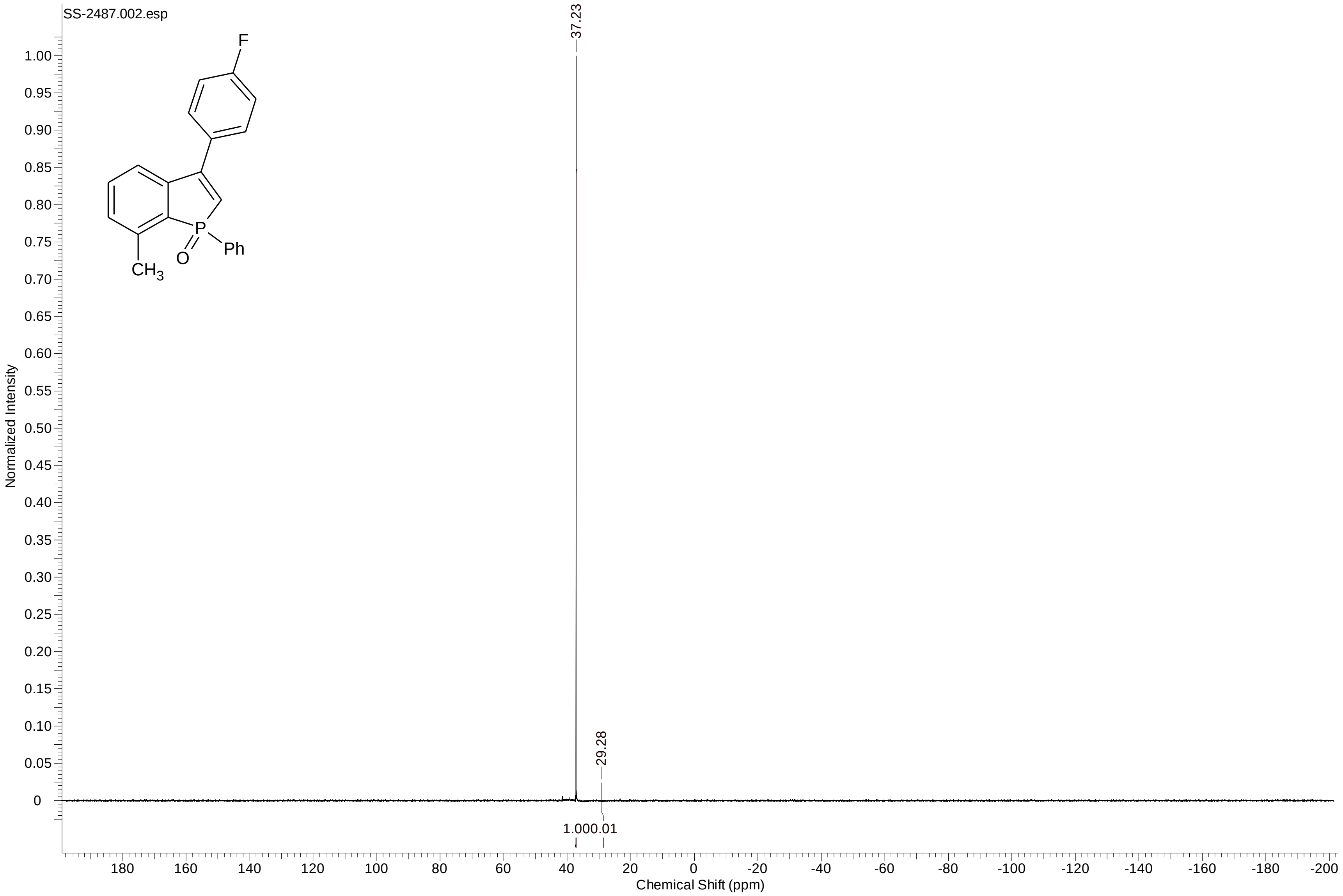

$^{31}P\{^{1}H\}$ NMR spectrum of 3-(*p*-fluorophenyl)-7-methyl-1-phenylbenzophosphole oxide (**6j**) (202 MHz, $CDCl_3$)

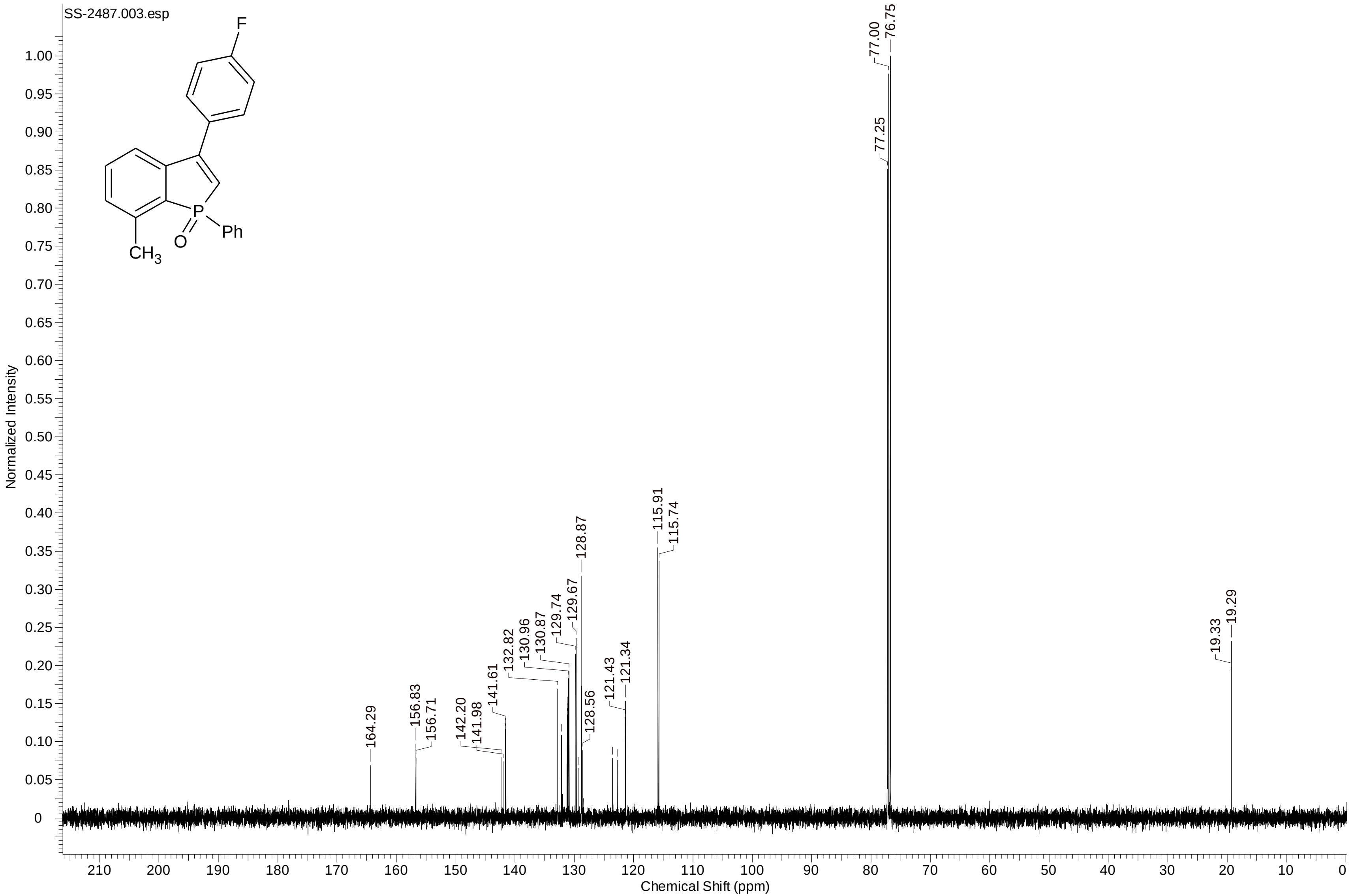

$^{13}C\{^{1}H\}$ NMR spectrum of 3-(*p*-fluorophenyl)-7-methyl-1-phenylbenzophosphole oxide (**6j**) (125 MHz, $CDCl_3$)

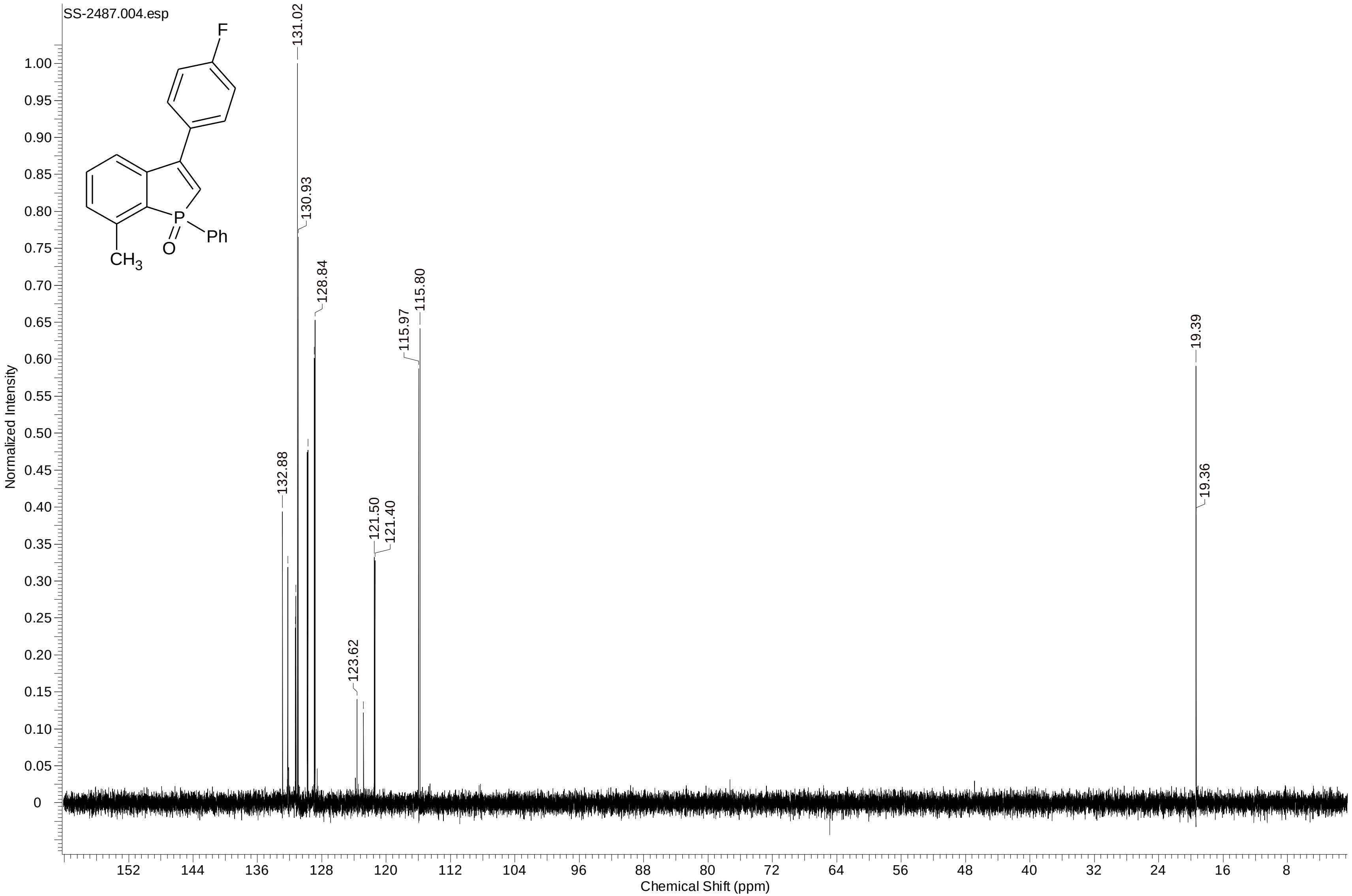

DEPT 135 NMR spectrum of 3-(*p*-fluorophenyl)-7-methyl-1-phenylbenzophosphole oxide (**6j**) (125 MHz, $CDCl_3$)

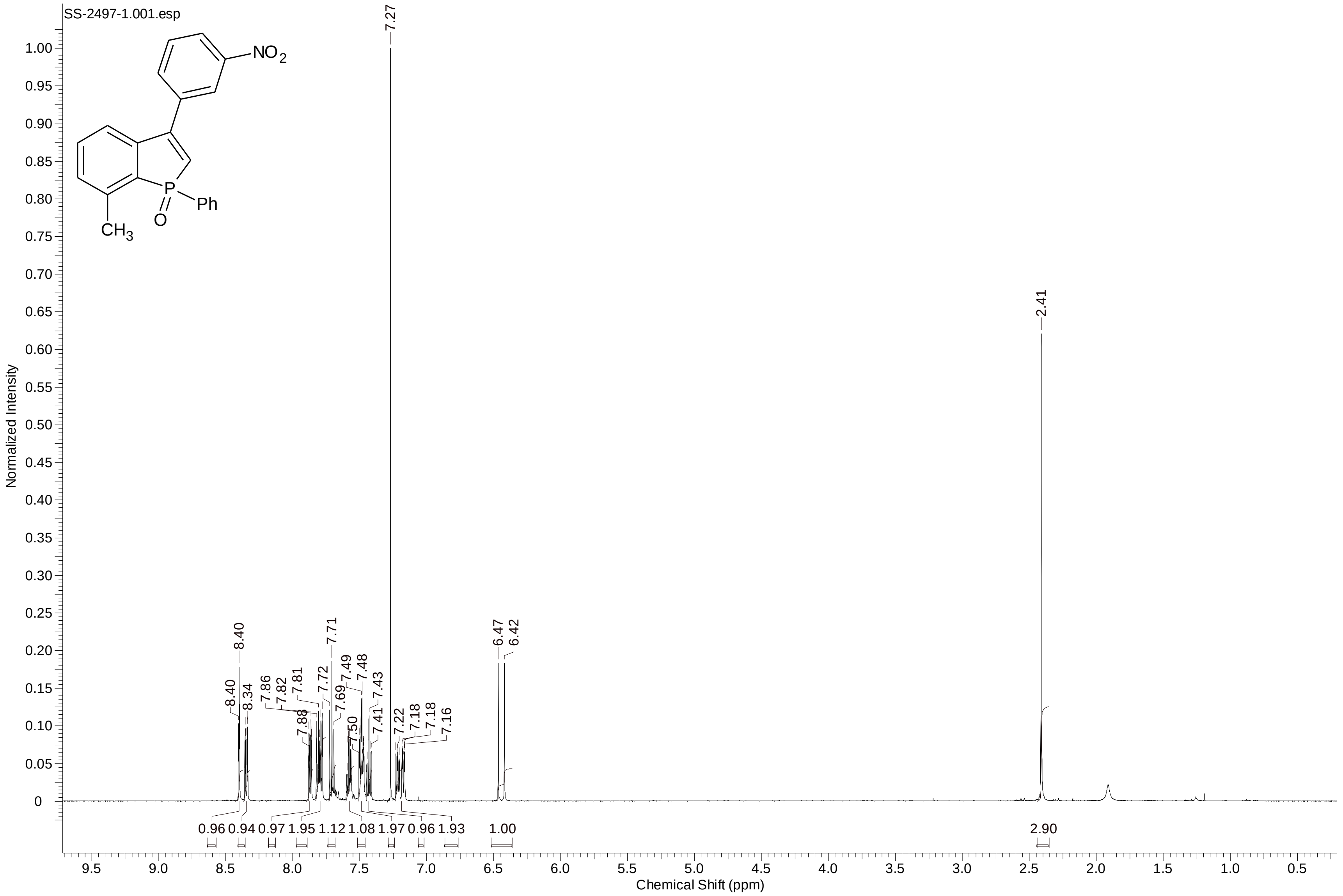

^{1}H NMR spectrum of 3-(*m*-nitrophenyl)-7-methyl-1-phenylbenzophosphole oxide (**6n**) (500 MHz, $CDCl_3$)

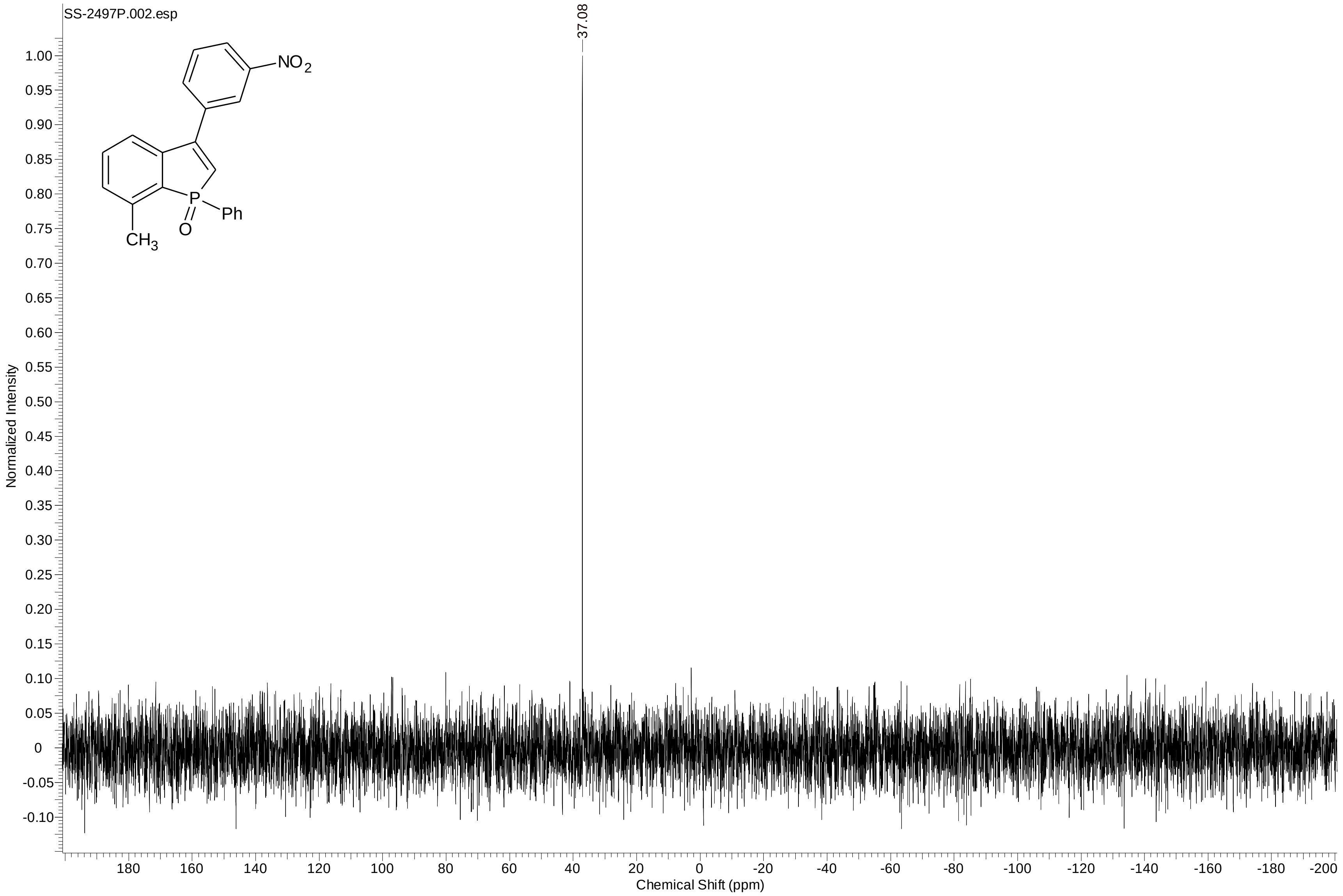

$^{31}P\{^1H\}$ NMR spectrum of 7-methyl-3-(*p*-nitrophenyl)-1-phenylbenzophosphole oxide (**6n**) (202 MHz, $CDCl_3$)

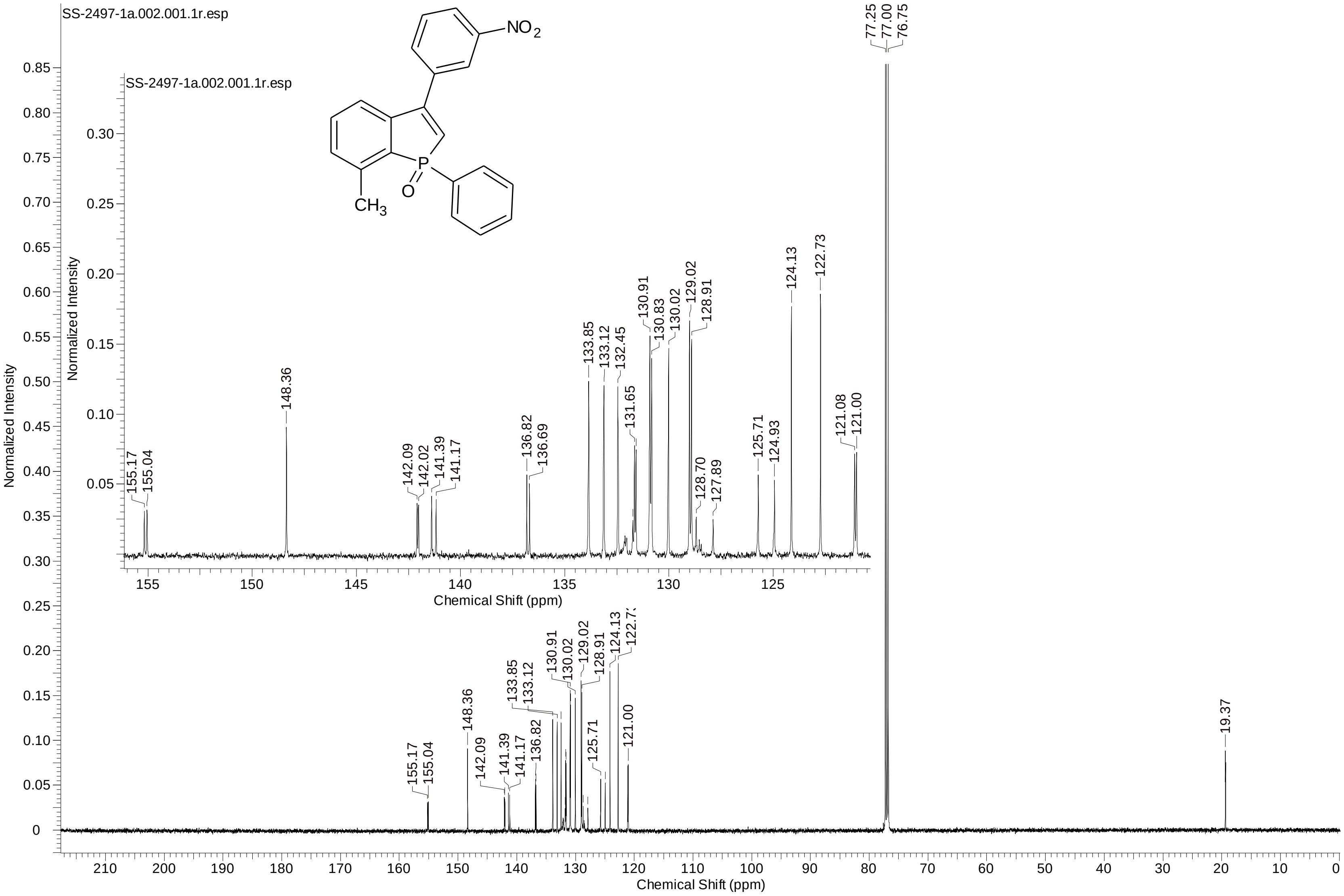

$^{13}C\{^1H\}$ NMR spectrum of 3-(*m*-nitrophenyl)-7-methyl-1-phenylbenzophosphole oxide (**6n**) (125 MHz, $CDCl_3$)

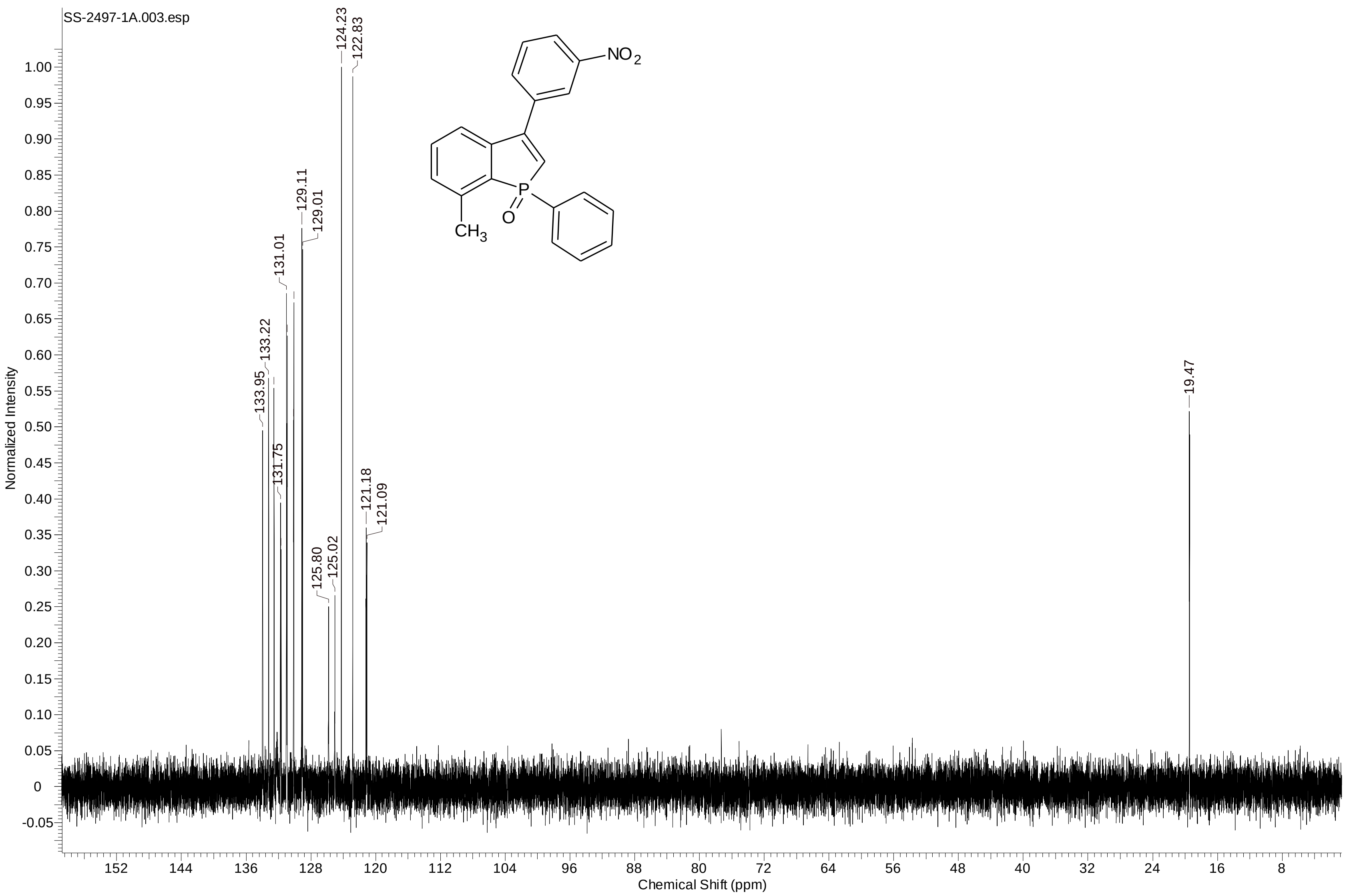

DEPT 135 NMR spectrum of 3-(*m*-nitrophenyl)-7-methyl-1-phenylbenzophosphole oxide (**6n**) (125 MHz, $CDCl_3$)

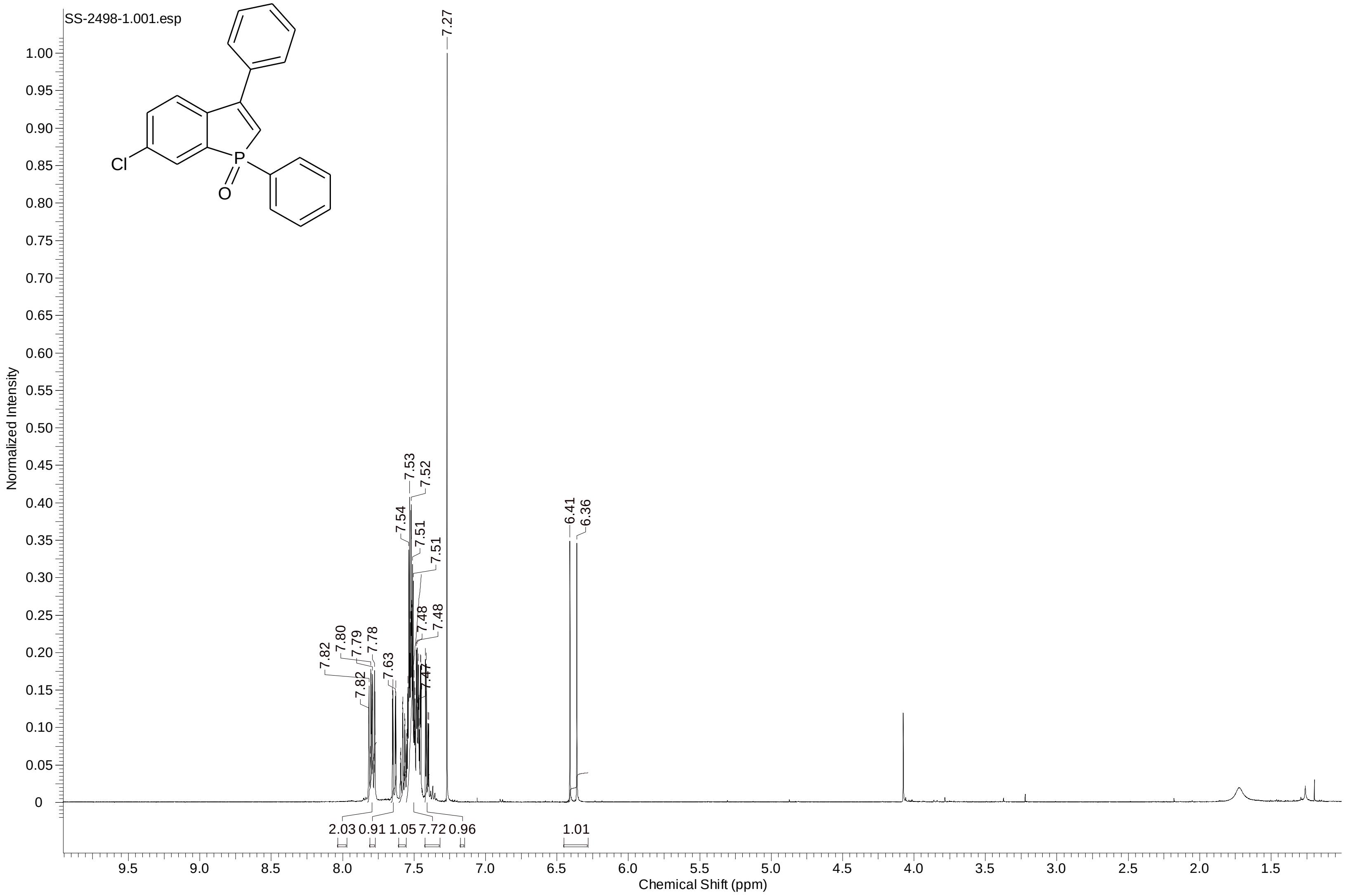

^{1}H NMR spectrum of 6-chloro-1,3-diphenylbenzophosphole oxide (**7a**) (500 MHz, $CDCl_3$)

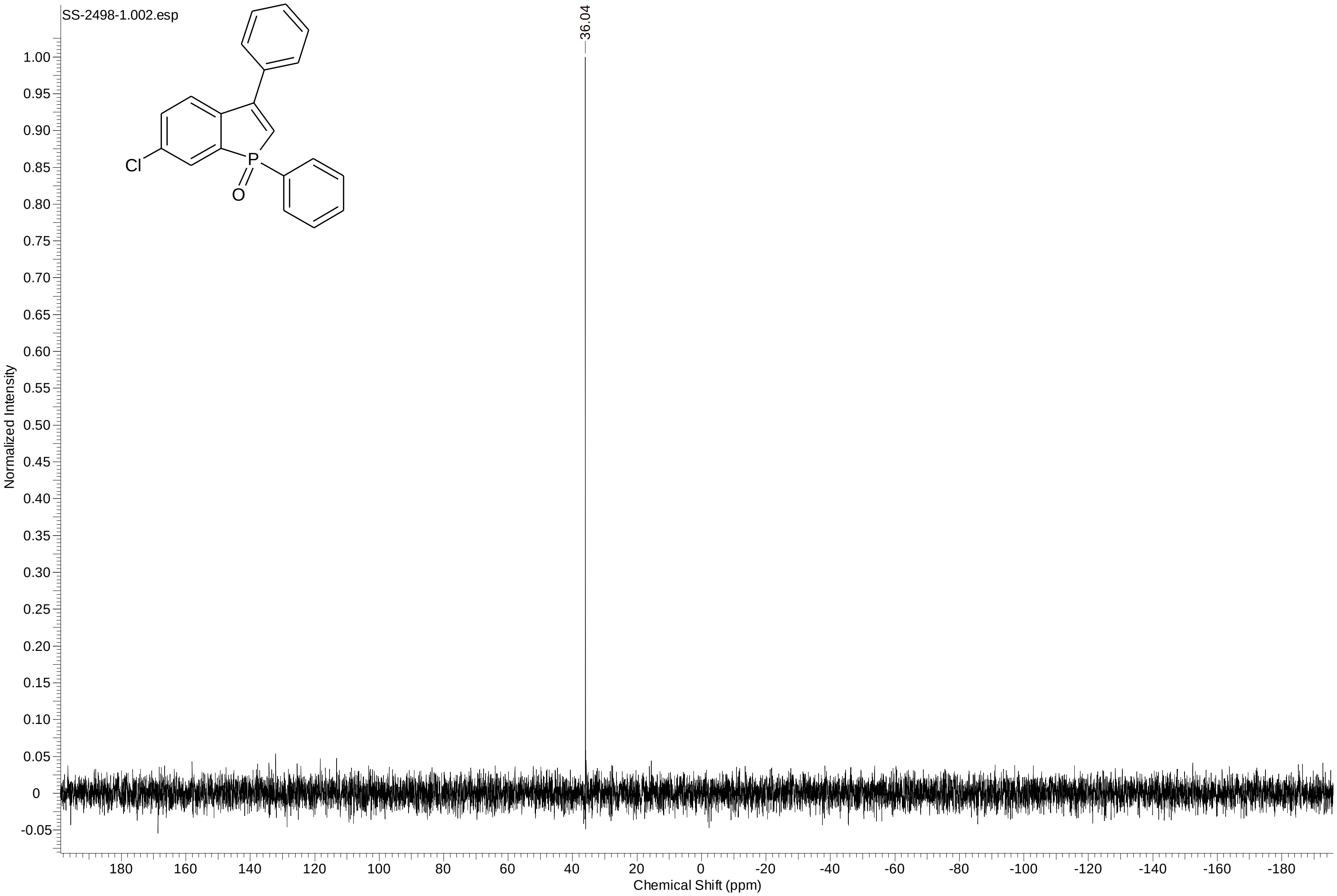

$^{31}P\{^{1}H\}$ NMR spectrum of 6-chloro-1,3-diphenylbenzophosphole oxide (**7a**) (202 MHz, $CDCl_3$)

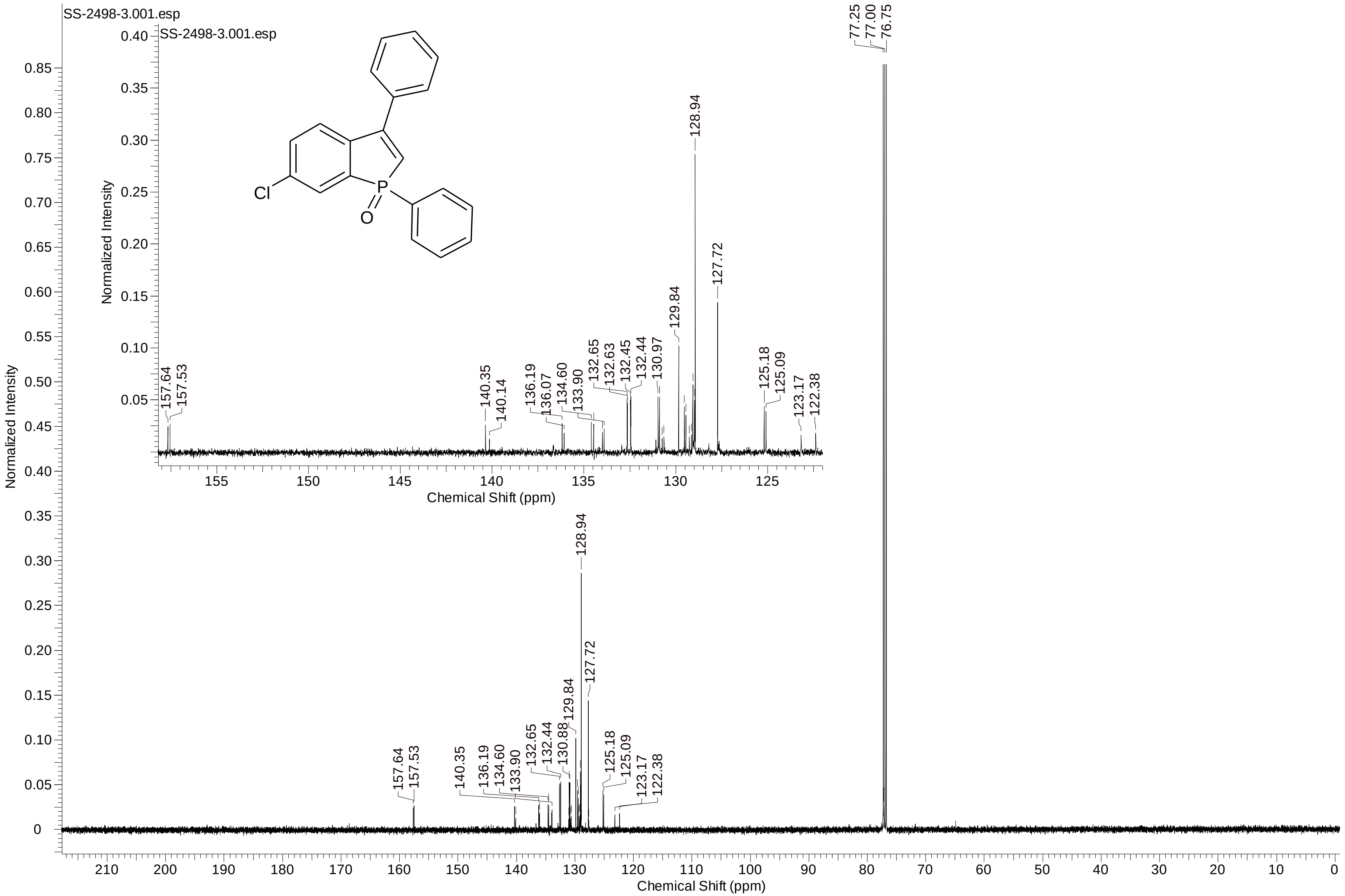

$^{13}C\{^{1}H\}$ NMR spectrum of 6-chloro-1,3-diphenylbenzophosphole oxide (**7a**) (125 MHz, $CDCl_3$)

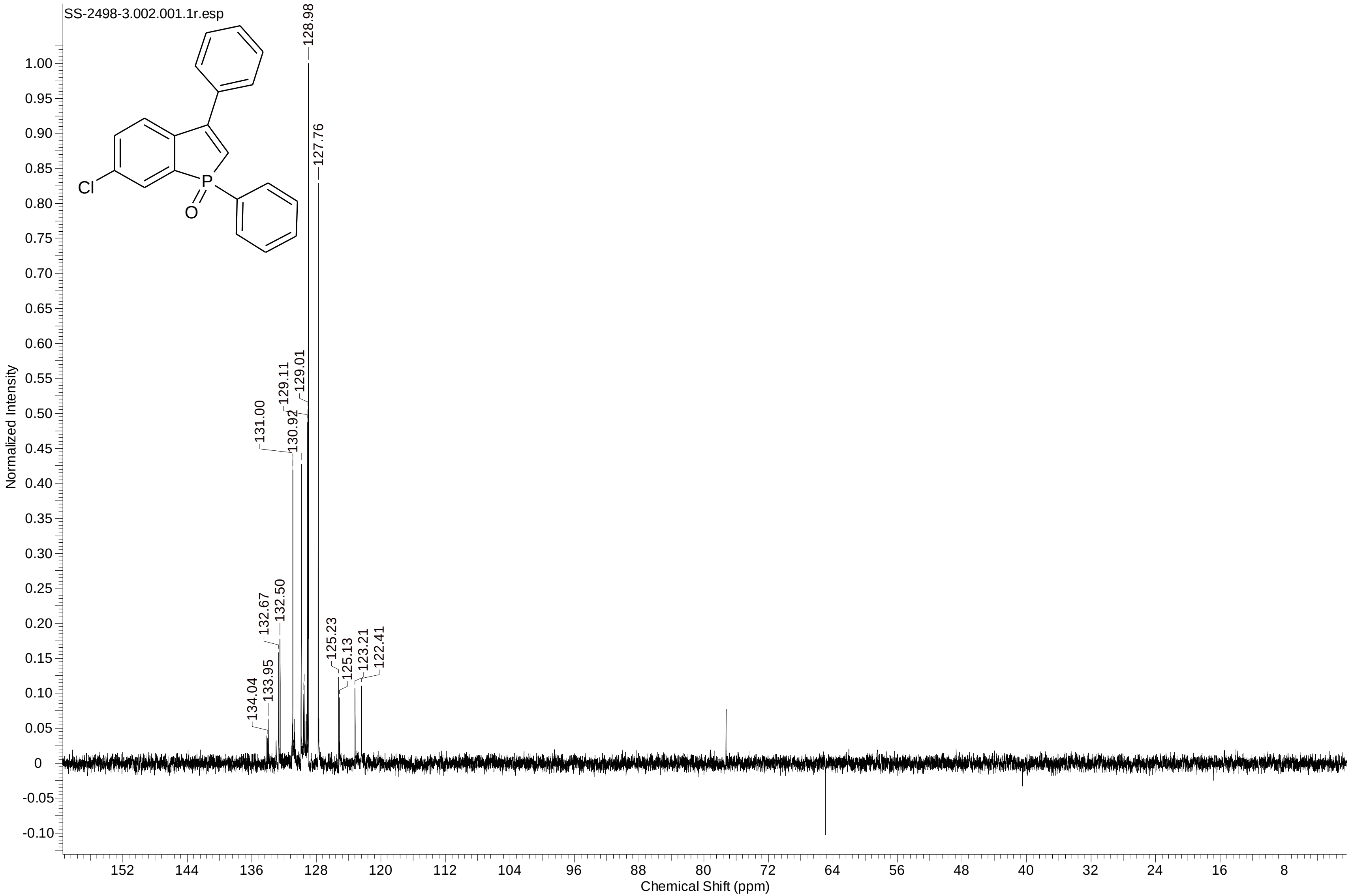

DEPT 135 NMR spectrum of 6-chloro-1,3-diphenylbenzophosphole oxide (**7a**) (125 MHz, $CDCl_3$)

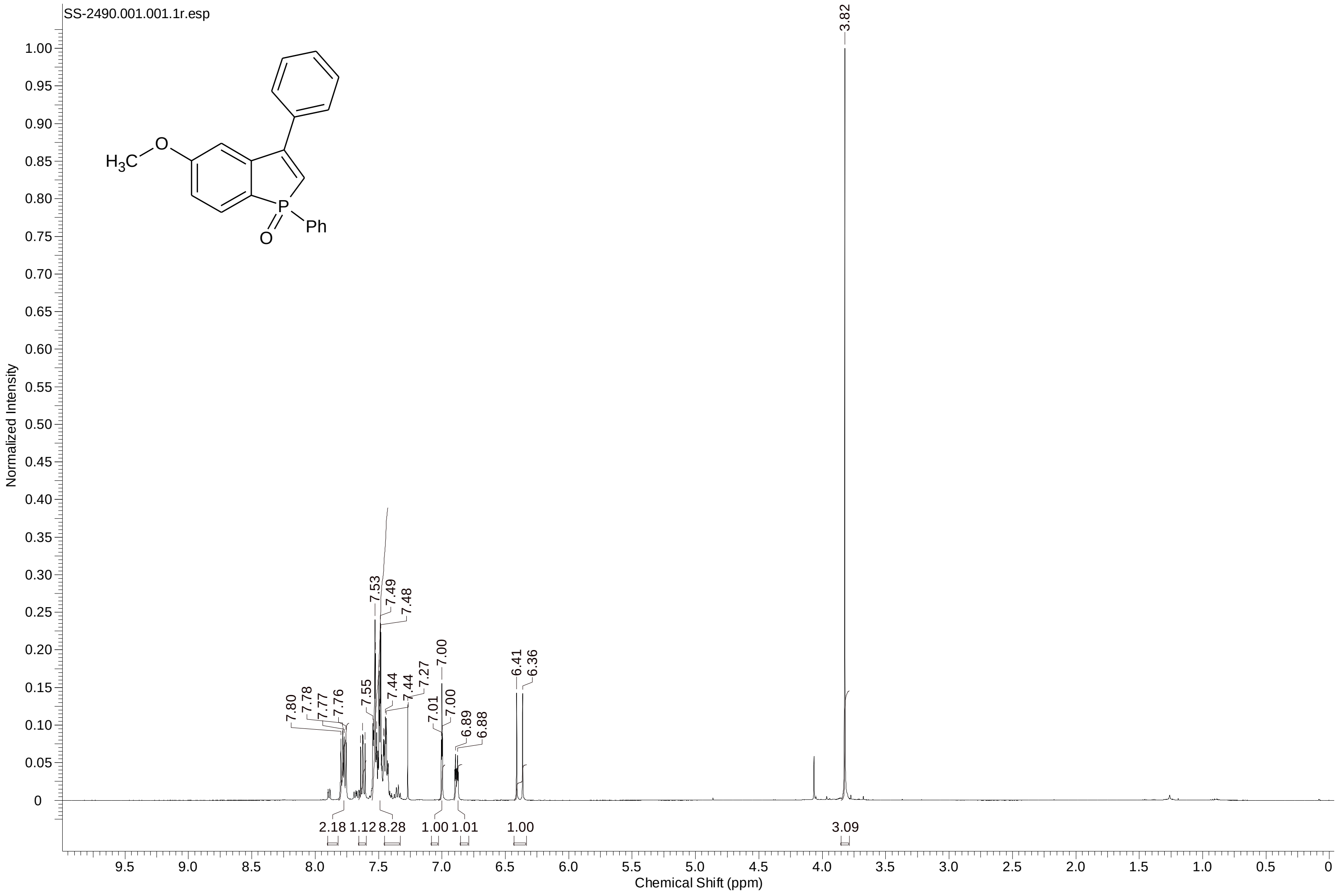

^{1}H NMR spectrum of 1,3-diphenyl-5-methoxybenzophosphole oxide (**8a**) (500 MHz, $CDCl_3$)

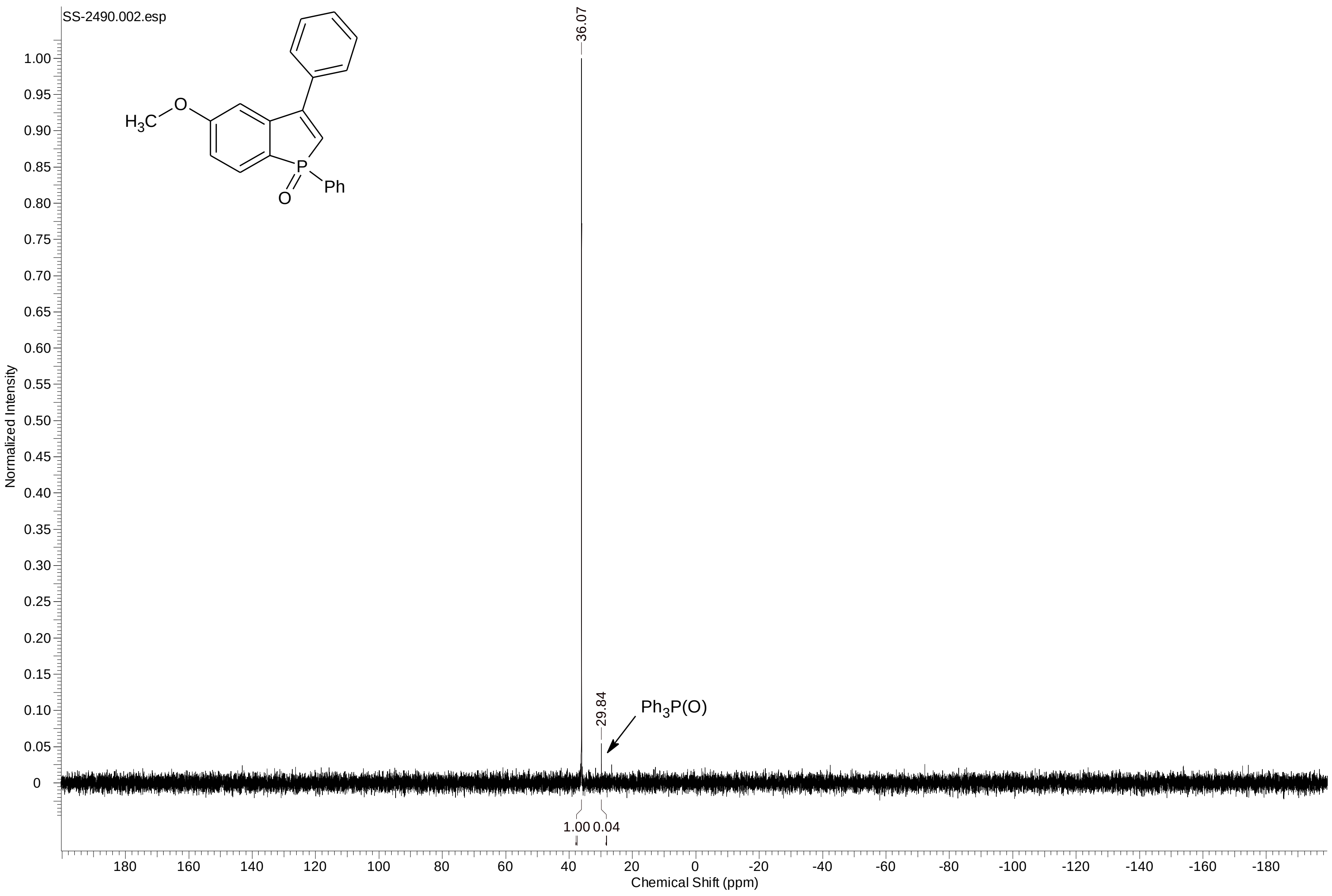

$^{31}P\{^{1}H\}$ NMR spectrum of 1,3-diphenyl-5-methoxybenzophosphole oxide (**8a**) (202 MHz, $CDCl_3$)

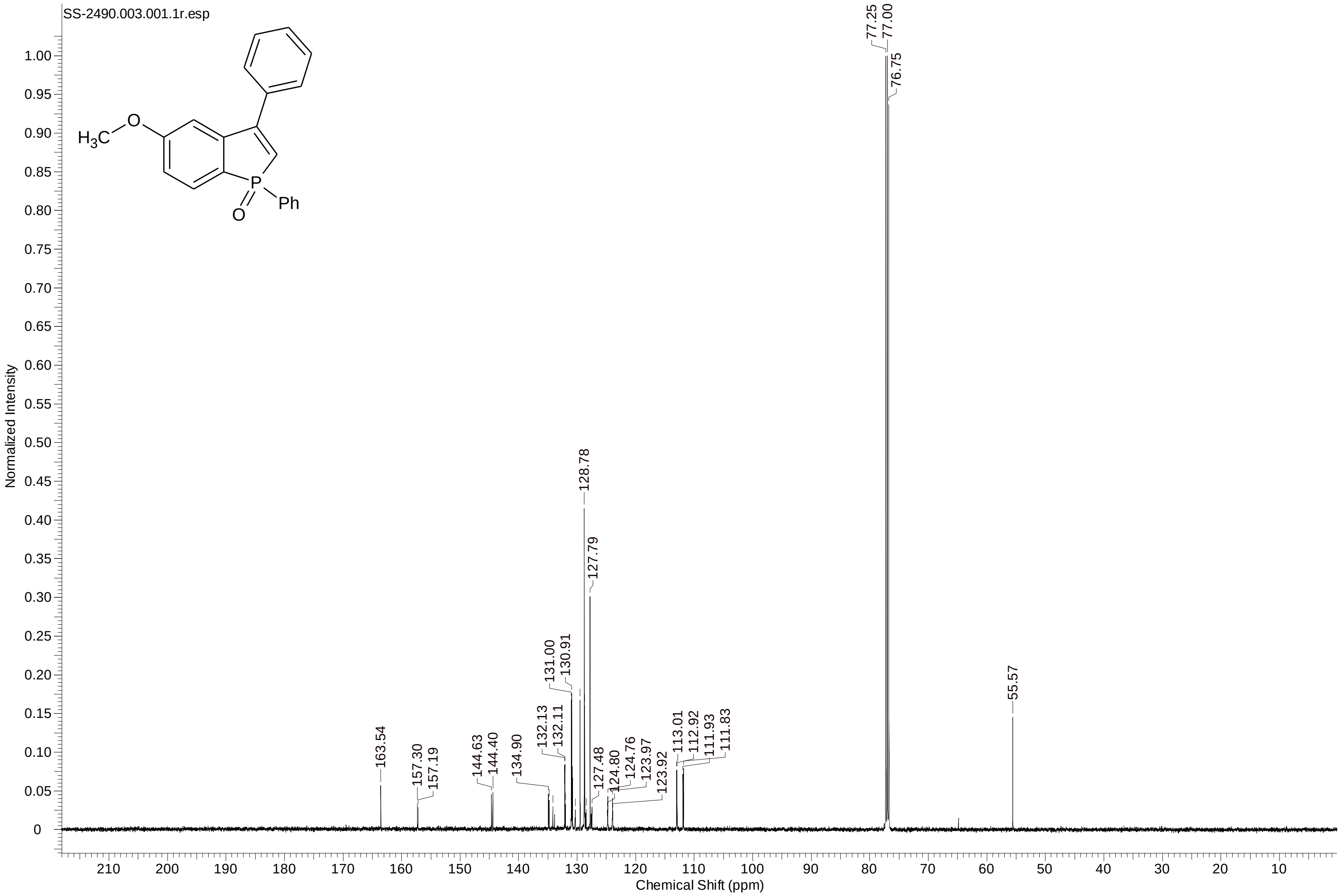

$^{13}C\{^{1}H\}$ NMR spectrum of 1,3-diphenyl-5-methoxybenzophosphole oxide (**8a**) (125 MHz, $CDCl_3$)

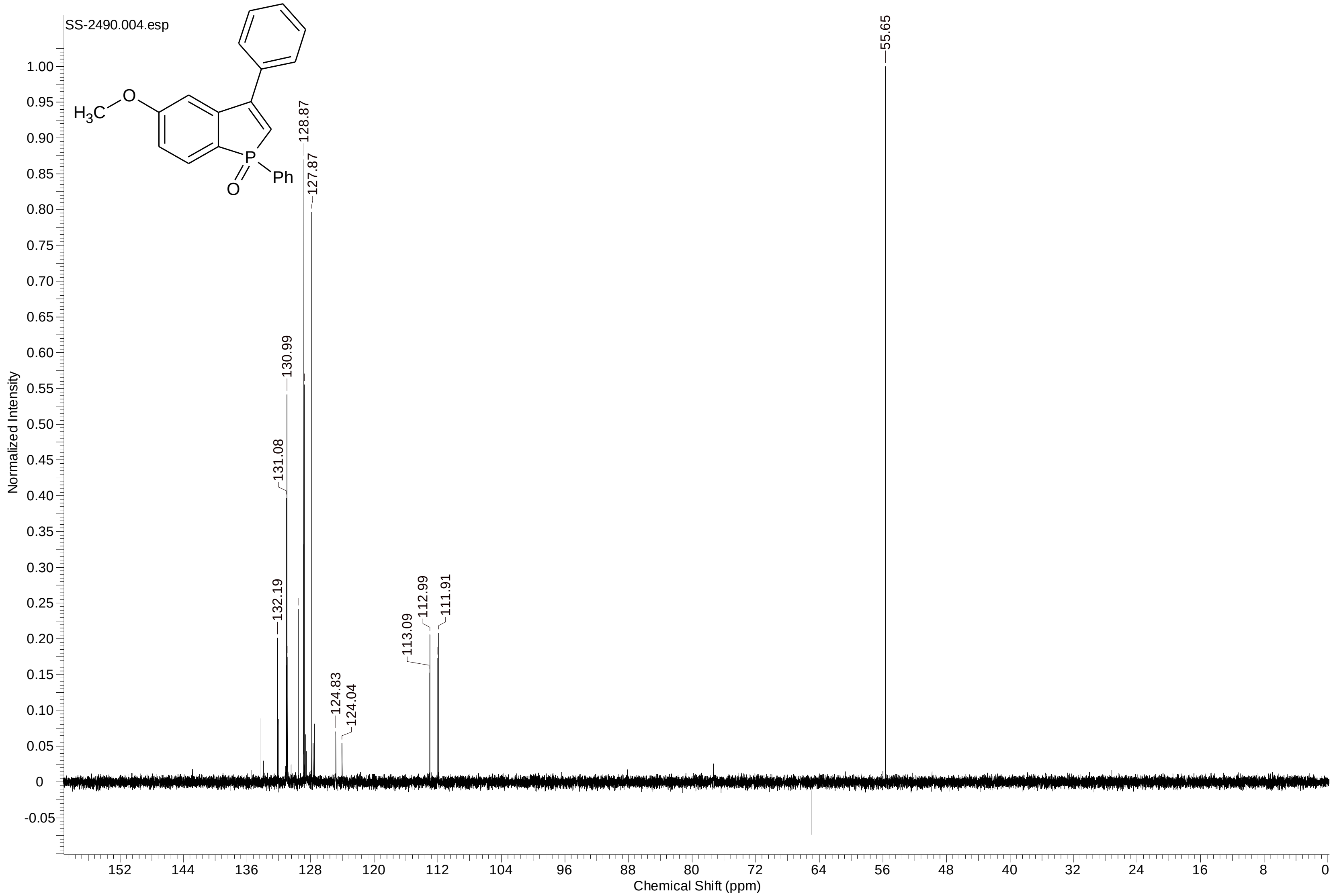

DEPT 135 NMR spectrum of 1,3-diphenyl-5-methoxybenzophosphole oxide (**8a**) (125 MHz, $CDCl_3$)

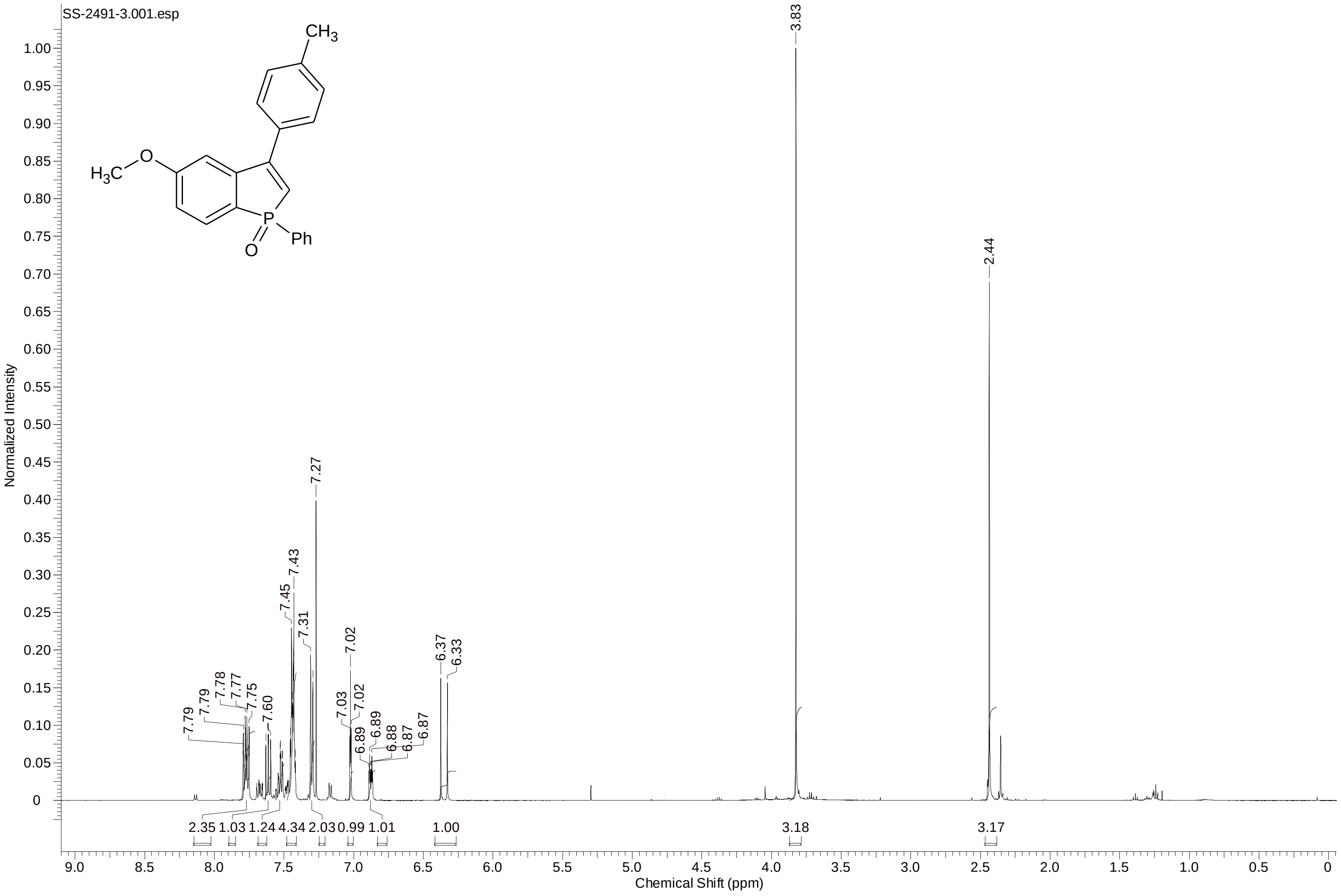

^{1}H NMR spectrum of 1-phenyl-5-methoxy-3-(*p*-tolyl)benzophosphole oxide (**8b**) (500 MHz, $CDCl_3$)

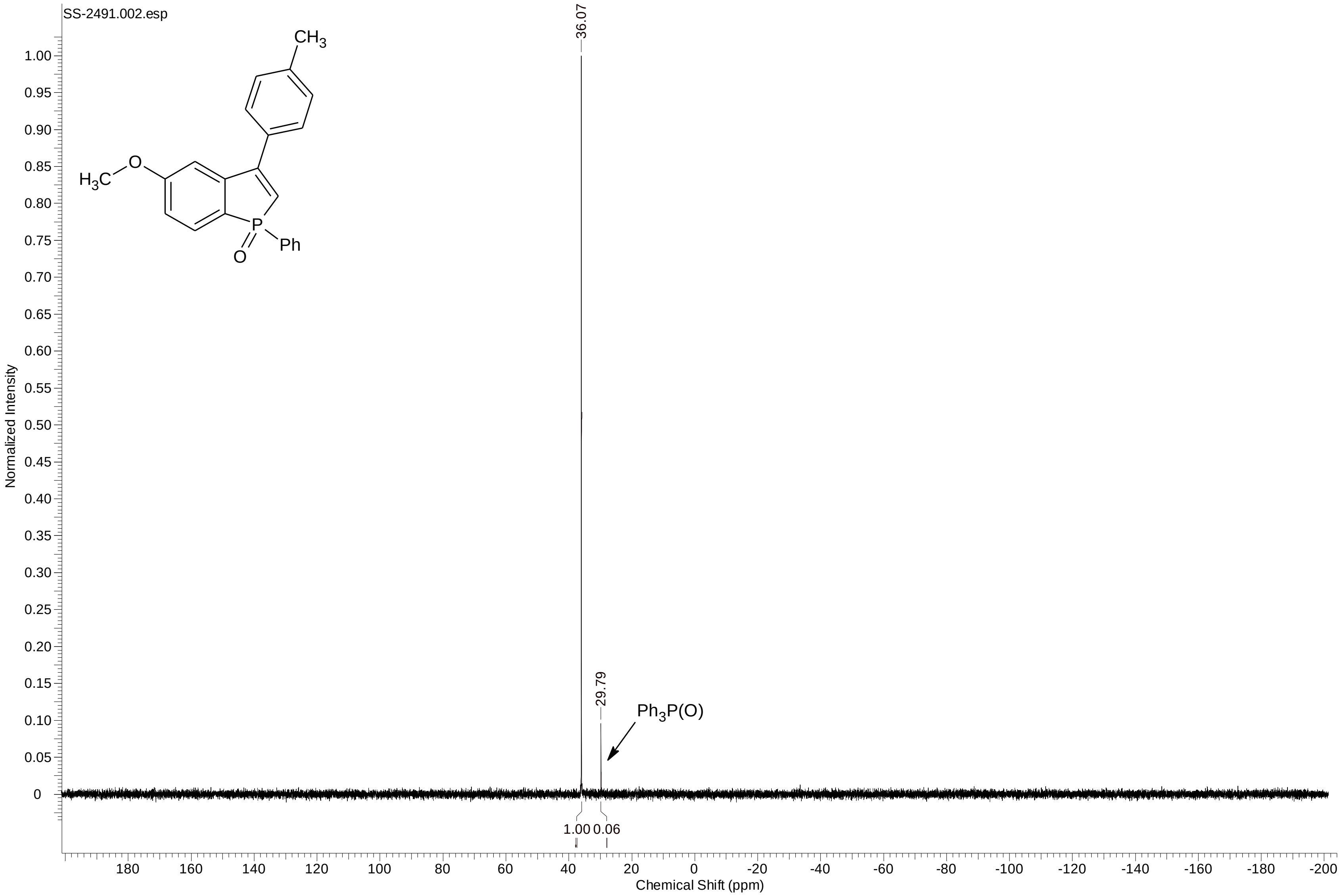

$^{31}P\{^{1}H\}$ NMR spectrum of 1-phenyl-5-methoxy-3-(*p*-tolyl)benzophosphole oxide (**8b**) (202 MHz, $CDCl_3$)

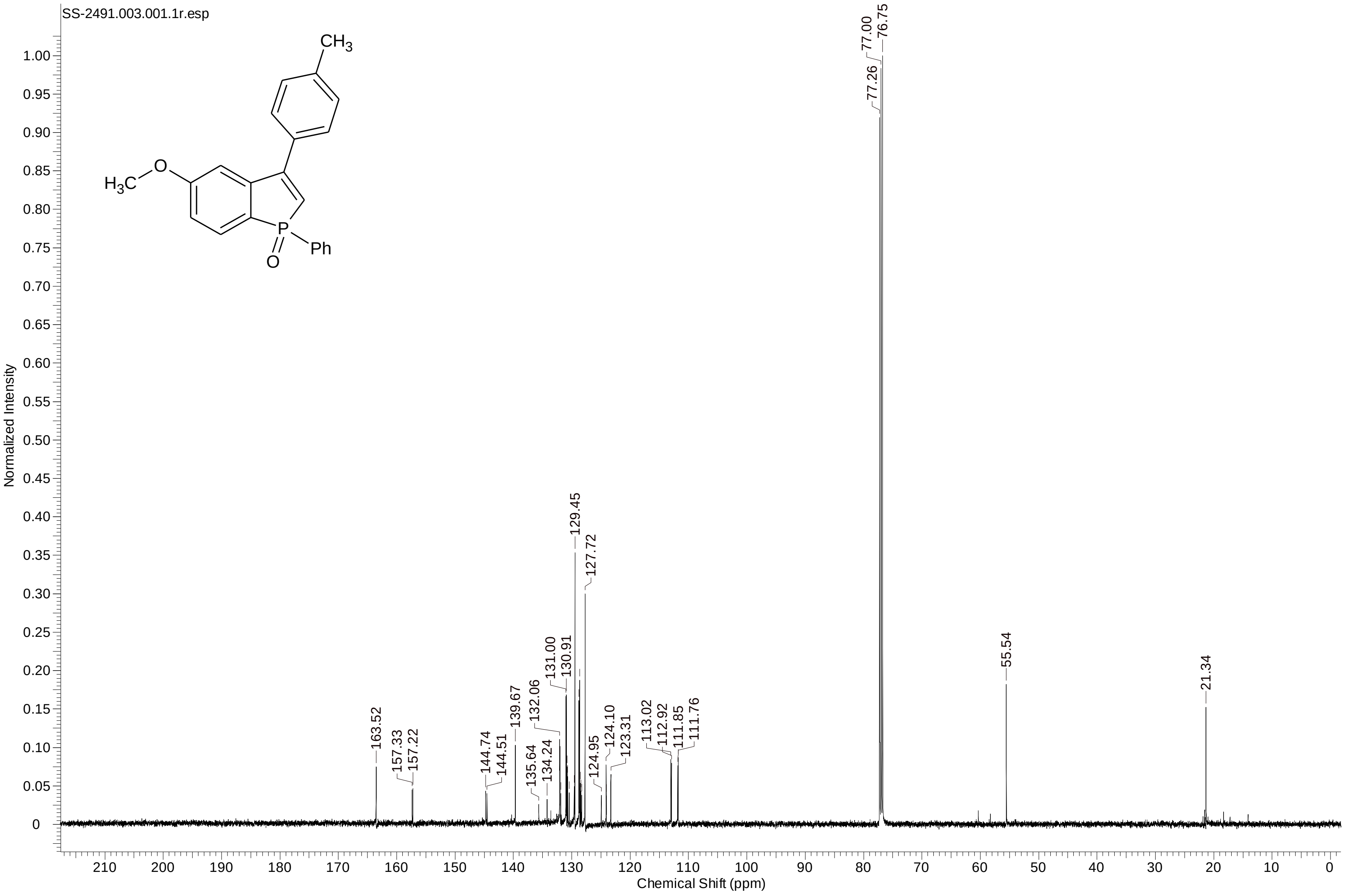

$^{13}C\{^{1}H\}$ NMR spectrum of 1-phenyl-5-methoxy-3-(*p*-tolyl)benzophosphole oxide (**8b**) (125 MHz, $CDCl_3$)

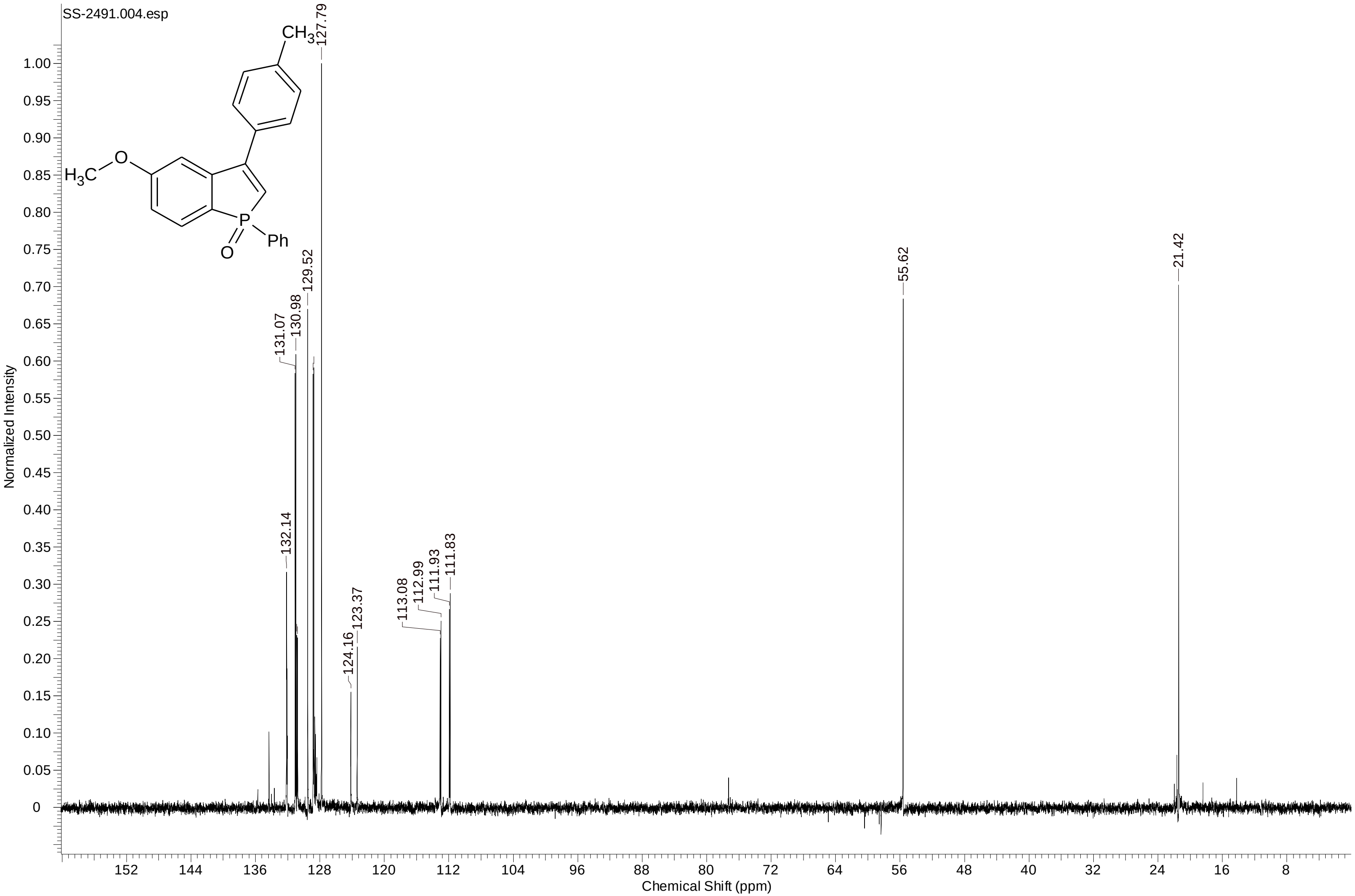

DEPT 135 NMR spectrum of 1-phenyl-5-methoxy-3-(*p*-tolyl)benzophosphole oxide (**8b**) (125 MHz, $CDCl_3$)

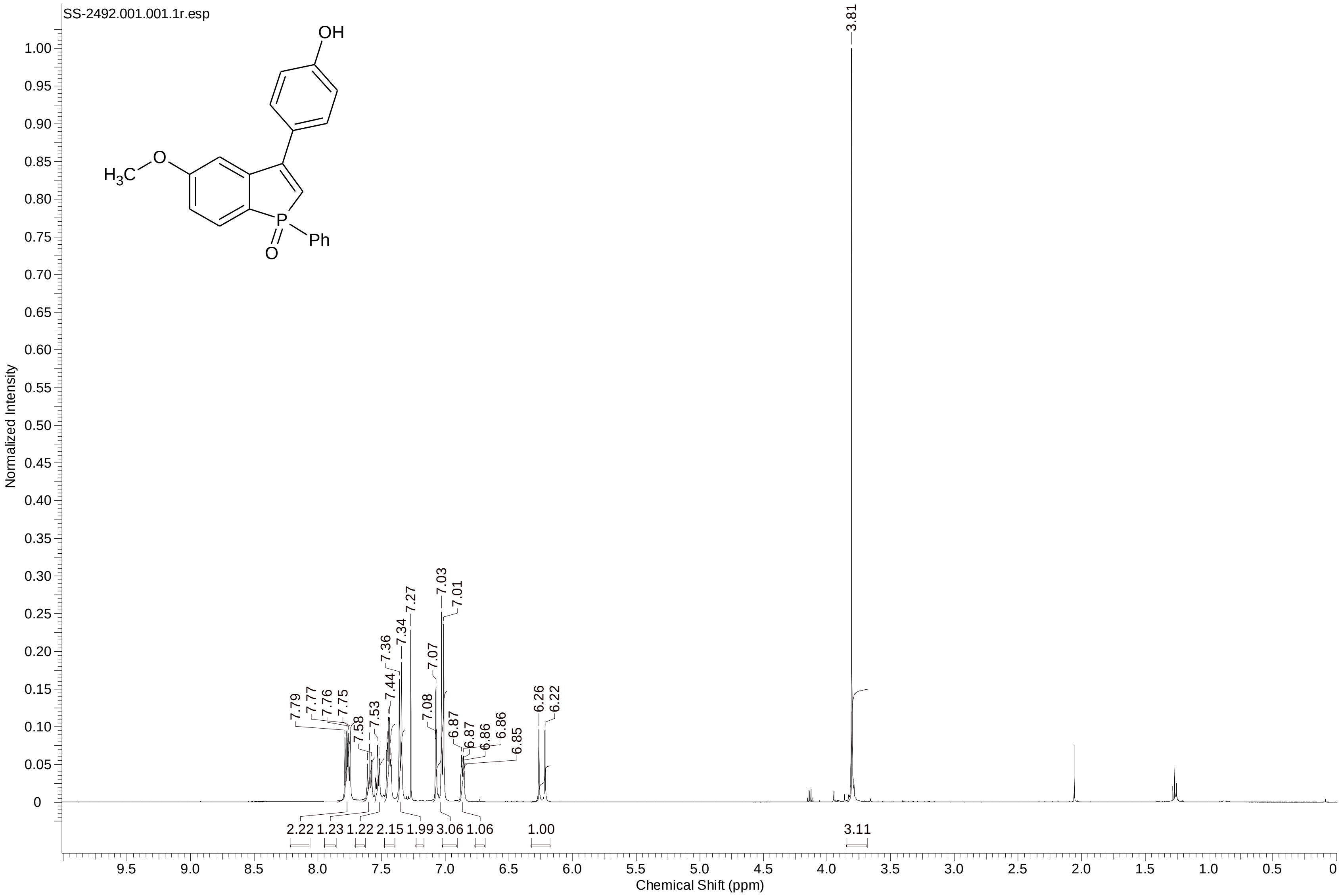

^{1}H NMR spectrum of 3-(*p*-hydroxyphenyl)-1-phenyl-5-methoxybenzophosphole oxide (**8h**) (500 MHz, $CDCl_3$)

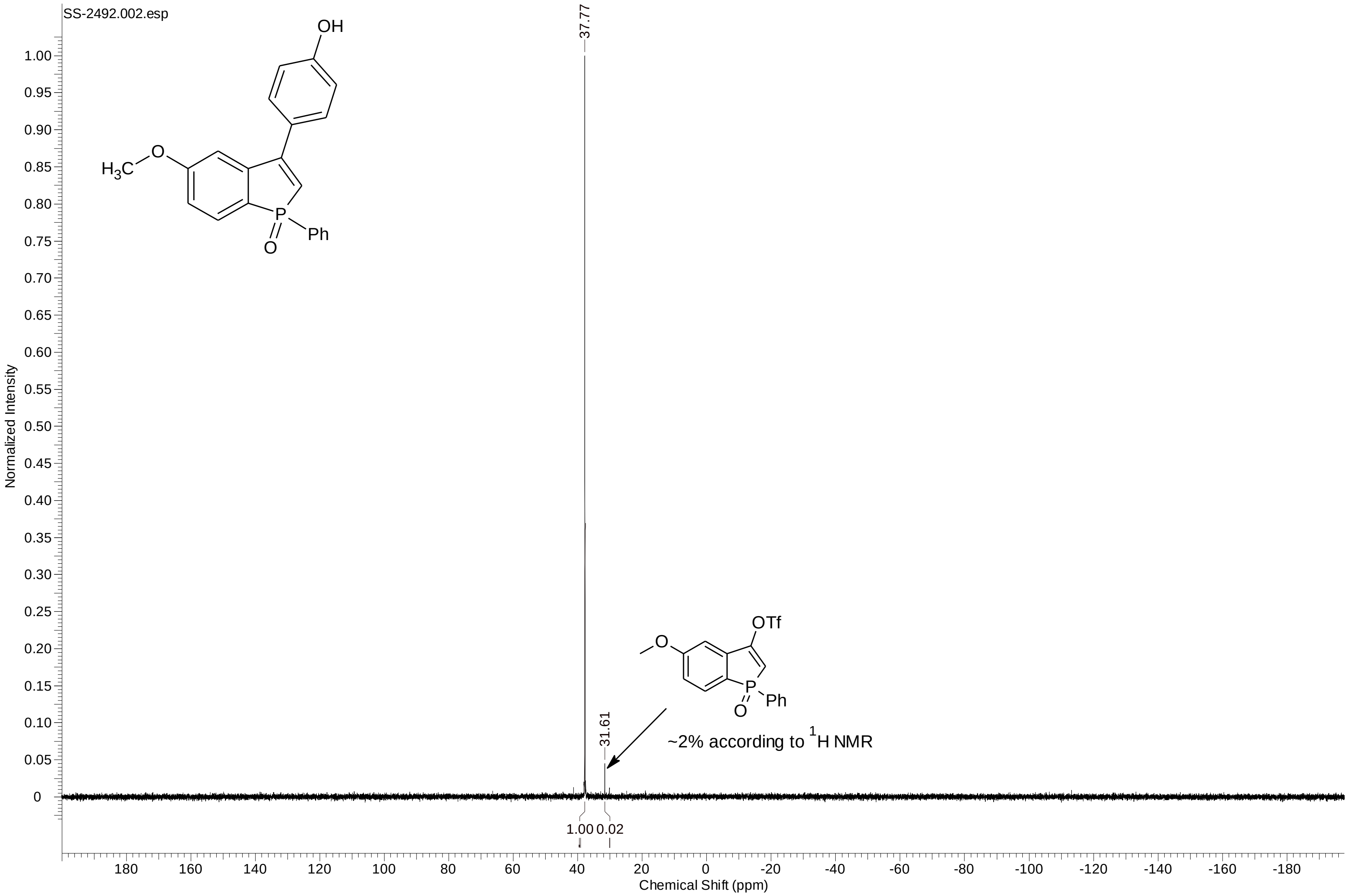

$^{31}P\{^{1}H\}$ NMR spectrum of 3-(*p*-hydroxyphenyl)-1-phenyl-5-methoxybenzophosphole oxide (**8h**) (202 MHz, $CDCl_3$)

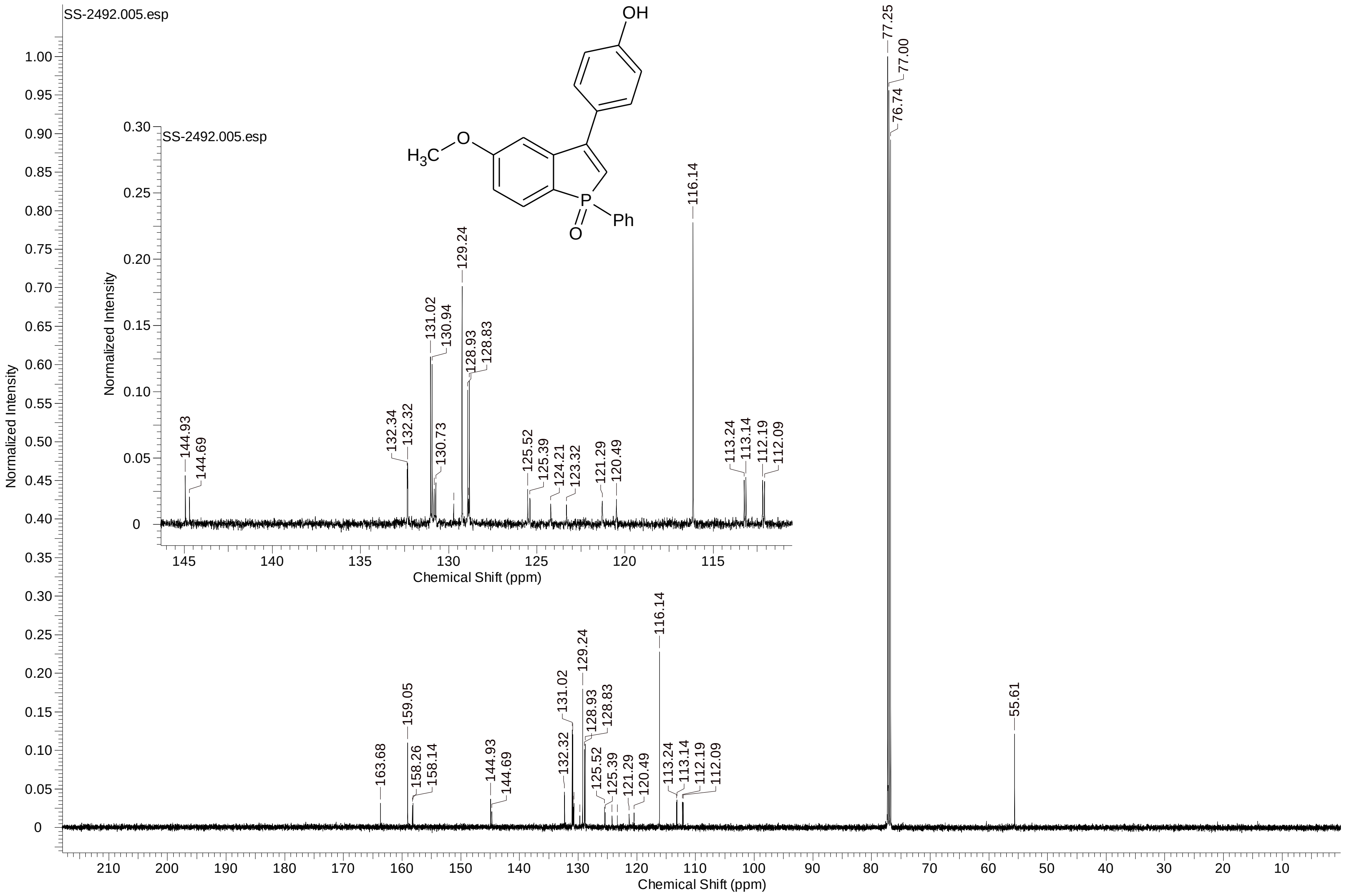

$^{13}C\{^{1}H\}$ NMR spectrum of 3-(*p*-hydroxyphenyl)-1-phenyl-5-methoxybenzophosphole oxide (**8h**) (125 MHz, $CDCl_3$) (low solubility in $CDCl_3$)

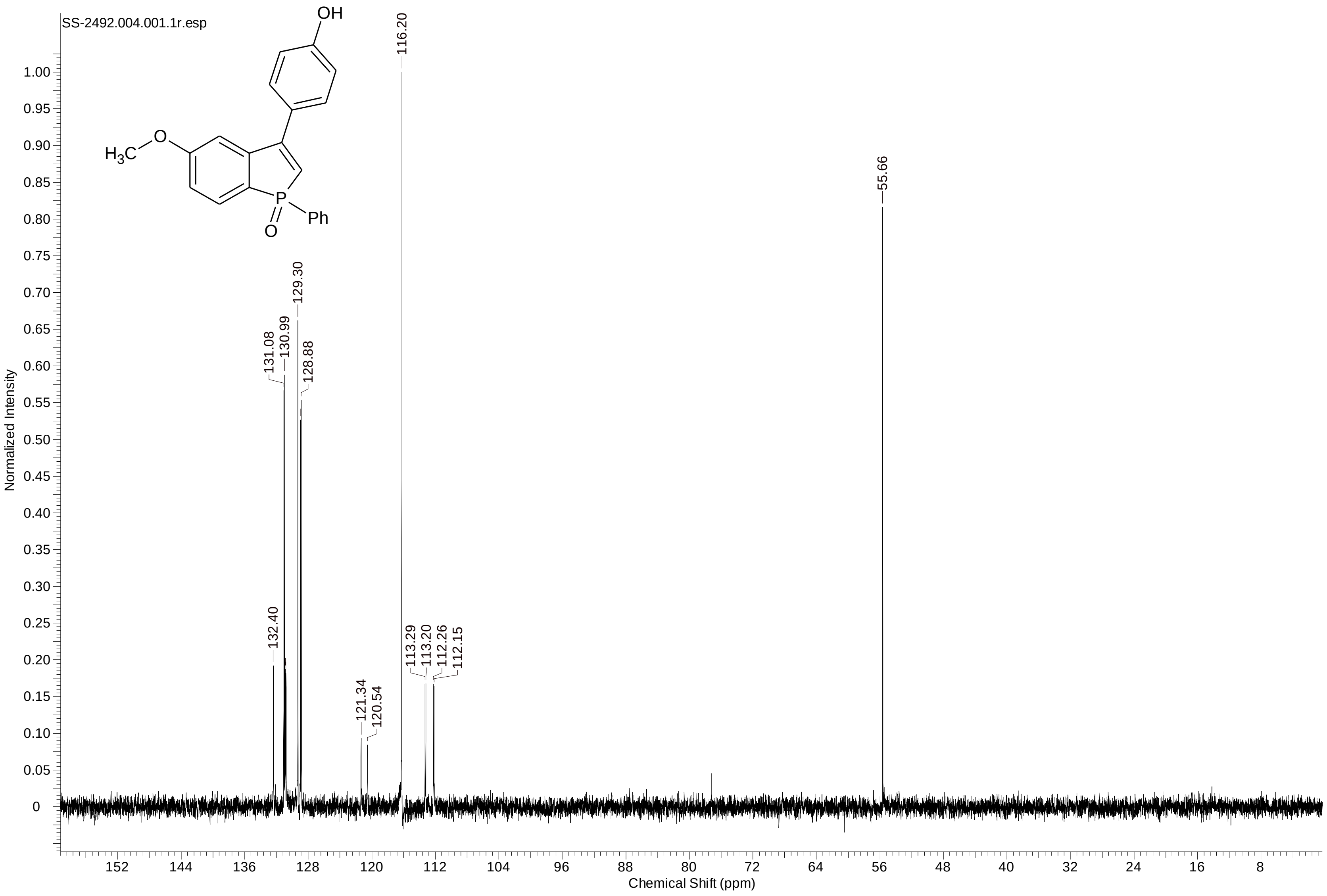

DEPT 135 NMR spectrum of 3-(*p*-hydroxyphenyl)-1-phenyl-5-methoxybenzophosphole oxide (**8h**) (125 MHz, $CDCl_3$)

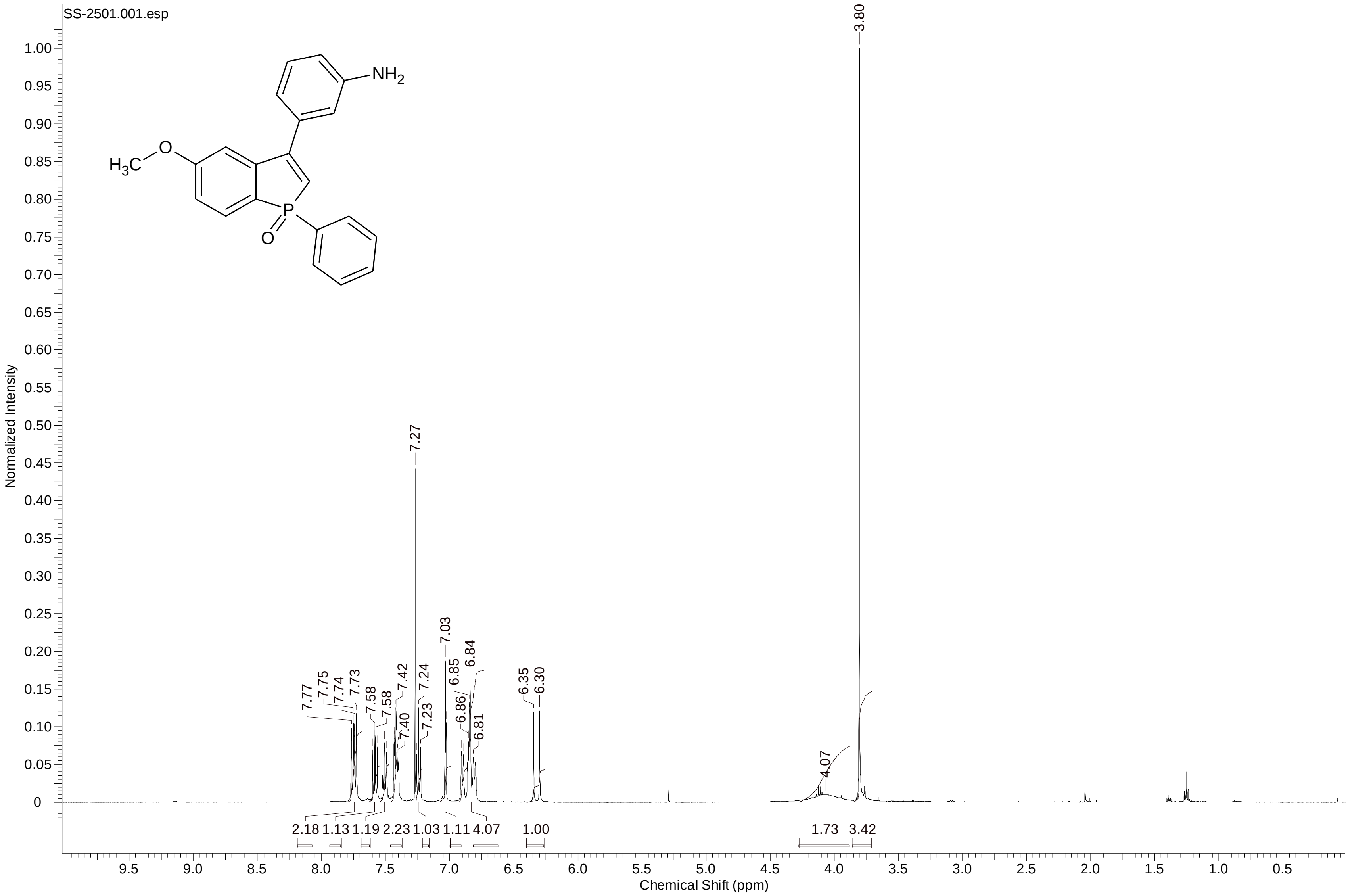

^{1}H NMR spectrum of 3-(*m*-aminophenyl)-1-phenyl-5-methoxybenzophosphole oxide (**8i**) (500 MHz, $CDCl_3$)

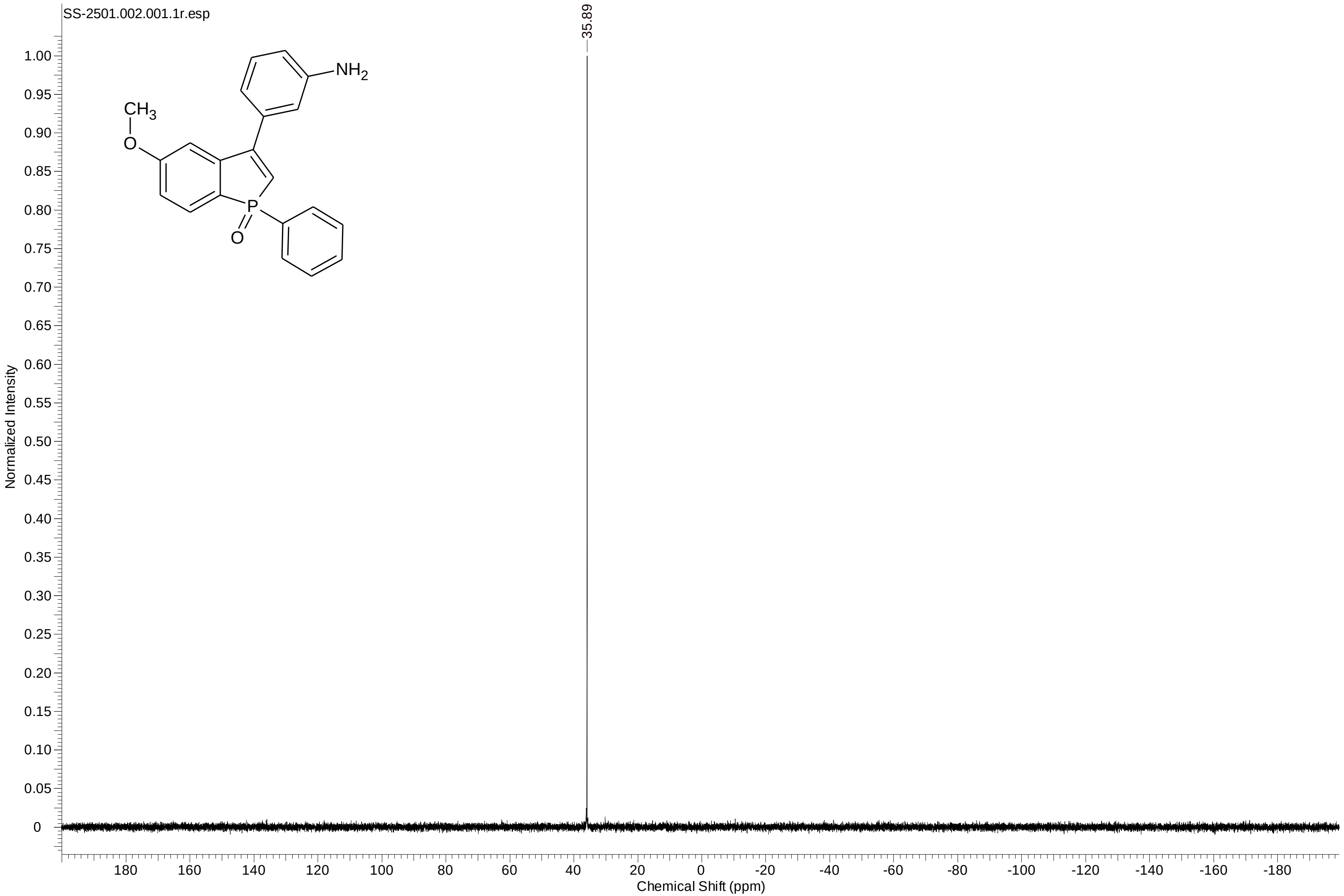

^{31}P{^{1}H} NMR spectrum of 3-(*m*-aminophenyl)-1-phenyl-5-methoxybenzophosphole oxide (**8i**) (202 MHz, $CDCl_3$)

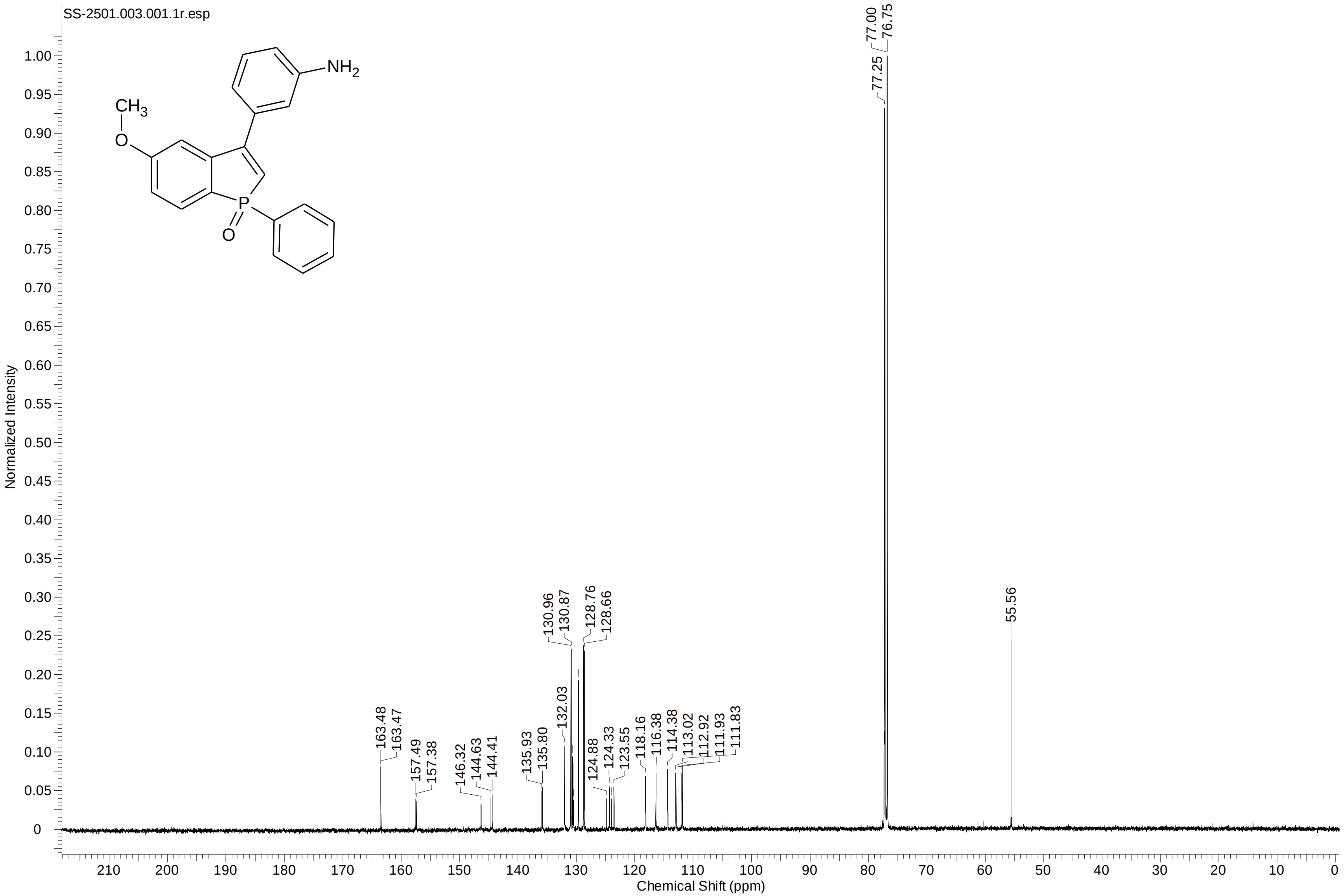

$^{13}C\{^{1}H\}$ NMR spectrum of 3-(*m*-aminophenyl)-1-phenyl-5-methoxybenzophosphole oxide (**8i**) (125 MHz, $CDCl_3$)

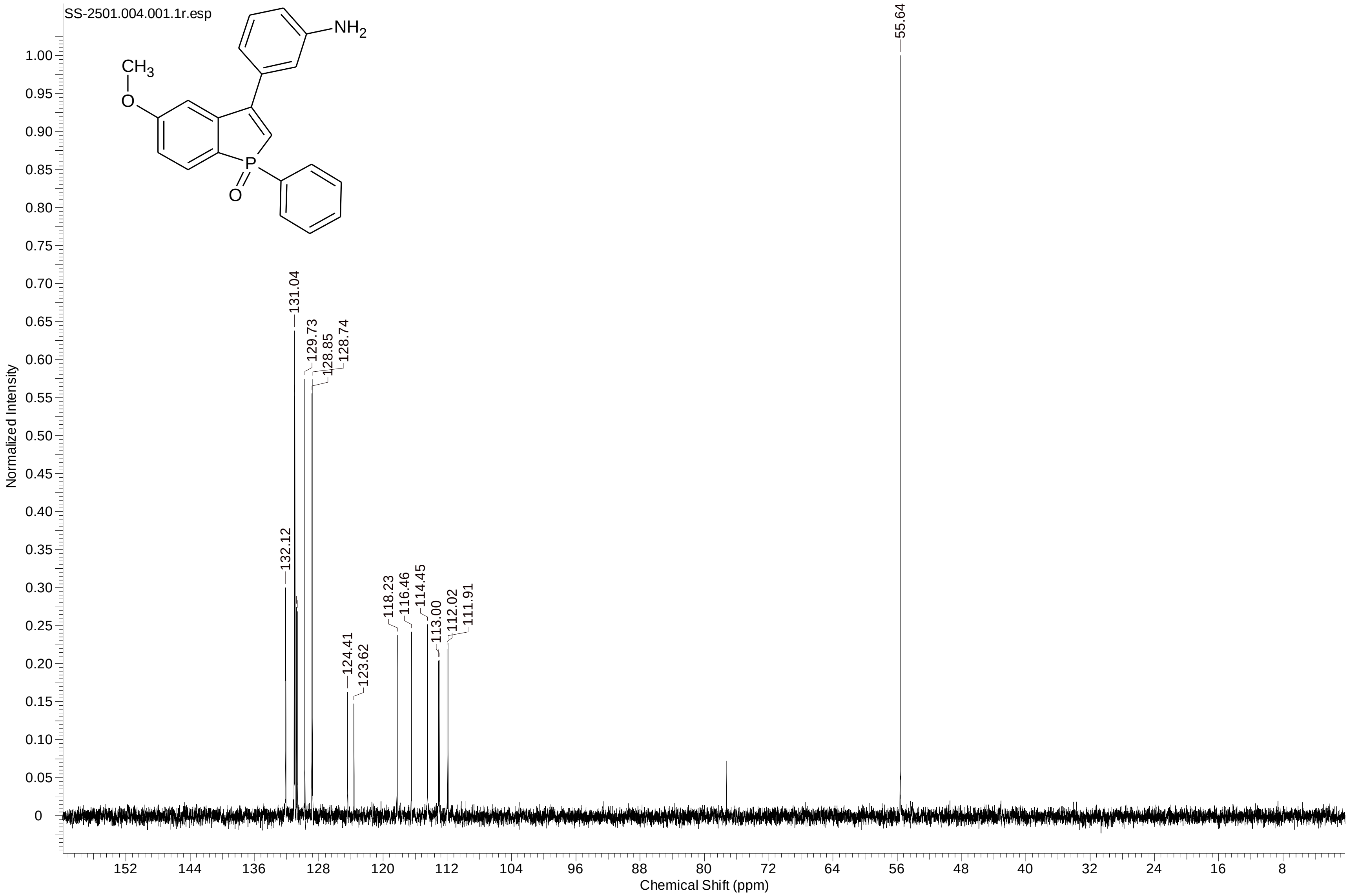

DEPT 135 NMR spectrum of 3-(*m*-aminophenyl)-1-phenyl-5-methoxybenzophosphole oxide (**8i**) (125 MHz, $CDCl_3$)

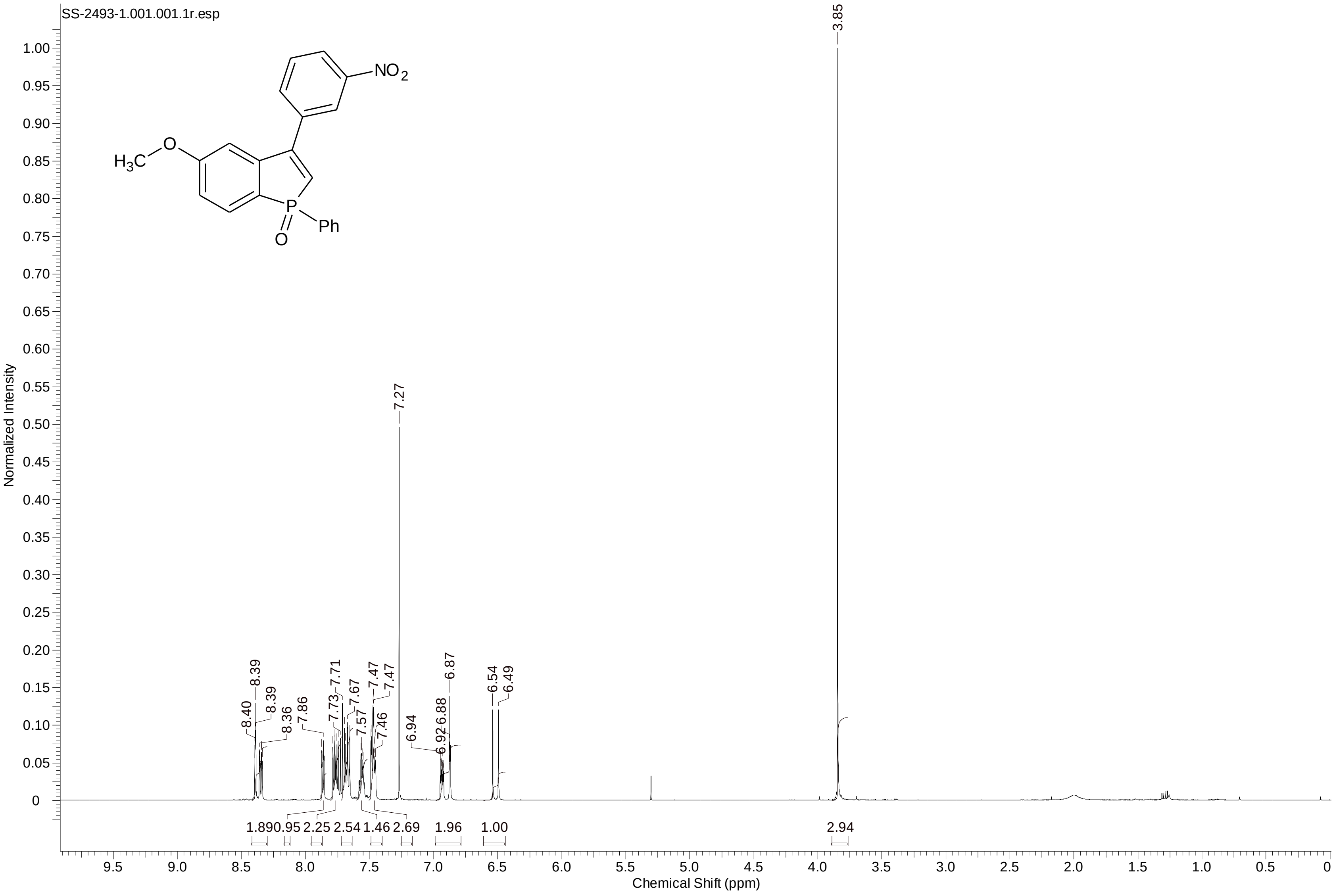

^{1}H NMR spectrum of 1-phenyl-5-methoxy-3-(*p*-nitrophenyl)benzophosphole oxide (**8n**) (500 MHz, $CDCl_3$)

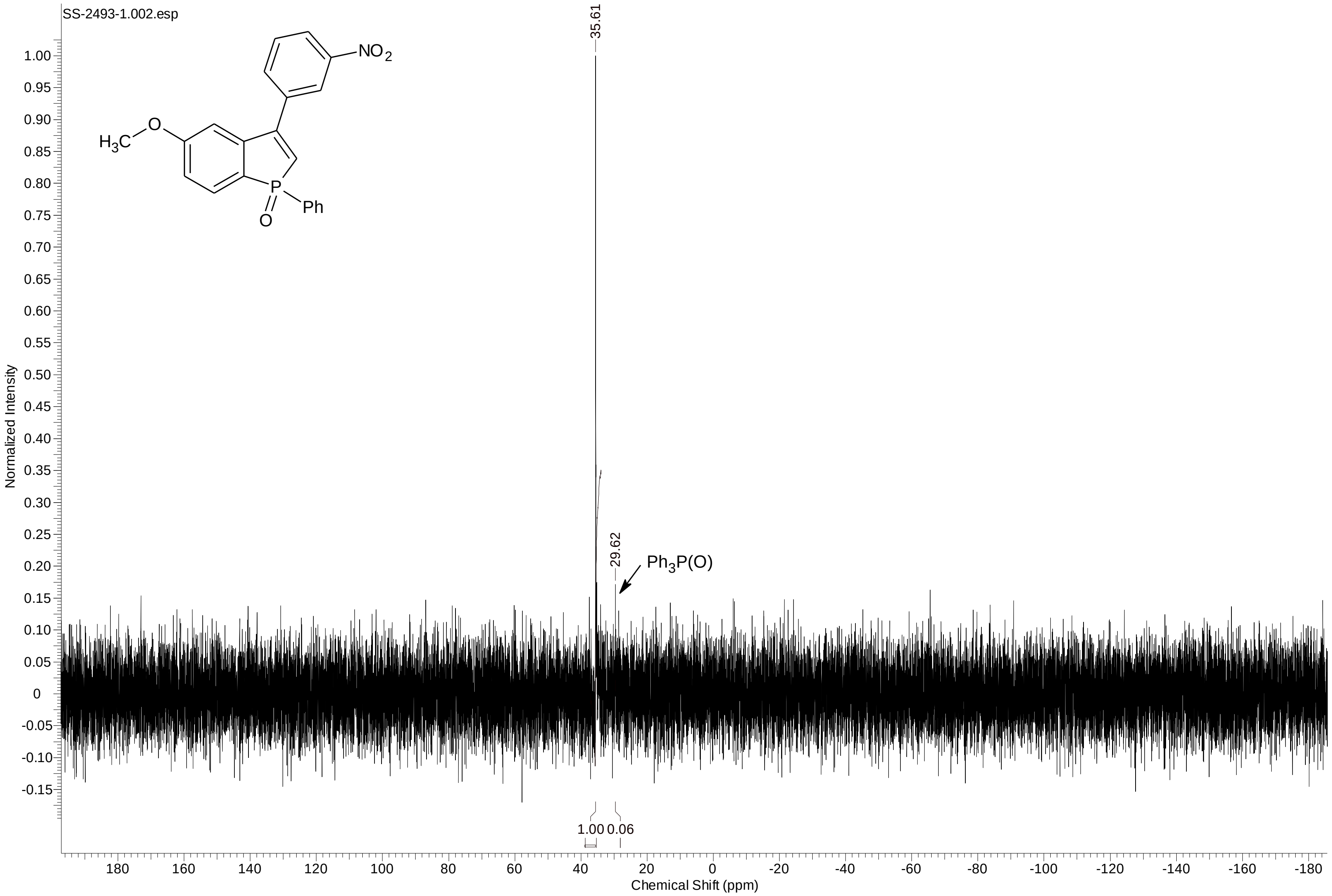

$^{31}P\{^{1}H\}$ NMR spectrum of 1-phenyl-5-methoxy-3-(*p*-nitrophenyl)benzophosphole oxide (**8n**) (202 MHz, $CDCl_3$)

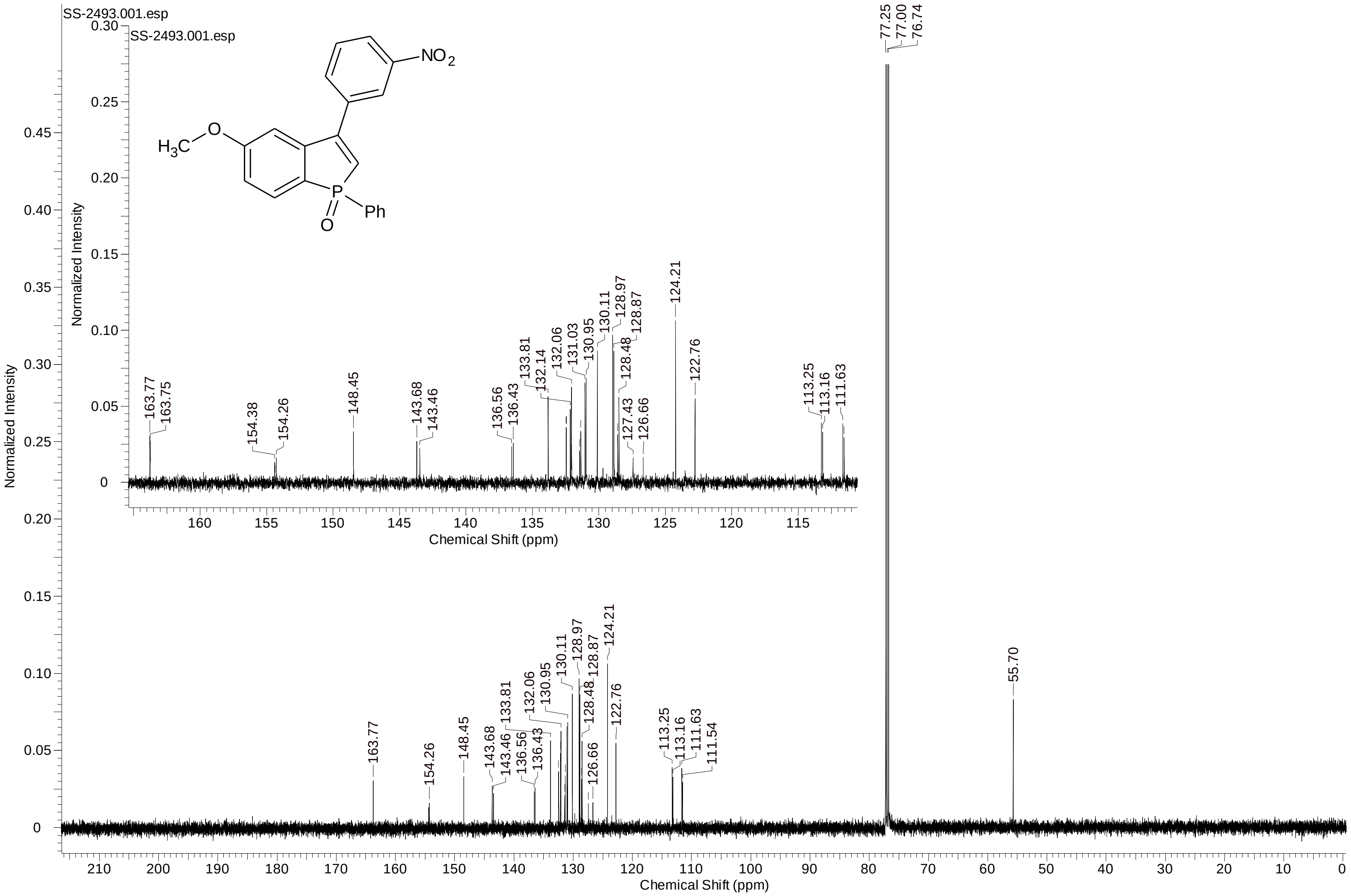

$^{13}C\{^{1}H\}$ NMR spectrum of 1-phenyl-5-methoxy-3-(*p*-nitrophenyl)benzophosphole oxide (**8n**) (125 MHz, $CDCl_3$)

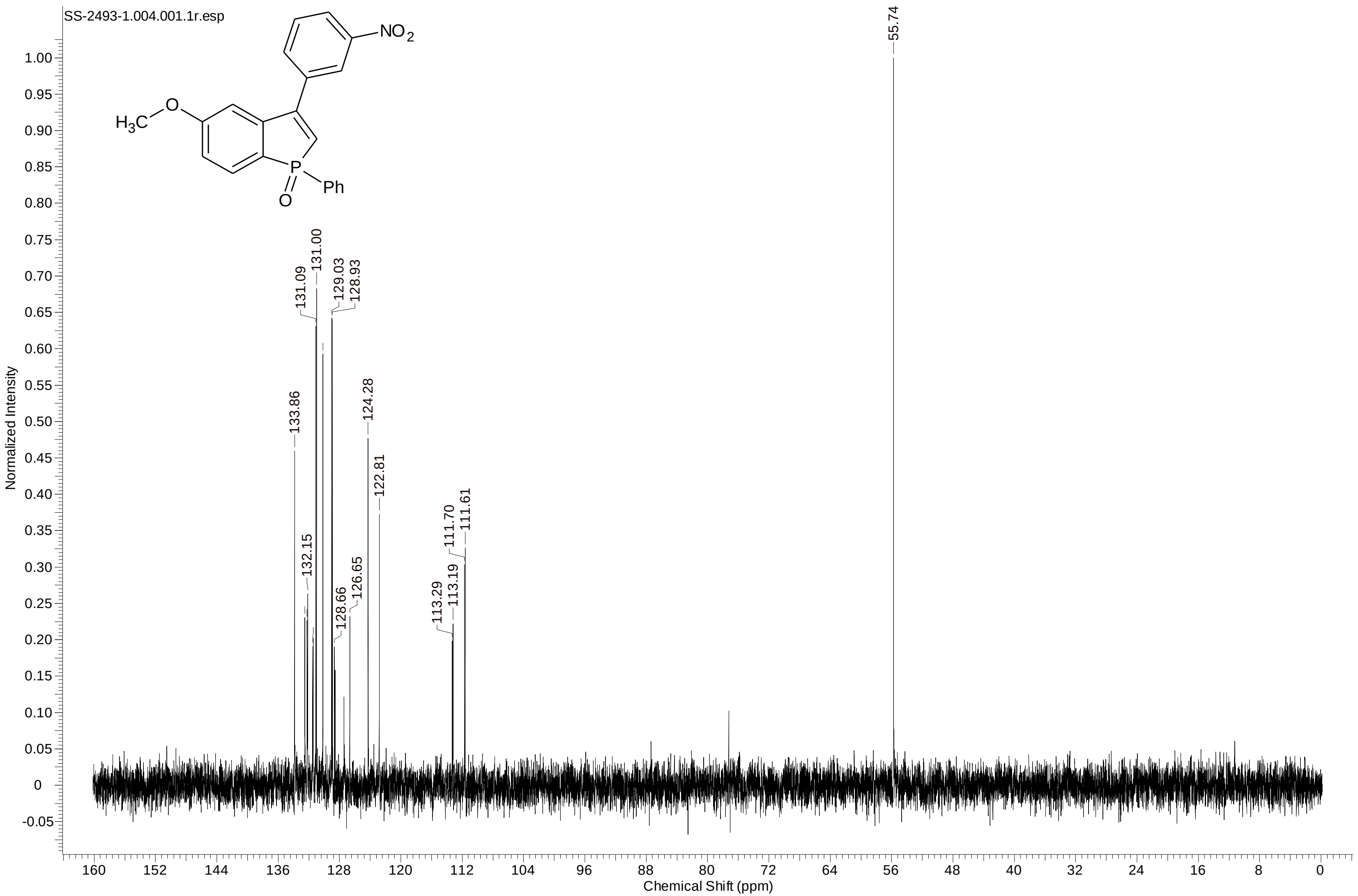

DEPT 135 NMR spectrum of 1-phenyl-5-methoxy-3-(*p*-nitrophenyl)benzophosphole oxide (**8n**) (125 MHz, $CDCl_3$)

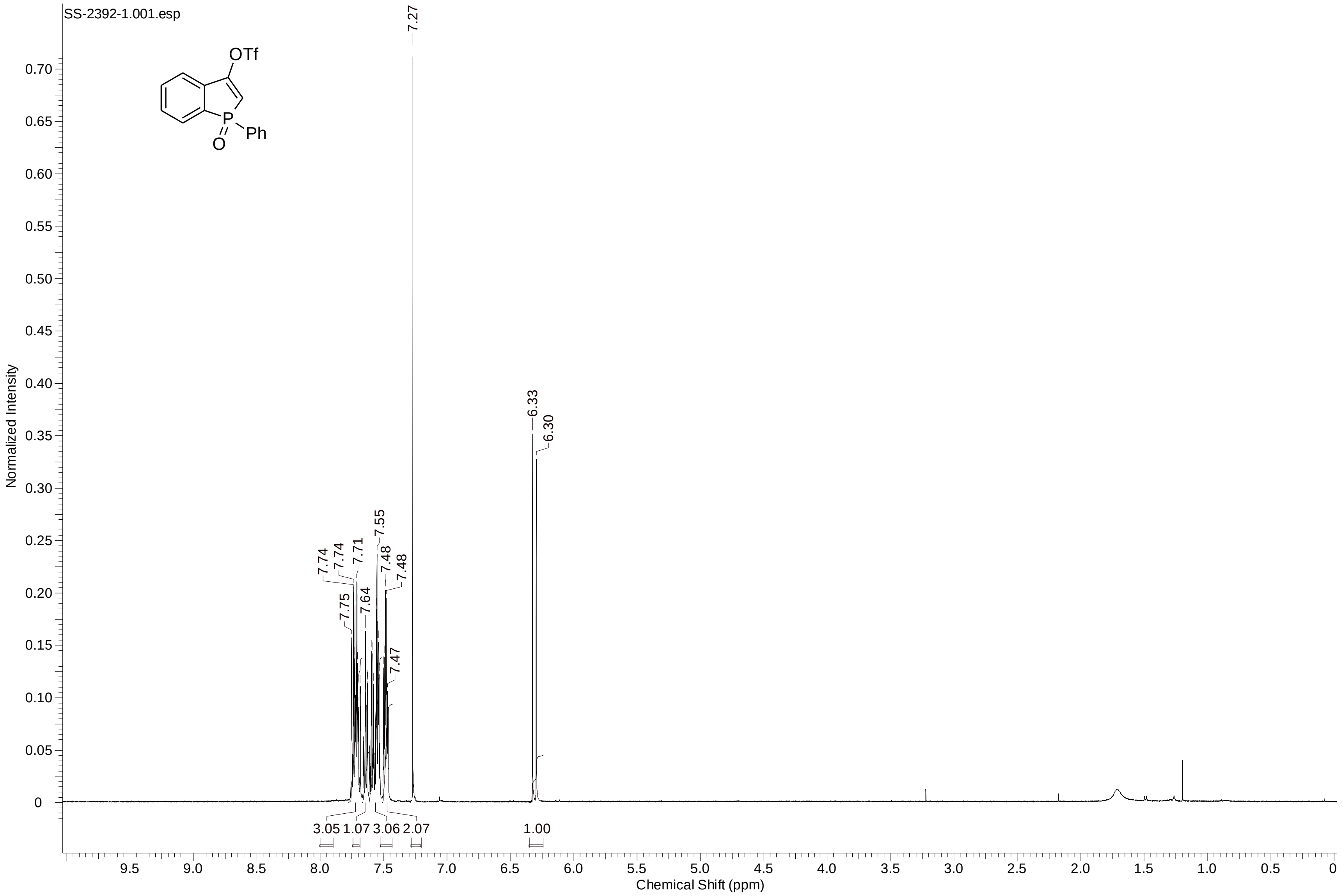

^{1}H NMR spectrum of 1-oxido-1-phenyl-1H-phosphindol-3-yl trifluoromethanesulfonate (**3a**) (500 MHz, $CDCl_3$)[3]

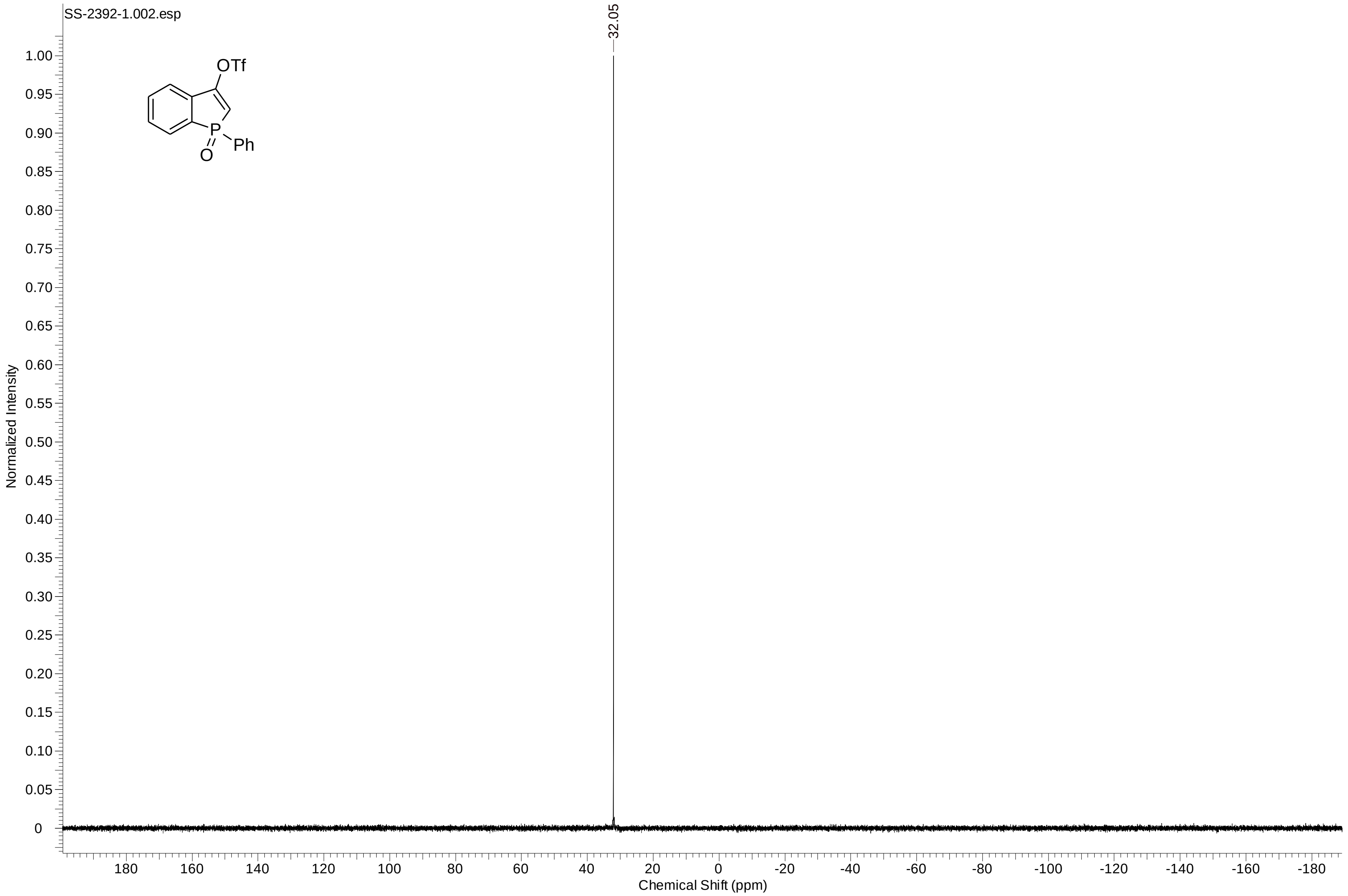

$^{31}P\{^1H\}$ NMR spectrum of 1-oxido-1-phenyl-1H-phosphindol-3-yl trifluoromethanesulfonate (**3a**) (202 MHz, $CDCl_3$)

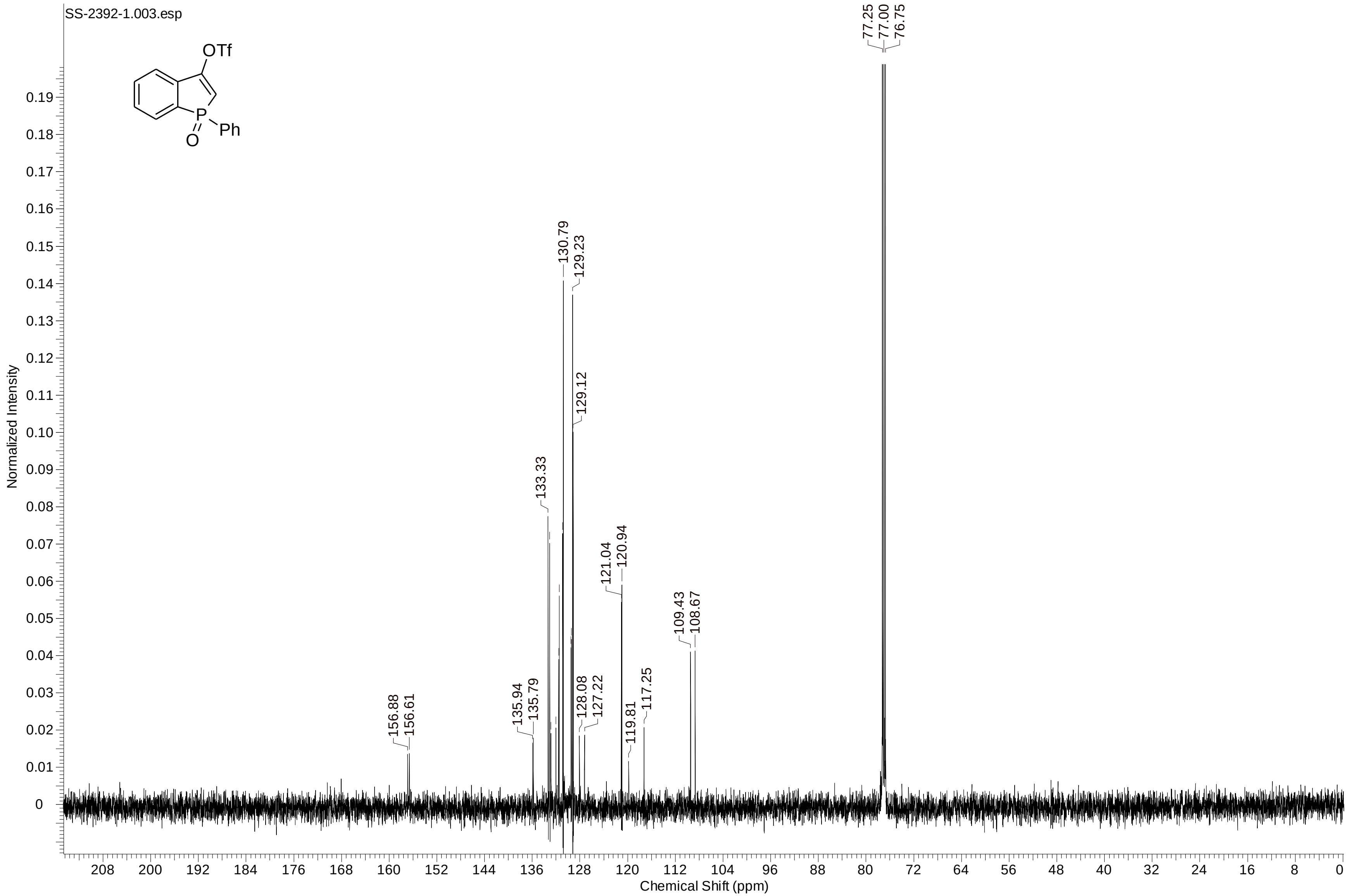

$^{13}C\{^{1}H\}$ NMR spectrum of 1-oxido-1-phenyl-1H-phosphindol-3-yl trifluoromethanesulfonate (**3a**) (125 MHz, $CDCl_3$)

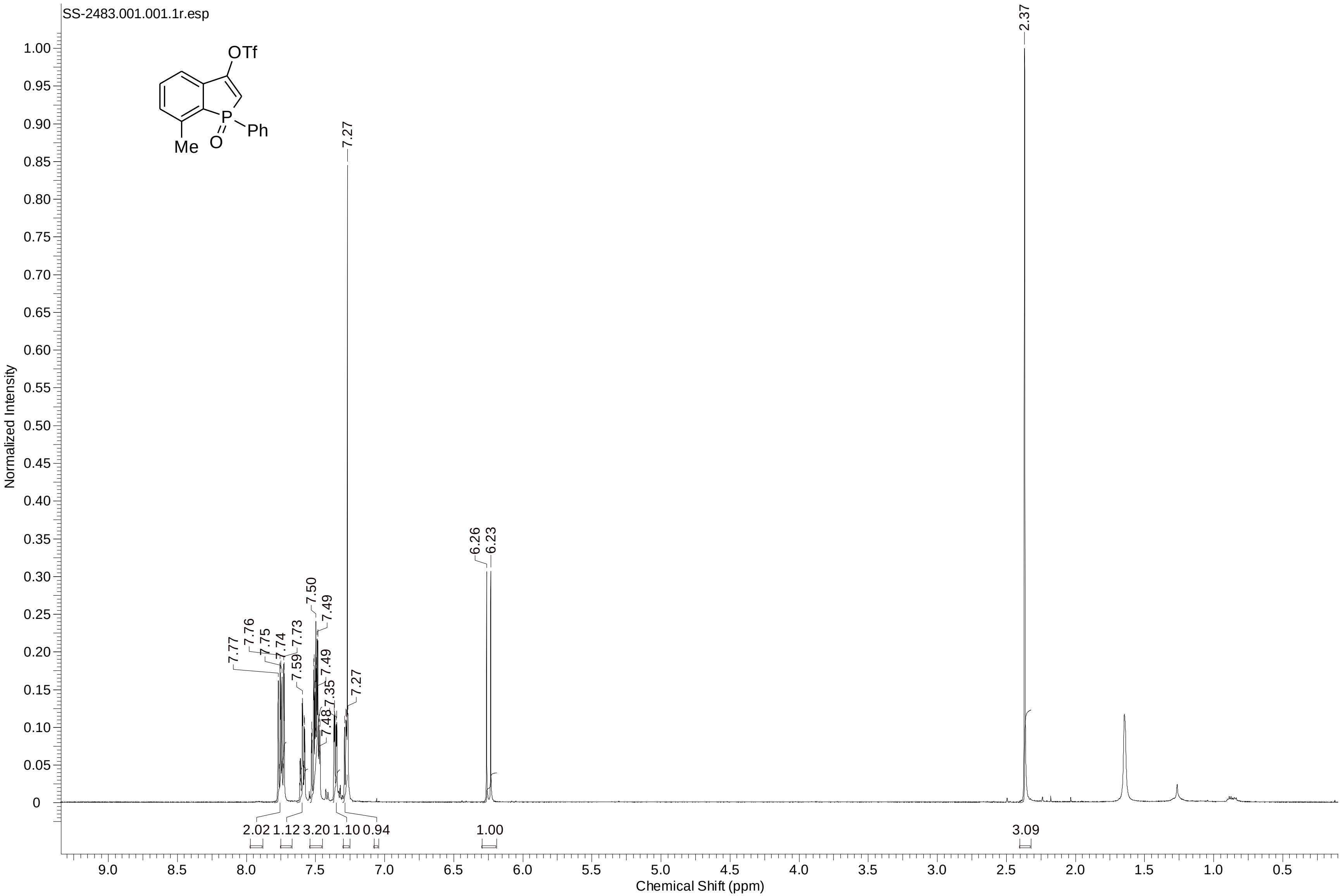

^{1}H NMR spectrum of 1-oxido-1-phenyl-1H-7-methylphosphindol-3-yl trifluoromethanesulfonate (**3b**) (500 MHz, $CDCl_3$)

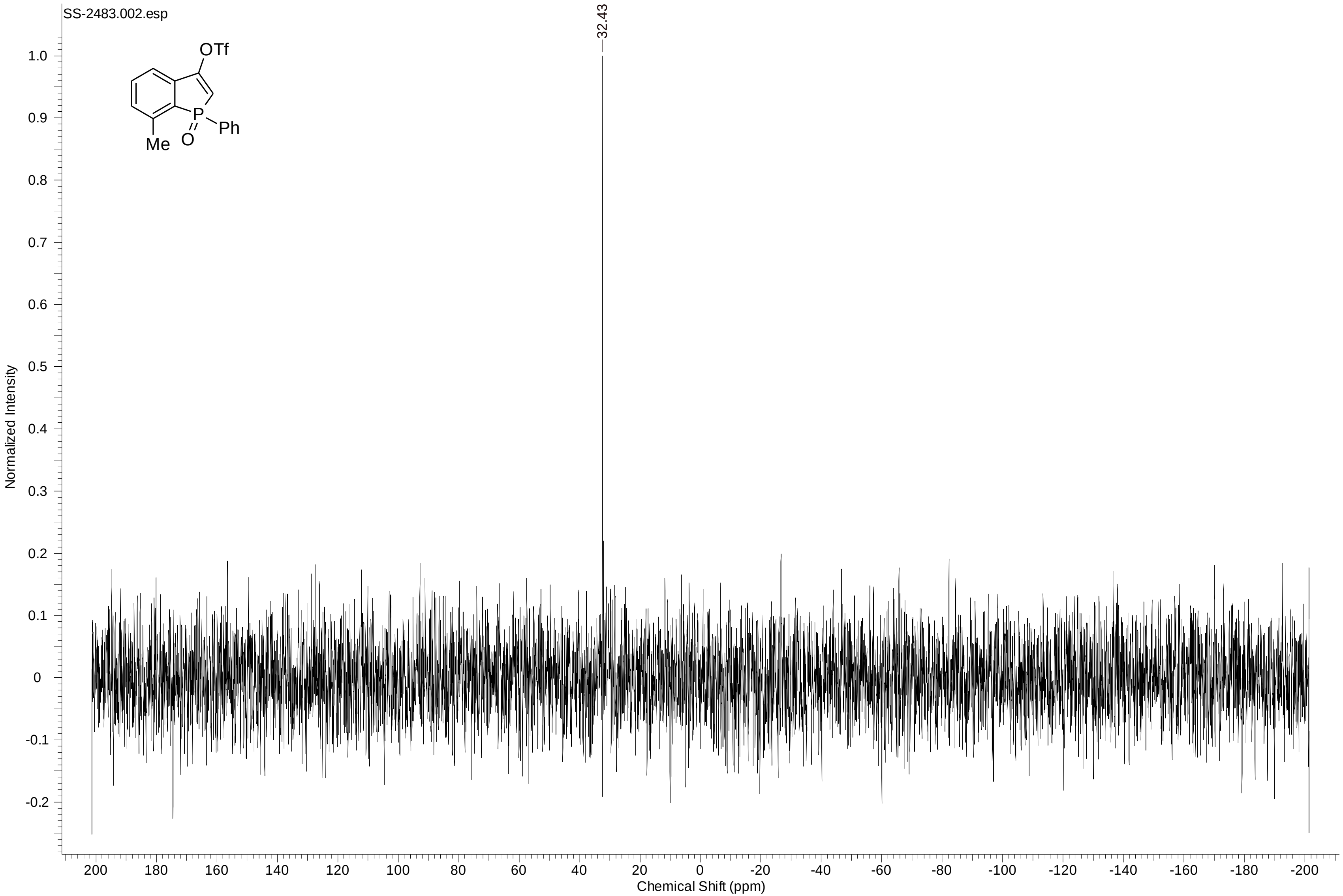

$^{31}P\{^{1}H\}$ NMR spectrum of 1-oxido-1-phenyl-1H-7-methylphosphindol-3-yl trifluoromethanesulfonate (**3b**) (202 MHz, $CDCl_3$)

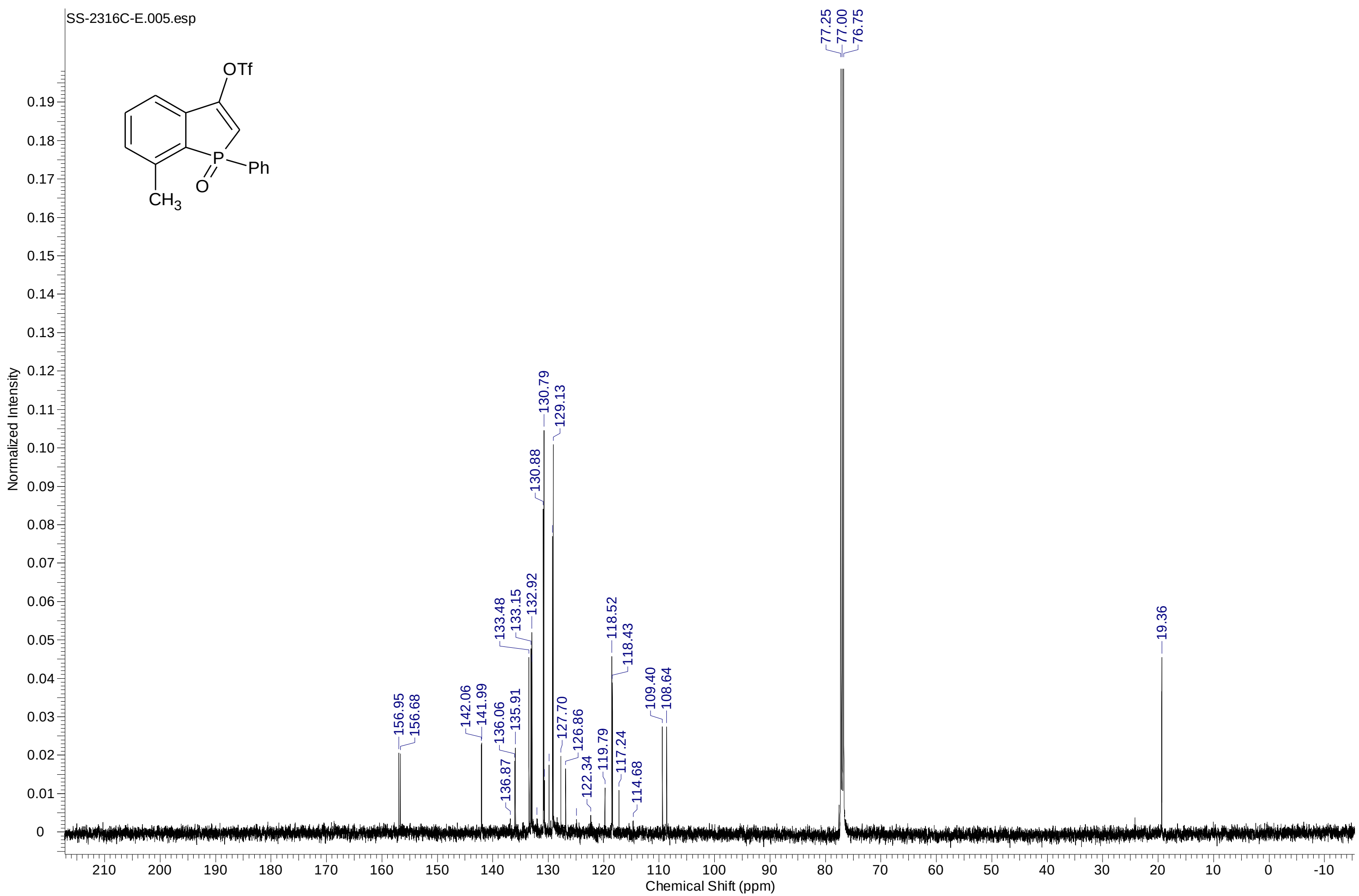

$^{13}C\{^1H\}$ NMR spectrum of 1-oxido-1-phenyl-1H-7-methylphosphindol-3-yl trifluoromethanesulfonate (**3b**) (125 MHz, $CDCl_3$)

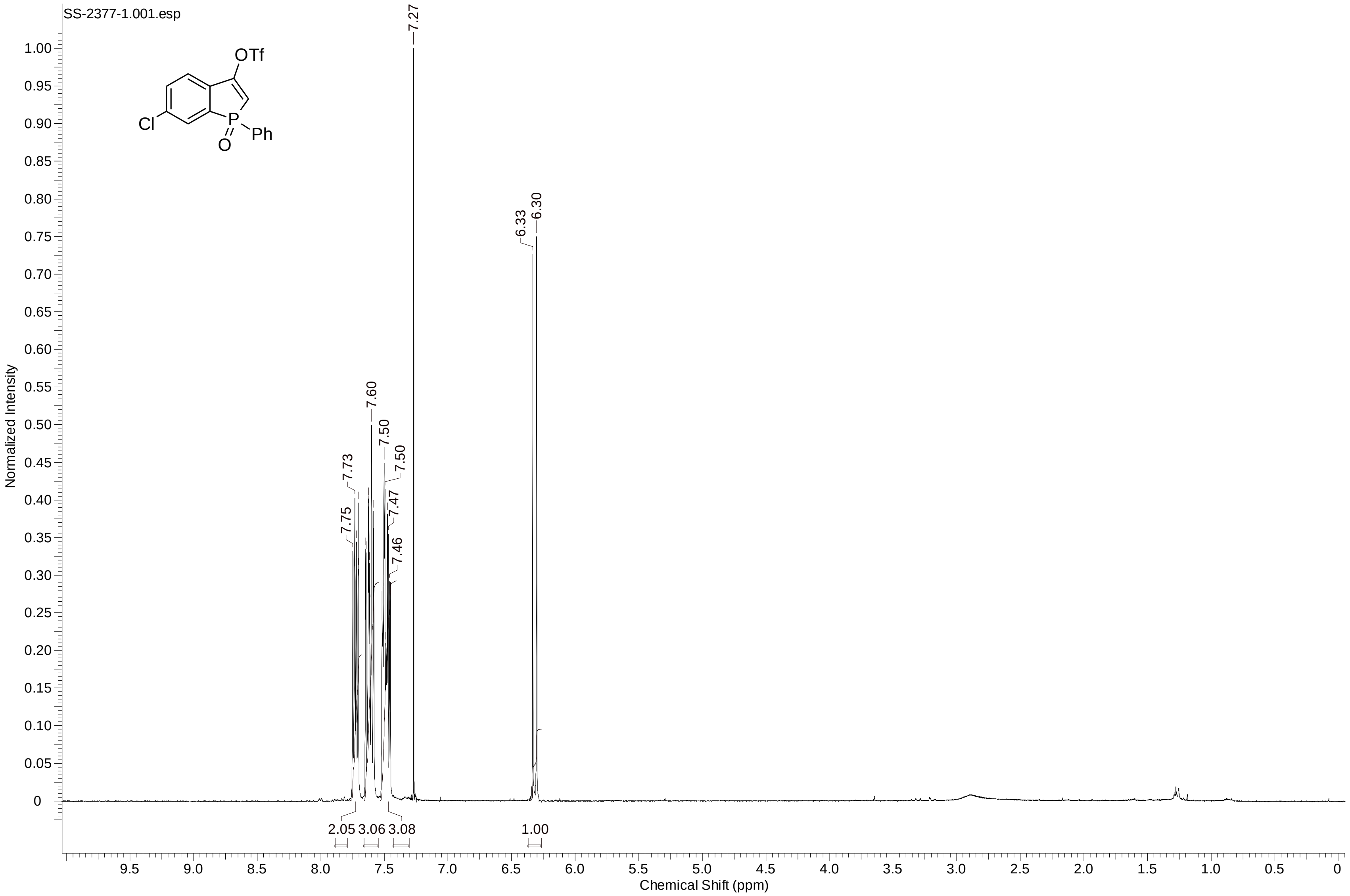

^{1}H NMR spectrum of 1-oxido-1-phenyl-1H-6-chlorophosphindol-3-yl trifluoromethanesulfonate (**3c**) (500 MHz, $CDCl_3$)

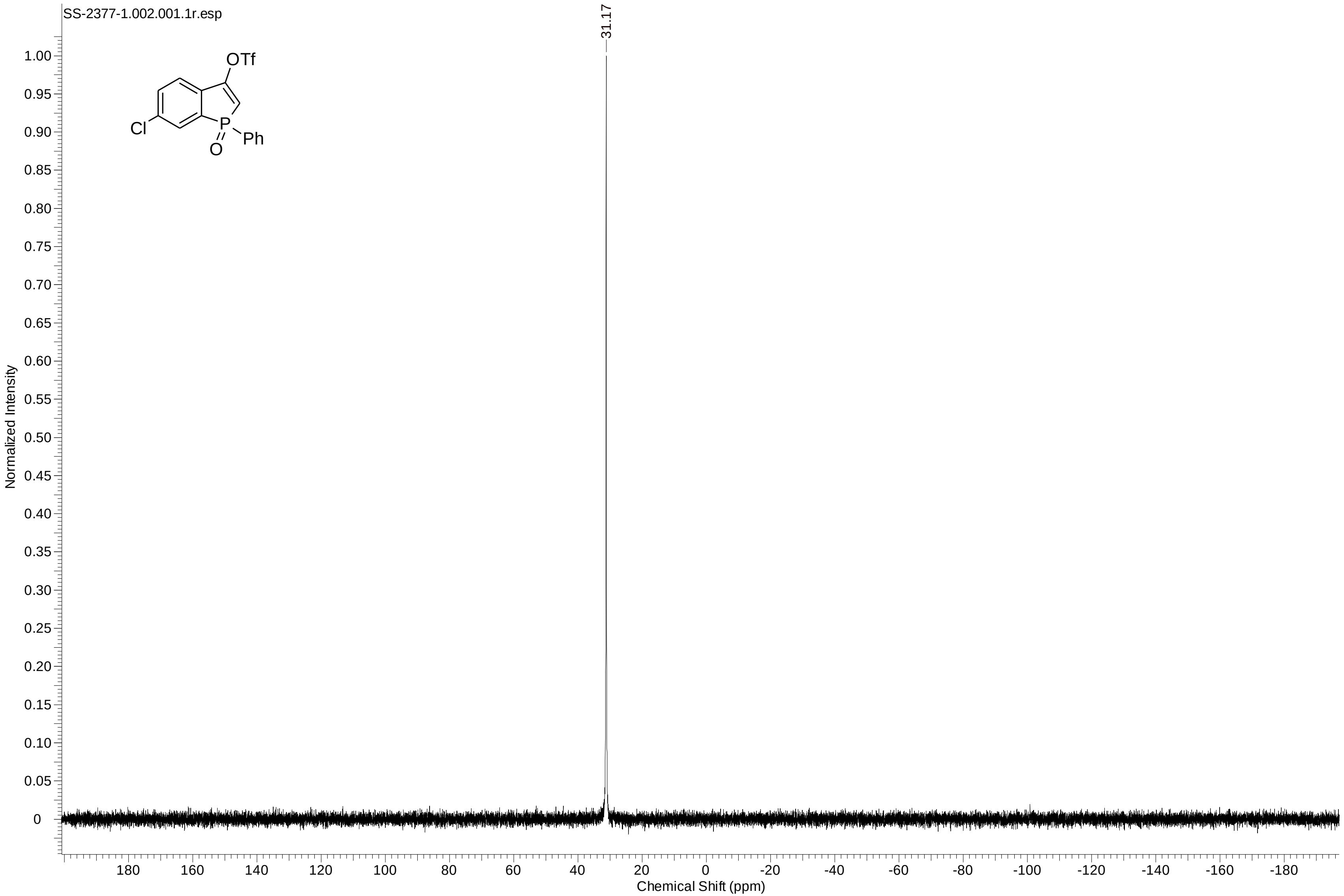

$^{31}P\{^1H\}$ NMR spectrum of 1-oxido-1-phenyl-1H-6-chlorophosphindol-3-yl trifluoromethanesulfonate (**3c**) (202 MHz, $CDCl_3$)

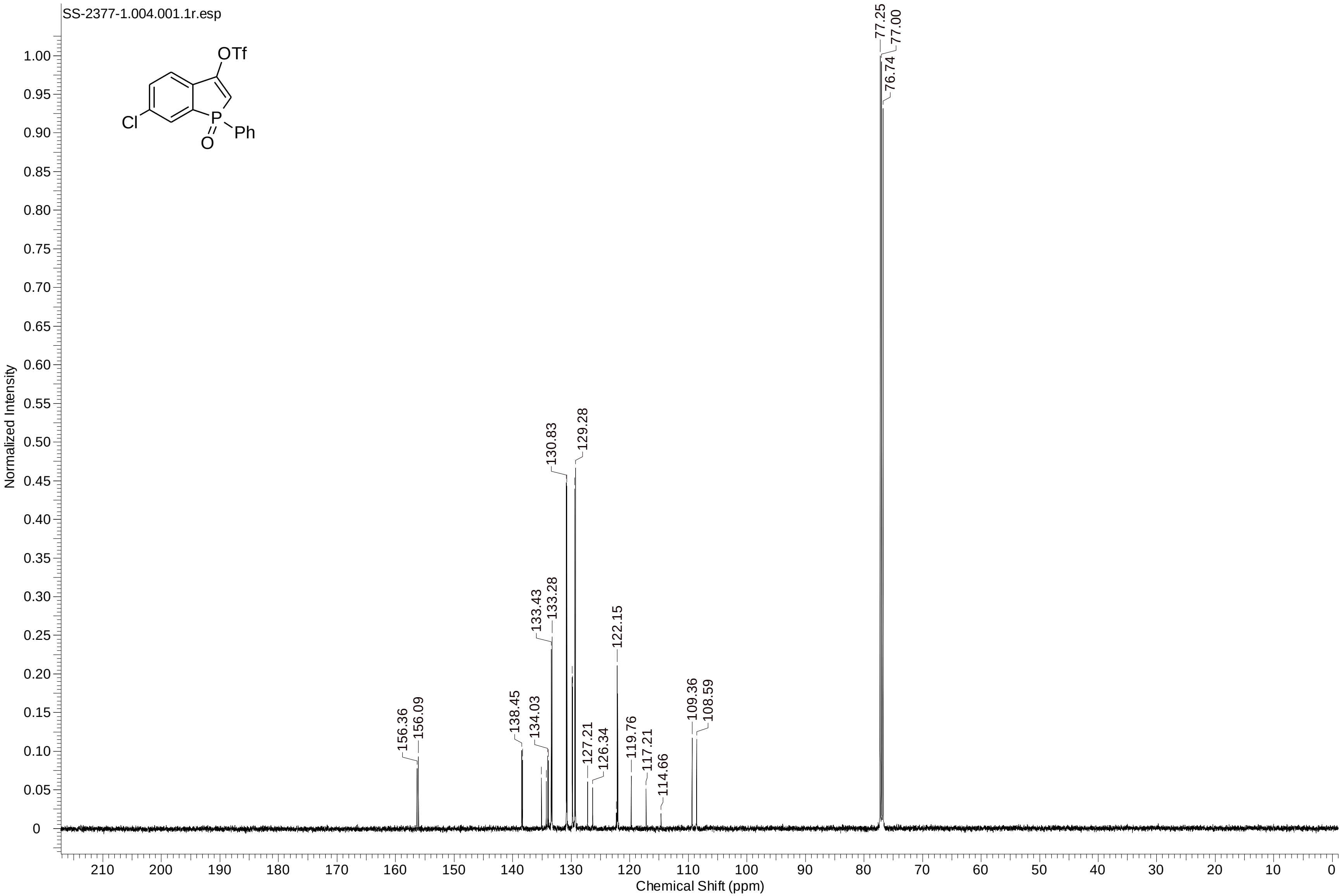

$^{13}C\{^1H\}$ NMR spectrum of 1-oxido-1-phenyl-1H-6-chlorophosphindol-3-yl trifluoromethanesulfonate (**3c**) (125 MHz, $CDCl_3$)

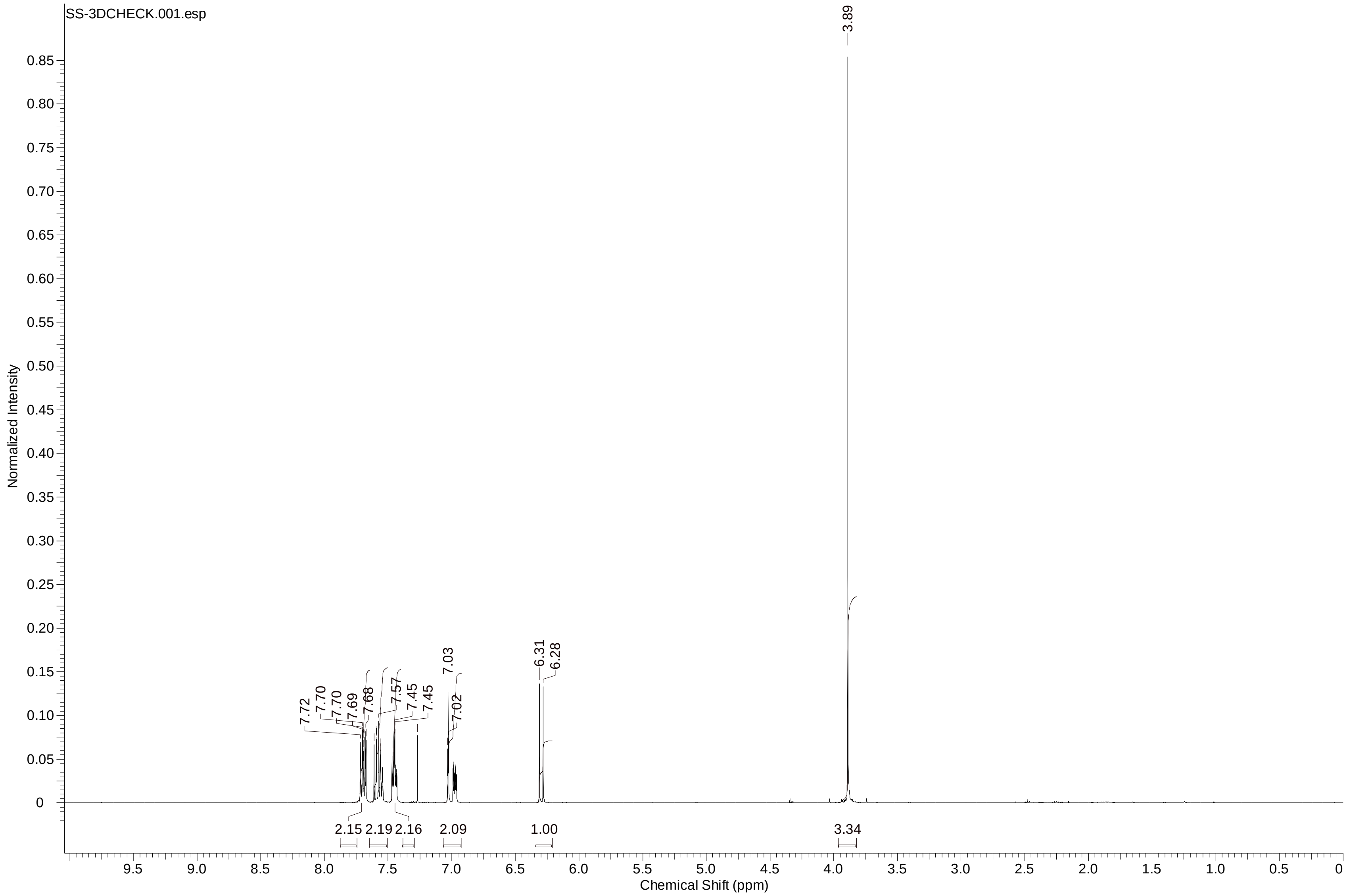

^{1}H NMR spectrum of 1-oxido-1-phenyl-1H-5-methoxyphosphindol-3-yl trifluoromethanesulfonate (**3d**) (500 MHz, $CDCl_3$)

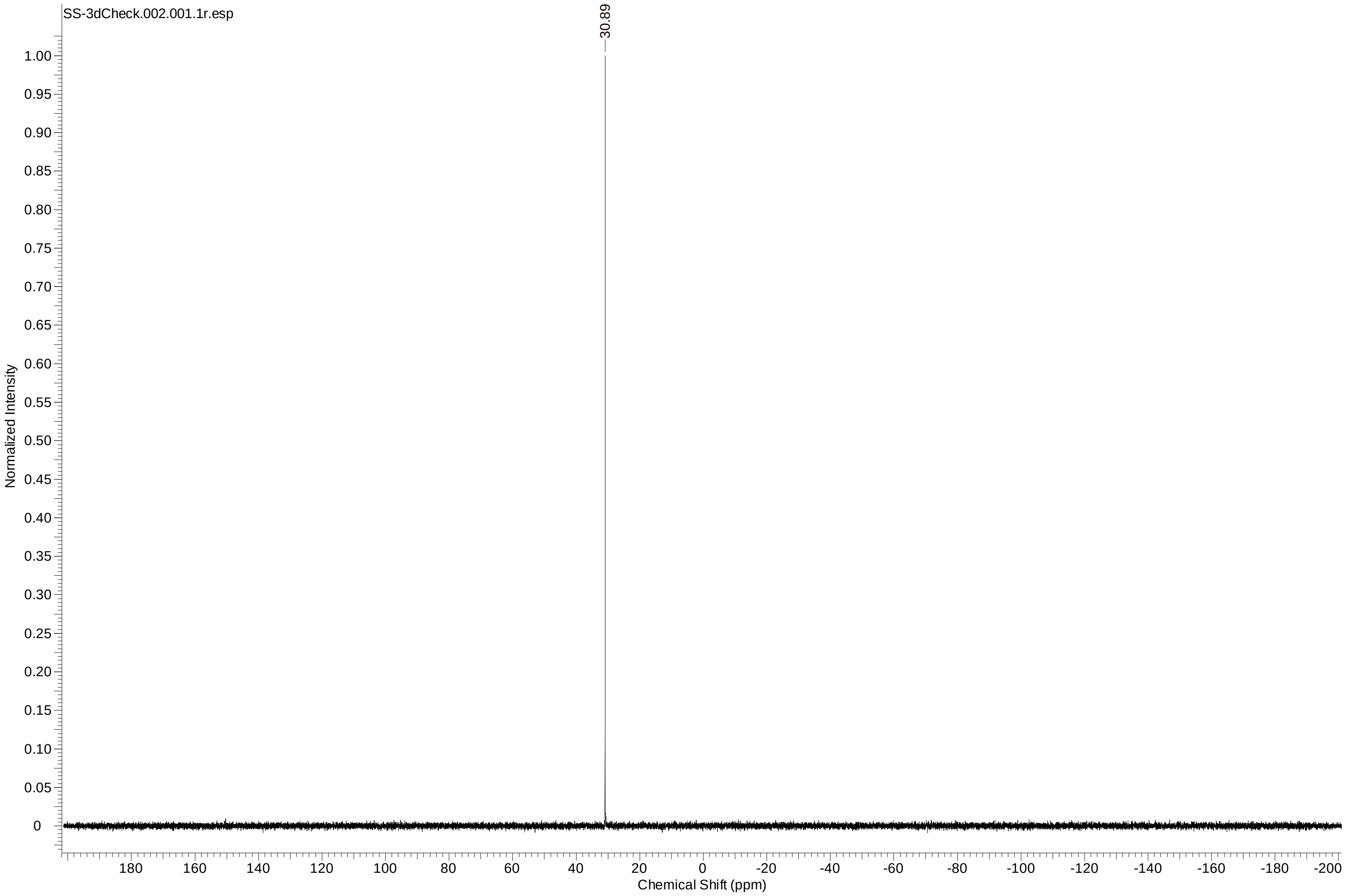

$^{31}P\{^{1}H\}$ NMR spectrum of 1-oxido-1-phenyl-1H-5-methoxyphosphindol-3-yl trifluoromethanesulfonate (**3d**) (202 MHz, $CDCl_3$)

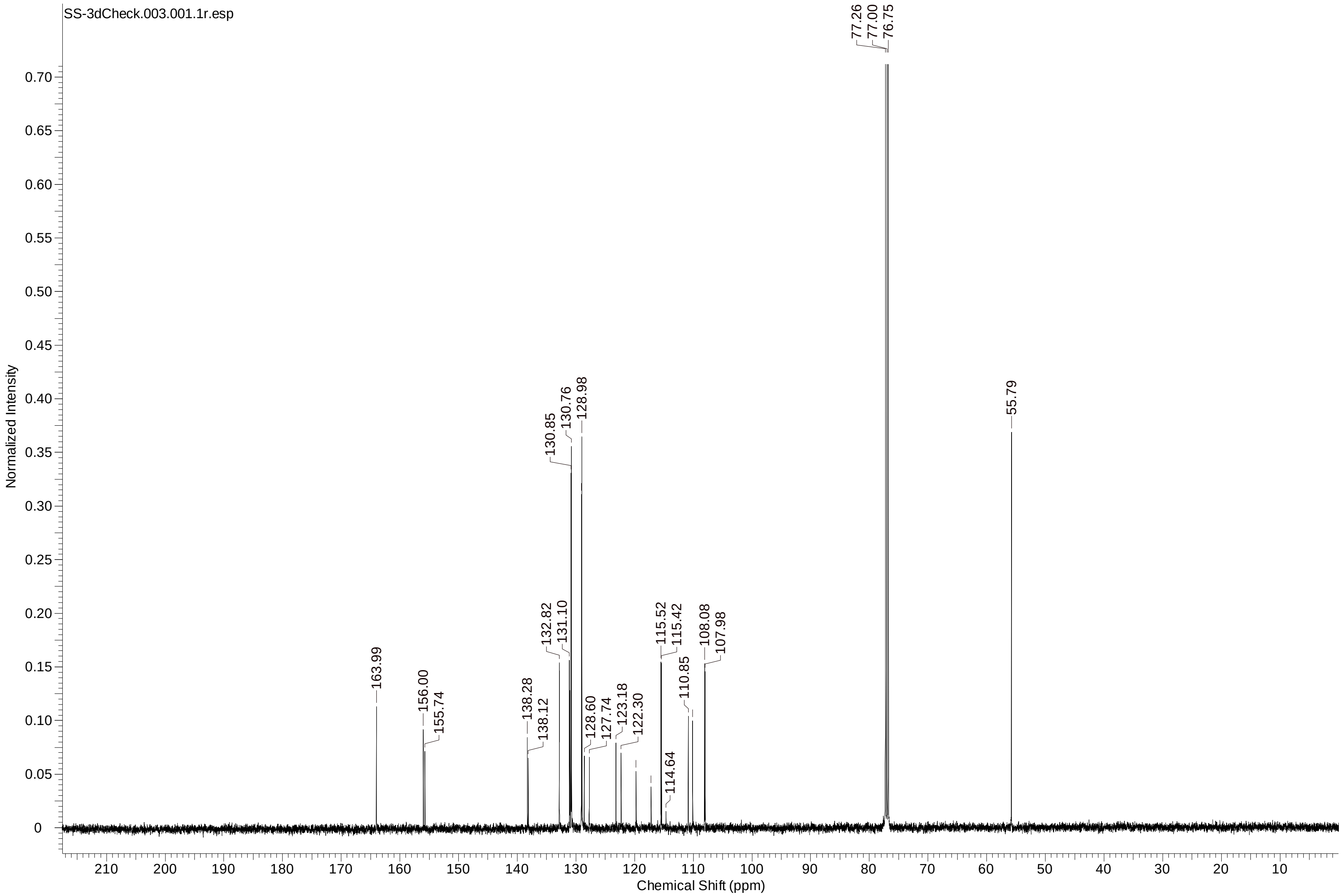

$^{13}C\{^{1}H\}$ NMR spectrum of 1-oxido-1-phenyl-1H-5-methoxyphosphindol-3-yl trifluoromethanesulfonate (**3d**) (125 MHz, $CDCl_3$)

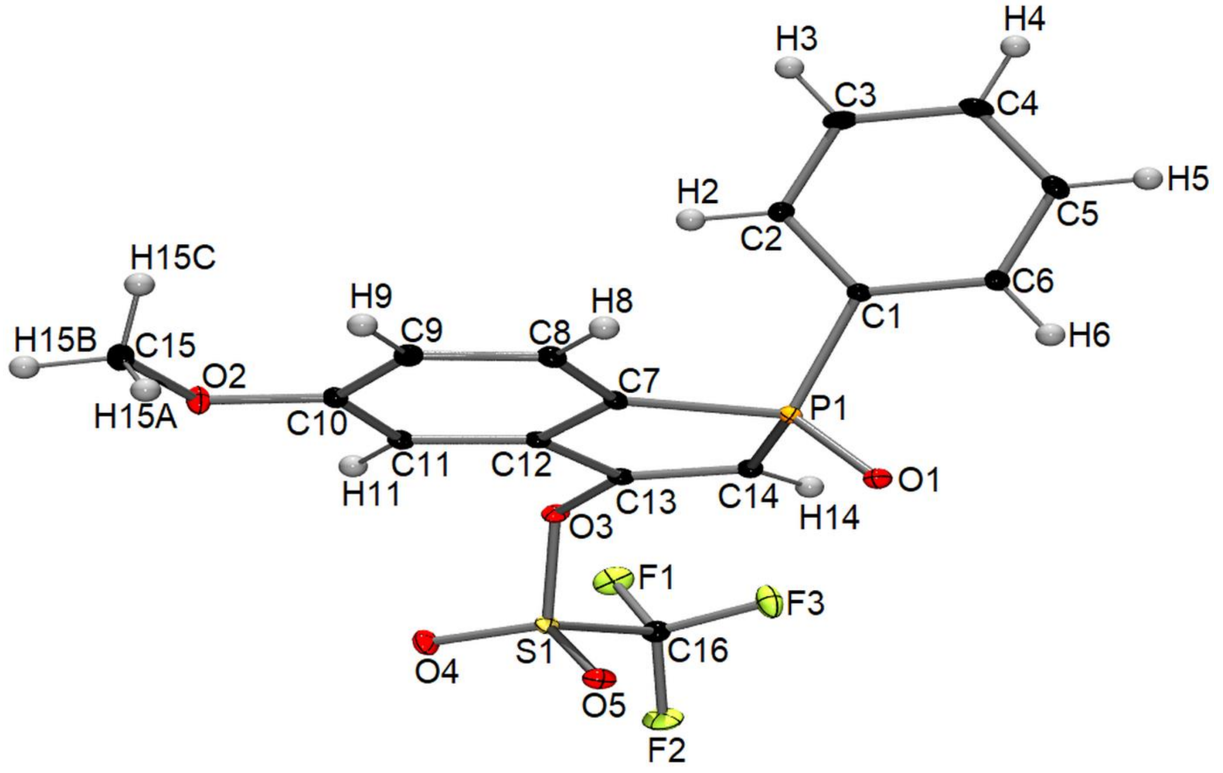

Fig. S1a. Molecular structure of **3d**. Ellipsoids are drawn at 50% probability level, hydrogen atoms are omitted for clarity (except H14 atom). Selected bond distances [Å] and angles [°]: P1-O1 1.4863(13), S1-O3 1.5760(12), S1-O4 1.4141(13), S1-O5 1.4137(14), P1-C1 1.7901(17), P1-C14 1.8119(18), P1-C7 1.7964(17), C13-C14 1.328(2); C1-P1-O1 112.16(7), C1-P1-C14 109.43(8), C1-P1-C7 108.10(7).

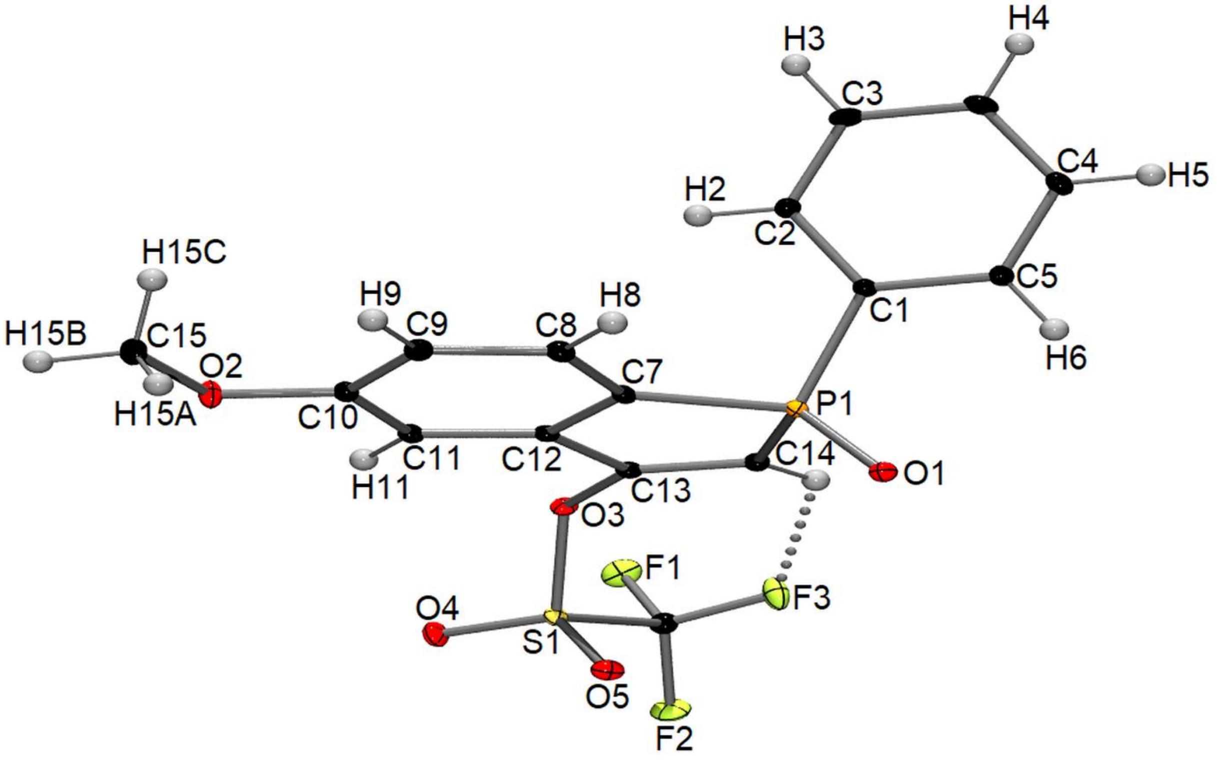

Fig. S1b. Molecular structure of **3d** showing intramolecular bond.

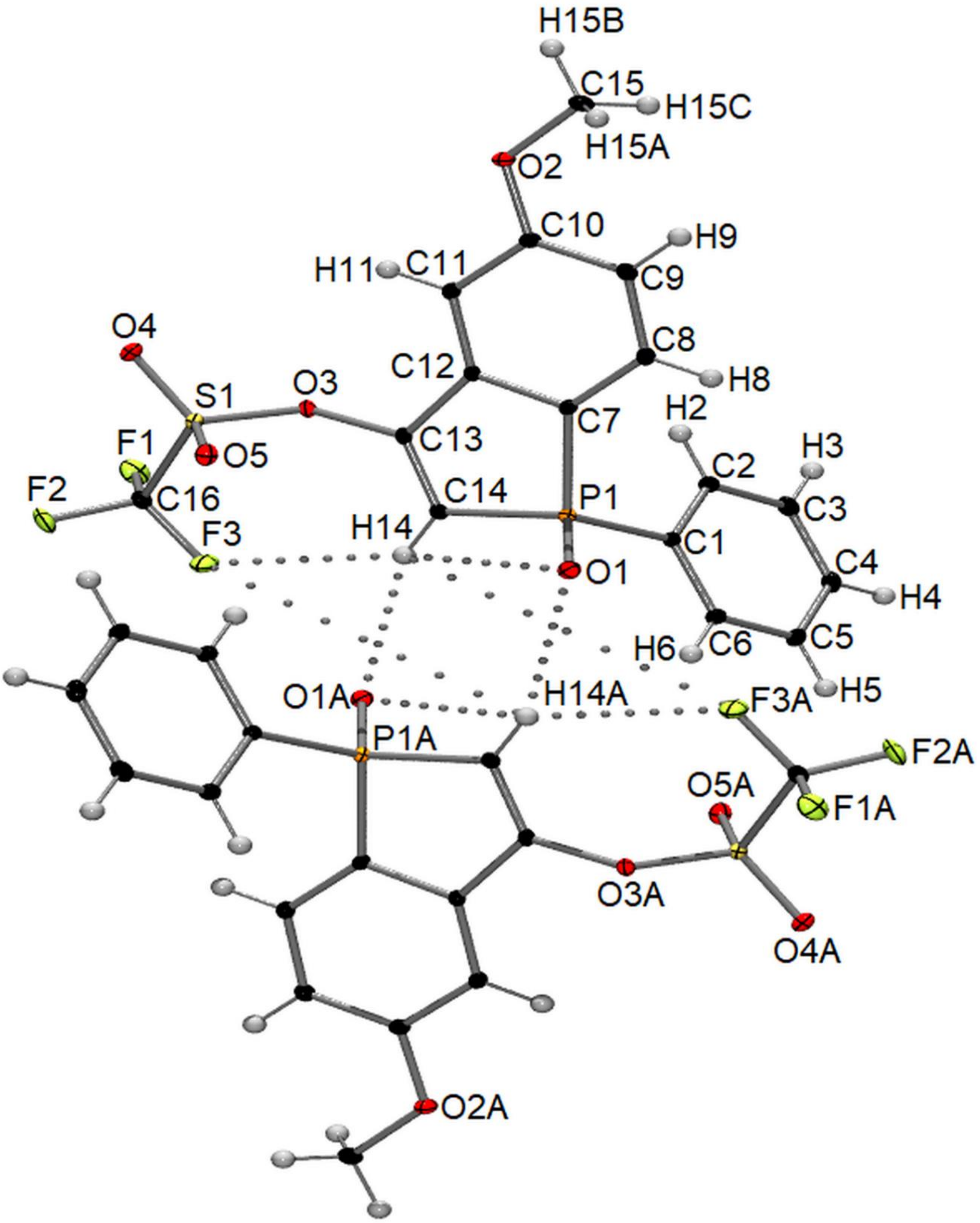

Fig. S1c. Molecular structure of **3d**, showing intra- and intermolecular hydrogen bonds.

Table S1. The intra- and intermolecular hydrogen bonds lengths.

	D-H	H...A	D...A	<(DHA)
C14-H14...F3	0.93	2.58	3.218(2)	126.5
C14-H14...O1$^{(i)}$	0.93	2.47	3.250(2)	141.9
C14-H14...F3	0.93	2.58	3.218(2)	126.5
C14-H14...O1$^{(i)}$	0.93	2.47	3.250(2)	141.9

symmetry operations: (i) -x+1, -y+1, -z.

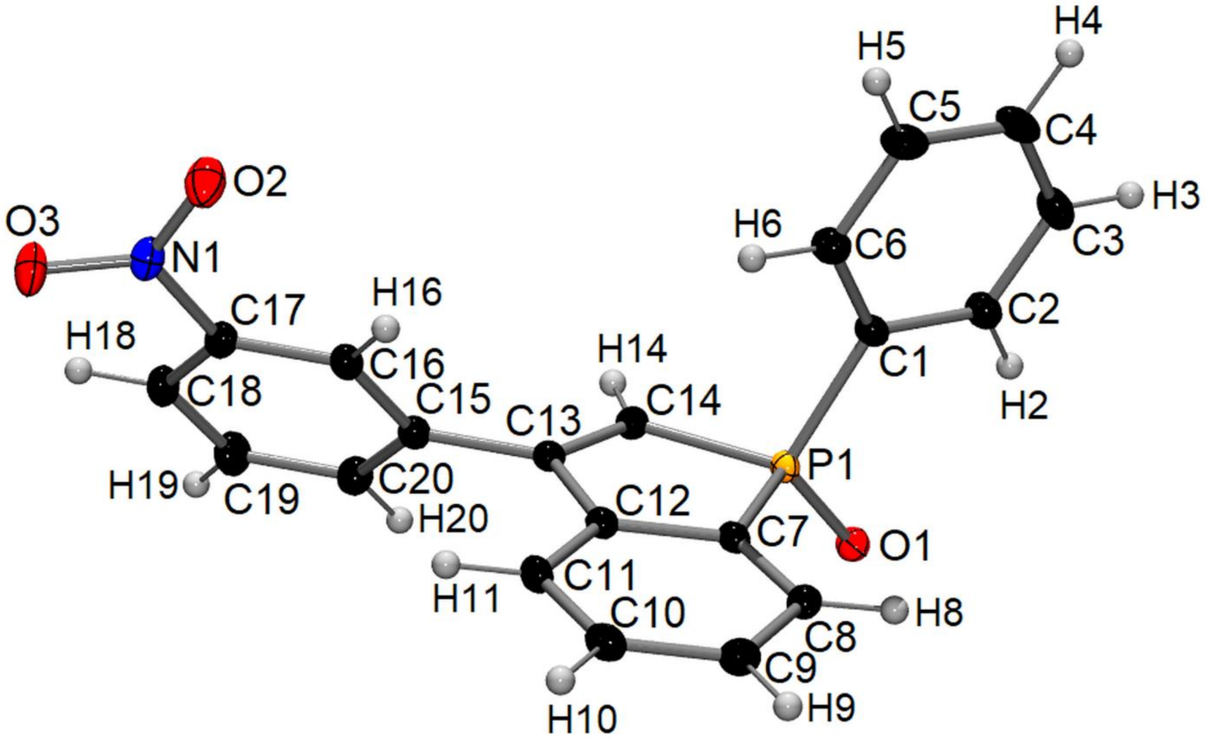

Fig. S2. Molecular structure of **5n**. Ellipsoids are drawn at 50% probability level, hydrogen atoms are omitted for clarity (except H14 atom). Selected bond distances [Å] and angles [°]: N1-O3 1.2241(17), N1-O2 1.2274(19), O1-P1 1.4876(11), P1-C7 1.7996(15), C12-C13 1.488(2), C13-C14 1.346(2), C14-P1 1.7927(15); C1-P1-O1 111.44(7), C1-P1-C14 108.92(7); ΣC13 = 359.99(13)°, ΣC14 = 359.9(12)°.

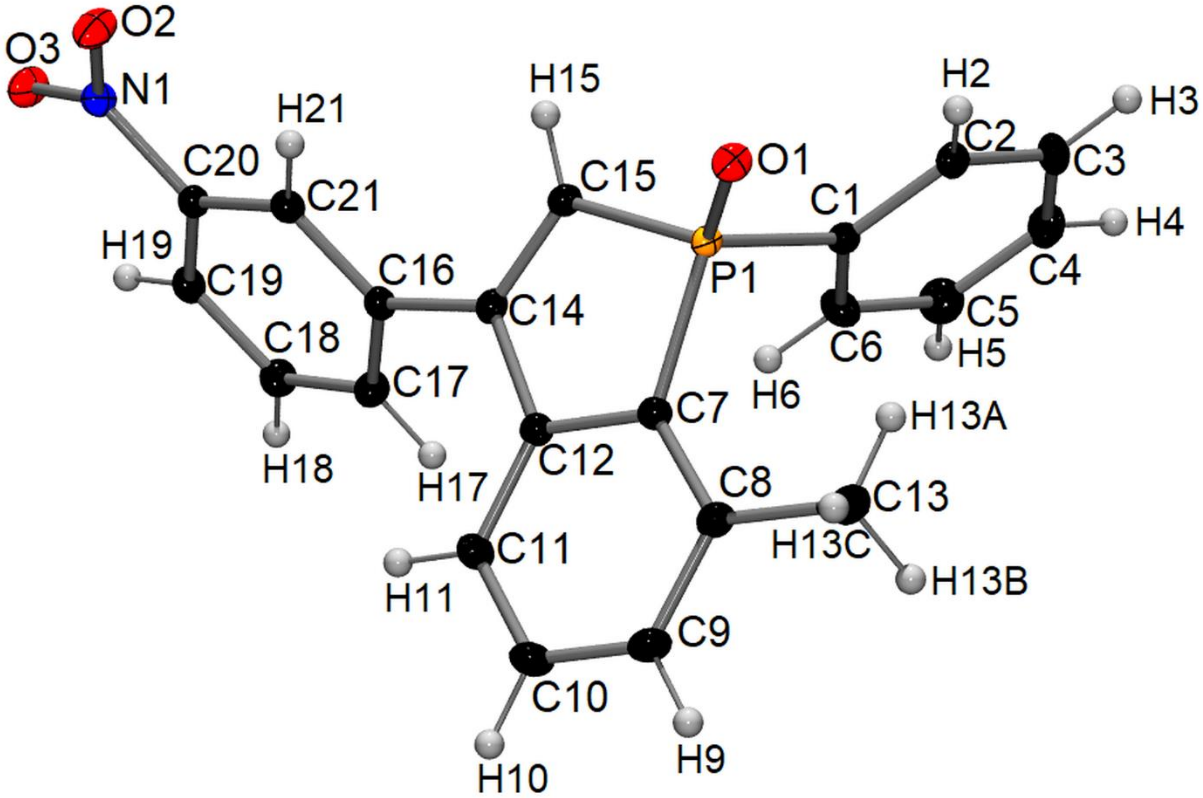

Fig. S3. The molecular structure of **6n**. Ellipsoids are drawn at 50% probability level, hydrogen atoms are omitted for clarity (except H15 atom). Selected bond distances [Å] and angles [°]: N1-O3 1.2357(14), N1-O2 1.2250(14), O1-P1 1.4858(9), P1-C7 1.8051(13), C12-C14 1.4910(17), C14-C15 1.3429(17), C14-P1 1.7940(13); C1-P1-O1 112.56(6), C1-P1-C15 117.94(6); ΣC13 = 359.98(11)°, ΣC14 = 360.0(9)°.

Table S2. Crystallographic data for **3d**, **5n** and **6n**.

	**3d**	**5n**	**6n**
Empirical formula	$C_{16}H_{12}F_3O_5P_1S_1$	$C_{20}H_{14}N_1O_3P_2$	$C_{21}H_{16}N_1O_3P_1$
Formula weight	404.29	347.29	361.32
Radiation source	Mo-*K*α	Mo-*K*α	Mo-*K*α
Wavelength [Å]	0.71073	0.71073	0.71073
Crystal System	monoclinic	monoclinic	monoclinic
Space group	*P*-1	*I*2/*a*	*P*21/*n*
a [Å]	8.5548(8)	19.7919(6)	10.5350(5)
b [Å]	10.2265(13)	8.2804(3)	8.4270(3)
c [Å]	11.1571(10)	20.1889(7)	19.8830(9)
α [°]	98.825(9)	90	90
β [°]	105.175(7)	95.531(3)	96.113(4)
γ [°]	113.024(8)	90	90
V [$Å^3$]	830.28	3293.25(19)	1755.14(13)
Z	120	8	4
Calculated Density [$g{\cdot}cm^{-1}$]	1.617	1.401	1.367
T [K]	120(2)	120(2)	120(2)
μ [mm^{-1}]	0.349	0.186	0.177
Theta range for data collection [°]	2.26-29.58	2.66-29.60	2.29-29.59
Index ranges	-11<=h<=11<br>-14<=k<=13<br>-14<=l<=15	-20<=h<=26<br>-10<=k<=10<br>-26<=l<=26	-14<=h<=13<br>-10<=k<=11<br>-27<=l<=27
Data / restraints / parameters	4424/0/236	3973/0/226	4729/0/236
Goodness-of-fit on F^2	1.025	1.066	1.107
Final R indices	0.0384	0.0386	0.0378
[*I*>2σ(I)]	0.0891	0.0978	0.1044
R indices (all data)	0.0508	0.0524	0.049
[*I*>2σ(I)] (all data)	0.9055	0.1032	0.1079
Largest diff. peak and hole [e.$Å^{-3}$]	0.529 and -0.422	0.361 and -0.423	0.463 and -0.466
CCDC	2247935	2190931	2190932

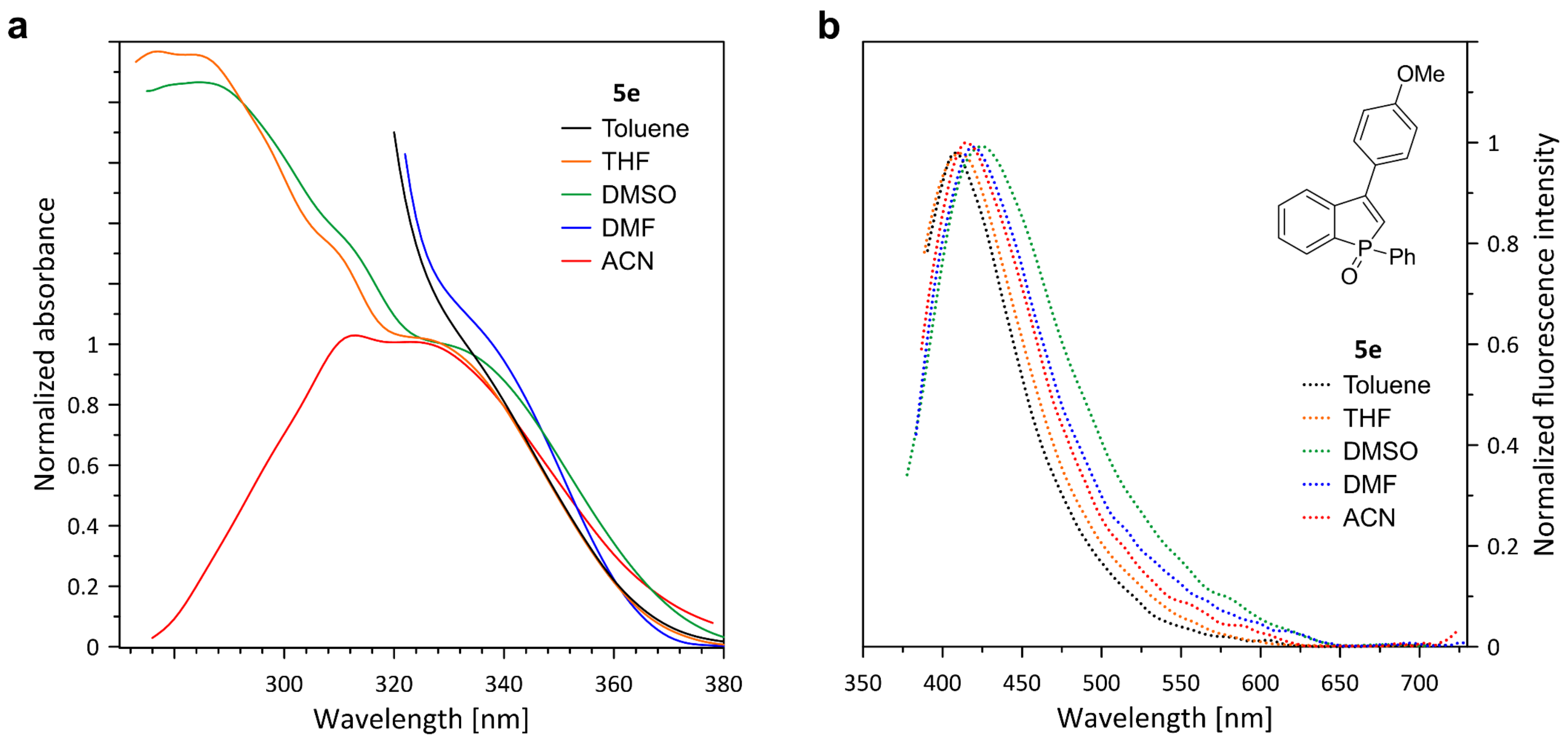

Fig. S4. a) Normalized absorption spectra of **5e** in different solvents at 10^{-5} M. b) Normalized fluorescence spectra of **5e** in different solvents at 10^{-5} M. Excitation wavelength: $\lambda_{abs.}$

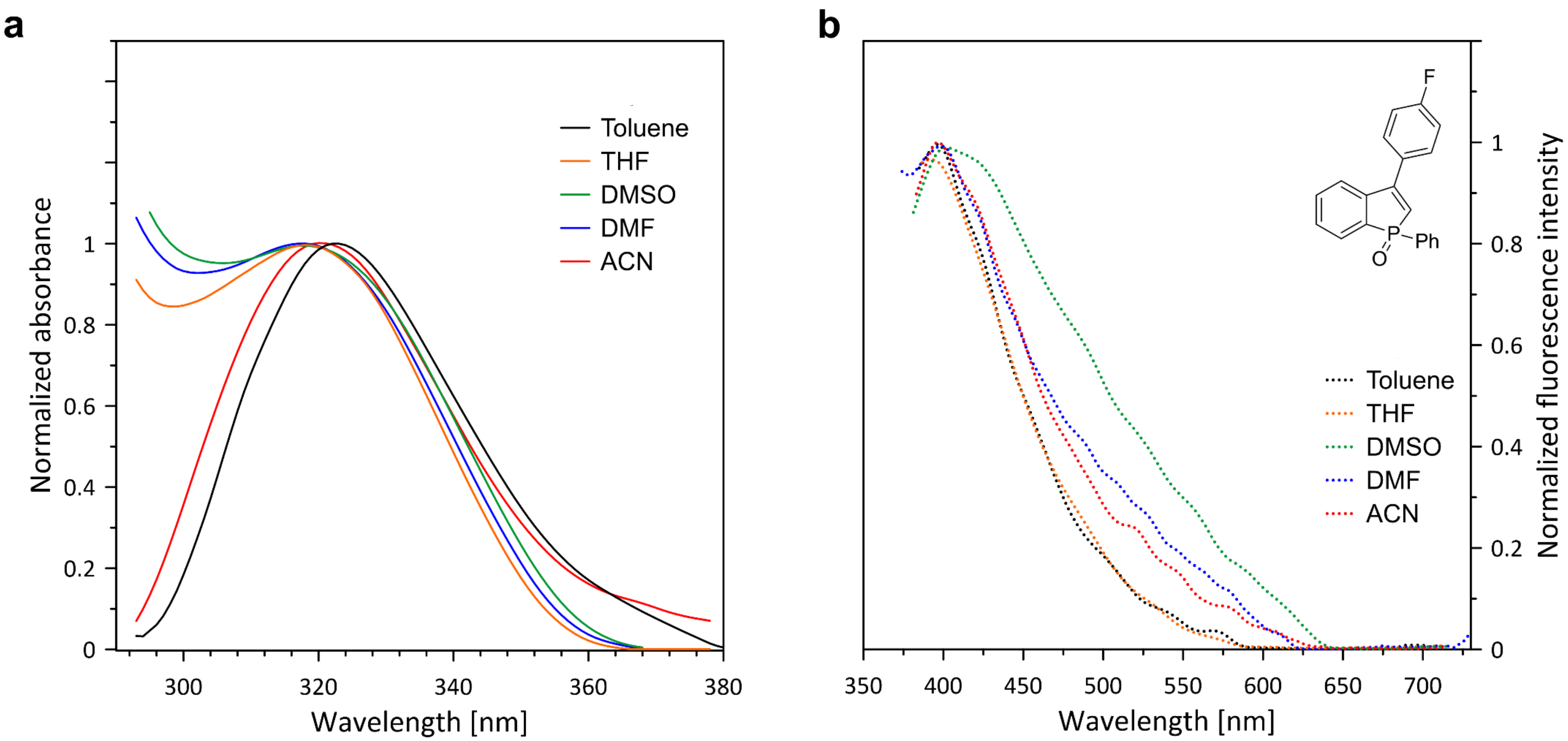

Fig. S5. a) Normalized absorption spectra of **5j** in different solvents at 10^{-5} M. b) Normalized fluorescence spectra of **5j** in different solvents at 10^{-5} M. Excitation wavelength: $\lambda_{abs.}$

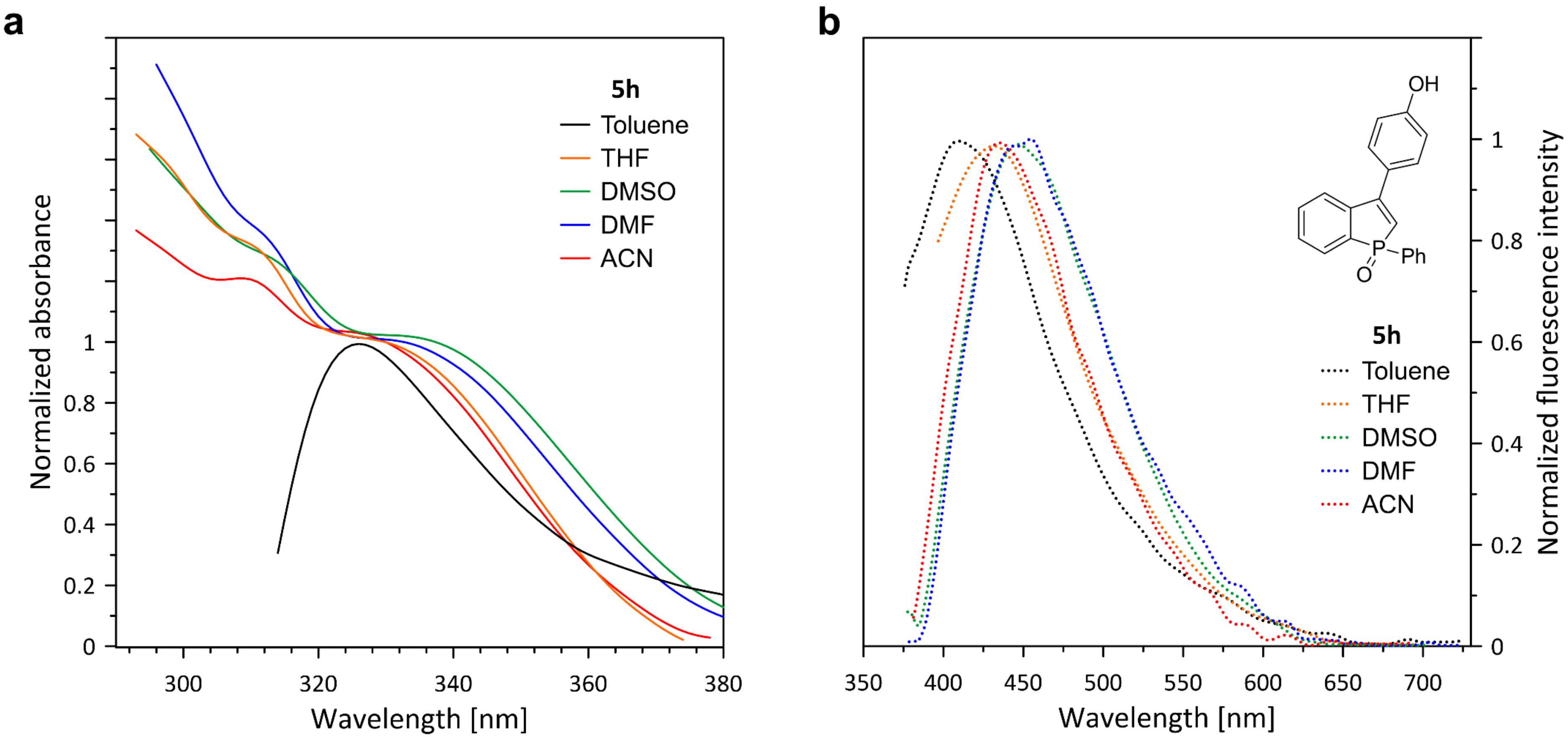

Fig. S6. a) Normalized absorption spectra of **5h** in different solvents at 10^{-5} M. b) Normalized fluorescence spectra of **5h** in different solvents at 10^{-5} M. Excitation wavelength: $\lambda_{abs.}$

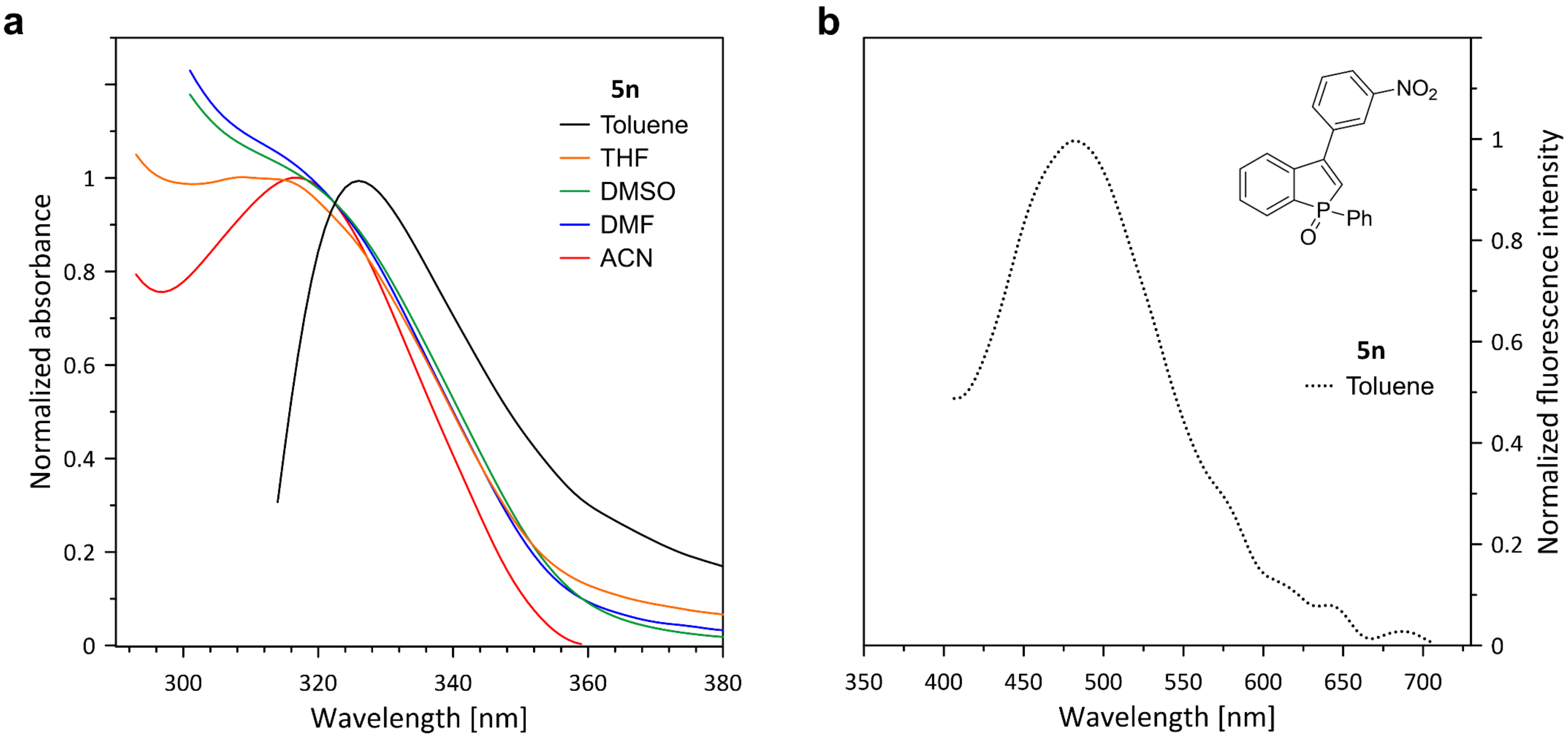

Fig. S7. a) Normalized absorption spectra of **5n** in different solvents at 10^{-5} M. b) Normalized fluorescence spectra of **5n** in different solvents at 10^{-5} M. Excitation wavelength: $\lambda_{abs.}$

The orientation polarizability of solvent (Δf) is defined as

$$\Delta f = \frac{\varepsilon - 1}{2\varepsilon + 1} - \frac{n^2 - 1}{2n^2 + 1}$$

where: ε - dielectric constant (at 20°C), η - index of refraction (at 20°C).[4] The Stokes shift ($\Delta\nu = (\nu_A - \nu_F)$[cm^{-1}], ν_A - absorption band expressed in wavenumbers, ν_F - maximum of the fluorescence spectrum expressed in wavenumbers) was plotted for following values of the orientation polarizability of solvents: Toluene: 0.013, THF: 0.210, DCM: 0.217, DMSO: 0.263, DMF: 0.274, ACN: 0.305.

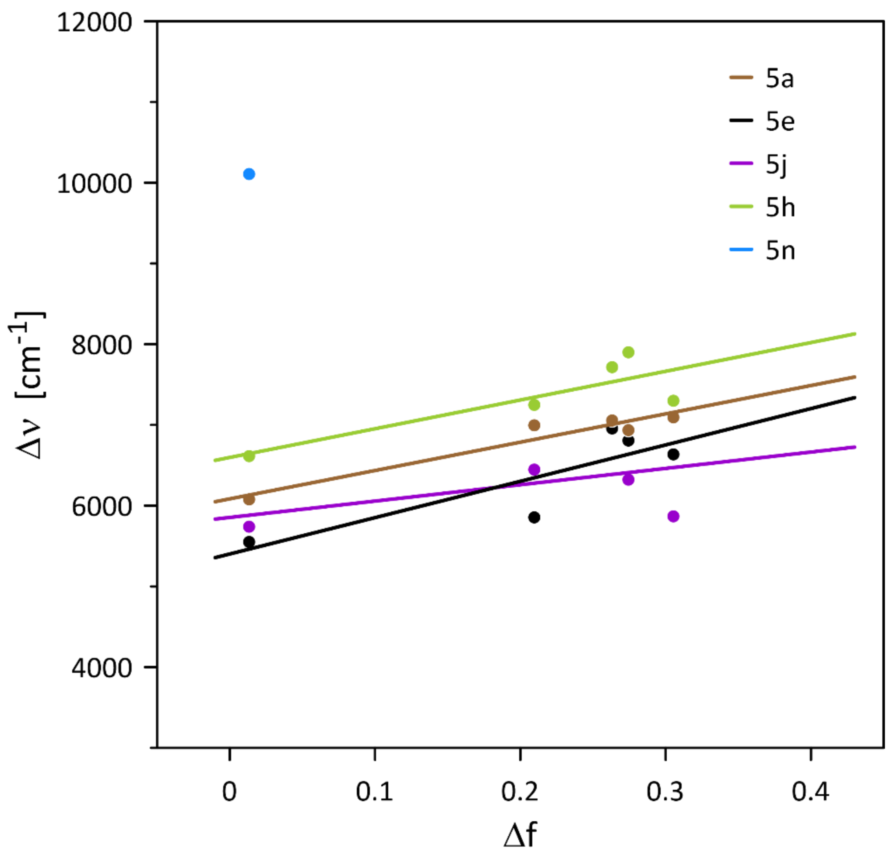

Fig. S8. Lippert-Mataga plot for **5a,e,j,h,n.**

5a
Equation Y = 3509.674838 * X + 6084.498571
Coefficient of determination, $\mathbf{R^2 = 0.93}$

5e
Equation Y = 4502.89773 * X + 5401.57136
Coefficient of determination, $\mathbf{R^2 = 0.72}$

5j

Equation Y = 2024.656287 * X + 5854.299303
Coefficient of determination, $\mathbf{R^2 = 0.20}$

5h
Equation Y = 3557.939539 * X + 6597.261886
Coefficient of determination, $\mathbf{R^2 = 0.70}$

5n
-

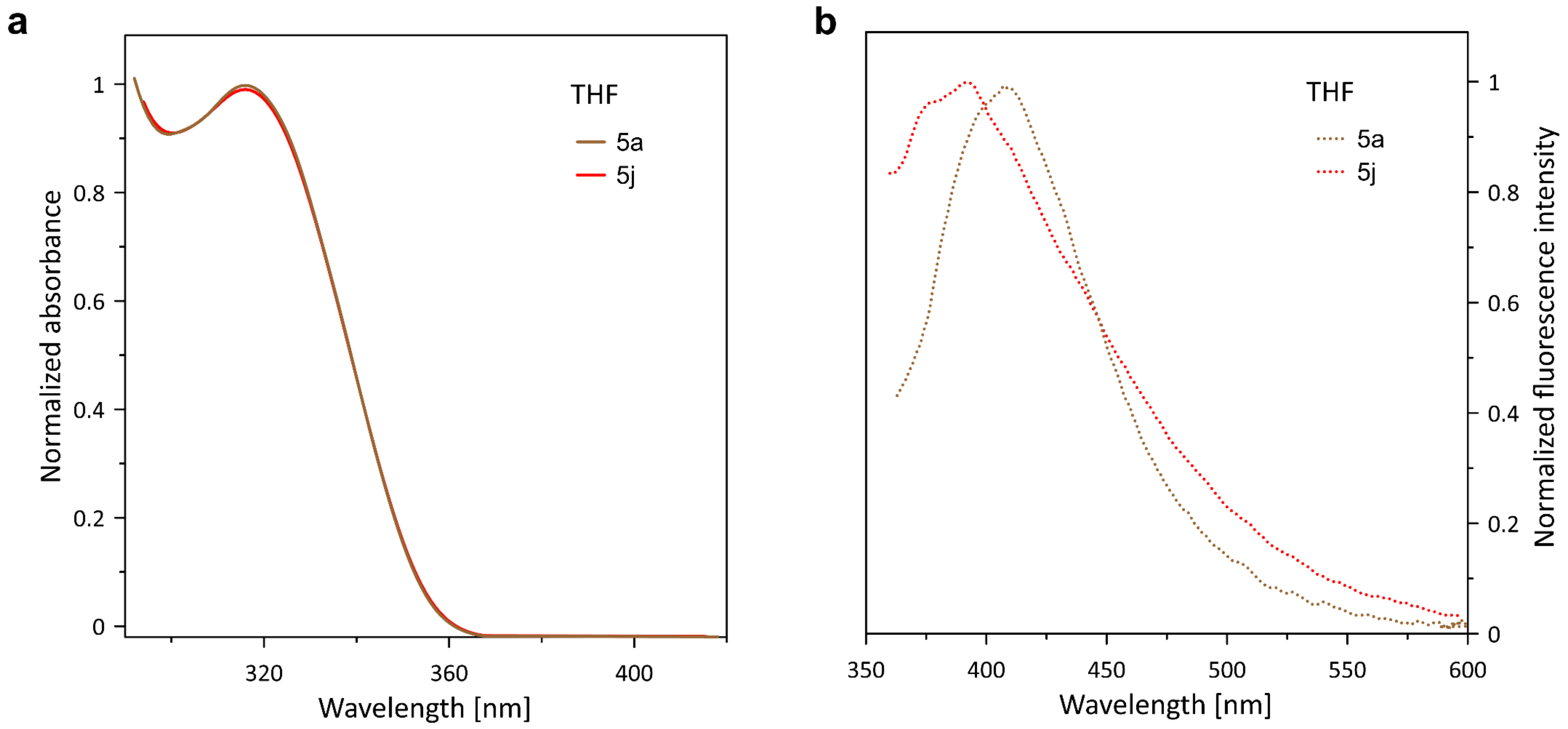

Fig. S9. a) Normalized absorption spectra of **5a,j** in THF at 10^{-4} M. b) Normalized fluorescence spectra of **5a,j** in THF at 10^{-4} M. Excitation wavelength: $\lambda_{abs.}$

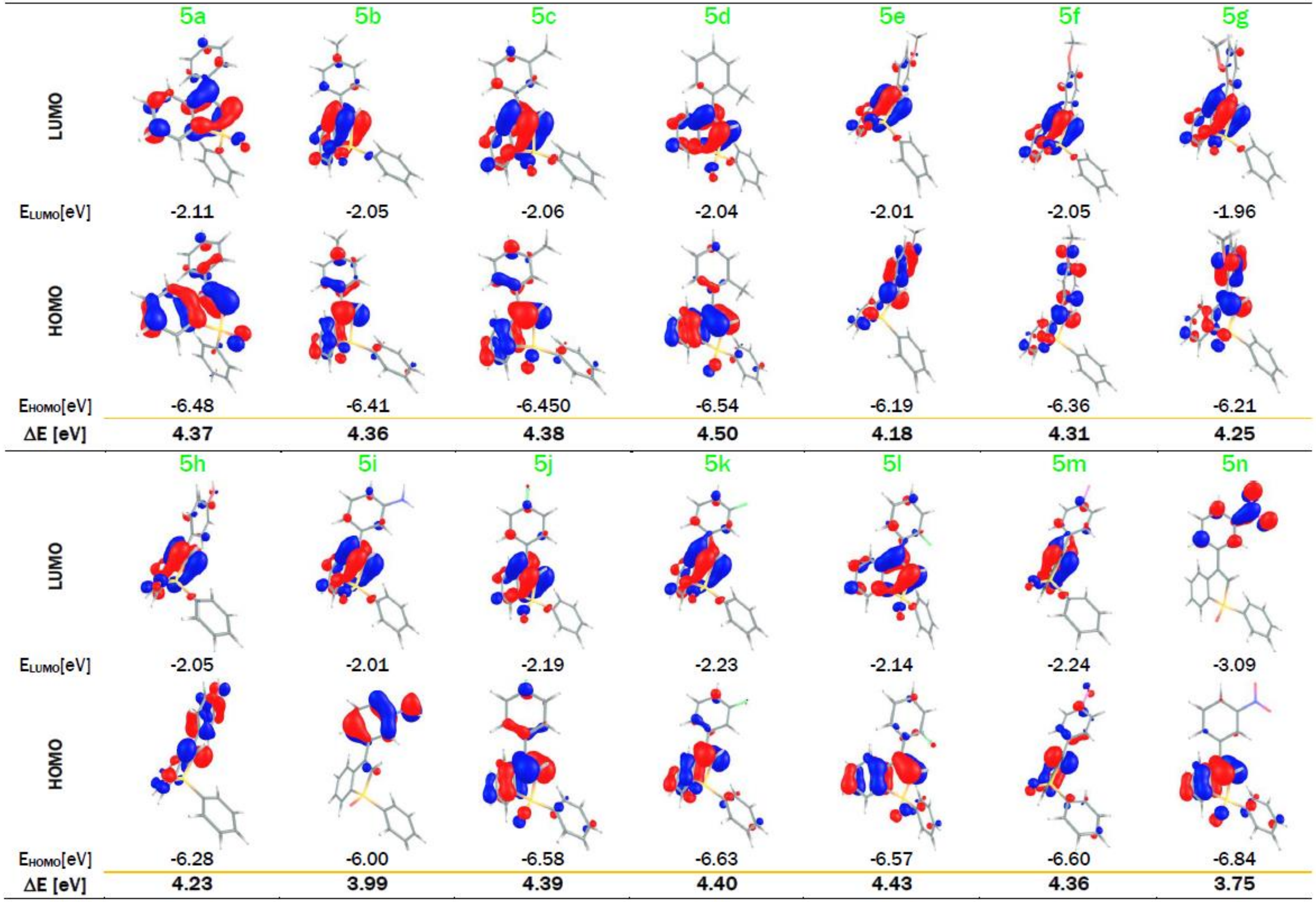

Fig. S10 The HOMO-LUMO frontier molecular orbitals for **5a-n** obtained at DFT/B3LYP/6-31+G(d,p) level.

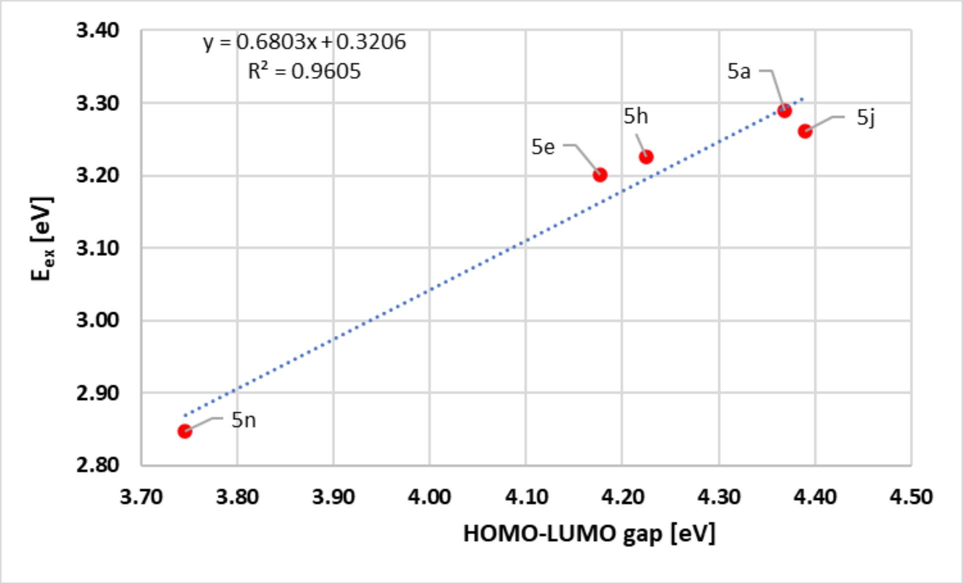

Fig. S11 The correlation diagram (H-L gap vs. excitation energy).

Table S3. The calculation of energy gap from excitation energy (S_1-S_0) (TD DFT/B3LYP).

**Comp.**	**HL GAP (DFT) [eV]**	**E_{S0} [au]**	**E_{S1} [au]**	**Eex [eV] (E_{S1}-E_{S0})**
**5a**	4.37	-1187.810486	-1187.689454	3.29
**5e**	4.18	-1302.340103	-1302.222449	3.20
**5h**	4.23	-1263.036624	-1262.918086	3.23
**5j**	4.39	-1287.050460	-1286.930636	3.26
**5n**	3.75	-1392.317979	-1392.213313	2.85

The excitation energy is calculated as the energy difference between the S_1 and S_0 states (Table S3). The data showed a similar effect of the substitution pattern on the excitation energies as the DFT calculations. Correlation of the energy gap with the excitation energies is done by the method of least squares (linear regression) (Fig. S11). Since we obtained the dependence described in the form of an equation, we additionally determined the parameter R^2. (The R^2 factor is a statistical measure of how well the regression predictions approximate the actual data points.) Our correlation diagram showed a high correlation linearity ($R^2 = 0.96$), indicating that the regression predictions fit the data perfectly.

The HOMO and LUMO levels are modulated slightly by the character of the substituent in the phenyl ring. For *p*-substituted analogues, increasing donor strength of the substituent (**5e,g,h**) result in higher values of HOMO and LUMO levels than reference compound **5a** and the presence of deactivating substituents (**5j,m**) give the opposite effect (Fig. S12).

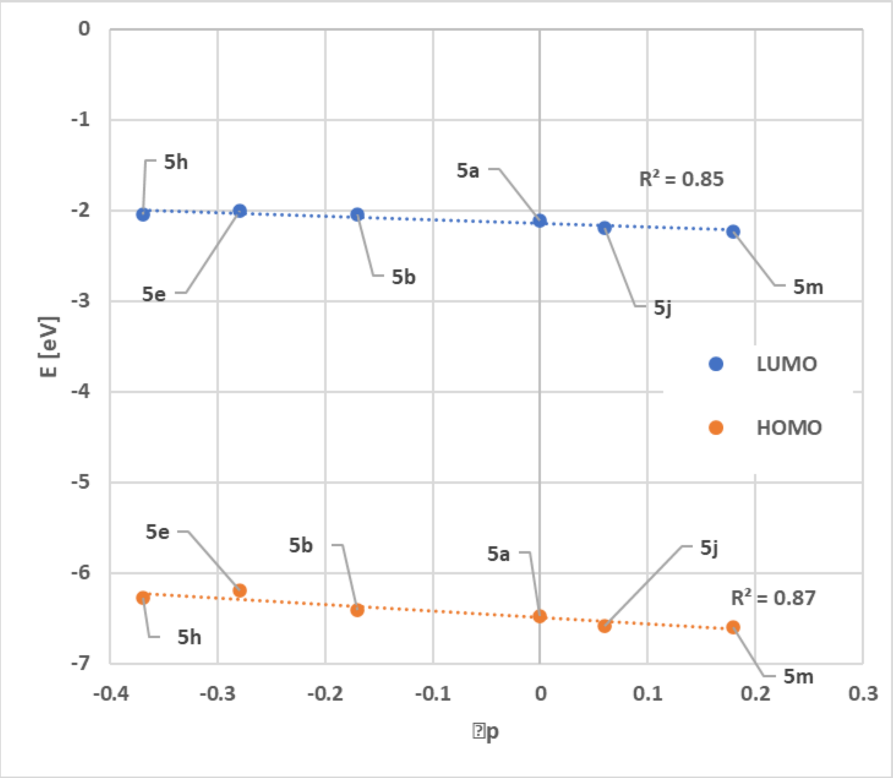

Fig. S12. HOMO (orange) and LUMO (blue) energies of the **5** compound series vs. Hammet's σ_P of para-substituent.

Table S4. The experimental and computed UV-Vis parameters and electronic transitions for **5a,e,h,j,n** compounds.

Structure	Experiment	Theory			
	**λ [nm]**	**λ [nm]**	**f**	**Major contribution**	
**5a**	316	331	0.0381	H→L	93%
**5e**	330	354	0.1555	H→L	98%
	305	308	0.1284	H-1→L	96%
**5h**	330	349	0.1276	H→L	97%
	305	305	0.1347	H-1→L	96%
**5j**	316	331	0.0666	H→L	97%
**5n**	315	329	0.0367	H→L+1	90%
	-	-	0.0045	H→L	99%

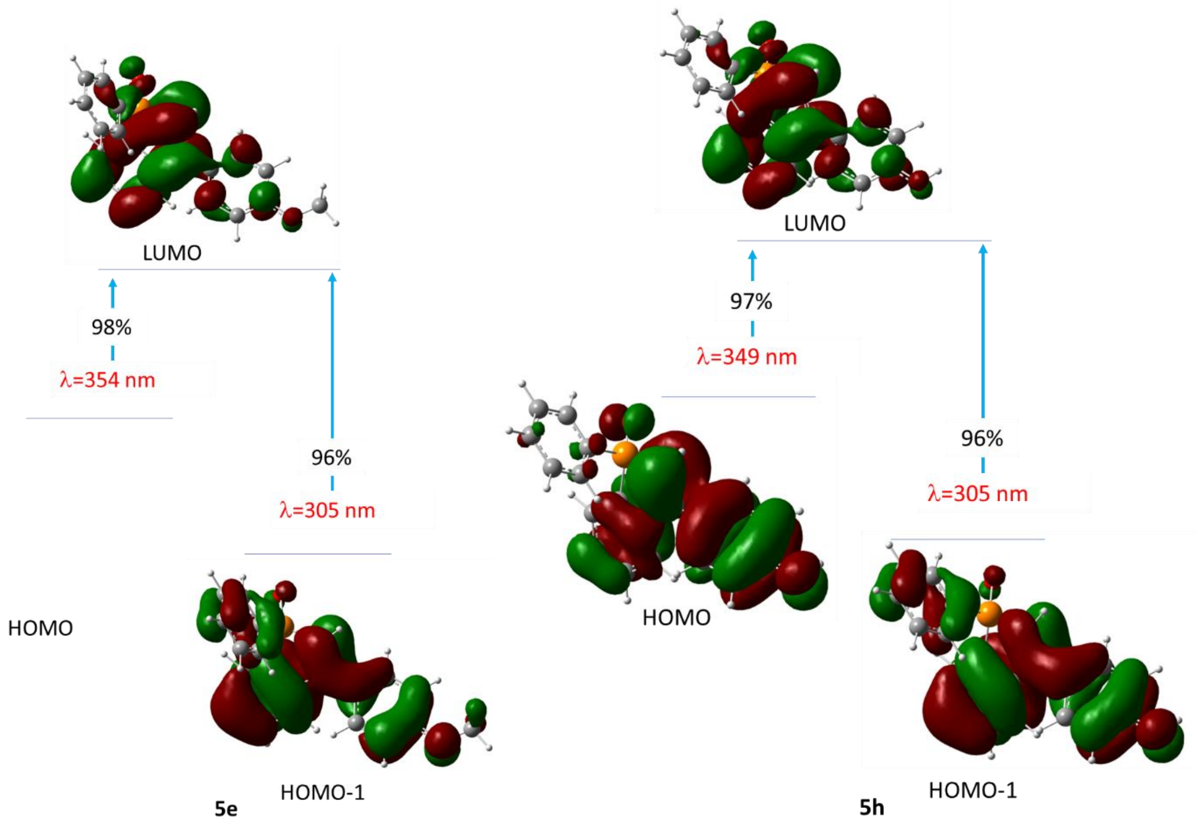

Fig. S13. Calculated distribution patterns of the HOMOs-1, HOMOs and LUMOs of **5e** and **5h** at the ground states in gas phase and the transitions related to most probable absorption peaks.

Table S5. The comparison of dipole moments of S_0 and S_1 states for **5a,e,h,j,n.**

Compound	Dipole moment [D]	
	S_0	S_1
**5a**	4.98	4.16
**5e**	4.85	8.28
**5h**	4.18	6.38
**5j**	4.40	4.97
**5n**	4.88	20.94

Table S6. Geometries of S_0 vs S_1 state for **5a,e,h,j,n**. Selected bond lengths, interatomic distances (Å) and the torsion angles between benzophosphole and peripheral aryl group are given.

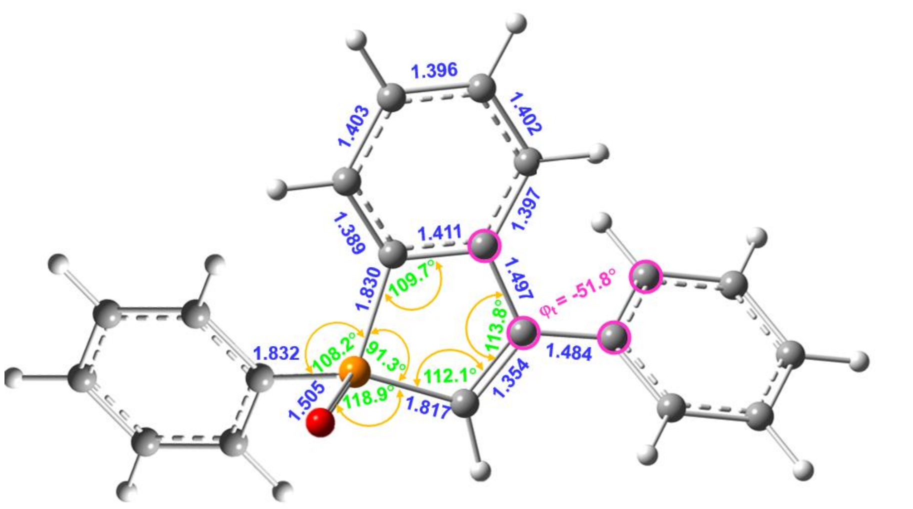

5a S_0

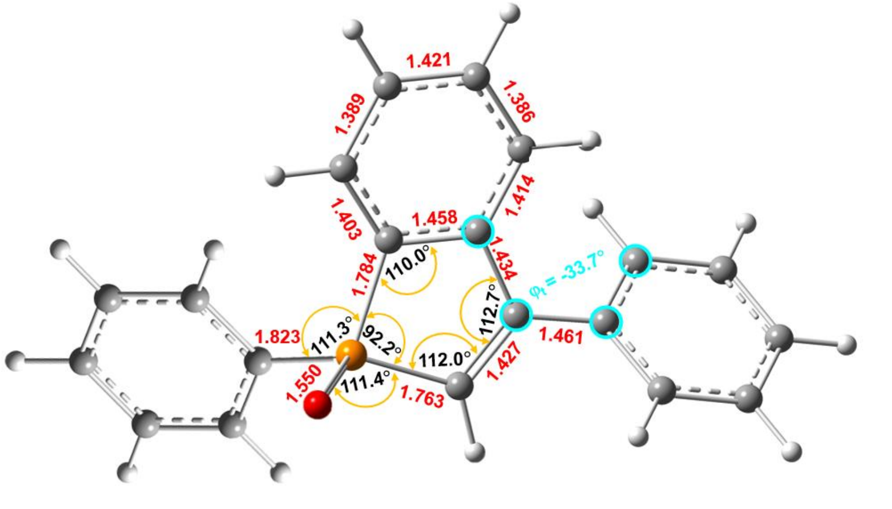

5a S_1

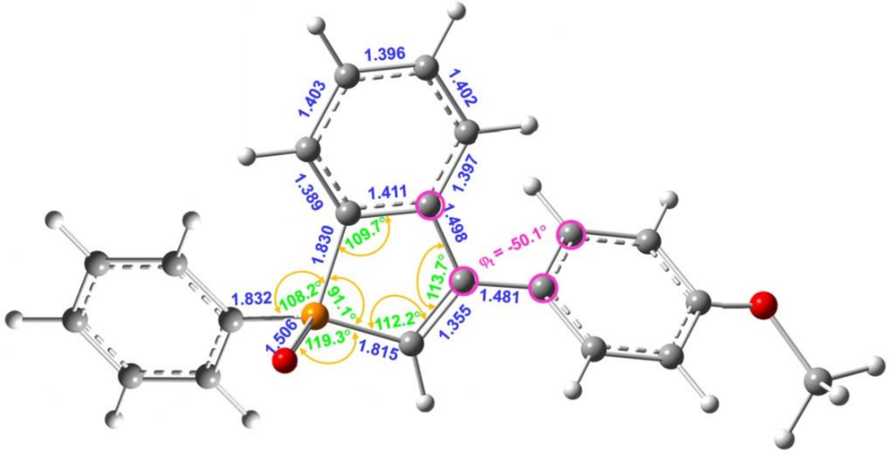

5e S_0

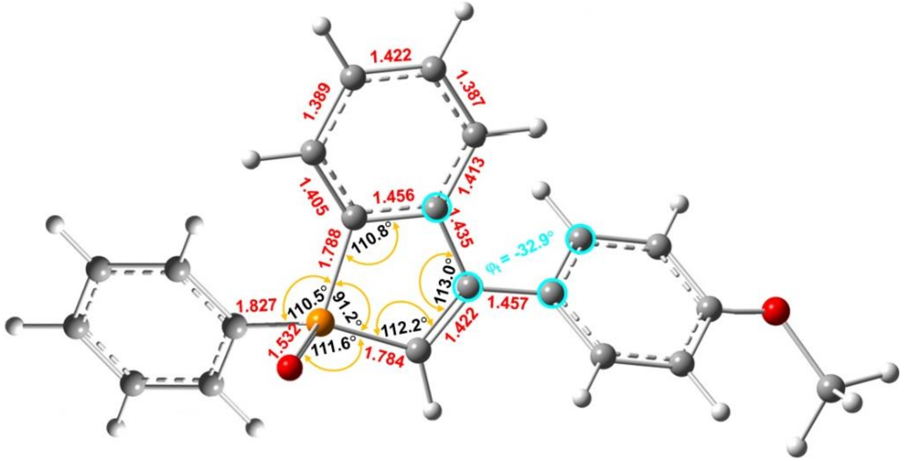

5e S_1

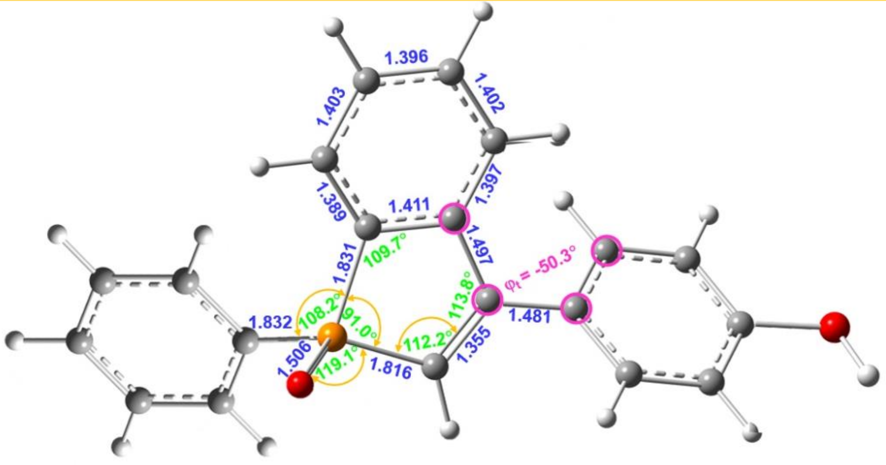

5h S_0

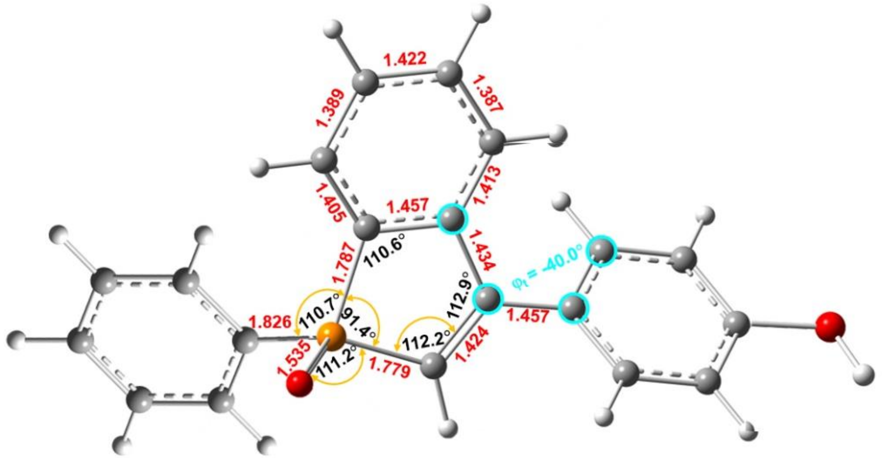

5h S_1

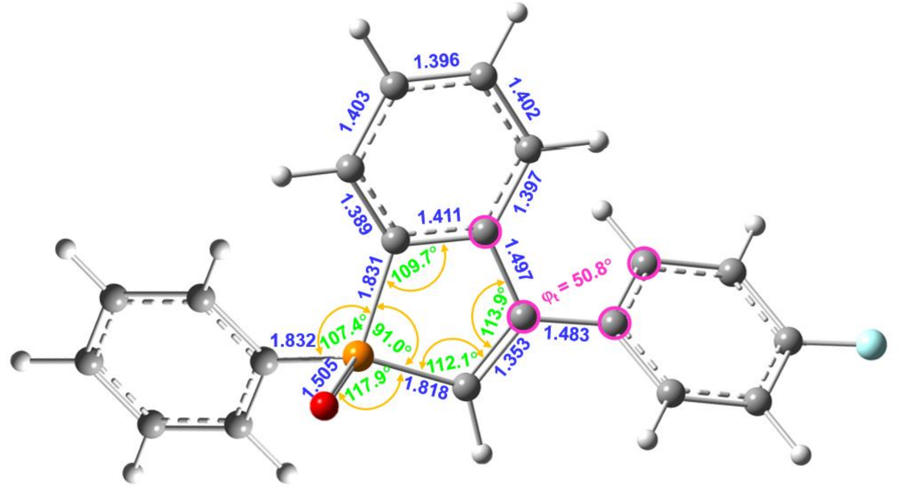

5j S_0

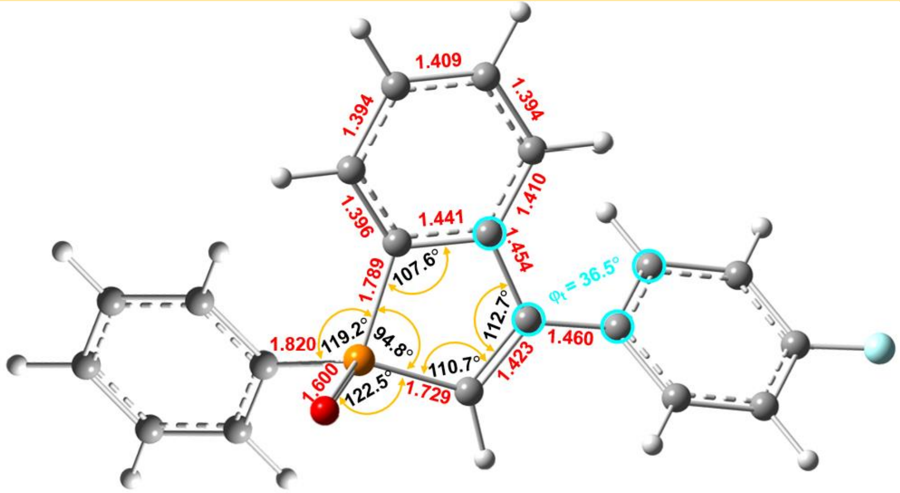

5j S_1

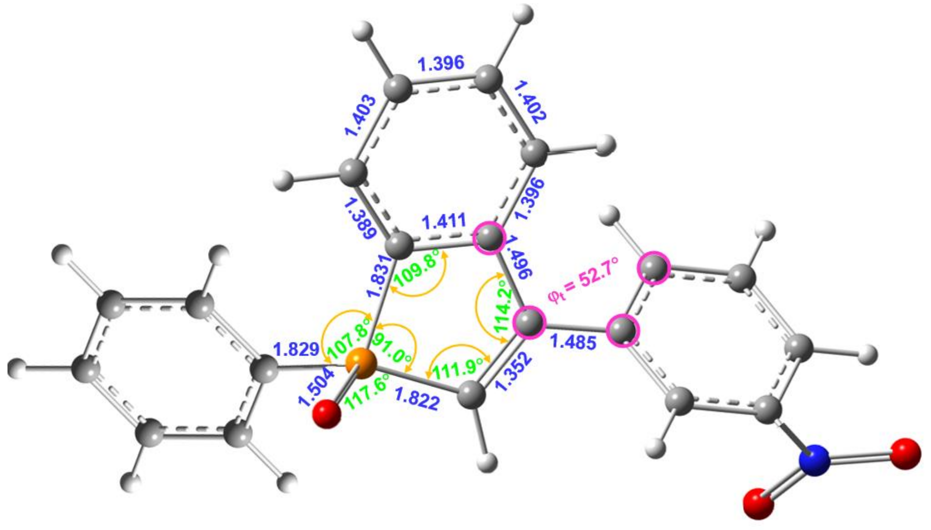

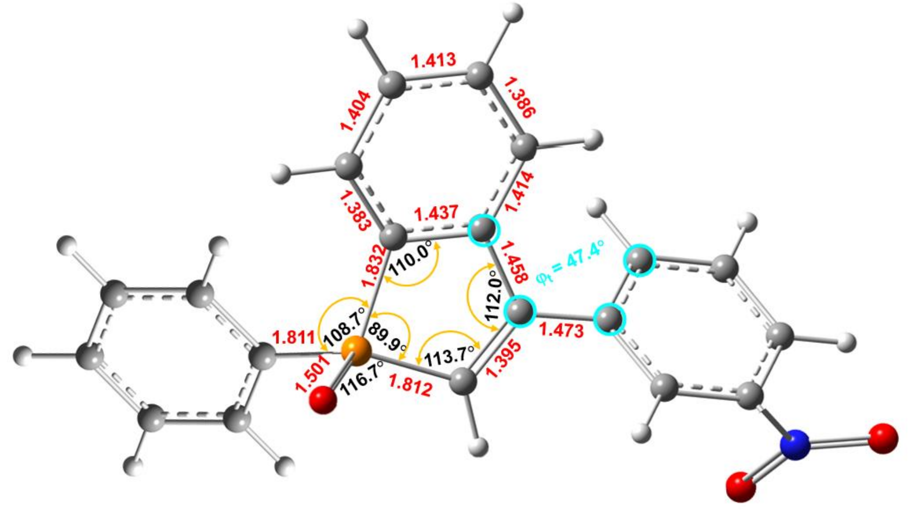

**5n S_0**	**5n S_1**

Table S7. Dihedral (tortion) angles (φ_t) for **5a,e,h,j,n**.

**No.**	**Comp.**	**Substituents**	**Calculated φ_t in S_0 [º]**	**Calculated φ_t in S_1 [º]**	**φ_t in S_0 from X-ray crystallography [º]**
**1**	**5a**	$R^1 = R^2 = H$	-51.8	-33.7	-
**2**	**5e**	$R^1 = H$, $R^2 = OMe$	-50.1	-32.9	-
**3**	**5h**	$R^1 =H$, $R^2 = OH$	-50.3	-40.0	-
**5**	**5j**	$R^1 = F$, $R^2 = H$	50.8	36.5	-
**6**	**5n**	$R^1 = H$, $R^2 = NO_2$	52.7	47.4	59.5(6)

Benzophosphole oxides **5a,e,h** have aryl at the 3-position oriented in twisted fashion (in the same direction with a dihedral angle of around -50°) relative to the benzophosphole core in S_0, their orientation becomes more planar in the excited state. The planarity of the molecule enhances the conjugation. And for electron-withdrawing substituted **5j** and **5n**, the ring is twisted in the opposite direction (with a dihedral angle 50.8-52.7°). For **5j,** the change of dihedral angle in the excited state is less marked than for **5a**, while for **5n** the change is minimal. The dihedral angle for **5n** in S_0 is consistent with the value obtained from X-ray crystallography.

The further comparison of geometries (Table S6) of compounds **5a,e,h,j,n** in S_0 showed that the bonds in the conjugated system are not affected by the substitution pattern and are in good correlation with X-ray crystallography (as was showed for **5n**). From S_0 to S_1 for **5a,e,h,j** double bonds: O1-P1 and C2-C3 are elongated (by 0.045-0.095Å and ~0.07Å, respectively), and single bonds P1-C2 and C3-C4 are shortened by (~0.055 Å and ~0.02 Å, respectively). In case of **5n,** O1-P1, P1-C2 and C3-C4 bonds were slightly shortened (<=0.01 Å) and C2-C3 was elongated by 0.043Å.

In the ground state, the conjugated bond system in benzophosphole oxides **5a,e,h** is less enriched in charge than that of their analogues **5j** and **5n** which possess F and NO_2 substituents. In the excited state, the charge is shifted from the 3-substituent to the conjugated bond system for benzophospholes **5a,e,h** (small increase of μ) while a minimal change is observed for **5f** (decrease of μ) and **5n** (huge increase of μ - highly polarizable excited state). Especially, donating substituents at the 3-position (in **5a,e,h**) affects conjugation in S_1 state due to delocalization (change in electron density and the bond length). In turn, in **5j**, less charge is delocalized in the conjugated bond system, but the bond lengths are still affected. In contrast, for **5n** these changes are less marked. Therefore, we assume that the geometry of the structure has less impact on the optical properties of **5n** than it is for **5a,e,j**.

In line with experimental and DFT energy gap analysis, TD-DFT geometry analysis of S_0/S_1 led to two groups of compounds in which optical properties are originated from conjugation in benzophosphole ring (**5a,e,h,j**) and another (represented by **5n**) in which optical properties are determined by ICT character.

Table S8. Some calculated bonds length and Mulliken charges at selected atoms in **5a,e,h,j,n** (* data obtained from X-ray crystallography).

No.	Comp.	Substituents	Calculated bonds length in S_0				Mulliken charge in S_0				Σ S_0	
			O1-P1	P1-C2	C2-C3	C3-C4	O1	P1	C2	C3		
**1**	**5a**	$R^1 = R^2 = H$	1.505	1.817	1.354	1.484	-0.536	0.608	-0.588	0.437	-0.079	
**2**	**5e**	$R^1 = H, R^2 = OMe$	1.506	1.815	1.355	1.481	-0.547	0.597	-0.592	0.484	-0.058	
**3**	**5h**	$R^1 = H, R^2 = OH$	1.506	1.816	1.355	1.481	-0.540	0.589	-0.593	0.4	-0.144	
**5**	**5j**	$R^1 = F, R^2 = H$	1.505	1.818	1.353	1.483	-0.537	0.584	-0.539	-0.024	-0.516	
**6**	**5n**	$R^1 = H, R^2 = NO_2$	1.504 [1.4876]*	1.822 [1.7927]*	1.352 [1.346]*	1.485	-0.549	0.578	-0.497	-0.167	-0.635	
**No.**	**Comp.**	**Substituents**	**Calculated bonds length in S_1**				**Mulliken charge in S_1**				**Σ S_1**	**S_1-S_0**
			**O1-P1**	**P1-C2**	**C2-C3**	**C3-C4**	**O1**	**P1**	**C2**	**C3**		
**1**	**5a**	$R^1 = R^2 = H$	1.550	1.763	1.427	1.461	-0.327	0.556	-0.981	0.271	-0.481	-0.402
**2**	**5e**	$R^1 = H, R^2 = OMe$	1.532	1.784	1.422	1.457	-0.392	0.556	-0.929	0.4	-0.365	-0.307
**3**	**5h**	$R^1 = H, R^2 = OH$	1.535	1.779	1.424	1.457	-0.371	0.566	-1.009	0.367	-0.447	-0.303
**5**	**5j**	$R^1 = F, R^2 = H$	1.600	1.729	1.423	1.460	-0.223	0.598	-0.62	-0.31	-0.555	-0.039
**6**	**5n**	$R^1 = H, R^2 = NO_2$	1.501	1.812	1.395	1.473	-0.494	0.508	-0.524	-0.150	-0.66	-0.025

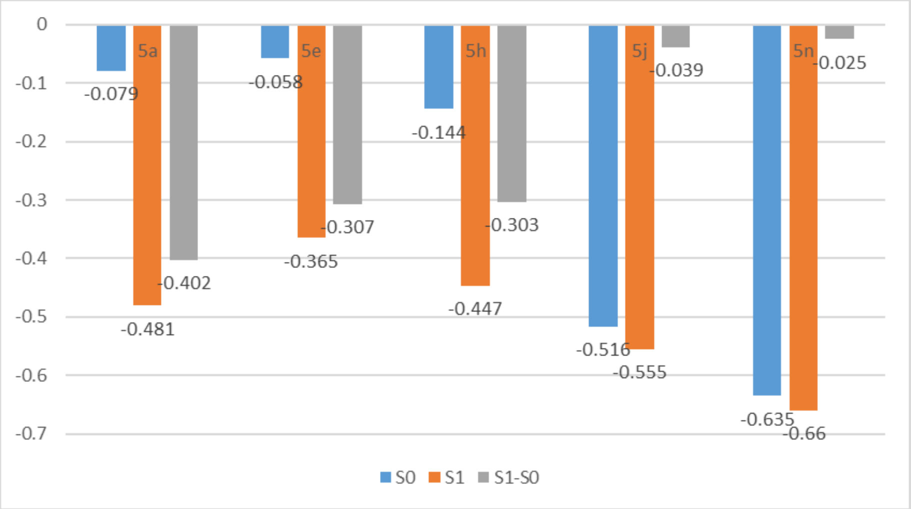

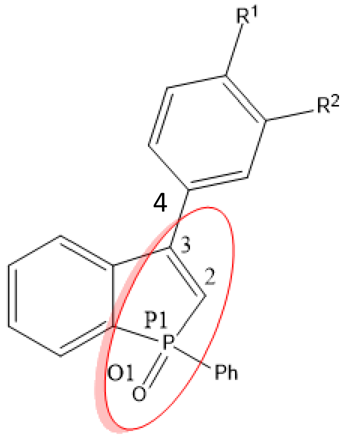

Fig. S14. The Changes of charge in the conjugated bond system from S_0 to S_1.

Table S9. The ground state (minima) geometries for the analyzed compounds (optimized at the DFT/B3LYP/6-31+g(d,p) level)

**5a**	**X**	**Y**	**Z**
c	0.621877342749	1.323177426277	0.210465609955
c	-0.716235406182	1.614289063991	0.551359020769
c	-1.276888603382	2.853613066252	0.271006762111
c	-0.495621180018	3.827788481070	-0.368681367032
c	0.830795522627	3.549875783362	-0.703677625265
c	1.399273065541	2.301489197992	-0.413546448270
c	1.056212210462	-0.043726216303	0.638979382444
c	0.102435064020	-0.739339585808	1.301554263347
h	-2.303297625037	3.071132230832	0.552564601998
h	-0.920433812599	4.801633036588	-0.592959259952
h	1.435774024536	4.311396262488	-1.187259067348
h	2.437785637752	2.109682261408	-0.661422546141
h	0.229850066651	-1.750936073407	1.669285664860
c	2.414561927799	-0.575310145752	0.368360144514
c	2.964436002856	-0.549699834754	-0.925750137707
c	3.164214204022	-1.154939982392	1.406477015106
c	4.223835399278	-1.098081382694	-1.174329030560
h	2.393066866091	-0.123304403762	-1.744896139386
c	4.427397904261	-1.694398689506	1.158173607291
c	4.961179776796	-1.668989942569	-0.133072754438
h	4.627629798320	-1.081830768162	-2.182584278660
c	-2.669854281471	-0.606218029703	0.345129125239
c	-2.388765337266	-0.930224789254	-0.991056386022
c	-3.930046867235	-0.902194677581	0.881687901336
c	-3.360749446962	-1.544200961996	-1.781723046949
h	-1.413661537402	-0.704426349881	-1.414380498000
c	-4.901550078656	-1.518048644584	0.086841197773
h	-4.133721749101	-0.645672832064	1.916994300438
c	-4.618357185362	-1.838792978841	-1.242772748495
h	-3.139069411668	-1.792974948015	-2.815587652669
h	-5.876850381072	-1.746091107391	0.507192904766
p	-1.448491031293	0.196733291876	1.448393052715
o	-2.018054870992	0.452441703069	2.818252072825
h	-5.373529237830	-2.317643636499	-1.859659781565
h	4.995204120414	-2.131277585902	1.974526425676
h	2.756154713899	-1.165174849236	2.412766236422
h	5.943494395458	-2.089753389144	-0.326650521129
**5b**	**X**	**Y**	**Z**
c	-0.030136181045	1.478584759950	0.142135913631
c	-1.379731794048	1.680842106498	0.502440755961
c	-2.065060448876	2.824353391093	0.113007468573
c	-1.400569272463	3.790111156303	-0.657909424353
c	-0.074021147712	3.585061039459	-1.041477483674
c	0.617183090811	2.429715592435	-0.649964441724
c	0.534088939419	0.189740853105	0.655179514191
c	-0.361380091072	-0.565977317504	1.333563315864
h	-3.101828021446	2.971522423321	0.402281829282
h	-1.921133193833	4.693415819473	-0.961598257338

h	0.431667344625	4.327481482123	-1.652172233622
h	1.642656323647	2.278033927649	-0.969793953075
h	-0.127162764040	-1.511451701864	1.808885740298
c	1.944618740376	-0.210855270282	0.437314741340
c	3.009031772725	0.658972604221	0.731624701857
c	2.255257251013	-1.500551396534	-0.024333989042
c	4.331993018401	0.246189746799	0.574425892787
h	2.802075657644	1.651406050271	1.120504940063
c	3.580454496119	-1.903359650720	-0.188095632573
h	1.449004963174	-2.187706639862	-0.264382714875
c	4.644293256387	-1.038455259571	0.105426528600
h	5.135880564150	0.932792052403	0.828609768035
h	3.790392980929	-2.906951592434	-0.550015562778
c	-3.154384439716	-0.672150029280	0.390836646848
c	-2.881338927758	-0.971896039334	-0.952734651482
c	-4.366072113406	-1.083589371412	0.962022502463
c	-3.812071466666	-1.678378484320	-1.715437735728
h	-1.945565830584	-0.653574207395	-1.404639282973
c	-5.296446396278	-1.791429398613	0.194932753301
h	-4.565245307576	-0.841978372330	2.001794772990
c	-5.020787265028	-2.089018326528	-1.141652814009
h	-3.596918426596	-1.906998542013	-2.755351403036
h	-6.234413781324	-2.108125382259	0.641986156649
p	-1.982664522114	0.242833461935	1.461789320665
o	-2.552518184046	0.479601050989	2.834737125121
h	-5.744324191852	-2.638542205315	-1.737207590523
c	6.078291339156	-1.466985444536	-0.099481905429
h	6.193715068081	-2.548199751189	0.023525152882
h	6.424354713056	-1.213684774060	-1.109679366407
h	6.748814247764	-0.970798360668	0.608902901250
**5c**	**X**	**Y**	**Z**
c	0.093111714453	1.659544407127	0.097876806364
c	-1.269670210082	1.824971311962	0.425693683904
c	-1.996792082763	2.910129185929	-0.046245136731
c	-1.361389316517	3.852788636178	-0.868595556546
c	-0.020765438478	3.682473202854	-1.218841124873
c	0.712836084111	2.586118140160	-0.743763552681
c	0.703310124944	0.432516623359	0.701130595725
c	-0.168398112564	-0.315747745698	1.416938917807
h	-3.043411802846	3.030318593891	0.219019589295
h	-1.915025077283	4.711152686393	-1.237207483394
h	0.463046563359	4.406368432277	-1.868263513237
h	1.749903442628	2.460370205497	-1.036350387585
h	0.098946429909	-1.217901725761	1.954823855335
c	2.134664720439	0.082466621295	0.528151044464
c	3.148980987235	1.016663417279	0.792472164356
c	2.498066029603	-1.217214342935	0.133418186814
c	4.489392197095	0.645145910316	0.669918563725
h	2.891772148933	2.017719911672	1.124062270940
c	3.837173344543	-1.599583195993	-0.002358062008
h	1.713189403290	-1.937669391659	-0.081774243812

c	4.831502375879	-0.648062166485	0.272916963197
h	5.270062140927	1.367423442993	0.891202509041
c	-2.936305026371	-0.607886045675	0.444522976927
c	-2.629072544805	-0.976925549956	-0.874278664467
c	-4.135405847522	-1.041412598015	1.025721204031
c	-3.513335128491	-1.773825587200	-1.602255958393
h	-1.703058559075	-0.641717622026	-1.333972237917
c	-5.019216406973	-1.839975427715	0.293450049758
h	-4.361747036183	-0.745452017224	2.045706901600
c	-4.709521832671	-2.206385789242	-1.018350381237
h	-3.272042332388	-2.055737588760	-2.623176206262
h	-5.947669824768	-2.173400670171	0.748126745687
p	-1.825480010771	0.427648626606	1.469657978233
o	-2.429066819618	0.727354399147	2.815537693649
h	-5.396996515825	-2.826307794105	-1.586868046913
h	5.879106283858	-0.923332069109	0.177098056431
c	4.208340317051	-2.998303569856	-0.439645712764
h	3.322912872454	-3.630279669853	-0.551301241756
h	4.734325550648	-2.986490548268	-1.401554422905
h	4.873727194636	-3.477562639230	0.287355176193
**5d**	**X**	**Y**	**Z**
c	0.519468099889	1.465217313774	0.075063399318
c	-0.807528426112	1.828540611149	0.387725178825
c	-1.367243482758	2.996662041166	-0.113282243412
c	-0.594903023117	3.820904048542	-0.946605654645
c	0.714403773156	3.459618428543	-1.270776562172
c	1.280132970149	2.280634040959	-0.764747305449
c	0.954305476581	0.184592381154	0.713044323892
c	-0.003325233727	-0.407426901097	1.459577576874
h	-2.386703535248	3.273981030827	0.139717613934
h	-1.017418204002	4.740943364136	-1.339279234733
h	1.304691547692	4.099154326278	-1.920679649780
h	2.298532405791	2.007021253215	-1.022351846633
h	0.133340093499	-1.325819218147	2.018829703451
c	2.343706691014	-0.331000503134	0.547687287484
c	3.396250208913	0.385180081460	1.141343181903
c	2.622161806706	-1.510701478626	-0.178559514355
c	4.715795986821	-0.056167876357	1.041203675731
h	3.169845963710	1.289968754623	1.698639397018
c	3.956620993029	-1.932165322209	-0.274900348153
c	4.996635302187	-1.222507283145	0.327588956329
h	5.513949847063	0.506273293160	1.516456380331
c	-2.802392712513	-0.347656040914	0.522466211949
c	-2.605912233581	-0.734835116220	-0.812067450951
c	-4.005149322147	-0.666274772542	1.167406877835
c	-3.601246775934	-1.438141266610	-1.491533484173
h	-1.680507471066	-0.483628176707	-1.323229490506
c	-5.000268246883	-1.371716645675	0.484118089128
h	-4.148829665441	-0.351822900111	2.196788627311
c	-4.799334768533	-1.758380355956	-0.842969766807
h	-3.445327303558	-1.733069254108	-2.525303099715

h	-5.930926104046	-1.615721234613	0.988308857120
p	-1.542295228184	0.564862705591	1.490068063944
o	-2.075233374362	0.998257876092	2.829388637569
h	-5.573442204376	-2.305415680629	-1.373641442161
h	6.019022468717	-1.577815201729	0.236432553350
h	4.181032071093	-2.835643131855	-0.836291711046
c	1.532169432397	-2.314255627099	-0.853223310078
h	0.904218260750	-2.840522823807	-0.126125869682
h	0.866519832830	-1.677503056962	-1.444800760240
h	1.965184083593	-3.063621682410	-1.521485848598
**5e**	**X**	**Y**	**Z**
c	-0.362991106853	1.578343781018	0.126441690989
c	-1.733852480801	1.630485152371	0.457672813583
c	-2.502132514342	2.751184547830	0.169988644865
c	-1.901046655142	3.845564772038	-0.469873474076
c	-0.544049928435	3.805115990234	-0.794939861996
c	0.233778046415	2.677723442981	-0.495229054194
c	0.303717445466	0.311032372031	0.565748650554
c	-0.518584217004	-0.535305048534	1.231781800344
h	-3.552144746107	2.785799392995	0.446373921341
h	-2.489484742183	4.728486265623	-0.700966652157
h	-0.079306122011	4.660105283536	-1.277507479498
h	1.292120801837	2.673476297342	-0.732781498365
h	-0.219309024110	-1.508700971846	1.602818943983
c	1.733848221173	0.027132765505	0.308520263374
c	2.301362101844	0.174521072496	-0.973693912894
c	2.558755817674	-0.448244153791	1.337325638757
c	3.630447701885	-0.148942946479	-1.213743754507
h	1.685777358302	0.518213134437	-1.799444357373
c	3.900646923571	-0.767656375467	1.113847072618
c	4.442432310380	-0.619928671493	-0.169378020780
h	4.061893027538	-0.050113539864	-2.204546565836
c	-3.264479526182	-0.903985943825	0.269077993569
c	-2.899560405039	-1.232309352556	-1.045541115140
c	-4.482683486833	-1.363807530219	0.786960515820
c	-3.747477698710	-2.011072323117	-1.833989364890
h	-1.954662151722	-0.883030831563	-1.453124269009
c	-5.329941963458	-2.144253933515	-0.005458984358
h	-4.750210430740	-1.105975937713	1.807315492836
c	-4.964021724576	-2.467616373235	-1.314142110281
h	-3.460353522285	-2.262999339121	-2.850875308429
h	-6.272899135248	-2.498924348716	0.400747936338
p	-2.208152545302	0.113866634303	1.366390689934
o	-2.825759505377	0.284368247214	2.729147059052
h	-5.622428279216	-3.074716267582	-1.929045720826
h	4.504568931378	-1.120845922552	1.941003513858
h	2.150130574659	-0.555064014443	2.337813745637
o	5.734539210105	-0.903038635502	-0.502580064971
c	6.607739243074	-1.394491291730	0.508922740139
h	6.247639218946	-2.346212107060	0.918626310858
h	7.569338486179	-1.551497564852	0.019326712841

h	6.726796491257	-0.666685727180	1.321009418282
**5f**	**X**	**Y**	**Z**
c	0.306501165557	1.564624343046	-0.263254981433
c	1.631111133898	1.547226966765	-0.749590122701
c	2.426911177423	2.684813271873	-0.710399489886
c	1.900689943725	3.868077880104	-0.170420652490
c	0.589217169295	3.895040595064	0.307375203803
c	-0.216739206762	2.748791557430	0.260960857071
c	-0.404417404911	0.259479234097	-0.440410768832
c	0.334503190877	-0.687823816334	-1.063579662728
h	3.439768901313	2.663357500081	-1.102508980801
h	2.510871656356	4.765626326144	-0.134726023011
h	0.181938538023	4.816700168325	0.712987851741
h	-1.240203608000	2.791460990563	0.617803188025
h	-0.008841012942	-1.696799601852	-1.260442820246
c	-1.796057619054	0.035540485363	0.024714958667
c	-2.179275749827	0.339953002176	1.346582193577
c	-2.735004661721	-0.527013232483	-0.845143767646
c	-3.477049499870	0.068105009108	1.769802807177
h	-1.457134315211	0.756890395826	2.040665236833
c	-4.043953191894	-0.788823948839	-0.414382994806
c	-4.421584915493	-0.491451110910	0.900984941463
h	-3.767033264543	0.289687058793	2.792939737574
c	3.166846442644	-0.957268327710	-0.349907126149
c	2.954804511696	-1.058269432314	1.033639541748
c	4.307886178138	-1.535378740040	-0.921995547192
c	3.877540726899	-1.730311284957	1.836014396892
h	2.070964826596	-0.614932992638	1.484433470535
c	5.230640160007	-2.207988533823	-0.115139186359
h	4.457966358184	-1.451366312879	-1.994187637019
c	5.016791996806	-2.305685879037	1.261723353001
h	3.709206465275	-1.806313892936	2.906443098605
h	6.113642299121	-2.654703776563	-0.563093929223
p	2.003112929345	-0.093886348108	-1.470385118991
o	2.468983572186	-0.140721608249	-2.901111299740
h	5.733740176448	-2.829166546173	1.887907756007
h	-2.470733100168	-0.755619721831	-1.872284715640
h	-5.426532625979	-0.685699259064	1.255495830718
o	-4.872965183600	-1.332027172382	-1.353667333555
c	-6.217372554194	-1.624063648904	-0.990188876045
h	-6.682020523811	-2.040428353733	-1.884582263481
h	-6.260831297742	-2.362771647086	-0.180089617848
h	-6.755889784092	-0.716859595911	-0.688981507619
**5g**	**X**	**Y**	**Z**
c	-0.134230615581	1.460689739579	-0.266543465366
c	1.167985926196	1.511260582192	-0.807485566788
c	1.843603814139	2.716470489245	-0.947582347461
c	1.216653332174	3.902938901954	-0.537604885576
c	-0.073531473946	3.862348431329	-0.006101884968
c	-0.757774003120	2.645982030533	0.129708184889
c	-0.712153872084	0.078580186306	-0.241908706977

c	0.100296957616	-0.862645899548	-0.773570473371
h	2.840053320070	2.743042387240	-1.379511267201
h	1.731911388496	4.852960702619	-0.643285759820
h	-0.559925243918	4.784188451851	0.299814694493
h	-1.766794512950	2.634095793192	0.528078101720
h	-0.151812577994	-1.912689313331	-0.853075653775
c	-2.051841486194	-0.194978805634	0.338866598641
c	-2.347396107748	0.215026547062	1.646685096400
c	-3.052636655365	-0.888231912835	-0.386751929702
c	-3.584740032829	-0.045401061877	2.239866693387
h	-1.580826521000	0.734649077728	2.214016731154
c	-4.300773974033	-1.137772295588	0.199833560507
c	-4.560489827187	-0.719790341870	1.507855406519
h	-3.779248203445	0.274801119218	3.258455038667
c	2.966714603794	-0.750117001509	-0.153752452002
c	2.806698340578	-0.691092353742	1.239072881798
c	4.148300098787	-1.273693034112	-0.695196715247
c	3.820555571197	-1.150127819151	2.080721317679
h	1.892102620775	-0.288935887305	1.666518649239
c	5.162373091014	-1.733141116975	0.150722248849
h	4.257609264488	-1.315102206451	-1.774793076231
c	4.999862881314	-1.671685264605	1.536673872792
h	3.692013092157	-1.102482184730	3.158315176006
h	6.076482997253	-2.138298421257	-0.273843439083
p	1.684275737707	-0.165899814881	-1.325362208945
o	2.113487849843	-0.357070031202	-2.755897781882
h	5.787906035087	-2.029289451451	2.193426752084
h	-5.531410080047	-0.927692428587	1.948114512676
h	-5.072087205450	-1.659318618685	-0.353499386910
o	-2.728995274331	-1.269740017678	-1.654353844985
c	-3.713230263763	-1.909542808920	-2.458955969956
h	-4.028004662399	-2.865483988344	-2.022343197193
h	-4.589323508490	-1.265746344937	-2.605042651333
h	-3.231660820809	-2.091066014851	-3.420282852721
**5h**	**X**	**Y**	**Z**
c	0.296796693053	1.437216277113	0.168795030989
c	-1.071529548651	1.599934104031	0.473983462545
c	-1.747234461119	2.769610566246	0.151307177707
c	-1.053738183364	3.802346390284	-0.497464610255
c	0.301510182942	3.652587408743	-0.796718616595
c	0.985976497908	2.475514130927	-0.462181073514
c	0.856770562567	0.131205646306	0.641851284166
c	-0.039061211511	-0.638102561696	1.306193600408
h	-2.796103906920	2.889251934716	0.407605803733
h	-1.569135577905	4.722637309441	-0.755536806271
h	0.838090675945	4.460145477774	-1.286346484308
h	2.044756384209	2.386439052142	-0.680448276794
h	0.178217879326	-1.624459690095	1.699463665454
c	2.265717564687	-0.265820569910	0.415425755174
c	2.861462285998	-0.189625494489	-0.857988095794
c	3.036068575987	-0.779066408914	1.471585160010

c	4.168740396292	-0.616297564805	-1.072956293845
h	2.285265226157	0.181450329893	-1.700055020736
c	4.350281659305	-1.199988311521	1.270598845677
c	4.919633757321	-1.120084054024	-0.004814316090
h	4.619308755093	-0.569926021542	-2.058900346166
c	-2.790966089739	-0.810713465787	0.301684611317
c	-2.442773221828	-1.165843568191	-1.010494451537
c	-4.037961082595	-1.189969042221	0.816237279288
c	-3.335221700478	-1.892611004594	-1.799404850876
h	-1.476705974903	-0.877543747266	-1.416160337278
c	-4.929932617521	-1.918282389929	0.023222058678
h	-4.293717859990	-0.909410065494	1.833605192319
c	-4.580143815476	-2.269365651732	-1.282693646316
h	-3.061236243499	-2.165395283621	-2.814514186927
h	-5.895338388847	-2.210242412508	0.426567925084
p	-1.676217771758	0.141301652545	1.399978019149
o	-2.298295805082	0.380922280409	2.750084900744
h	-5.273501082850	-2.835605796857	-1.898151778010
h	4.931464643734	-1.583371604860	2.106410710843
h	2.605195510633	-0.833004031945	2.466761140846
o	6.202083197554	-1.516360221855	-0.270460541326
h	6.621474095324	-1.849473596711	0.533928108501
**5i**	**X**	**Y**	**Z**
c	0.217450070478	1.572102969375	0.078801360634
c	-1.143321477463	1.746722145192	0.410572531646
c	-1.863411033685	2.839134537479	-0.055408596455
c	-1.223002071250	3.779567558064	-0.876330999150
c	0.115279736244	3.599862810488	-1.231044067522
c	0.841939036861	2.496432182792	-0.761815764956
c	0.819786569139	0.338392993434	0.676324067393
c	-0.055924074089	-0.405137897215	1.392166431283
h	-2.908353548807	2.966541862857	0.213316917813
h	-1.770963174377	4.643604402284	-1.240241217574
h	0.602834418464	4.322126938376	-1.879617840276
h	1.877280943865	2.363066716481	-1.056636287843
h	0.206303147509	-1.310557830438	1.926879945183
c	2.248095750573	-0.022825503190	0.497055216253
c	3.269972035710	0.904641503909	0.766681820159
c	2.586984034163	-1.321730535498	0.093380437394
c	4.603832484433	0.513017739002	0.638005410760
h	3.024349365750	1.903978093437	1.109144692496
c	3.928356736396	-1.714589578857	-0.046619186169
c	4.938923011183	-0.777408464274	0.231221646830
h	5.395770460853	1.221931335657	0.862318379019
c	-2.823690982806	-0.675811780968	0.419633514456
c	-2.518321983337	-1.040032269093	-0.900954787965
c	-4.024531475161	-1.106879985561	0.998874305963
c	-3.406217948895	-1.829606767404	-1.632610045065
h	-1.591214532351	-0.706214496657	-1.359521124875
c	-4.912040065746	-1.898119020662	0.263039193258
h	-4.249556301479	-0.814368770606	2.020151178427

c	-4.604215334305	-2.259742232727	-1.050535808943
h	-3.166512066233	-2.107220349636	-2.655078170346
h	-5.841998455282	-2.229319891656	0.716246784295
p	-1.707236060755	0.349588597578	1.449851168145
o	-2.310818796553	0.646387119727	2.796444522992
h	-5.294521029608	-2.873769393601	-1.621896905033
h	1.795076019660	-2.031221176572	-0.132467397528
h	5.982454727797	-1.062467836503	0.123603855906
n	4.247744683937	-2.991932759871	-0.511138644538
h	5.174391076696	-3.326858957599	-0.286439393754
h	3.539026102475	-3.701284007552	-0.385357142315

**5j**	**X**	**Y**	**Z**
c	0.515069502909	1.340789343376	0.130587320646
c	-0.830607662031	1.556515736026	0.497563505156
c	-1.503965378916	2.709940480147	0.116360489374
c	-0.830792894842	3.672336238692	-0.651314725313
c	0.492069234544	3.454334964643	-1.040520391182
c	1.171045390525	2.288883237654	-0.657763003773
c	1.065529723318	0.043207231794	0.635067899321
c	0.165105637383	-0.707015221590	1.311846680702
h	-2.537814080801	2.867577657453	0.409962233291
h	-1.341768532476	4.583218208665	-0.948067820088
h	1.004298593085	4.194257802969	-1.648840738538
h	2.193385240642	2.127891839283	-0.983404718567
h	0.390232443233	-1.657101840093	1.782275257575
c	2.472521850800	-0.370166648583	0.412332994786
c	3.542897952238	0.487722693202	0.723951968573
c	2.764335643157	-1.655730869880	-0.075630756443
c	4.866214698655	0.076103800215	0.561860050302
h	3.342237235660	1.475676513149	1.126160660458
c	4.081771349412	-2.080496714802	-0.251588772241
c	5.107920489070	-1.202047433151	0.072679352086
h	5.698159253698	0.724787923522	0.813993661245
c	-2.634230342317	-0.774936634953	0.383950408006
c	-2.381251482009	-1.051357743106	-0.968640821166
c	-3.836764038225	-1.197088351072	0.966659579969
c	-3.322801023807	-1.745967364866	-1.728821360973
h	-1.453542546158	-0.722901684441	-1.429834415625
c	-4.777890856562	-1.892958816943	0.201814182814
h	-4.021158605621	-0.972700160345	2.012927980951
c	-4.522160084886	-2.167686619217	-1.143526967291
h	-3.123715128271	-1.956228474091	-2.775723071677
h	-5.708788938398	-2.217918212332	0.657558834918
p	-1.447153526280	0.121279111301	1.452458384188
o	-2.002193588464	0.355632065240	2.831412256363
h	-5.254136481879	-2.707909854671	-1.737230461506
h	4.315118620086	-3.068049363608	-0.634492284472
f	6.393810704079	-1.606613805774	-0.094665970961
h	1.949011629455	-2.325279033812	-0.331357420903

**5k**	**X**	**Y**	**Z**
c	0.472282627099	1.369008085244	0.059694663231

c	-0.885926965309	1.557195576815	0.394027629360
c	-1.591148505318	2.664105884967	-0.060292539984
c	-0.938277292217	3.604834954359	-0.871085487864
c	0.397687356639	3.412297122390	-1.227341385359
c	1.109628963630	2.294460321227	-0.769786529008
c	1.056526317499	0.121106404994	0.643692060093
c	0.173518923518	-0.620742662697	1.350708508096
h	-2.634287293040	2.802505520160	0.209581216399
h	-1.475037149742	4.479395618013	-1.226160274781
h	0.894620553710	4.135121977492	-1.867858985166
h	2.143227790272	2.152359887779	-1.067379054827
h	0.423485827330	-1.536668869376	1.873683804085
c	2.481864610705	-0.253769966218	0.466086469123
c	3.515772141874	0.654684475762	0.755919370727
c	2.814391913579	-1.551052228457	0.042144970207
c	4.852175478123	0.270194428198	0.632309932174
h	3.272565357327	1.651563492508	1.108221564491
c	4.153155323266	-1.898169547762	-0.078801472562
c	5.188906326528	-1.017550396641	0.206165927143
h	5.640497613008	0.976646902497	0.873699973819
c	-2.606971934066	-0.840415992880	0.394883493564
c	-2.321350666043	-1.192618965098	-0.933371785905
c	-3.805596533379	-1.264177113610	0.984387559217
c	-3.226801619533	-1.964026200427	-1.662684650120
h	-1.396202830139	-0.864445480645	-1.399847216303
c	-4.710684553426	-2.036680947340	0.250330828164
h	-4.015164938196	-0.981362880661	2.011628954515
c	-4.422471021159	-2.386844144101	-1.070774812397
h	-3.002184847472	-2.233740415074	-2.690604077765
h	-5.638703465336	-2.362793485287	0.711007752396
p	-1.468526103200	0.159284994519	1.422841847778
o	-2.050276366835	0.455893190911	2.778441316976
h	-5.126322217834	-2.986959772057	-1.640422670346
h	2.046171496933	-2.276707297131	-0.201554567920
h	6.219223860265	-1.337530614064	0.096596144685
f	4.460231820943	-3.154401858297	-0.498088475929
**5l**	**X**	**Y**	**Z**
c	0.593009118180	1.300615529519	0.045680765644
c	-0.739219274759	1.617990472186	0.384341223699
c	-1.338321019216	2.782708078498	-0.076990714519
c	-0.602507846779	3.651029273760	-0.897985454724
c	0.708903949517	3.332227332705	-1.254818120555
c	1.314274997738	2.156309321527	-0.789241216076
c	1.061056139955	0.010766942236	0.641278911927
c	0.123514073775	-0.635603734092	1.368172272692
h	-2.362312525376	3.021882499617	0.195447571685
h	-1.056376356038	4.568904427093	-1.259567889684
h	1.269982850231	4.001041426868	-1.901052869470
h	2.330853025169	1.916469218032	-1.083777312205
h	0.284114295856	-1.576631412107	1.880435232863
c	2.450679068908	-0.476431377937	0.451419372680

c	3.557735428080	0.308454654508	0.823615737522
c	2.728919545730	-1.741422531163	-0.078999259557
c	4.863721044768	-0.159244167788	0.677802195150
h	3.379445057374	1.289672954303	1.252706836394
c	4.019275014899	-2.229259234420	-0.245114660887
c	5.095744403048	-1.429059001764	0.140643348181
h	5.696905157166	0.464277503819	0.986327999862
c	-2.669968530320	-0.618012775980	0.439147411614
c	-2.376496938537	-1.145285566971	-0.827938419323
c	-3.950845184508	-0.783529096321	0.983043894632
c	-3.359359445650	-1.828641432607	-1.544636535858
h	-1.382995649873	-1.029190399217	-1.252540099898
c	-4.932998730252	-1.468392008152	0.261313122725
h	-4.161122760573	-0.376813718512	1.967728458310
c	-4.638740147928	-1.989640935692	-1.000802724132
h	-3.127726459671	-2.237369028490	-2.523970593631
h	-5.924516454256	-1.595055151138	0.686393498049
p	-1.439107566405	0.294851420268	1.439450741325
o	-1.981932958031	0.678759787296	2.789601835279
h	-5.401999857280	-2.523071669349	-1.560399357319
h	6.109558282105	-1.798539671956	0.021942892711
h	4.161143609604	-3.217746269924	-0.668697717866
f	1.697712643342	-2.537021658649	-0.469960377229

**5m**	**X**	**Y**	**Z**
c	0.577528262328	1.320903137637	0.047225162218
c	-0.754847847976	1.636132325233	0.389831138013
c	-1.360114957661	2.798308954299	-0.070614008952
c	-0.631314247785	3.667547998300	-0.896354209936
c	0.677804287014	3.349474230523	-1.262004713115
c	1.288427559210	2.175495351208	-0.798502301367
c	1.051188924594	0.030918800489	0.640892624773
c	0.108179374197	-0.620027059180	1.360871909885
h	-2.383944272253	3.033761907868	0.205898682434
h	-1.088758140745	4.584362481410	-1.255830298287
h	1.232835125839	4.016828982816	-1.914881044758
h	2.300830870245	1.935920013353	-1.106361163805
h	0.277612188436	-1.548025845565	1.894699998649
c	2.432923890752	-0.475345350801	0.457153999117
c	3.549922644706	0.338881758450	0.713742042583
c	2.655027207213	-1.804879330563	0.061423509735
c	4.847335849231	-0.157336272075	0.587167800432
h	3.407145421014	1.362303159198	1.046038545574
c	3.947093925472	-2.311846118715	-0.077665695511
c	5.035789163494	-1.480993827466	0.187251319981
h	5.702734274601	0.473777719766	0.802003144934
c	-2.684045511455	-0.595115763072	0.416231858661
c	-2.438464648255	-0.975464813235	-0.912344649984
c	-3.911346670316	-0.910903326292	1.014210297938
c	-3.412000535231	-1.667777985997	-1.633261246500
h	-1.491442486005	-0.729886051115	-1.385665640035
c	-4.884640414447	-1.604640680315	0.288668470800

h	-4.089710040538	-0.606439375431	2.041163249685
c	-4.636193020330	-1.983211228870	-1.032636658961
h	-3.218446097841	-1.958773815906	-2.661726237297
h	-5.834767871414	-1.846851415187	0.756008527047
p	-1.455728141558	0.305382566674	1.433426640966
o	-2.003859986988	0.662832967804	2.788344593067
h	-5.393103986609	-2.521726458975	-1.595796379145
cl	6.667442283720	-2.109347591584	0.014746271094
h	4.107244156299	-3.337720134110	-0.390834624179
h	1.805663469035	-2.446519910565	-0.152520915746
**5n**	**X**	**Y**	**Z**
c	-0.084986774366	1.582754841289	-0.025704356510
c	-1.466918952296	1.517780623335	0.253364090359
c	-2.355212426410	2.428250774012	-0.304098205507
c	-1.865212805078	3.422626651209	-1.164294722974
c	-0.503334566387	3.477166088505	-1.465519563695
c	0.393834540869	2.557822276911	-0.903500172466
c	0.705963443304	0.520947363412	0.670307068965
c	-0.041063999162	-0.331620860113	1.407014885933
h	-3.415951673347	2.374252564631	-0.076252328003
h	-2.547569836787	4.145909182258	-1.600337447367
h	-0.130945616368	4.238695985527	-2.144389120762
h	1.446721496459	2.605550364989	-1.161934583648
h	0.361106341645	-1.141831236278	2.004503959344
c	2.184379371445	0.428903339061	0.566306535240
c	3.005133439918	1.539346222750	0.837553855317
c	2.789669780889	-0.790541839474	0.233354175912
c	4.396548628582	1.433669072327	0.785149269707
h	2.549528641796	2.484008919340	1.117188628045
c	4.179721440407	-0.869552526601	0.180010384094
c	5.002342520746	0.222701430900	0.450247995445
h	5.012680894489	2.298046358831	1.011215869062
c	-2.682771995144	-1.171424371736	0.406265502907
c	-2.287872403564	-1.548937675951	-0.886678817787
c	-3.784731376873	-1.794316669147	1.007704919429
c	-2.988935023394	-2.543529624121	-1.569123241663
h	-1.437439386219	-1.067780128293	-1.362520380500
c	-4.484975225572	-2.789957716481	0.320127827486
h	-4.081777193622	-1.489683355873	2.006700672484
c	-4.088047615118	-3.164985348369	-0.965475696056
h	-2.680127414793	-2.833199576240	-2.569288258904
h	-5.338600945538	-3.270329681961	0.789411351006
p	-1.808224340023	0.110660550844	1.374322123604
o	-2.484111098436	0.389007098489	2.688904580346
h	-4.632235689615	-3.939413104895	-1.498396022799
h	2.194646827615	-1.666824298380	0.007824496516
h	6.078393465957	0.114231711054	0.397939266487
n	4.802132069333	-2.159366961471	-0.179660982333
o	6.031893471374	-2.207630026673	-0.225705504430
o	4.056349983273	-3.111406417623	-0.412538052276

Table S10. The equilibrium geometry coordinates (XYZ) for S_1 states for the analyzed compounds

	**5a-S_1**		
**Symbol**	**X**	**Y**	**Z**
C	0.9127675	1.2674480	0.1279386
C	-0.5052802	1.4772212	0.3915016
C	-1.0766892	2.7504510	0.2466928
C	-0.2932227	3.8114931	-0.1885454
C	1.0948935	3.6239131	-0.4303569
C	1.6878260	2.3811382	-0.2706543
C	1.3331866	-0.0822943	0.3700220
C	0.2883296	-0.8979302	0.8997954
H	-2.1297643	2.9042693	0.4659875
H	-0.7360063	4.7927303	-0.3290816
H	1.7040701	4.4738189	-0.7231184
H	2.7576687	2.2735553	-0.4146344
H	0.4070108	-1.9358758	1.1822416
C	2.6346549	-0.6559805	0.0360379
C	3.3648870	-0.2568944	-1.1089566
C	3.1871118	-1.6760463	0.8492419
C	4.6001975	-0.8263554	-1.4037125
H	2.9373523	0.4745243	-1.7857985
C	4.4250301	-2.2388710	0.5527694
C	5.1408863	-1.8164672	-0.5730791
H	5.1381934	-0.5104340	-2.2929283
C	-2.6140799	-0.5964044	0.0418595
C	-2.6530191	-0.3603127	-1.3439174
C	-3.6418606	-1.3438206	0.6445213
C	-3.7028479	-0.8621817	-2.1123367
H	-1.8616331	0.2153732	-1.8150207
C	-4.6837651	-1.8552440	-0.1314110
H	-3.6207569	-1.5017386	1.7178088
C	-4.7171724	-1.6145381	-1.5083193
H	-3.7299405	-0.6702468	-3.1810936
H	-5.4744212	-2.4319040	0.3400344
P	-1.2254930	-0.0088564	1.0665377
O	-1.7196883	-0.0062533	2.5352242
H	-5.5316295	-2.0085258	-2.1095348
H	4.8353942	-3.0071087	1.2017152
H	2.6499903	-1.9944634	1.7370402
H	6.1033689	-2.2610439	-0.8080241

5e-S_1

Symbol	X	Y	Z
C	-0.0395902	1.6069635	0.1371562
C	-1.4755559	1.5262642	0.3632911
C	-2.2867903	2.6500951	0.1355163
C	-1.7252694	3.8292588	-0.3373168
C	-0.3210354	3.9224053	-0.5418460
C	0.5050872	2.8337994	-0.3034516
C	0.6397909	0.3853167	0.4605034
C	-0.2249499	-0.6054686	1.0009760
H	-3.3556211	2.5957570	0.3255450
H	-2.3559927	4.6910173	-0.5335293
H	0.1116103	4.8642745	-0.8655284
H	1.5790155	2.9419086	-0.4216575
H	0.1005477	-1.5811007	1.3405772
C	2.0240245	0.0449575	0.1576009
C	2.6828494	0.5086033	-1.0130014
C	2.7555362	-0.8136862	1.0174387
C	3.9952969	0.1710334	-1.2802351
H	2.1346502	1.1166929	-1.7232665
C	4.0772799	-1.1560486	0.7635981
C	4.7109443	-0.6603148	-0.3904198
H	4.4961014	0.5184804	-2.1778408
C	-3.0649393	-0.9618951	0.0363689
C	-3.1097019	-0.7600422	-1.3541945
C	-3.9013632	-1.9318191	0.6171833
C	-3.9758444	-1.5131114	-2.1478975
H	-2.4691515	-0.0102598	-1.8095965
C	-4.7567162	-2.6926878	-0.1818888
H	-3.8844548	-2.0634458	1.6944509
C	-4.7967208	-2.4846828	-1.5644369
H	-4.0105477	-1.3435503	-3.2204825
H	-5.4004921	-3.4391091	0.2751113
P	-1.9122917	-0.0379772	1.1117958
O	-2.4391178	-0.1138273	2.5480013
H	-5.4674068	-3.0729743	-2.1844613
H	4.6046342	-1.7980767	1.4585958
H	2.2795221	-1.1833963	1.9196885
O	5.9875442	-0.9324815	-0.7442077
C	6.7791675	-1.7838466	0.0867589
H	6.3340417	-2.7822183	0.1625194
H	7.7495352	-1.8515224	-0.4043197
H	6.9019729	-1.3529128	1.0866901

5h-S_1

Symbol	X	Y	Z
C	0.4441724	1.4721293	0.1381185
C	-0.9955560	1.5326271	0.3525286
C	-1.6916710	2.7300016	0.1188342
C	-1.0135355	3.8474625	-0.3503499
C	0.3949103	3.8030194	-0.5434066
C	1.1094788	2.6401403	-0.2980813
C	0.9984641	0.1903270	0.4636090
C	0.0347815	-0.7110795	0.9985859
H	-2.7621463	2.7808393	0.2999103
H	-1.5554226	4.7662086	-0.5532101
H	0.9194010	4.6982170	-0.8635972
H	2.1897578	2.6430666	-0.4064379
H	0.2594039	-1.7145494	1.3382035
C	2.3478976	-0.2780557	0.1739500
C	3.0597402	0.1252376	-0.9856879
C	2.9839459	-1.2046299	1.0402697
C	4.3406955	-0.3326538	-1.2448587
H	2.5777898	0.7832361	-1.6995611
C	4.2685024	-1.6650826	0.7915994
C	4.9565824	-1.2268377	-0.3512169
H	4.8787551	-0.0287668	-2.1365755
C	-2.8328851	-0.7805384	0.0327844
C	-2.8529381	-0.5863375	-1.3595340
C	-3.7725966	-1.6462014	0.6205603
C	-3.7968931	-1.2443259	-2.1485160
H	-2.1314307	0.0826445	-1.8196505
C	-4.7070485	-2.3128002	-0.1740851
H	-3.7703864	-1.7713552	1.6986505
C	-4.7223201	-2.1124879	-1.5581746
H	-3.8110500	-1.0816805	-3.2225617
H	-5.4302271	-2.9793243	0.2875163
P	-1.5846370	0.0192296	1.0984381
O	-2.1150390	0.0021882	2.5388602
H	-5.4541053	-2.6270946	-2.1745451
H	4.7431828	-2.3594230	1.4811112
H	2.4670646	-1.5310569	1.9366152
O	6.2133365	-1.6431720	-0.6581869
H	6.5402262	-2.2610225	0.0106011

5j-S_1

Symbol	X	Y	Z
C	0.4945072	1.3876703	-0.0949865
C	-0.9046805	1.5629028	0.2029894
C	-1.6071258	2.7235684	-0.1242345
C	-0.9476994	3.7441686	-0.8083490
C	0.4036504	3.5714505	-1.1670438
C	1.1114301	2.4178093	-0.8332166
C	1.0248704	0.0952585	0.3073536
C	0.0172190	-0.7830354	0.7942689
H	-2.6554919	2.8265628	0.1398310
H	-1.4733989	4.6555357	-1.0731868
H	0.9090034	4.3543902	-1.7256914
H	2.1390380	2.3122262	-1.1615136
H	0.2152940	-1.7615218	1.2100790
C	2.4154470	-0.3210940	0.1472779
C	3.4976022	0.5766950	0.3105268
C	2.7329408	-1.6733181	-0.1279344
C	4.8206320	0.1571580	0.1865945
H	3.3034709	1.6067321	0.5870739
C	4.0505740	-2.1059871	-0.2557452
C	5.0747615	-1.1790107	-0.1006429
H	5.6471043	0.8457162	0.3275367
C	-2.9062759	-0.8210914	0.1855219
C	-2.6833951	-1.7732526	-0.8235585
C	-4.2227174	-0.5080969	0.5704709
C	-3.7649116	-2.3993705	-1.4427637
H	-1.6663727	-2.0098213	-1.1195892
C	-5.3001199	-1.1324202	-0.0608874
H	-4.3928694	0.2058439	1.3683668
C	-5.0732230	-2.0780812	-1.0645577
H	-3.5875635	-3.1342401	-2.2223054
H	-6.3147954	-0.8890377	0.2400107
P	-1.4941226	0.0433811	0.9408415
O	-2.0894245	0.5009405	2.3537072
H	-5.9131492	-2.5662510	-1.5502467
H	4.2878715	-3.1404650	-0.4807922
F	6.3665744	-1.5941761	-0.2241694
H	1.9290080	-2.3878661	-0.2708314

5n-S_1

Symbol	X	Y	Z
C	-0.2682689	1.7671431	-0.1638545
C	-1.6793909	1.6669233	0.0888549
C	-2.5663652	2.5765783	-0.4569719
C	-2.0650237	3.5886075	-1.2903964
C	-0.6852043	3.6714135	-1.5841850
C	0.2137153	2.7701758	-1.0364430
C	0.5355700	0.7502038	0.5039401
C	-0.2505446	-0.1477114	1.2262715
H	-3.6305442	2.5082214	-0.2554207
H	-2.7473779	4.3080390	-1.7320703
H	-0.3305662	4.4487096	-2.2526991
H	1.2692599	2.8199130	-1.2689197
H	0.1752503	-0.9589299	1.8048502
C	2.0007489	0.6420258	0.3999189
C	2.8374673	1.7688028	0.5570989
C	2.5417704	-0.6249733	0.1510230
C	4.2211942	1.5861341	0.4593747
H	2.4307190	2.7388212	0.8210060
C	3.9294685	-0.7816376	0.0052752
C	4.7694266	0.3372529	0.1733809
H	4.8826872	2.4341980	0.6118100
C	-2.8535508	-1.0473803	0.2126382
C	-2.3848222	-1.4615332	-1.0479836
C	-3.9853296	-1.6536789	0.7806055
C	-3.0511469	-2.4742447	-1.7355292
H	-1.5016510	-1.0078375	-1.4889784
C	-4.6483828	-2.6642707	0.0832267
H	-4.3293469	-1.3298955	1.7579643
C	-4.1821822	-3.0736969	-1.1705459
H	-2.6851228	-2.8006613	-2.7035889
H	-5.5237328	-3.1351485	0.5195044
P	-2.0208840	0.2364522	1.1811030
O	-2.6790279	0.5446759	2.4949489
H	-4.6963659	-3.8659215	-1.7061577
H	1.9270443	-1.5068910	0.0177419
H	5.8381216	0.1886855	0.0801814
N	4.4582886	-2.0537333	-0.2958512
O	5.7302011	-2.1643808	-0.4066072
O	3.6303232	-3.0288897	-0.4364673

References:

[1] Duan, Z.; Hu, W.; Li, E-Q.; Mathey, F.; Concise Synthesis of phospholene and its *P*-strereogenic derivatives. *J. Org. Chem*. **2020**, *85*, 22, 14772-14778.
[2] Nishimura, K.; Hirano, K.; Miura, M. Direct synthesis of dibenzophospholes from biaryls by double C–P bond formation via phosphenium dication equivalents. *Org. Lett.* **2020**, *22,* 8, 3185–3189.
[3] Ponikiewski, Ł.; Sowa, S. Ring opening of triflates derived from benzophospholan-3-one oxides by aryl Grignard reagents as a route to 2-ethynylphenyl(diaryl)phosphine oxides. *J. Org. Chem.* **2021**, *86*, 14928−14941.
[4]Valeur, B., Berberan-Santos, M. N. *Molecular fluorescence: principles and applications.* John Wiley & Sons, **2012**.
